# An improved human memory algorithm with multi-directional and chaotic approaches for global optimization and energy-efficient cluster head selection in WSNs

**DOI:** 10.1038/s41598-026-61131-3

**Published:** 2026-07-16

**Authors:** Mahmoud Abdel-Salam, Wael A. Gab-Allah, Eman Mohamed Eldaydamony, Ahmed Atwan

**Affiliations:** Information Technology Department, Faculty of Computers and Information, Mansoura University, Mansoura, 35516 Egypt

**Keywords:** WSN, Human memory algorithm, Cluster head selection, Adaptive parameters, Chaotic learning, Energy science and technology, Engineering, Mathematics and computing

## Abstract

Wireless Sensor Networks (WSNs) play a crucial role in infrastructure monitoring across domains such as smart grids, industrial automation, and environmental sensing. However, energy efficiency remains a key challenge due to the limited battery life of sensor nodes. This work addresses the energy-efficient cluster head (CH) selection problem, formulated as a dynamic optimization task. Human Memory Optimization (HMO) is chosen as the foundation for its low memory footprint and adaptive learning, which align well with WSN constraints. Its memory-based recall mechanism naturally balances exploration and exploitation with minimal parameter tuning, making it suitable for decentralized CH decisions. On the other hand, traditional metaheuristic algorithms (MAs) suffer from premature convergence and fall into local optima, which makes them less effective in addressing the dynamic and energy-sensitive nature of CH selection in WSNs. To overcome these challenges, this work develops an improved version of the HMO, named Adaptive Enhanced Human Memory Optimization (AEHMO). The proposed AEHMO integrates four targeted strategies tailored for the WSN CH selection context: (1) Adaptive parameters dynamically adjust exploration and exploitation phases, enabling the algorithm to respond to changing node energy levels and topological shifts; (2) the Multi-Directional Mutation Strategy (MDMS) increases the diversity among CH candidates, ensuring broader spatial coverage and preventing clustering imbalances; (3) Dynamic Drift Search (DDS) enhances global exploration, allowing AEHMO to discover energy-efficient CH configurations across the entire network field early in the process; and (4) Chaotic Reverse-based Learning (CRL) introduces structured randomness to help the algorithm escape from suboptimal CH arrangements that could otherwise lead to rapid energy depletion or coverage holes. AEHMO was validated on the CEC2017 benchmark suite, showing superior performance in high-dimensional optimization. Applied to WSN CH selection, it achieved an average energy consumption of 0.395 J, FND at 1150 rounds, HND at 1853, and LND at 3136 rounds in the 150-node scenario. Furthermore, AEHMO was validated under a large-scale real-world heterogeneous WSN scenario comprising 1200 sensor nodes with a three-tier node architecture and a mobile sink strategy, achieving an FND of 362 rounds, HND of 1903 rounds, and LND of 56,946 rounds—outperforming all competing algorithms in total network lifetime. These results confirm AEHMO’s superiority in energy efficiency, scalability, and adaptability under both homogeneous and heterogeneous dynamic WSN conditions.

**Supplementary Information:**

The online version contains supplementary material available at 10.1038/s41598-026-61131-3.

## Introduction

Wireless Sensor Networks (WSNs) provide an efficient, promising, and cost-effective way for industries to perform infrastructure monitoring and management. A WSN may consist of hundreds of small sensor nodes with processing and wireless communication capabilities. The sensing nodes, also called motes, are distributed within various environments for application domains such as smart grids, industrial automation, military operations, and environmental monitoring^1^. Recent advancements in electronic components have addressed many of the challenges related to sensor manufacturing with respect to power consumption, weight, and cost. However, for most researchers, the energy conservation remains one of the most important goals and issues^2^. Most of these sensors are powered by non-rechargeable batteries, hence putting a limit on the lifetime of the overall network^3^. Different definitions have been considered to study the network lifetime, referring to (1) the time when a certain percentage of nodes fail, (2) the instant when the first node exhausts its energy, or (3) the moment when the last node becomes inoperative^4^. Indeed, once the first node fails, the network’s performance degrades. The interest is towards connectivity, coverage, and lifetime of each node to ensure longer operation time. Although some WSNs use renewable energy sources to extend the lifetime of sensor nodes, careful management of the available energy is still vital for maximizing the operational period of the network^5^. Various environmental parameters monitored by different WSNs include humidity, temperature, and location, which generally require aggregating the data from sensors in the vicinity owing to the high correlation in the measured values. Data processing could require even more energy compared to the energy needed to transmit, hence further justifying data compression^6^. On the other hand, energy is significantly reduced by aggregating the data before transmission hence extending the network’s operation life^7^.

The motes rapidly get exhausted since the data from every node has to be directly sent to the base station (BS)^8^. Mote failure results in network failure. Indeed, it is assumed that the poor power resources of motes are one of the major issues found in WSN. Energy management is very important to extend the lifetime of the WSN^9,10^. Thus, clustering techniques are widely used in all those applications where sensor nodes have to directly transmit data to the BS due to the rapid depletion of energy. In a clustering configuration, the sensor nodes are grouped into clusters, and a CH is assigned for each cluster^11^. CH is responsible for intra-cluster communication and transmission of data to the BS, thereby reducing the energy expenditure of an individual node by a great amount, hence enhancing network scalability^12^. Optimal selection of CHs is one of the most complex and dynamic problems. Classic clustering schemes^13^, while being effective in practical implementation, suffer from several disadvantages when applied to energy-constrained scenarios. These approaches mostly suffer from initial conditions and likely converge to local optima due to poor exploration of the solution space. Furthermore, most of them are static and cannot adapt to the changing network conditions, which may lead to unequal energy distribution and lead to a network lifetime reduction^14,15^.

Therefore, recent studies have proposed competitive solutions by adopting MAs, which try to select the CHs and get a higher success rate for network reliability. The use of MAs has become a very popular technique in finding a solution to complex optimization problems since they offer adaptability, flexibility, and efficiency to explore large, diverse, and complex search spaces. MAs take their inspiration from natural phenomena, such as biological evolution, group dynamics, and physical processes, and often perform better than the conventional approaches in cases where the latter fails, especially for high-dimensional problems or avoiding local optima. In this respect, different established MAs have been tried to avoid local optima issues by providing novel solutions to enhance WSN by replacing traditional approaches with efficient ones for solving problems in real-time^7,13,14,16–19^. Besides, MAs were successfully applied for a variety of purposes including feature selection^20,21^, image segmentation^22,23^, engineering applications^24^, intrusion detection^25^, urban traffic with the help of adaptive systems^26^ and even enhancement of agricultural predictions, including crop yield estimation^27^.

The HMO algorithm takes its inspiration from the human cognitive process making it a very promising algorithm since it is simple, robust, and requires relatively fewer parameters when compared to other MAs^28^. Additionally, the HMO proved a good performance in solving many complex, high dimensional and non-linear issues^28^. Moreover, HMO appropriately balances exploration and exploitation through the simulation of recalling in human memory within the process of searching. However, HMO has a couple of weaknesses regarding complex and high-dimensional optimization problems, such as WSN energy management applications. The parameters of the original HMO, which control the exploration and exploitation by fixed parameters $\:b$ and $\:\delta\:$, respectively, may result in premature convergence or poor adaptability to evolving optimization landscapes. Besides, dependence on the best solution at each iteration increases the lack of diversity within the algorithm and enhances the possibility of falling into local optima. Furthermore, the limited exploration and late exploitation ability of HMO may lead to poor performance in the CH selection problem in WSN and hence obtaining a shorter network lifespan. These limitations, together with the computational overhead due to the memory management system and heavy parameter tuning of HMO, motivate the search for a better alternative approach.

To overcome these limitations, this paper presents an improved version of HMO, namely AEHMO, where the contribution lies in adaptively regulating and strengthening the original recall-driven search rather than introducing recall itself. In the proposed AEHMO, the fixed parameters $\:b$ and $\:\delta\:$ were replaced by novel adaptive parameters $\:{w}_{1}$ and $\:{w}_{2}$ that vary dynamically during the progress of iterations in the optimization process. This modification enables the algorithm to pay more attention to global exploration in its early stages and smoothly shift to local exploitation in later stages, enhancing convergence speed and search efficiency. Furthermore, AEHMO introduces the MDMS strategy which combines Gaussian and Cauchy mutations in an attempt to balance the step sizes of individuals during the optimization of the CH selection process. This strategy ensures more diverse exploration in the early stages and fine-tunes the search process as the iteration of the algorithm progresses, reducing the possibility of premature convergence. Further, the DDS is then utilized to strengthen global exploration during the initial phases of optimization for visiting new search regions while searching for the optimal CHs. Hence, it helps the algorithm to explore higher solution space and avoid falling into the traps of local optima. Lastly, the CRL strategy introduces structured randomness into the search process by incorporating chaotic mapping into the opposition-based learning framework. This strategy enhances the algorithm’s capability of escaping from local optima and further enhances the global search capability by maintaining diversity and preventing the solution from falling into local solutions. These four strategies are not arbitrarily combined; rather, they are designed as complementary mechanisms operating at different structural levels of the search process. The adaptive parameters regulate the global exploration–exploitation balance, DDS and CRL preserve population diversity and prevent premature convergence, and MDMS performs controlled intensification around elite solutions, forming a theoretically coordinated multi-layer search framework.

This paper validated the proposed AEHMO algorithm using the benchmark functions from CEC2017 suite to assess its applicability for global optimization and generalizability. The experimental results show that this approach has an excellent performance in finding the global optimum of complex and high-dimensional optimization problems. Then, the WSN CH selection problem is conducted using the proposed AEHMO to minimize energy consumption, extend lifetime, and flexibly adapt to the evolutionary conditions of the networks tested. The results show that AEHMO outperforms advanced, recent, and state-of-the-art algorithms in terms of energy efficiency and network resilience, providing a significant enhancement in convergence speed and solution quality. Therefore, the contributions of this paper can be concise as follows:

1. To conduct a background study and literature review related to the energy-efficient CH selection in WSNs. This identifies shortcomings in existing methods in order to optimize the prolongation of network lifetime with reduced energy consumption.
2. To propose a novel algorithm, named AEHMO, for energy-efficient CH selection in WSNs and global optimization. In AEHMO, adaptive parameters, MDMS, DDS, and CRL strategies are introduced to enhance exploration and exploitation providing more diversity and adaptability in dynamic network conditions.
3. To test and validate the proposed AEHMO through benchmark functions from CEC2017 for global optimization, which validate the effectiveness of this proposed methodology for different and complex optimization problems. Further validation of AEHMO is applied through the applicability of AEHMO to the CH selection problem in WSN which validates the effectiveness of the proposed algorithm for energy consumption, network lifetime, and scalability.
4. To compare the proposed AEHMO with existing algorithms for global optimization and CH selection energy-efficient performance. The comparisons highlight that AEHMO has better energy efficiency and longer lifetime of a network, as well as higher adaptability to meet the challenges in WSN optimization and global optimization.

The rest of the paper is organized as follows: the section “Related work” represents the related work on energy-efficient CH selection in WSNs. The section “Human memory optimization algorithm” presents a mathematical presentation of the HMO algorithm and related terminologies. Section “The proposed AEHMO algorithm” explains the proposed algorithm AEHMO, the strategies behind it, and its computational complexity. The section “AEHMO for global optimization” represents the performance evaluation of AEHMO with CEC2017 benchmark functions over global optimization. Section “Application of AEHMO for cluster CH selection in cluster-based routing for wireless sensor networks (WSN)” applies and evaluates AEHMO for the CH selection problem in WSNs. Finally, Sect. “Conclusion and future directions” concludes the paper and discusses the future research direction.

## Related work

Recent works in WSNs have focused on energy efficiency, optimized CH selection, and enhancement of data transmission. Several schemes were proposed to address the main challenges faced by WSNs in managing resources to keep the network alive as long as possible. This section reviews these approaches and describes their contributions and limitations as shown in Table 1. It also highlights the motivational gaps that drive the development of the AEHMO algorithm.

Somula et al.^29^ proposed the SWARAM algorithm, which is a contribution toward optimization in CH selection to improve energy efficiency within IoT networks, with a claimed 10% improvement over other methods. Liu et al.^30^ proposed a dual-CH model using a hybrid Grey Wolf Optimization (GWO) with Whale Optimization Algorithm (WOA) named as (GWOA-CH); this enhanced energy efficiency by the fusion of the GWO and WOA algorithms. Their work focused on CH re-election to prevent premature node depletion.

Kathiroli and Selvadurai^31^developed a hybrid clustering method using Salp Swarm Algorithm (SSA) and Differential Evolution (DE) algorithms. It enhanced the efficiency of node energy and network lifetime. Sharada et al.^32^ proposed Adaptive Ant Colony Distributed Intelligent based Clustering algorithm AACDIC to enhance energy efficiency in cognitive radio systems by reducing power consumption by 9.646% and enhancing accuracy in spectrum sensing.

The method proposed by Manoharan et al.^33^ employed Density-based Adaptive Soft (DAS) clustering and Entropy value based Bald Eagle Search (EBES) optimization to enhance throughput and packet delivery. Sahayaraj et al.^34^ proposed an Improved Dingo and Boosted Beluga Whale Optimization Algorithm (IDBBWOA) optimization algorithm for the selection of CH and routing in WSNs, hence enhancing throughput and prolonging network life by 18.92%.

In^16^, Saemi and Goodarzian proposed a Global Search Local Search (GSLS) hybrid MA for energy-efficient routing in UWSNs that outperformed the other works in pathfinding and energy consumption. Also, there is an energy-efficient hybrid IoT-enabled WSN clustering protocol called K-LionER that was proposed by Rekha and Garg^35^, which improved the network lifetime bound between 10 and 48%. Houssein et al.^36^ extended Sea Horse Optimization (SHO) with opposition-based learning for preventing stagnation and improving stability in WSNs; El Khediri et al.^37^ proposed a hybrid Artificial Bee Colony (ABC) and Ant Colony Optimization (ACO) named as ABC-ACO for CH selection, increasing the lifetime of the network by 40.5% and improving energy consumption.

Roberts et al.^38^ proposed a hybrid Zebra Fish Optimization (ZFO) and Sea Horse Optimization (SHO) named as ZFO-SHO algorithm applied for WSN CH selection, improving the throughput by up to 24% and enhancing network lifetime. Hu et al.^39^ proposed Quantum Particle Swarm Optimization with Fuzzy Logic (QPSOFL), introducing quantum particle swarm optimization with fuzzy logic to enhance the energy efficiency and scalability of the network. Yang et al.^40^ proposed the Multi-Strategy fusion Snake Optimizer (MSSO) protocol for WSNs. These authors improved energy efficiency by 26.64% and extended the network stable period using dynamic updating parameters.

Huang et al.^41^ proposed HBWO, which is a hybrid algorithm of Beluga Whale Optimization algorithm combined with quasi-oppositional learning, adaptive spiral predation, and Nelder-Mead local refinement for the improvement of convergence speed. The method was tested for CEC2017 and CEC2019 benchmark functions, for a number of engineering design problems. Ran et al.^42^ proposed HGA-FACO, an automatic K-means clustering scheme with genetic operators, ant colony search, adaptive fuzzy control and noise handling to eliminate the need of predefined cluster numbers and initial centers. The method was validated on large-scale urban GPS data from several cities and compared with several intelligent variants of K-means.

Qiao et al.^43^ proposed NDWPSO which is an improved particle swarm optimization algorithm by incorporating elite opposition-based initialization, dynamic inertia weighting, local escape mechanism and hybrid spiral mutation strategies inspired by WOA and DE to address the premature convergence problem. The algorithm was tested on 23 benchmark functions and three engineering design problems against eight well-known metaheuristic algorithms. Yıldız et al.^44^ proposed AOA-NM, a hybrid optimizer which embeds the Nelder–Mead local search into the Arithmetic Optimization Algorithm in order to improve the convergence accuracy and avoid local stagnation. The method was evaluated on CEC2020 benchmark functions and several constrained engineering design and manufacturing problems.

Several recent works have addressed energy efficiency and security in WSNs through hybrid bio-inspired frameworks. Lonkar and Karmore^10^ conducted a statistical evaluation of power-aware routing protocols for wireless networks, establishing a foundational comparative framework that highlighted the trade-offs between energy efficiency and communication performance across different routing strategies. Building upon this foundation, Lonkar and Karmore^45^ proposed BCEWN, a hybrid bio-inspired clustering model integrating GWO and PSO for energy-aware wireless network deployment, where GWO performs node clustering and PSO optimizes routing paths. The method was validated on heterogeneous wireless network scenarios and achieved superior deployment performance in terms of energy efficiency and communication quality. Subsequently, Lonkar et al.^46^ investigated hybrid CH selection strategies through a comprehensive comparative empirical framework evaluating multiple clustering techniques across several network performance metrics including energy consumption, delay, and network lifetime, demonstrating improvements in network energy efficiency. Lonkar and Karmore^47^ proposed EBBSMWN, a blockchain-enabled bio-inspired framework combining Ant Lion Optimization and Elephant Herding Optimization to enhance both the security and energy efficiency of wireless networks, demonstrating improvements in energy consumption, communication delay, and network reliability under multiple cyberattack scenarios. Most recently, Lonkar et al.^15^ presented a comprehensive review of hybrid clustering and routing protocols specifically addressing the hotspot problem in wireless sensor networks, comparing representative methods based on energy consumption, delay, routing overhead, and network lifetime. Despite these significant contributions, these works share several common limitations. First, the hybrid structures employed tend to increase computational complexity, making them less suitable for resource-constrained WSN deployments with strict energy budgets. Second, parameter configurations in these frameworks remain largely static and are not dynamically adjusted in response to evolving network conditions such as changing node energy levels and topological shifts. Third, none of these approaches employ an explicitly coordinated mechanism that governs the transition between exploration and exploitation phases during the optimization process, which can lead to premature convergence or suboptimal cluster head configurations. These gaps directly motivate the design of AEHMO, which addresses them through adaptive parameter regulation, structured diversification via MDMS and CRL, and a memory-driven search framework that dynamically responds to changing network conditions without significantly increasing computational overhead.

**Table 1:** Summarized related work.

Study	Main methodology	Advantages	Limitations
Somula et al.^29^	SWARAM algorithm with osprey optimization for CH selection	Improves network lifetime and packet delivery by 10%	Limited adaptability to dynamic network environments; sensitive to improper CH selection
Liu et al.^30^	Dual-CH clustering model, GWOA-CH algorithm	Increases energy efficiency by combining GWO and WOA	Premature node death still occurs; relies heavily on residual energy and distance
Kathiroli & Selvadurai^31^	SSA + DE hybrid for optimal CH selection	Improves alive nodes, dead nodes, and throughput	Lacks dynamic parameter tuning, limiting adaptability to different network conditions
Sharada et al.^32^	AACDIC algorithm for energy efficiency in CR systems	Reduces power consumption by 9.646%, improves SNR to 2 dB	High computational cost; may not scale efficiently with large sensor networks
Manoharan et al.^33^	DAS clustering and EBES optimization	Improves throughput, energy efficiency, and packet delivery ratio	Complex entropy calculation for data transmission; computationally expensive
Sahayaraj et al.^34^	IDBBWOA algorithm for energy-efficient routing	Increases throughput by 18.92%, prolongs network lifespan	The hotspot problem persists; lack of exploration-exploitation balance in routing
Saemi & Goodarzian^16^	GSLS hybrid for UWSNs	Lowers energy consumption, improves pathfinding efficiency	Limited to underwater applications; may not generalize well to terrestrial WSNs
Rekha & Garg^35^	K-LionER hybrid clustering for IoT-enabled WSNs	Extends network lifespan by up to 48%, improves stability	High sensitivity to initial CH selection; lacks robustness in highly dynamic environments
Houssein et al.^36^	SHO-OBL algorithm with opposition-based learning for WSNs	Prevents algorithm stagnation, improves residual energy and stability	Convergence speed is still an issue; heavily dependent on the opposition-based strategy
El Khediri et al.^37^	ABC + ACO hybrid for energy-efficient CH selection	Increases network lifetime by 40.5%, improves energy consumption	Requires frequent updates of ACO pheromone trails, leading to high computational overhead
Roberts et al.^38^	ZFO + SHO hybrid for CH selection in WSNs	Increases throughput by 6.7%–24%, improves network lifetime	Complex parameter tuning and high computation time due to hybrid nature
Hu et al.^39^	QPSOFL with fuzzy logic for energy-efficient routing	Superior accuracy and convergence speed use Sobol sequences	Fuzzy logic-based next-hop selection may be prone to inefficiencies in large-scale networks
Yang et al.^40^	MSSO with dynamic updates and FCM for energy efficiency	Reduces energy consumption by 26.64%, improves stable period by 52.43%	High computational cost due to multi-strategy fusion; limited adaptability to different WSN topologies

Based on the comparative analysis in Table 1, it is evident that recent approaches have achieved notable improvements in energy efficiency, throughput, and network stability through hybridization, adaptive updating, and opposition-based mechanisms. These contributions have significantly advanced CH selection strategies in WSNs. However, despite these strengths, several challenges remain partially addressed. In particular, many approaches rely on fixed or loosely adaptive control parameters or combine multiple operators without an explicit coordination mechanism governing exploration–exploitation transition. Moreover, as hybrid structures become more complex, scalability and computational overhead may increase, especially in dynamic network conditions. Therefore, there remains room for designing a more structurally integrated framework that adaptively regulates search behavior while maintaining computational efficiency. Unlike existing CH selection approaches that are mainly based on swarm-motion or hybrid search operators, AEHMO is based on the original memory recall paradigm of HMO and improved by adaptive regulation and structured diversification mechanisms. The novelty of AEHMO is not the introduction of memory recall, but the reconfiguration of the control of memory recall intensity, memory influence, and memory diversification in a dynamic manner through adaptive parameters, multi-directional mutation, drift-based exploration, and chaotic opposition. This leads to the emergence of a coordinated memory-driven search framework instead of a recall mechanism.

Compared with existing MH-based CH selection methods, AEHMO dynamically adjusts its parameters, to continuously balance the exploration with the exploitation to avoid early convergence and ensure adaptability to changing network conditions. MDMS enhances the exploration by enabling AEHMO to explore several regions, so it can avoid local minima, ensuring space is explored more intensively. DDS strengthens the global search in the initial periods of operation; hence, AEHMO is able to adapt to WSN dynamics and avoid early convergence. Besides, CRL enhances exploration and accelerates convergence by chaotic mapping; hence, it solves the issues of computational overhead and provides scalability. After applying these strategies, AEHMO improves the trade-off between exploration and exploitation, thus providing more energy-efficient routing in WSNs. For cluster-based routing and CH selection, AEHMO shows a strong solution with adaptation to dynamic conditions and reducing energy consumption to extend the network lifetime and address the gaps that previous works left unresolved. In addition, the presence of local minima is avoided in order to ensure the optimal CH selection and the energy usage is efficiently distributed over the network by AEHMO. For dynamic environments, AEHMO can handle such dynamics of the network by continuous adaptation to changes in node positions and their energy levels.

## Human memory optimization algorithm

Human Memory Optimization (HMO) draws its inspiration from the fact that humans rely on their memory when making complex decisions. Humans revisit past decisions and their outcomes, repeating successful actions and avoiding repeated mistakes. This cognitive process is mirrored in the HMO algorithm, which guides the search for optimal solutions using memories of previously conducted iterations, managing exploration and exploitation effectively.

### Initialization

The initialization phase forms the starting point for the algorithm, through which a population of possible solutions is created in a diverse manner. This diversity in initial positions is essential for thorough exploration of the search space. The position of each solution at the beginning is defined as follows:

1 $$\:{X}_{i}={l}_{b}+({u}_{b}-{l}_{b})\times\:\mathrm{r}\mathrm{a}\mathrm{n}\mathrm{d}(1,\mathrm{d}\mathrm{i}\mathrm{m})$$

where $\:{l}_{b}$ and $\:{u}_{b}$ are the lower and upper bounds of the search problem. The range between the lower and upper bounds influences the diversity of the initial population: the wider the range, the broader the exploration. Similarly, the term $\:rand(1,dim)$ provides randomness to the initialization of solutions; each solution starts from a different position within the bounds. This randomness is important to ensure diversity in order to avoid premature convergence.

### Memory production and update

In this phase, new candidate solutions (memories) are generated based on the collective experience of the population. This update rule of this phase is defined as:

2 $$X_{i}^{{{\mathrm{new}}}} = \overline{X} + \nabla \: \times \:(u_{b} - l_{b} ) \times \:{\mathrm{rand}}(1,{\mathrm{dim}})$$

3 $$\:\nabla\:=k\times\:\mathrm{s}\mathrm{i}\mathrm{n}\left(\frac{1}{k}\right)\times\:b$$

4 $$\:b=2\times\:\left(1-\frac{t}{T}\right)$$

In the above equations, $\overline{X}$ characterizes the average position of the population that plays the role of a central point guiding the search. $\:\nabla\:$ is the step-size adjustment, where $\:k$ is a random constant factor that determines the variability of the search to be performed in the range [0.1, 0.3], and $\:b$ is the time-varying coefficient whose value decays during the evolution process from 2 to 0. Smaller values of $\:k$ yield larger step sizes, favoring exploration of new regions, whereas larger values of $\:k$ reduce the step size, directing the algorithm toward the most promising areas. In this way, the coefficient $\:b$ is gradually reduced over time by a factor, smoothly but systematically biasing the algorithm’s focus from global exploration to local exploitation as termination is approached. This phase tries to balance wide exploration with the refinement of the search for the best solutions found.

In the HMO algorithm, events of memories are labeled as success and failure. The recorded events are stored in a matrix whose capacity is determined by the population size $\:N$. This memory retention mechanism is formalized in Algorithm 1.

**Algorithm 1:**
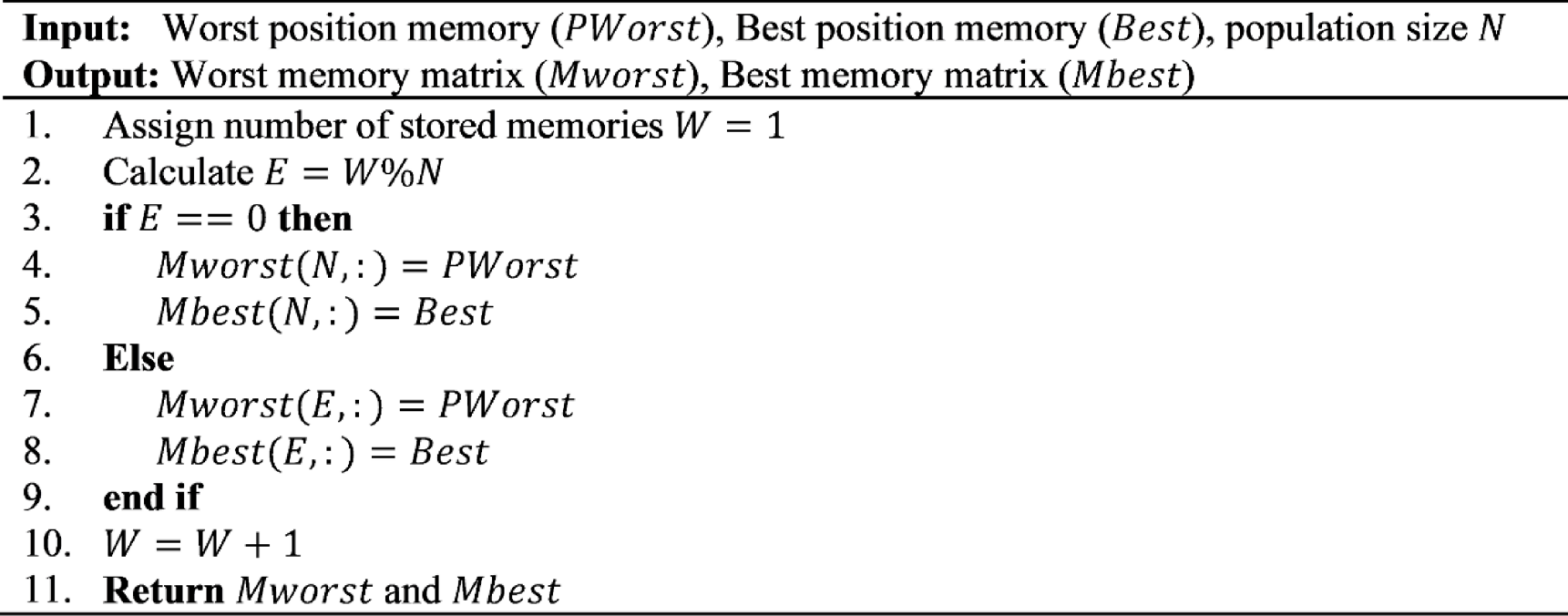
Strategies of memory storage.

### Memory recall behavior

Human beings consistently receive both failed and successful experiences in the setting of memory formation, which plays a significant role in dictating human actions. At this stage, the algorithm in memory recall is divided into five major categories, which are explained below:

- ***Recalling failure events***: To simulate the process of remembering the failed experiences, solutions are modified using Eqs. (5) and (6) in the algorithm:

5 $$X_{i}^{{{\mathrm{new}}}} = X_{{{\mathrm{best}}\:}} + \beta \: \times \:\left( {r_{1} \times \:W_{a} - r_{2} \times \overline{X} } \right)$$

6 $$\:\beta\:=-\frac{1}{\delta\:}\mathrm{l}\mathrm{o}\mathrm{g}(1-\phi\:)$$

where $\:\phi\:$ is a randomly generated vector of size $\:1\times\:D$, $\:{X}_{best}$ represents the best memory so far and $\:{X}_{w}$ is a randomly selected solution from the $\:Worst$ memory. Two random values ranging from 0 to 1, $\:{r}_{1}$ and $\:{r}_{2}$, act upon the emotional factor $\:\beta\:$. The value of $\:\delta\:$ is kept fixed at 4. In this phase, poor memories are updated more slowly, as the least favorable solutions have already been identified in earlier stages and further deterioration becomes increasingly unlikely. The position in the worst memory not being aligned with the position of the current population would, therefore, increase the possibility of global exploration.

- ***Recalling success events***: This step is important because the algorithm simulates the recall of successful experiences to balance individual search behaviors. Each solution is updated using Eq. (7), to simulate this step:

7 $$X_{i}^{{{\mathrm{new}}}} = X_{{{\mathrm{best}}\:}} + \beta \: \times \:\left( {r_{1} \times \:B_{{a2}} - r_{2} \times \overline{X} } \right)$$

In this equation, $\:{B}_{a2}$ is a randomly chosen solution from the $\:Best$ memory. Human memory tends to retrieve successful experiences from a large pool of past events, with distinctions among them diminishing over time. This leads to a rapid transition from broad exploration to a more focused exploitation phase during the algorithm’s runtime. $\:{B}_{a2}$ in the equation, leads the search into regions that turned out to be good in the past. As such, this phase favors local exploitation and enables the algorithm to fine-tune solutions around successful known regions.

- ***Recovering lost memories***: This step emulates the memory loss process, which encourages global search extensively. The mathematical description of retrieving lost memory can be given by using Eq. (8):

8 $$X_{i}^{{{\mathrm{new}}}} = X_{r} \left( t \right) + \sigma \: \times \:\left( {r_{1} \times \:\overline{X} - P_{{{\mathrm{worst}}\:}} } \right)$$

where $\:{X}_{r}$ denotes a randomly selected individual from the population, $\:{P}_{worst}$ is the most recent poor memory, and $\:{r}_{1}$ is a random number between 0 and 1. From Eq. (8), one could notice that the optimization is not obsessed with the successful events, but rather utilizes the current position of the population.

- ***Recalling present memories***: Individual solutions tend to access their current memories constantly, by which process their capability of local solution refinement can be significantly improved. The recollection process of the present memories can be simulated by Eq. (9):

9 $$\:{X}_{i}^{\mathrm{n}\mathrm{e}\mathrm{w}}={X}_{\mathrm{best\:}}+{r}_{2}\times\:\left({X}_{l}-{X}_{\mathrm{i\:}}\right)$$

In that respect, $\:{X}_{l}$ is the best solution within the current population and $\:{r}_{2}$ is randomly chosen between 0 and 1. Equation (9) emphasizes that the local optimal solutions are combined with the best-known solutions and kept over successive iterations.

- ***Greedy selection***: This will replace the best solution and the fitness value iteratively to make sure that the more the algorithm progresses, the more effective the global search gets.

10 $$\:{X}_{i}(t+1)=\left\{\begin{array}{ll}{X}_{i}^{\mathrm{new\:}},&\:\mathrm{\:if\:}f\left({X}_{i}^{\mathrm{new\:}}\right)\le\:f\left({X}_{i}\left(t\right)\right)\\\:{X}_{i}\left(t\right),&\:\mathrm{\:otherwise\:}\end{array}\right.$$

## The proposed AEHMO algorithm

In the study^28^, the HMO performance is tested against several traditional and newly developed intelligent algorithms on multiple benchmark functions. Another direction of application is to implement the HMO into various real-world applications, such as multilevel thresholding image segmentation, which thus demonstrates their efficiency in solving real-world optimization problems. These results confirm that HMO is effective for both theoretical benchmarks and practical optimization problems. However, HMO has some drawbacks concerning the fixed parameters $\:b$ and $\:\delta\:$, which reduce its adaptability throughout the search process. Fixed parameter values can cause premature termination of exploration in complex problems and decrease the flexibility to adapt to various optimization landscapes. Furthermore, HMO’s dependence on the current best solution across multiple phases limits exploration, increasing the risk of getting stuck in a local optimum, particularly in multi-modal problems. In later iterations, the algorithm tends toward insufficient exploitation, which in turn delays convergence. Additionally, the memory management system generally incurs extra computational overhead; further, the wide range of parameter tuning inhibits its application to varied optimization tasks.

The new variant of the HMO algorithm called AEHMO stands for Adaptive Enhanced Human Memory Optimization which adds to the original algorithm several strategies that may enable the surmounting of these limitations which adds four targeted strategies to overcome these limitations. First, it replaces the fixed parameters $\:b$ and $\:\delta\:$ by adaptive parameters $\:{w}_{1}$ and $\:{w}_{2}$, respectively, where $\:{w}_{1}$ transitions the algorithm from early global exploration **to** local exploitation through a Sigmoid function, and $\:{w}_{2}$ is used for improving the recall of failure memories in order to ensure better adaptability. A MDMS strategy is adopted that combines Gaussian and Cauchy mutations to balance exploration and exploitation more effectively. Third, DDS simulates irresistible motion to realize the intention of wider exploration in the early stage. Finally, the CRL strategy incorporates chaotic mapping into the algorithm for further enhancement of escaping from local optima. Each of the four strategies enhances the search efficiency, adaptability, and convergence speed of AEHMO for complex optimization problems. The next subsection explains the proposed strategies in more detail.

### Adaptive parameters modifications

In HMO, the two parameters control the global exploration and exploitation behavior of the original algorithm named $\:b$ and $\:\delta\:$ at Eqs. (4) and (6). These parameters can enhance and largely influence the capability of searching for the HMO toward the best global solutions. In this paper, these parameters are replaced by new adaptive parameters that can provide adaptive changes to the HMO with the progress of iterations that enhance the global exploration and local exploitation of HMO.

At the human activities phase of HMO, parameter $\:b$ is used to control the behavior of the human memory to retain the different memories. On the other hand, Eq. (4) shows that the parameter $\:b$ shows weak linearity with the number of iterations, which could be inefficient considering the substantial nonlinearity of the optimized process. Therefore, using a higher nonlinear convergence factor can improve the efficiency of the HMO algorithm. Our goal is to strengthen the HMO algorithm’s ability to explore globally in the early stages and exploit locally in the later stages, in order to boost the chances of identifying better locations and speed up convergence. Hence, it is necessary to modify the parameter in a manner that results in a gradual decline during the initial stages and a more pronounced decrease towards the later stages. To accomplish this, the suggested AEHMO incorporates an adaptable parameter $\:{w}_{1}$ as a replacement for the value $\:b$. The Sigmoid function is used to control the position updates of the HMO algorithm during the process, resulting in enhanced search efficiency of the algorithm. The Sigmoid function and the adaptive $\:{w}_{1}$ are defined as follows:

11 $$\:w1\left(t\right)=2\times\:(1-\frac{1}{1+{e}^{R\left(t\right)}})R\left(t\right)=-\frac{t-(\frac{1}{2}\times\:\mathrm{T})}{T}\times\:10$$

**Fig. 1:**
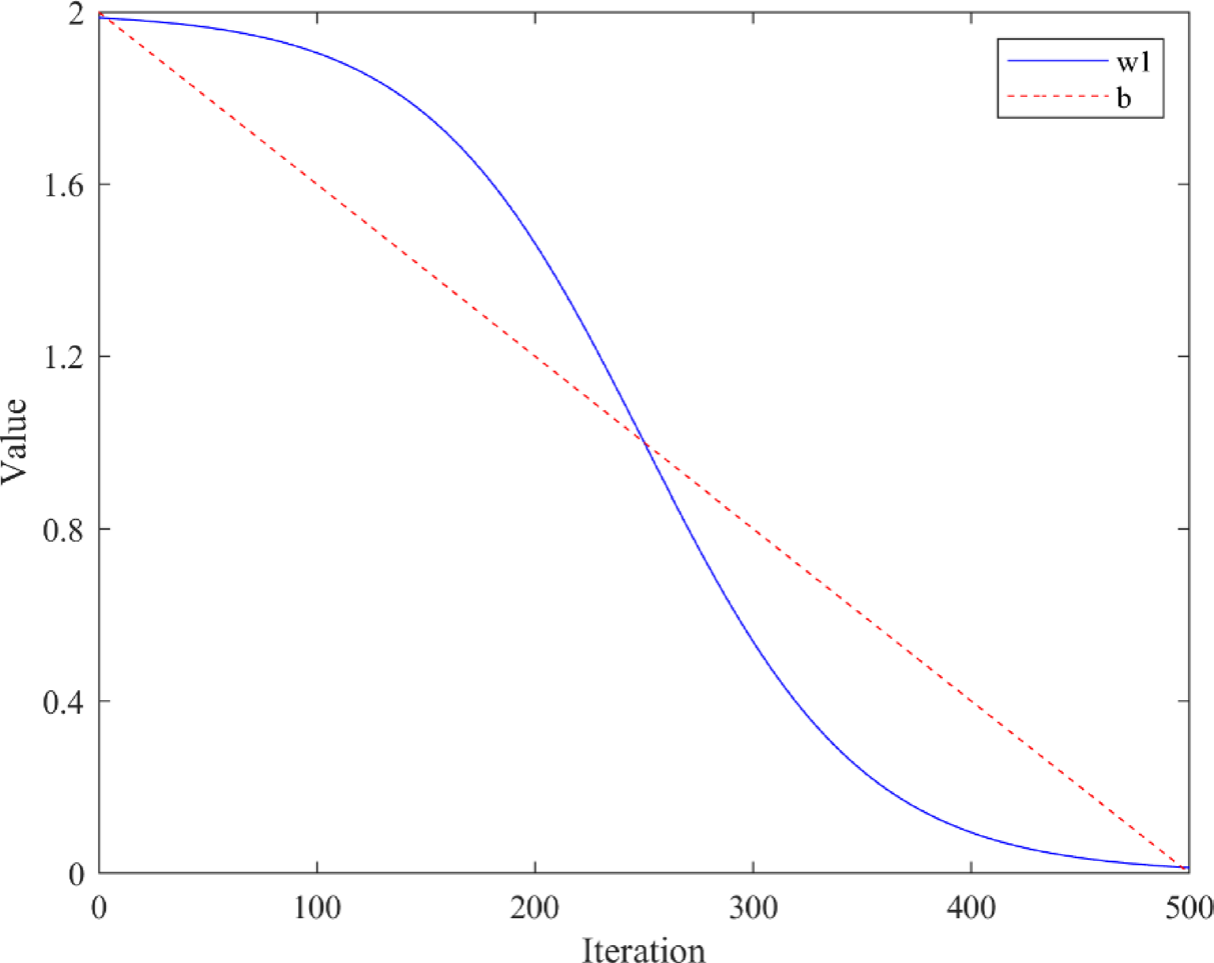
Changes of $\:b$ and $\:{w}_{1}$ with respect to the progress of iterations.

The Sigmoid function is selected deliberately for the following reasons. Unlike linear decay which leads to a constant reduction in exploration over time, the Sigmoid function gives a slow decay in the early iterations, a fast transition around the mid-point, and a smooth saturation in the later iterations. This S-shaped function is especially suitable for the balancing of exploration and exploitation as it maintains good global search capability in the early part of the process while allowing for a controlled and accelerated change over to exploitation around $\:t=T/2$. Moreover, the scaling factor in $\:R\left(t\right)$ was chosen so that the transition region is centered at the middle of the search process, so that the behavior will not change abruptly. Preliminary comparisons with linear and exponential decay schedules showed that Sigmoid-based control produced more stable and less oscillatory convergence behavior. Also, the Sigmoid function achieves a favorable equilibrium between linear and nonlinear attributes, exhibiting smooth upper and lower limits, as depicted in Fig. 1. In the initial stages of the optimization process, the value of $\:{w}_{1}$ remains close to its upper bound, promoting broad global search. In addition, the wider range of $\:{w}_{1}$ values during the initial stages help in preserving the diversity of HMO. Around the midpoint of the optimization, the value of $\:{w}_{1}$ falls quickly, which speeds up the transition from global to local search. During the later phases, the value of $\:{w}_{1}$ remains at a low level, which increases the length of time for local search and enhances the effectiveness of local search performed by various agents.

In addition, the parameter $\:\delta\:$ is set to a fixed value in the original HMO while retrieving the failure memories which limits the search ability of the agents to retrieve the failure memories. The parameter $\:\delta\:$ is a crucial parameter while updating the agents at this phase as mentioned in Eq. (6) which limits the movement of search agents through the search space hence, falling into the local optimum would be of great chance. Therefore, this paper proposes an adaptive parameter to improve the updating mechanism in Eq. (6) which enhances the exploration of HMO and avoids falling into the local optimum solutions. Hence, a new adaptive parameter $\:{w}_{2}$ is proposed to replace $\:\delta\:$ as follows:

12 $$\:{w}_{2}={\left(4-4\frac{t}{T}\right)}^{\left(1-\mathrm{t}\mathrm{a}\mathrm{n}\left(\frac{\pi\:t(r-0.5)}{T}\right)\right)}$$

The tangent component in $\:{w}_{2}$ is introduced to produce controlled nonlinear oscillatory modulation of the intensity of the recall. The tangent term, unlike the purely monotonic functions of decay, provides bounded variability that provides the opportunity to expand and contract the search step periodically during failure-memory retrieval. This mechanism gives more probability to get out of local optima by preventing the uniform contraction of the search radius. The structure of the exponent guarantees that the modulation effect fades away gradually as →, no loss of stability in the later iterations. Hence the tangent term is a controlled regulator of diversification and not a nonlinear mapping which is arbitrary. Hence the new position updating of recalling failure memories can be formulated as:

13 $$\:{X}_{i}^{\mathrm{n}\mathrm{e}\mathrm{w}}={X}_{\mathrm{best\:}}+\beta\:\times\:\left({r}_{1}\times\:{X}_{w}-{r}_{2}\times\:{\mathrm{X}}_{\mathrm{m}}\right)$$

14 $$\:\beta\:=-1/{w}_{2}\times\:\mathrm{l}\mathrm{o}\mathrm{g}(1-\phi\:)$$

where $\:{r}_{1}$ and $\:{r}_{2}$ are random numbers between − 1 and 1 and similarly $\:\phi\:$ is a random vector of values between − 1 and 1 to control the randomness of the search process. Therefore, the new updated search formula can provide a more adaptive search process that can discover new search regions and help avoid dipping into the local optimum.

From a cognitive perspective, the adaptive parameters $\:{w}_{1}$ and $\:{w}_{2}$ model the time-varying sensitivity of human memory, where attention and recall intensity naturally change as experience accumulates. Early stages emphasize exploratory recall and weaker memory filtering, while later stages reflect stronger consolidation and focused retrieval. This adaptive regulation preserves the core human memory paradigm by directly controlling how experiences are retained, recalled, or attenuated throughout the decision-making process.

### Multi-Directional Mutation Strategy (MDMS)

One of the major disadvantages of the original HMO algorithm is its inability to effectively utilize the best solutions in the population. It thus converges slowly and cannot efficiently adjust superior solutions in the last iterations. To enhance such a capability, MDMS is utilized with a special emphasis on applying mutations to 20–30% of the best solutions in the population. By targeting only individuals with superior fitness, MDMS enhances exploitation while maintaining a balance with exploration.

This strategy hybridizes Gaussian and Cauchy mutations to adaptively update the step sizes during optimization. In this variant, only the top 20–30% performing part of the population is chosen for mutation at every iteration, which drastically enhances this algorithm’s concentration on refining the most promising solutions while still allowing new areas to be explored in a controlled manner. Application of mutations to the best solutions means the algorithm effectively exploits high-quality areas within the search space. The number of elite individuals selected for mutation is computed as:

15 $$\:{M}_{\mathrm{mut\:}}\left(t\right)={M}_{\mathrm{m}\mathrm{i}\mathrm{n}}+\:\mathrm{r}\mathrm{o}\mathrm{u}\mathrm{n}\mathrm{d}\:\left(\left({M}_{\mathrm{m}\mathrm{a}\mathrm{x}}-{M}_{\mathrm{m}\mathrm{i}\mathrm{n}}\right)\times\:\frac{t}{T}\right)$$

where $\:{M}_{\mathrm{m}\mathrm{i}\mathrm{n}}$ is the minimum number of the best solutions for which mutation is applied, usually set to $\:20\mathrm{\%}$ of the population size $\:N$, and $\:{M}_{\mathrm{m}\mathrm{a}\mathrm{x}}$ is the maximum number of the best solutions for which mutation is applied, set at $\:30\mathrm{\%}$ of $\:N.t$ is the current iteration, and $\:T$ is the total number of iterations. Thus, the mutation process for each selected best-performing individual follows the equation:

16 $$\:{X}_{mut\left(i\right)}(t+1)={X}_{mut\left(i\right)}\left(t\right)\times\:(1+\alpha\:(t)\times\:G+\beta\:(t)\times\:C)$$

where $\:{X}_{mut\left(i\right)}\left(t\right)$ represents the position of the $\:i$th best individual selected for mutation at iteration $\:t$, $\:G$ is drawn from a standard normal distribution $\:\mathcal{N}\left(\mathrm{0,1}\right)$, $\:C$ is sampled from a standard Cauchy distribution with location parameter 0 and scale parameter 1. For numerical stability, extreme Cauchy values are truncated within a predefined bound. In practice, Gaussian samples are generated using standard normal generators (mean 0, variance 1), while Cauchy samples are generated using the inverse transform method $\:C=\mathrm{t}\mathrm{a}\mathrm{n}\left(\pi\:\right(u-0.5\left)\right)$, where $\:u\sim\:U\left(\mathrm{0,1}\right)$. Since the Cauchy distribution has undefined variance, its scale parameter is normalized to 1, and large outliers are clipped to prevent numerical instability, $\:\alpha\:\left(t\right)$ and $\:\beta\:\left(t\right)$ are time-varying scaling factors that adapt the size of mutation steps and are defined as:

17 $$\:\alpha\:\left(t\right)=c\times\:(\mathrm{e}\mathrm{x}\mathrm{p}(t/T)-c)$$

18 $$\:\beta\:\left(t\right)=c\times\:(\mathrm{e}\mathrm{x}\mathrm{p}(1-t/T)-c)$$

where c = 0.8. MDMS focuses on the top 20–30% of the population so that for further refinement, the best solutions are explored and exploited. Therefore, a focused approach improves convergence speed along with increasing the accuracy of final solutions. In other words, MDMS adaptively switches between Cauchy and Gaussian mutations during the run time of the optimization process. The Cauchy mutations with large step sizes offering wider exploration across the solution space are favored by the early generations. As the generation progresses, it shifts in favor of the Gaussian mutation to apply smaller steps in the interest of exploitation and fine-tuning the best solutions.

The MDMS focuses on 20–30% of the finer solutions, thereby diversifying explorations in the earlier stages but refining those finer solutions during the exploitation phase of iteration. Such a balance of exploration and exploitation gives the algorithm greater efficiency, especially for complex and multi-modal optimization problems. Within the human memory framework, MDMS can be interpreted as selective reinforcement of high-quality experiences. Rather than mutating the entire population indiscriminately, AEHMO refines only the most successful memories, analogous to how humans repeatedly revisit and slightly adjust effective past decisions. The combination of Cauchy and Gaussian mutations reflects coarse recall followed by fine-grained refinement, maintaining consistency with memory-based learning rather than introducing an independent search metaphor.

### Dynamic Drift Search Strategy (DDS)

The DDS strategy takes inspiration from the unpredictable and dynamic motion of rime-ice particles^48^. It was developed with the idea of overcoming the entrapment in a local optimum problem by incorporating wild, extensive exploration during the early iterations of the process. DDS provides AEHMO with the possibility of making an extensive and varied exploration of the solution space, especially during the initial iterations of the algorithm when the probability of discovering new, unvisited areas is crucial. Extremely erratic and diverse mobility is exhibited by rime-ice particles. The mathematical model of the DDS takes into account random directional translations, borrowing from inspirations such as the movement of particles or conditions within the environment. Its dynamic behavior is governed by the relationships among several parameters: $\:\theta\:$, $\:\gamma\:$, and $\:L$, depending on the so-far number of function evaluations $\:FEs$. This enables the adaptation to change the search behavior over time and to let it focus stronger and stronger. During the optimization process, DDS performs a broad exploration of the solution space early and later confines this search to promising areas. Therefore, DDS helps to keep the algorithm from becoming stuck in local optima by enabling quick exploration of the whole search space at the outset. Equations (19–22) exhibit the mathematical representation of the DDS strategy as follows:

19 $$\:{X}_{ij}^{new}={X}_{best,j}+{r}_{8}.cos\theta\:.\gamma\:.\left(h.\left({u}_{b}-{l}_{b}\right)+{l}_{b}\right),{r}_{9}<L$$

20 $$\:\theta\:=\pi\:.\frac{\mathrm{t}}{10T}$$

21 $$\:\gamma\:=1-\frac{\left[\frac{w.t}{T}\right]}{w}$$

22 $$\:L=\sqrt{\frac{t}{T}}$$

In the $\:j-th$ dimension, the position of the best individual is represented by $\:{X}_{best,j}$, while $\:{X}_{ij}^{new}$ indicates the new position following the DDS. The random integer $\:{r}_{8}$, which ranges from − 1 to 1, determines the direction in which the rime-ice particles move. A variable called $\:\gamma\:$, which varies depending on the number of evaluations, models environmental conditions. Given the great degree of unpredictability in the particles’ movements, the distance between two particles is represented by $\:h$, which is arbitrarily chosen between [0, 1]. As stated in the original algorithm, $\:w$, which regulates the number of segments in the step function, is set to 5. The current and maximum number of evaluations are denoted by $\:t$ and $\:T$, respectively. The random number $\:{r}_{9}$, which ranges from 0 to 1. The primary rationale for using DDS is that it reduces the risk of premature convergence. The rapid and erratic exploration in performance does not allow the algorithm to stick with the local optima. This strategy is very helpful for high-dimensional problems, where the possibility of premature convergence is higher. DDS provides a random contribution that conserves the diversity of solutions throughout the process. Therefore, AEHMO has enhanced global search capability for better convergence toward the global optimum in difficult cases.

Although DDS is mathematically inspired by drift-like motion, its role within AEHMO aligns with cognitive exploratory behavior rather than a physical metaphor. In human decision-making, periods of uncertainty often trigger exploratory deviation from familiar patterns, allowing individuals to search broadly before committing to refined choices. DDS captures this exploratory drift by enabling large, irregular movements early in the search process, while remaining subordinate to the memory recall structure that governs solution evaluation and retention.

### Chaotic reverse learning strategy (CRL)

One of the major drawbacks of the original HMO algorithm is related to its convergence towards the local optimum, especially when searching within complex multi-modal search space. The core issue is that the algorithm lacks an effective mechanism for exploring diverse regions of the search space and escaping local optima. The traditional update rules in HMO are not efficient in every case to explore the global search space, which delays convergence and hence reduces performance. In this regard, CRL strategy is developed which is capable of incorporating chaotic mapping with opposition-based learning to improve the exploration capability and enhance the escaping ability of the algorithm from the local optima.

Opposition-based learning (OBL) introduces new solution vectors by creating an opposite population of the current population. This helps the algorithm to find solutions that may not be easily reachable through normal update rules. OBL is useful when there are a large number of local optima spread in the solution space, and the algorithm needs to come out of the false local optima more effectively. The formula for OBL can be written as follows:

23 $$\:O{D}_{i}\left(t\right)={l}_{b}+{u}_{b}-{X}_{i}\left(t\right)$$

where $\:O{D}_{i}\left(t\right)$ is the position of the $\:i$^th^ opposite individual at iteration $\:t$. The traditional OBL approaches create an opposite solution only at fixed positions, reducing the flexibility of the algorithm; this may affect its dynamic ability to explore new areas. From this perspective, CRL uses chaotic mapping in its opposition process to introduce structured randomness, hence allowing more flexibility and greater exploration by the algorithm. On the other hand, some works have introduced randomness in the opposition process for:

24 $$\:O{D}_{i}\left(t\right)={l}_{b}+{u}_{b}-{R\times\:X}_{i}\left(t\right)$$

This random opposition-based learning method introduces a degree of variability, but the overall convergence rate can still be relatively slow. In this case, the CRL strategy utilizes chaotic mapping to oppose the learning strategy to enhance its exploration capability. The chaotic maps differ from random number arrays based on a probability distribution due to their dynamic properties. This results in a more rapid exploration of the solution space. The CRL formula is given as follows:

25 $$\:O{D}_{i}\left(t\right)={l}_{b}+{u}_{b}-{F}_{z}\times\:{X}_{i}\left(t\right)$$

26 $$\:{F}_{j+1}=\mathrm{s}\mathrm{i}\mathrm{n}\left(\pi\:{F}_{j}\right)$$

In this regard, $\:{F}_{0}$ is a vector of random values in the range of 0–1 and $\:{F}_{z}$ is a chaotic sequence after $\:z$ iterations of sine mapping. This chaotic sequence follows some structured randomness, enhancing the exploration capability of this algorithm. Indeed, the proposed chaotic sequence-based opposition brings periodicity to the sine map, allowing the algorithm to probe the solution space more effectively than purely randomly generated opposition. Having too many iterations would increase the running time beyond endurance, whereas having too few iterations could result in a loss of diversity. Therefore, the parameter $\:z$ is set to 20 as a balance to ensure that the algorithm has enough diversity to explore the solution space without significantly increasing the running time. Moreover, the proposed algorithm uses a greedy process for selection whereby the best $\:N$ individuals from both original and opposite populations remain in the set of solutions throughout an optimization process that keeps quality as high as possible. While maintaining diversity to avoid premature convergence, the chaotic mapping utilized in the CRL strategy considerably enhances the convergence speed and exploration capability of the basic algorithm. This dynamic approach lets the algorithm provide a more efficient search mechanism and, hence, improved overall performance compared to the standard opposition-based learning methods.

From a memory-theoretic standpoint, CRL represents nonlinear and associative recall behavior, where memories are revisited in a structured yet non-repetitive manner. The chaotic mapping introduces bounded irregularity, mimicking how human recall is neither strictly deterministic nor purely random. By embedding chaotic opposition within the memory-driven framework, AEHMO enhances diversity while maintaining conceptual consistency with cognitive recall processes.

### Synergistic rationale of the proposed enhancement strategies

Although AEHMO combines four strategies for enhancement, the combination of the strategies is not arbitrary, but guided by a complementary and phase-oriented design principle. Each strategy addresses one of the different limitations of the original HMO and works at different time and structure levels of the search process to guarantee a cooperative, rather than redundant, behavior.

The adaptive parameters $\:{w}_{1}$ and $\:{w}_{2}$ constitute a global control layer that is used to control the exploration-exploitation balance during the optimization process. By replacing the fixed variables $\:b$ and $\:\delta\:$ with the variable ones, this layer produces a smooth and non-linear transition from general exploration to specific exploitation to form a stable backbone for the rest of the strategies. Building upon this adaptive control, in the DDS strategy the main action is taken during the early search phase, by adding large-scale directional drift to cover more spatial area and quickly explore unexplored areas of the search space. DDS complements the $\:{w}_{1}$ by reinforcing the early exploration without interfering with the convergence because its effect naturally fades away with the progress of iteration. The CRL strategy works on the population diversity level and provide a structured mechanism of escaping of local optima, through the chaos opposition. Unlike purely random perturbations, CRL presents deterministic but not repeating transformations, and therefore AEHMO can return to unexplored regions despite the reduced diversity of the population. This makes CRL particularly effective in situations when the influence DDS starts weakening, and the search starts to concentrate. Finally, MDMS is also intentionally limited to a small elite subset of solutions, making up a local refinement layer. By combining both Cauchy and Gaussian mutations, MDMS fills the gap between global jumps and fine-grained tuning, ensuring that high quality regions identified using DDS and CRL are efficiently exploited, rather than prematurely being fixed in position.

Collectively, these strategies form a hierarchically cooperative mechanism of non-conflicting strategies: adaptive parameters regulate the general dynamics of search, DDS and CRL ensure sufficient global coverage and diversity and MDMS stimulates intensification of exploitation in promising regions. Moreover, the selection of these four strategies was based on a limitation-driven design analysis rather arbitrary stacking of operators. Each enhancement represents a different structural deficiency that was observed in the original HMO and preliminary experiments using partial combinations of enhancements showed that the individual enhancements improved specific aspects of performance but did not consistently ensure stable convergence across both benchmark and WSN scenarios. The complete four-strategy configuration was found to have the best trade-off between exploration depth, preservation of diversity and late-stage refinement, which was confirmed by the ablation analysis in the section “Ablation experiment”.

### Computational complexity

**Fig. 2:**
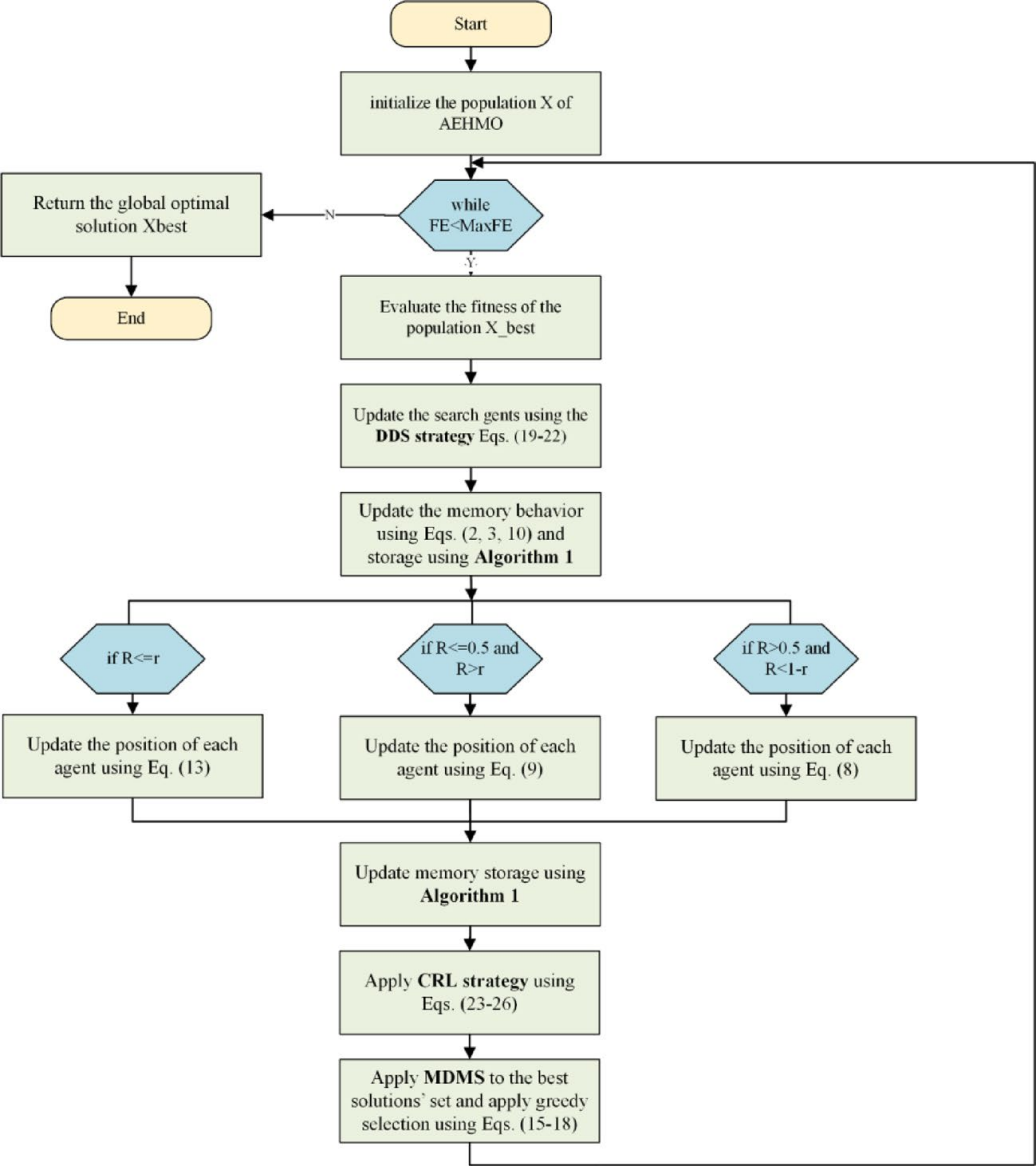
The steps of the proposed AEHMO algorithm.

The performance of an optimization algorithm defines its applicability across various problem domains, with computational complexity being the most important factor. The pseudocode and flowchart of the proposed AEHMO are depicted in Algorithm 2 and Fig. 2. According to Algorithm 2, in the case of the AEHMO algorithm, it can be analyzed concerning its building phases. Let $\:N$ denotes the number of solutions within the population, $\:T$ denotes the number of maximum function evaluations, and $\:D$ denotes the dimensionality of the problem.

- **This initialization step** is done once before the iteration starts. During that time, the population of solutions was created: initializing $\:N$ solutions, each having $\:D$ dimensions. So, the complexity in this phase will be $\:O(N\times\:D)$, because it corresponds to generating a diverse initial population.
- **The memory behavior update phase** then runs for every iteration once the population has been initialized. Since an update in every individual in the population is to be dependent on its memory, this sets up a time complexity of $\:O(T\times\:N)$, where $\:T$ is the iterations. Also, the phase of recollective behavior, which recalls and updates solutions according to previous successes and failures, has the same complexity bound of $\:O(T\times\:N)$ since its activity covers the population across iterations.
- **Chaotic Reverse-based Learning (CRL)**: CRL involves a chaotic mapping process. As specified in the section “Chaotic reverse learning strategy (CRL)”, the parameter $\:z$ (number of chaotic iterations) is set to $\:20$. For each individual $\:N$ in each iteration $\:T$, the algorithm performs $\:z$ iterations of the sine map. Thus, the CRL overhead is $\:O(T\times\:N\times\:D\times\:z)$. Since $\:z$ is a fixed constant ($\:z=20$), this remains $\:O(T\times\:N\times\:D)$ but increases the constant factor of the runtime.
- **Multi-Directional Mutation Strategy (MDMS)**: Unlike standard operators, MDMS is applied selectively to the elite portion of the population (the top $\:20\mathrm{\%}-30\mathrm{\%}$). If $\:k$ is the fraction of the population ($\:k\approx\:0.25$), the overhead for Gaussian and Cauchy mutations is $\:O(k\times\:T\times\:N\times\:D)$. This is computationally cheaper than applying mutation to the entire population.
- **Dynamic Drift Search (DDS)**: DDS introduces a secondary position update phase to prevent local optima stagnation. It calculates a drift vector and updates the position for all $\:N$ individuals across $\:D$ dimensions. This introduces one additional vector-based position update per iteration, represented as $\:O(T\times\:N\times\:D)$.

The total computational complexity of AEHMO is the summation of these parts:

$$Total complexity\:=\boldsymbol{O}(\boldsymbol{N}\cdot\:\boldsymbol{D})+\boldsymbol{O}(\boldsymbol{T}\cdot\:\boldsymbol{N}\cdot\:\boldsymbol{D}{)}_{\mathrm{HMO\:}}+\boldsymbol{O}(\boldsymbol{T}\cdot\:\boldsymbol{N}\cdot\:\boldsymbol{D}\cdot\:\boldsymbol{z}{)}_{\boldsymbol{C}\boldsymbol{R}\boldsymbol{L}}+\boldsymbol{O}{\left(\boldsymbol{k}\cdot\:\boldsymbol{T}\cdot\:\mathbf{N}\cdot\:\mathbf{D}\right)}_{\mathbf{M}\mathbf{D}\mathbf{M}\mathbf{S}}+\boldsymbol{O}{\left(\boldsymbol{T}\cdot\:\mathbf{N}\cdot\:\mathbf{D}\right)}_{\mathbf{D}\mathbf{D}\mathbf{S}}.$$

Factoring out the common terms:

$$Total complexity\:=\boldsymbol{O}(\boldsymbol{T}\cdot\:\boldsymbol{N}\cdot\:\boldsymbol{D}\cdot\:[1+\boldsymbol{z}+\boldsymbol{k}+1\left]\right)$$

Although the additional strategies (CRL, MDMS, and DDS) introduce more operations per iteration-specifically the 20 iterations within CRL-they are all linear functions of $\:T,N$, and $\:D$. Therefore, the overall complexity class of AEHMO remains $\:O(T\times\:N\times\:D)$. This is asymptotically equivalent to the original HMO, confirming that the significant performance gains in energy efficiency and convergence are achieved without moving to a higher order of computational complexity.

**Algorithm 2:**
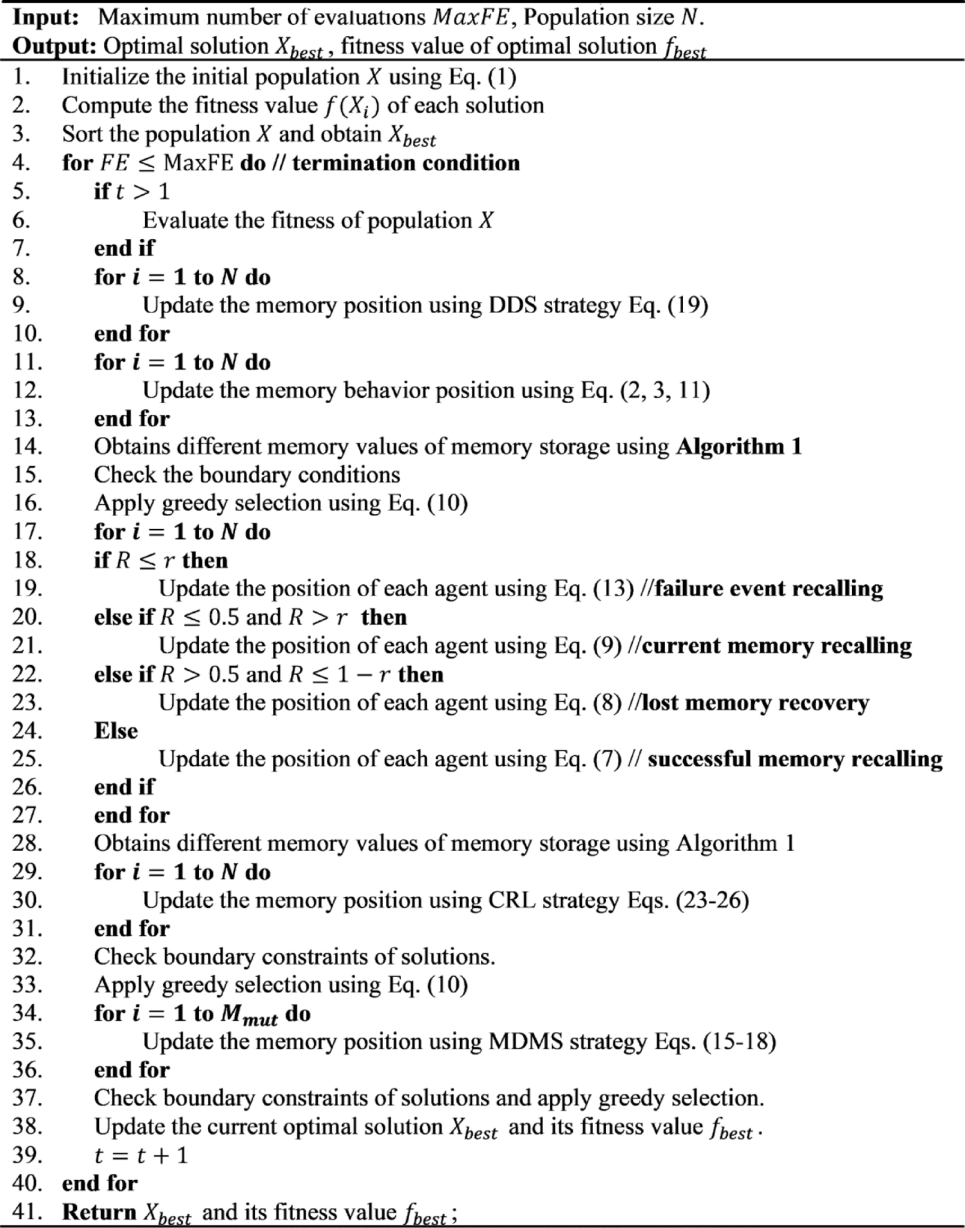
AEHMO algorithm.

## AEHMO for global optimization

This section presents a detailed evaluation of AEHMO, covering benchmark functions, experimental setup, and performance comparisons against competing algorithms. Given its representative diversity of optimization challenges, the CEC2017 benchmark suite is selected for evaluation. The goal is to investigate how effectively AEHMO solves complex optimization problems and traverses diverse fitness landscapes. The CEC2017 suite is categorized into four classes: F1 and F3 are unimodal, F4–F10 are multimodal, F11–F20 are hybrid, and F21–F30 are composite functions. These function classes collectively test the algorithm’s adaptability across scenarios that demand both broad global exploration and precise local refinement. The experiments are conducted at 10D, 30D, 50D and 100D problems for CEC2017 so that the proposed algorithm is challenged over diverse problem sizes.

### Environmental settings and rival algorithms

This section describes the experimental setup used in all conducted experiments. Experiments were conducted on a system with a CPU of i7-10750 H, Windows 10 (64-bit), running at 2.60 GHz, and a capacity of 32 GB of RAM. The Matlab2020b was used as the software environment for the implementation of the algorithm. All algorithms ran with a population size of 30. The maximum number of evaluations to be applied was set to 30,000. Each algorithm was independently run 30 times on each benchmark function to ensure statistical reliability. To ensure reproducibility, all experiments were initialized using a fixed random seed (rng(42) in MATLAB), applied uniformly across all 30 independent runs and all compared algorithms. This guarantees that initial population diversity is consistent, and results are fully reproducible.

The compared algorithms include Grey Wolf Optimization Algorithm (GWO)^49^, Arithmetic Optimization Algorithm (AOA)^50^, IVY Algorithm^51^, Hiking Optimization Algorithm (HOA)^52^, Human Evolutionary Optimization Algorithm (HEOA)^53^, Memory based Hybrid CSA (MHCSA)^54^, the hybrid AOA-HHO^55^, Modified Artificial Bee Colony and Particle Swarm Optimization (MHABC-PSO)^56^, adaptive enhanced human memory algorithm (ASG-HMO)^57^, Modified Group Teaching Optimization Algorithm (MGTOA)^58^ and the original HMO. The parameter settings of each algorithm are listed in Table 2 to ensure an unbiased and comprehensive comparison.

**Table 2:** Various parameter settings for the comparative algorithms.

Algorithm	Parameter set
HMO, AEHMO	$\:r=0.1,\:\delta\:=1$, Elite fraction=0.2, chaotic depth ($\:z$)=20, mutation mix ratio=30% for CEC2017, 25% for WSN, $\:{w}_{1},\:{w}_{2}=[0,\:1]$, scaling factor ($\:c$)=0.8 for CEC2017, 0.7 for WSN
AOA	$\:MOP=0.2,\:1,\:\alpha\:=5,\:\mu\:=0.499$
GWO	a: Linear reduction from 2 to 0
IVY	1 =[1, 1.5), =[0,1]
HOA	Angle of inclination of the trail =[0, 50o] Sweep factor (SF)=[1, 3]
HEOA	Leaders = top 40%, Explorers = next 30%, Followers = next 20%, Losers = bottom 10%;
MHCSA	$\:\alpha\:=\left[0.98,\:2E-7\right],\:{w}_{l}=0.1,\:{w}_{u}=0.9$
AOA-HHO	MOA increases from 0.2 to 1, µ = 0.5
MHABC-PSO	$\:{c}_{1}={c}_{2}=2,\:w=\left[0.9,\:0.4\right]$
MGTOA	Limit=$\:lg\left(t\right)$
ASG-HMO	$\:r=0.1,\:\delta\:=1\:$

All compared algorithms were configured using the default parameter settings reported in their respective original publications, ensuring that each algorithm operates under its recommended and validated configuration. This approach avoids any unintentional bias and is consistent with standard benchmarking practice in the metaheuristic optimization literature.

### Performance indicators

In the experiments designed for global optimization, the performance of the algorithms is measured with the following set of indicators: mean (AVG), standard deviation (SD), Friedman rank (FR), and the Wilcoxon rank test. These four metrics provide a comprehensive evaluation of each algorithm’s behavior, described as follows:

- **Mean (AVG)** represented in Eq. (27) is one of the most important measures of central tendency, reflecting the average solution quality achieved by an algorithm across multiple runs:

 

27 $$Mean\:=\frac{va{l}_{1}\left(x\right)+va{l}_{2}\left(x\right)+\cdots\:+{\mathrm{v}\mathrm{a}\mathrm{l}}_{M}\left(x\right)}{M}$$

- **The standard deviation (SD)**, calculated by Eq. (28), provides information about the dispersion or variability of the solutions supplied by the algorithm. A smaller SD reflects those solutions obtained are more similar which is defined as follows:

28 $$\:\mathrm{S}\mathrm{D}=\sqrt{\frac{{\sum\:}_{i=1}^{M}\:{\left({v}_{i}\left(x\right)-\mathrm{M}\mathrm{e}\mathrm{a}\mathrm{n}\right)}^{2}}{M-1}}$$

- **Friedman rank**^59^ ranks algorithms based on their overall performance across a set of benchmark problems, where a lower FR indicates superior performance.
- To compare the performance of any two algorithms statistically, the Wilcoxon rank sum test is performed^60^. A p-value below 0.05 indicates that the performance difference between algorithms is statistically significant and unlikely to have occurred by chance. Results show ‘R+’ to denote the number of functions where AEHMO significantly outperforms the competing algorithm, ‘R=’ to denote the number of functions where both algorithms show statistically similar performance, and ‘R−’ to denote the number of functions where AEHMO is significantly outperformed by the competing algorithm. Therefore, R− = 0 for a given competitor indicates that AEHMO never underperforms relative to that algorithm across all tested functions.

### Sensitivity analysis of the parameters

A sensitivity analysis was conducted to empirically justify the selection of the three key AEHMO parameters: the chaotic iteration depth ($\:z$) in CRL, the MDMS mutation percentage, and the MDMS scaling factor ($\:c$). The analysis consistently confirmed $\:z$ = 20, a mutation percentage of 20%–30%, and $\:c$ = 0.8 as the optimal values, balancing exploration strength, exploitation precision, and computational efficiency. Full results and discussion are provided in Supplementary Appendix A, where Table A1 presents the effect of varying the chaotic iteration depth ($\:z$), Table A2 reports the impact of different MDMS mutation percentage values, and Table A3 summarizes the performance variation across tested values of the scaling factor (c).

### Ablation experiment

In this section, we conduct an ablation experiment to evaluate the contribution of each single strategy introduced in order to enhance the performance of the HMO algorithm. The goal is to demonstrate, step by step, how each strategy individually enhances algorithmic performance and how their combination yields the best result in the full AEHMO configuration. In the ablation study, we consider the performance of various HMO variants on 29 benchmark functions of the CEC2017 test suite. Table 3 identifies whether each strategy is on or off in the various HMO variants. In other words, 1 means that the strategy is on while 0 means that the strategy is off.

**Table 3:** Different variants of HMO.

Variant	Adaptive Parameters (AHMO)	MDMS (MHMO)	DDS (DHMO)	CRL (CHMO)
HMO	0	0	0	0
AHMO	1	0	0	0
MHMO	0	1	0	0
DHMO	0	0	1	0
CHMO	0	0	0	1
AEHMO	1	1	1	1

The STD and AVG values obtained from this experiment are reported in Table B1. While the performance metrics for each individual strategy surpassed the HMO algorithm at most functions, the AHMO variant, for instance, with adaptive parameters, reduces it to an average rank of 4.52, indicating a key enhancement regarding how the balance between exploration and exploitation is performed. This allows the algorithm to smoothly transition between the global exploration phase and the local refinement phase and gives better convergence behavior compared to the original HMO, with an average rank of 5.07. This confirms that adaptability is important to allow for optimal performance over complex problem landscapes. Additionally, the DHMO variant implemented with the DDS strategy provided an even more impressive improvement; its average rank is 2.10. This strategy broadens exploration in the early stages and helps the algorithm avoid being trapped in local optima, hence making the overall search more efficient. Thus, DHMO is particularly powerful, demonstrating that the introduction of structured randomness can significantly enhance optimization results. Moderate improvements were achieved by the variants MHMO and CHMO, which had average ranks of 3.79 and 4.24, respectively.

**Fig. 3:**
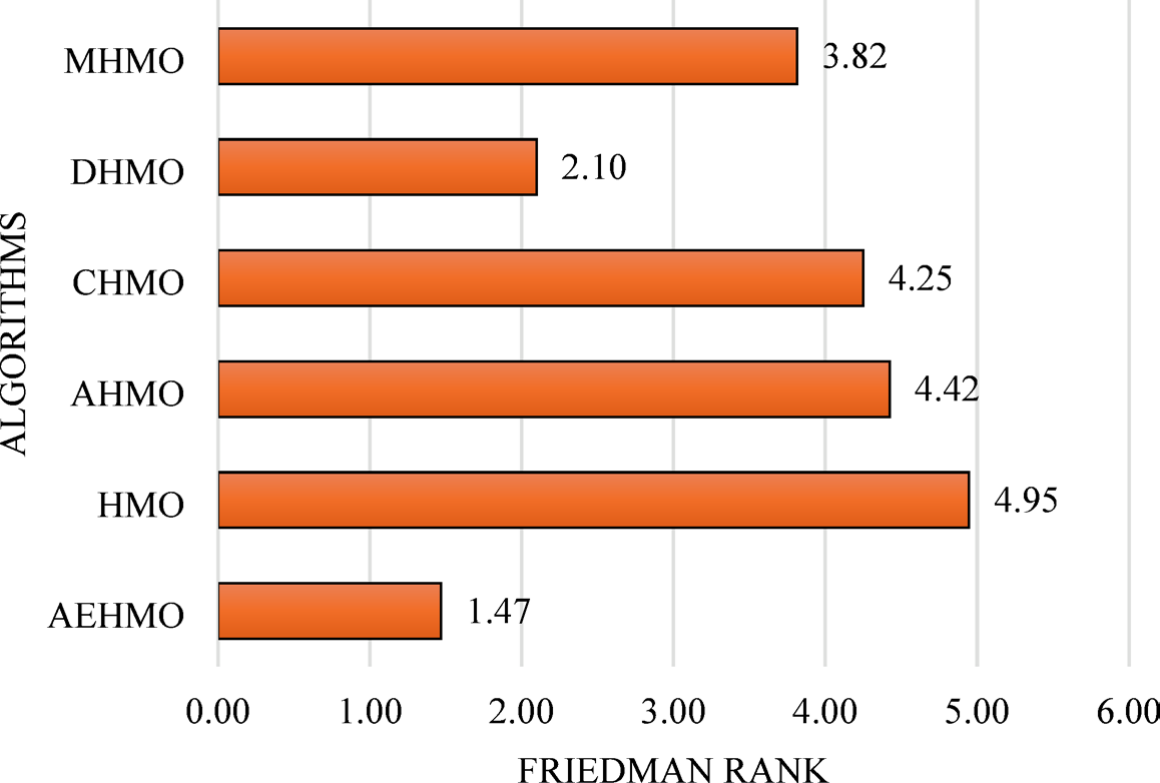
Friedman rank analysis of different variants of HMO.

On the other hand, Fig. 3 shows the FR results indicating the ranked performance of AEHMO to be superior among all variants with FR of 1.47, which is closely followed by the performance of DHMO with FR set to 2.10. This low performance of HMO over several functions points out that for enhancement in solving difficult optimization problems, the inclusion of diverse search strategies is essential. The results of the FR suggest that each strategy independently helps in enhancing the performance of the HMO algorithm. The combined effect of this, however, is stronger in the case of AEHMO to bring about an efficient and effective solution search mechanism. It confirms that all these strategies, while individually useful, when combined become much more powerful.

### Result analysis for global optimization

This section evaluates the performance of AEHMO for global optimization using various dimensions of CEC2017. In this experiment, 10D, 30D, 50D and 100D are tested.

#### Result analysis using CEC2017, 10D

The performance of AEHMO on unimodal functions demonstrates strong optimization capability in 10-dimension search space as presented in Table C1. For the unimodal functions F1 and F3 AEHMO has achieved the first rank in both cases with fitness values of 1.00E + 02 and 3.00E + 02. In F1, AEHMO performed much better than all the competitors namely HMO (4.68E + 02), MGTOA (2.42E + 03), AOA (8.32E + 07), IVY (1.50E + 08), and HEOA (8.06E + 08) with an extremely low standard deviation of 2.15E-06 indicating near-perfect convergence precision. The F3 result with a standard deviation of 0.00E + 00 has demonstrated complete stability across all independent runs and has shown the effectiveness of AEHMO in exploiting smooth unimodal landscapes. These results validate that AEHMO’s adaptive parameters were successful in achieving intensive local exploitation after adequate global exploration in unimodal terrains. In the multimodal functions (F4-F10), the dominant performance was maintained by AEHMO, ranking first in four out of seven functions: F4 (4.00E + 02), F6 (6.00E + 02), F8 (8.14E + 02), F9 (9.01E + 02). The algorithm achieved the second rank in F10 after ASG-HMO (1.42E + 03), third in F7 (7.34E + 02) behind GWO (7.24E + 02) and ASG-HMO (7.30E + 02), and the fifth ranking in F5 (5.24E + 02) where GWO had the best performance (5.16E + 02). The consistently low standard deviations for F4 (4.93E-03), F6 (4.12E-01), F8 (4.92E + 00) and F9 (1.17E + 00) suggest good search behavior and consistency of solution quality over multiple runs. The superior performance in multimodal functions can be explained by the DDS, which permits extensive exploration by chaotic drift movements that prevent premature convergence to local optima in landscapes with multiple local optima. The relatively larger variance of F5 (8.36E + 00) and the lower ranking suggest that the functions with regular periodic structures can overwhelm the exploration mechanism, which may lead to occasional entrapment in the suboptimal regions.

For hybrid functions (F11-F20), AEHMO demonstrated excellent performance in this difficult category as shown in Table C1. The algorithm ranked first in nine of the 10 hybrid functions: F12 (1.41E + 03), F13 (1.31E + 03), F14 (1.42E + 03), F15 (1.50E + 03), F16 (1.62E + 03), F17 (1.72E + 03), F18 (1.81E + 03), F19 (1.90E + 03) and F20 (2.01E + 03). For example, in F12 AEHMO obtained 1.41E + 03 as compared to HMO 3.36E + 05, HEOA 4.85E + 06 and HOA 2.01E + 07 giving improvement of more than 99%. Similarly, in F18, AEHMO had a value of 1.81E + 03 and HOA had a value of 1.71E + 08. The CRL plays a critical role in this performance by introducing chaotic sine mapping that generates structured randomness allowing AEHMO to escape the local optima effectively while preserving the directed search to promising regions in complex hybrid landscapes combining shifted, rotated and expanded functions. The performance is further enhanced by the MDMS by using Gaussian and Cauchy mutations on the best 20–30% of solutions to achieve a balance between fine tuning and diversity preservation. The very low standard deviations in F18 (1.06E + 00) and F19 (4.30E-01) indicate an excellent consistency of convergence, while the second rank position of F11 with small difference from the best result (1.11E + 03 vs. 1.10E + 03) supports the good performance of AEHMO in the whole hybrid function suite.

The composite functions (F21–F30) are the most difficult optimization scenarios due to their complicated structures with various combinations of multiple basic functions with different shift and rotation transformations. AEHMO exhibited strong overall performance in this category, as shown in Table C1. AEHMO achieved the first rank in 4 of 10 composite functions, F24 (2.66E + 03), F28 (3.21E + 03), F29 (3.16E + 03), F30 (3.43E + 03) and third rank in F21, F22 (2.30E + 03), fifth in F25 (2.93E + 03) and fourth in F26 (3.09E + 03). In F30, AEHMO obtained 3.43E + 03 as compared to HMO’s 9.88E + 05, HEOA’s 1.55E + 06 and HOA’s 2.29E + 06, which is of considerable performance advantages. The low value of the standard deviation in F24 (1.24E + 02) and F30 (2.41E + 01) indicates stable convergence behavior in independent runs. However, performance of the algorithm was more varied between composite functions than unimodal, multimodal and hybrid functions with several functions having third or lower ranks. The synergistic combination of adaptive parameters, MDMS, DDS, and CRL allows successful balance between exploration and exploitation, allowing AEHMO to move through the complex compositional landscapes in which multiple basic functions generate overlapping basins of attraction.

**Fig. 4:**
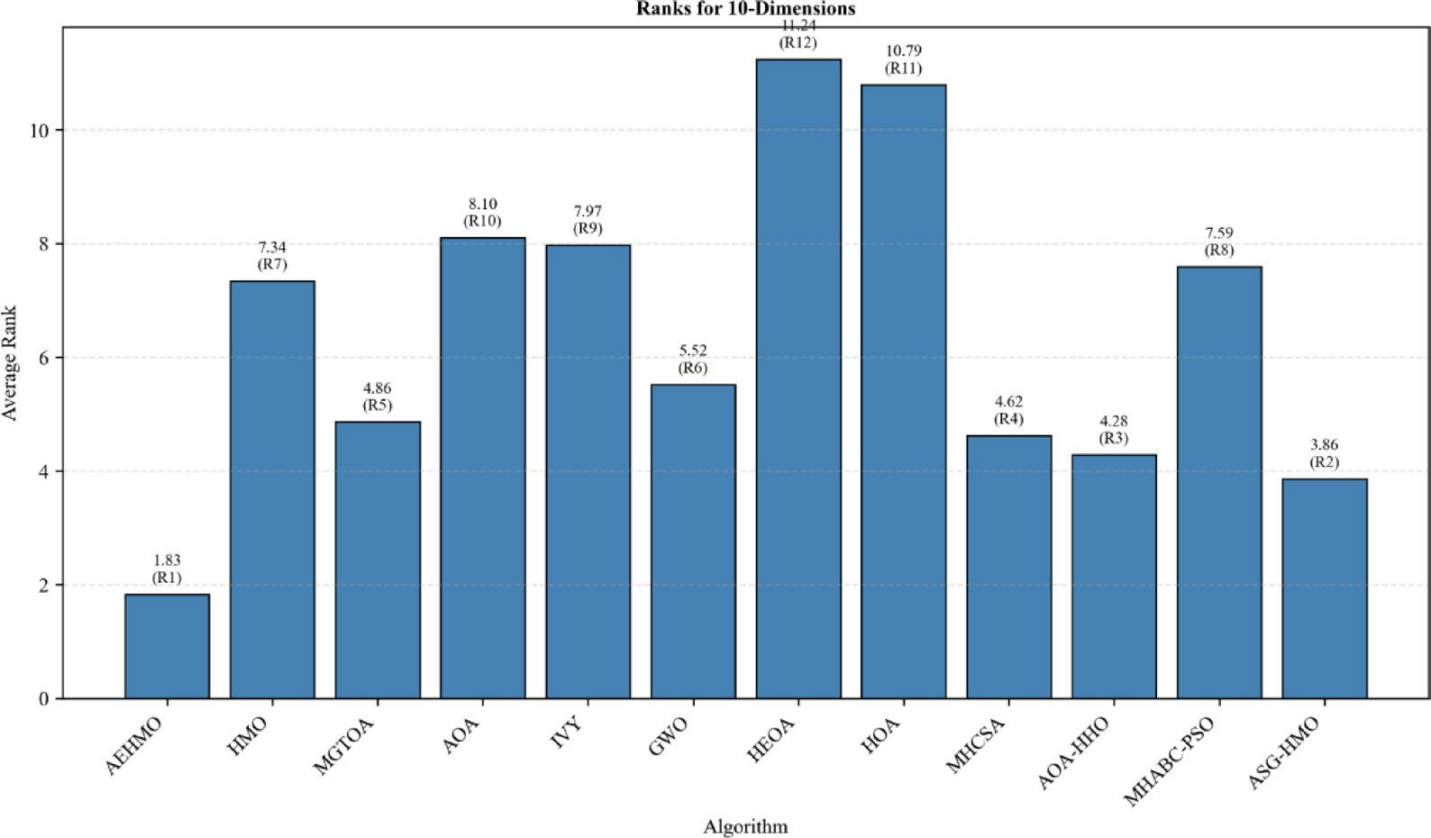
Average ranks of various algorithms using CEC2017, 10D.

The better than average overall performance of AEHMO with an average rank of 1.83 for all 29 CEC2017 functions in 10 dimensions shown in Fig. 4 shows AEHMO to be an effective global optimization algorithm for low dimensional problems. However, AEHMO did exhibit some significant deterioration in performance in some specific cases in composite functions which require careful analysis. In F21, AEHMO achieved the third rank with a fitness value of 2.26E + 03 (SD: 6.36E + 01), behind AOA-HHO (2.22E + 03) and MHCSA, while outperforming the remaining algorithms including ASG-HMO (2.30E + 03). This relatively high standard deviation indicates a lack of consistent convergent behavior and some convergent behavior into secondary optima and indicates that the composition function combining rotated hybrid functions is generating landscapes where the chaotic mapping parameter may need to be changed. The complex structure of the function with multiple rotation matrices and overlapping basins seems to interfere with the mechanism of adaptive balance and makes some runs move to the exploitation phases before having a sufficient global exploration. In F22, AEHMO achieved the third rank (2.30E + 03) with very low standard deviation (1.15E + 00), but MGTOA (2.25E + 03) obtained better fitness, suggesting that certain hybrid composition structures favor alternative exploration strategies. The F23 third-rank outcome (2.62E + 03) behind GWO (2.61E + 03) and AOA-HHO (2.62E + 03) seems to indicate that the composition functions benefit from the hierarchical search mechanisms instead of memory-based recall. The F25 fifth rank performance (2.93E + 03) is the most significant case of failure in composite functions, where ASG-HMO (2.92E + 03), AOA-HHO (2.92E + 03), MHCSA (2.92E + 03) and MHABC-PSO (2.93E + 03) were better than AEHMO. This composite functions with certain shift vectors result in regularly spaced local optima which can cause the DDS to spend too many iterations in non-promising areas. Similarly, for F26, AEHMO was ranked fourth (3.09E + 03) with a relatively high standard deviation (1.07E + 02) compared to those of top performers MHCSA (2.94E + 03) and AOA-HHO (2.94E + 03), indicating sensitivity to initial population distribution in asymmetric composite landscapes. The F27 fourth-rank position (3.10E + 03) indicates issues with composition functions that combine expanded functions, in which the search bias that memory-based updates generate might prevent even exploring asymmetrically distributed basins. These failure patterns indicate that, although AEHMO performs well on most optimization problems, certain composite functions remain challenging. Highly regular periodic structures, complex rotation matrices, and asymmetric basin distributions can disrupt the exploration–exploitation balance, resulting either in too much exploration in the converged regions or premature exploitation without enough global search completion in all the compositional components.

#### Result analysis using CEC2017, 30D

As dimensionality increased to 30, AEHMO was able to maintain an exceptional performance in unimodal functions as well as increased adaptability in multimodal landscapes as shown in Table C2. For the unimodal functions F1 and F3, AEHMO obtained first and third ranks respectively with fitness values of 2.35E + 03 and 8.52E + 03. In F1, AEHMO got first rank with a standard deviation of 1.78E + 03 that was far better than all other competitors including MHCSA with a standard deviation of 2.05E + 03, AOA-HHO with a standard deviation of 6.44E + 03, HMO with a standard deviation of 1.26E + 05, MGTOA with a standard deviation of 1.24E + 09. While the absolute fitness value did increase substantially from the 10-dimension result (1.00E + 02), AEHMO was able to retain its superior convergence capability and further maintain the largest performance gap among competitors, demonstrating an excellent scalability to higher-dimensional unimodal spaces. However, in F3, AEHMO was for the first time placed in the third rank (8.52E + 03) after MHCSA (3.02E + 03) and AOA-HHO (3.96E + 03) which represents a deviation from its first-rank position in 10 dimensions. The resulting increase of the standard deviation 1.84E + 03 shows the natural variability that comes with the increased 30-dimensional search space, which increases the difficulty of the search landscape as the dimensionality increases. For multimodal functions (F4-F10), AEHMO showed significantly better performance than 10 dimensions and ranked first in four out of seven functions: F6 (6.12E + 02), F7 (8.59E + 02), F9 (1.86E + 03) and F10 (3.78E + 03) with F5 in third position (6.56E + 02) behind ASG-HMO (5.70E + 02). The uniformly low standard deviations among F6 (5.74E + 00), F7 (3.36E + 01), F8 (1.97E + 01) and F9 (8.33E + 02) indicate that the DDS and CRL strategies become more efficient in higher dimensions by simultaneously preventing premature convergence and retaining solution diversity in complicated multimodal landscapes containing exponentially growing numbers of local optima.

The results of 30 dimensions hybrid and composite function show AEHMO’s outstanding scalability to complex high-dimensional optimization landscapes as shown in Table C2. For hybrid functions (F11-F20), AEHMO achieved the first rank in all of ten functions except for F16. Particularly good are the results for F12 (5.30E + 04), F13 (9.61E + 03), F14 (1.51E + 03), and F18 (2.63E + 03) where AEHMO gave orders of magnitude improvements over the competitors. In F12, AEHMO has obtained 5.30E + 04 whereas HMO has obtained 3.50E + 07, HEOA 1.97E + 09 and HOA 9.63E + 09, which is better than all algorithms. Similarly, in F13, AEHMO gave 9.61E + 03 while HEOA gave 1.11E + 09 and HOA gave 4.21E + 09. The very low standard deviations in F14 (1.43E + 01), F19 (1.41E + 01) and F18 (3.73E + 02) indicating stable and consistent convergence across all independent runs, which is a good sign of the stability of the algorithm in the case of complex hybrid landscapes. For composite functions (F21-F30), AEHMO got first rank in eight out of ten functions (F22 (2.30E + 03), F23 (2.73E + 03), F25 (2.89E + 03), F26 (3.91E + 03), F27 (3.20E + 03), F28 (3.18E + 03), F29 (3.80E + 03), F30 (7.36E + 03)), second rank in F24 (2.92E + 03). In F30, the performance of AEHMO was 7.36E + 03 compared with 5.17E + 06 for HMO, 2.37E + 08 for HEOA, and 8.89E + 08 for HOA which are significant performance improvement. The small standard deviation in F27 (2.33E-04) is a sign of near-perfect consistency in the convergence, while the small variance in F25 (7.07E + 00) and F28 (3.84E + 01) is a sign of the stability of AEHMO in wide range of composite structures. The enhanced efficiency of MDMS in higher dimensions by the selective mutation of elite solutions using adaptive Gaussian and Cauchy distributions makes possible the more refined balancing of exploration and exploitation as the search progresses through more extensive compositional solution spaces.

**Fig. 5:**
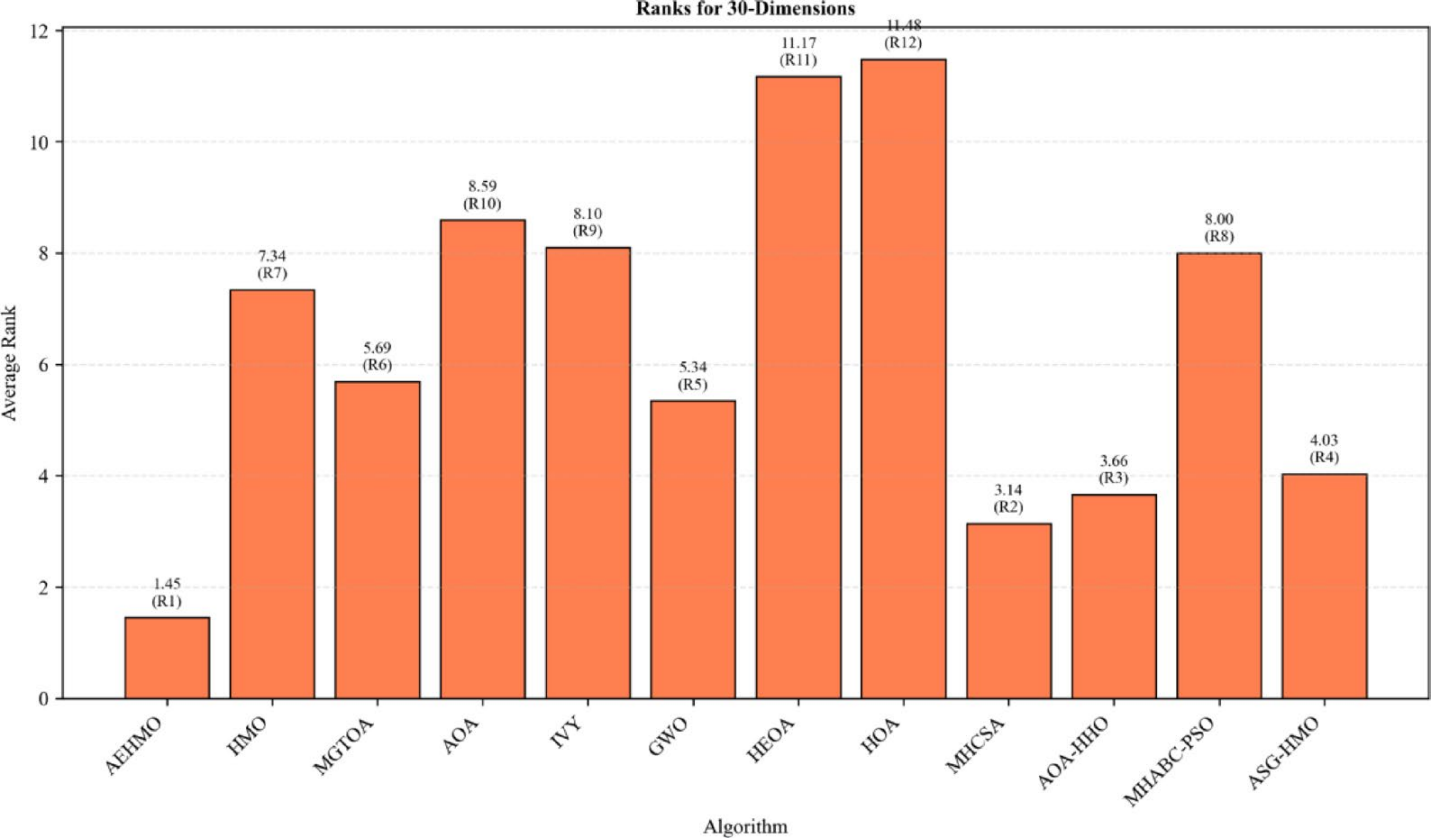
Average ranks of various algorithms using CEC2017, 30D.

Despite the fact that the overall better performance when averaged over 30 dimensions, which has an average rank of 1.45 as shown in Fig. 5, AEHMO experienced certain patterns of failures which are different from the 10-dimension situation and deserve in-depth analysis. The largest change is in unimodal function F3 where AEHMO reached rank 3rd (8.52E + 03) from its 1 st rank in 10 dimensions, while MHCSA (3.02E + 03) and AOA-HHO (3.96E + 03) have better performance. The large standard deviation value of 1.84E + 03 indicates that the convergence behavior is not consistent from one run to the other, which implies that the structure of 30-dimensional of the function is such that the adaptive parameters may enter the exploitation phases prematurely without having attained sufficient global exploration. The shifted and rotated nature of F3, when expanded to 30 dimensions, appears to result in misleading information from gradients, which result in the memory-based recall mechanism paying attention to suboptimal regions. In case of multimodal functions, F4 and F5 are the most remarkable cases where AEHMO is ranked at the third position in both cases with fitness values of 5.05E + 02 and 6.56E + 02, respectively. For F4, MHCSA (4.97E + 02) and AOA-HHO (4.91E + 02) showed better results than AEHMO and in F5, ASG-HMO (5.70E + 02) and GWO (6.24E + 02) gave better result. The expanded structures of these functions with 30 dimensions present exponentially more local optima which can overwhelm the exploration mechanism and the algorithm may spend too many iterations in non-promising areas before finding really optimal basins. The composite function F21 is the most important failure case where AEHMO showed the poorest rank (5th with 2.43E + 03, SD: 5.99E + 01) which is significantly poorer than the best performers ASG-HMO (2.40E + 03, SD: 2.44E + 01) and GWO (2.40E + 03, SD: 1.69E + 01). This performance is a significant deterioration from the third rank in 10 dimensions and the composition function of rotated hybrid components will be a more problematic composition function as the dimensionality increases. The high variance shows that the intensity of the chaotic mapping requires dimensional scaling, as the set of parameters is less effective when the solution space increases from 10 to 30 dimensions and the optimal combination of hybrid component combinations is not always found. Additionally, F16 and F24 got 2nd rank (2.42E + 03 and 2.92E + 03) behind GWO (2.31E + 03) and ASG-HMO (2.92E + 03) respectively, with F24 having a higher standard deviation (2.90E + 01) than ASG-HMO’s 2.38E + 01, which shows consistency problems of the expanded function. The result of F16 shows that GWO’s hierarchical hunting strategy provides advantages in specific hybrid compositions that include rotated expanded functions with dimension increase. These dimensional-dependent patterns of failures suggest that although the strategies of AEHMO are generally dimension-scalable, some function characteristics, particularly the shifted structure in F3, the regular distribution of multiple optima in the expanded functions (F4, F5), and the complex rotation matrices in composite function F21, necessitate dimension-adaptive parameter tuning mechanisms, which adjust the chaotic mapping iterations, the mutation step sizes, and the drift intensity on the basis of the problem dimensionality to ensure the optimal exploration-exploitation balance in the search process in 30-dimensional spaces.

#### Result analysis using CEC2017, 50D

In the 50-dimensional case, AEHMO turned out to be still scalable with dimensional dependent performance variations as shown in Table C3. For unimodal function F1 and F3, AEHMO obtained the third and the first rank with fitness value of 2.80E + 06 and 3.23E + 04, respectively. The F1 third rank behind AOA-HHO (7.80E + 03) and MHCSA (8.21E + 03) represents a decline from its first-rank performance in 10D and 30D suggesting that competitor algorithms become effective in high-dimensional unimodal spaces. But AEHMO first rank achievement in F3 (3.23E + 04) with a standard deviation of 7.42E + 03 provides evidence of robust handling of the function structure. For multimodal functions (F4-F10), AEHMO obtained first rank in six out of seven functions: F4 (5.28E + 02), F5 (7.01E + 02), F6 (6.19E + 02), F7 (1.04E + 03), F9 (4.99E + 03) and F10 (5.94E + 03) with improved performance from 30D where F4 and F5 both had 3rd rank. The uniform low standard deviations in F4 (6.29E + 01), F5 (2.92E + 01), and F6 (4.15E + 00) represent the enhanced convergence stability and the AEHMO’s adaptive parameters and DDS become more efficient in exploring expanded landscapes in 50 dimensions.

For hybrid functions (F11-F20), AEHMO was ranked first six times out of ten, maintaining good performance that was seen in 30D as shown in Table C3. Particularly notable are F13 (6.03E + 03) and F14 (1.79E + 03) where AEHMO performed better than HOA by larger orders of magnitude (4.22E + 10 and 8.84E + 07 respectively). The low standard deviation in F14 (6.01E + 01) indicates near-perfect consistency in 50-dimensional hybrid spaces. For composite functions (F21-F30), AEHMO got first rank in 7 out of 10 cases, F21 (2.50E + 03), F22 (7.86E + 03), F24 (3.08E + 03), F25 (3.08E + 03), F26 (6.75E + 03), F27 (3.20E + 03), F29 (4.51E + 03).

**Fig. 6:**
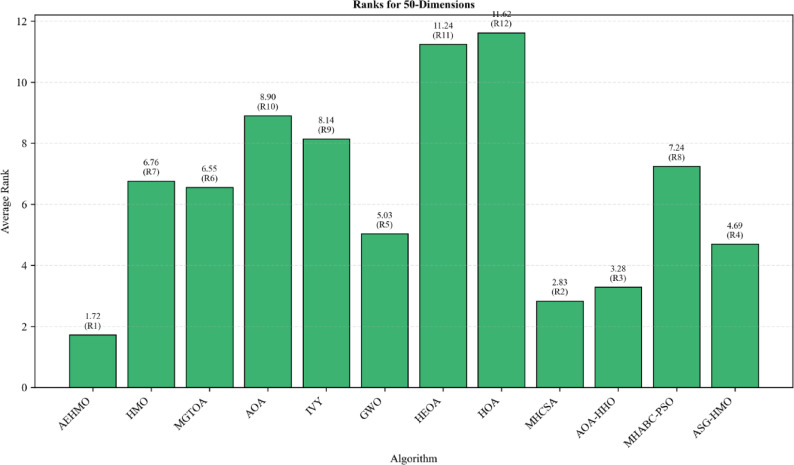
Average ranks of various algorithms using CEC2017, 50D.

Despite the fact that on the whole, AEHMO achieved better performance with an average rank of 1.72 in 50 dimensions as shown in Fig. 6, AEHMO did have some failures that required analysis. In F1 third rank position (2.80E + 06) is the position of degradation from first rank in both 10D and 30D scenarios, AOA-HHO (7.80E + 03) and MHCSA (8.21E + 03), which indicates that the expanded unimodal landscape of 50-dimensional function favors the algorithm using harmony search and hybrid hawk strategies which allow better population to spread. The large standard deviation value of 2.35E + 06 as compared to AOA-HHO’s 2.89E + 03 is a signal of inconsistent converging, presumably due to premature transition of adaptive parameters from exploration to exploitation before adequately exploring the vastly expanded 50-dimensional space. In multimodal functions, F8 had the third highest rank (1.11E + 03) preceded by ASG-HMO (1.00E + 03) and GWO (1.02E + 03), which suggested existence of occasional convergence variability in 50-dimensional functions. For hybrid function F11, the third-rank result (1.36E + 03) behind AOA-HHO (1.27E + 03) and MHCSA (1.30E + 03) indicates that there are some hybrid compositions with 50 dimensions that are supported by other mechanisms for diversity maintenance. The composite function failures are seen in the case of F17, F20, F23 and F28 where AEHMO got the second, fourth, fifth and fourth ranks, respectively. The F20 fourth-rank position (2.98E + 03) is the most important composite function failure, where MHCSA (2.96E + 03), ASG-HMO (2.97E + 03) and GWO (2.98E + 03) have outperformed AEHMO by small margins which indicate that there are near equivalent performances but with AEHMO showing slightly higher variance. In F23, AEHMO was ranked 5th (3.15E + 03) behind MHCSA (3.00E + 03), GWO (3.00E + 03), AOA-HHO (3.05E + 03) and ASG-HMO (3.08E + 03), showing that the landscapes formed by 50-dimensional composition functions combining Ackley and Rastrigin components are such that memory-based mechanisms have difficulty in keeping the exploration-exploitation balance reached in lower dimensions. The fact that the F28 4th rank (3.51E + 03) result is behind AOA-HHO (3.31E + 03), MHCSA (3.31E + 03) and HMO (3.40E + 03) shows that the expansion of the composition function to 50 dimensions has introduced complexity where the simpler approach of the original HMO (i.e. fixed parameters) sometimes outperforms the adaptive mechanism of AEHMO, suggesting that the parameter adaptation needs to be dimensionally scaled to avoid excessive exploration or premature convergence in ultra-high-dimensional compositional structures.

#### Result analysis using CEC2017, 100D

In the high-dimensional case of 100D, AEHMO proved to be continuously effective, exhibiting different performance characteristics from those observed in lower dimensions such as shown in Table C4. For unimodal functions F1 and F3, AEHMO achieved the fifth and first ranks with fitness value of 2.87E + 09 and 2.44E + 05, respectively. In F1, AEHMO’s fifth-rank position behind MHCSA (6.37E + 04), AOA-HHO (8.95E + 04), HMO (6.21E + 08), ASG-HMO (7.39E + 08) is a significant departure from former dimensional trends, suggesting that high-dimensional unimodal functions favor algorithms whose population diversity mechanisms differ from AEHMO’s memory-based approach. However, AEHMO’s first-rank performance in F3 (2.44E + 05) confirms the maintained superiority in dealing with function structure in all the dimensions tested. For multimodal functions (F4-F10), AEHMO came first for five of the functions (F4 (7.35E + 02), F5 (1.11E + 03), F6 (6.43E + 02), F7 (1.87E + 03) and F9 (2.02E + 04) and second for F8 (1.53E + 03) behind ASG-HMO (1.37E + 03) and seventh for F10 (2.41E + 04).

For hybrid functions (F11-F20), AEHMO got first rank in seven out of ten cases: F12 (2.61E + 07), F14 (2.48E + 05), F15 (5.04E + 03), F16 (5.42E + 03), F17 (4.92E + 03), F18 (6.35E + 05), and F19 (6.56E + 03), with good performance in high dimensional hybrid spaces as shown. For composite functions (F21-F30), AEHMO got the 1 st rank in four cases (F22 (1.94E + 04), F24 (3.95E + 03), F25 (3.29E + 03), F26 (1.63E + 04)), and 2nd rank at F21 (2.96E + 03).

**Fig. 7:**
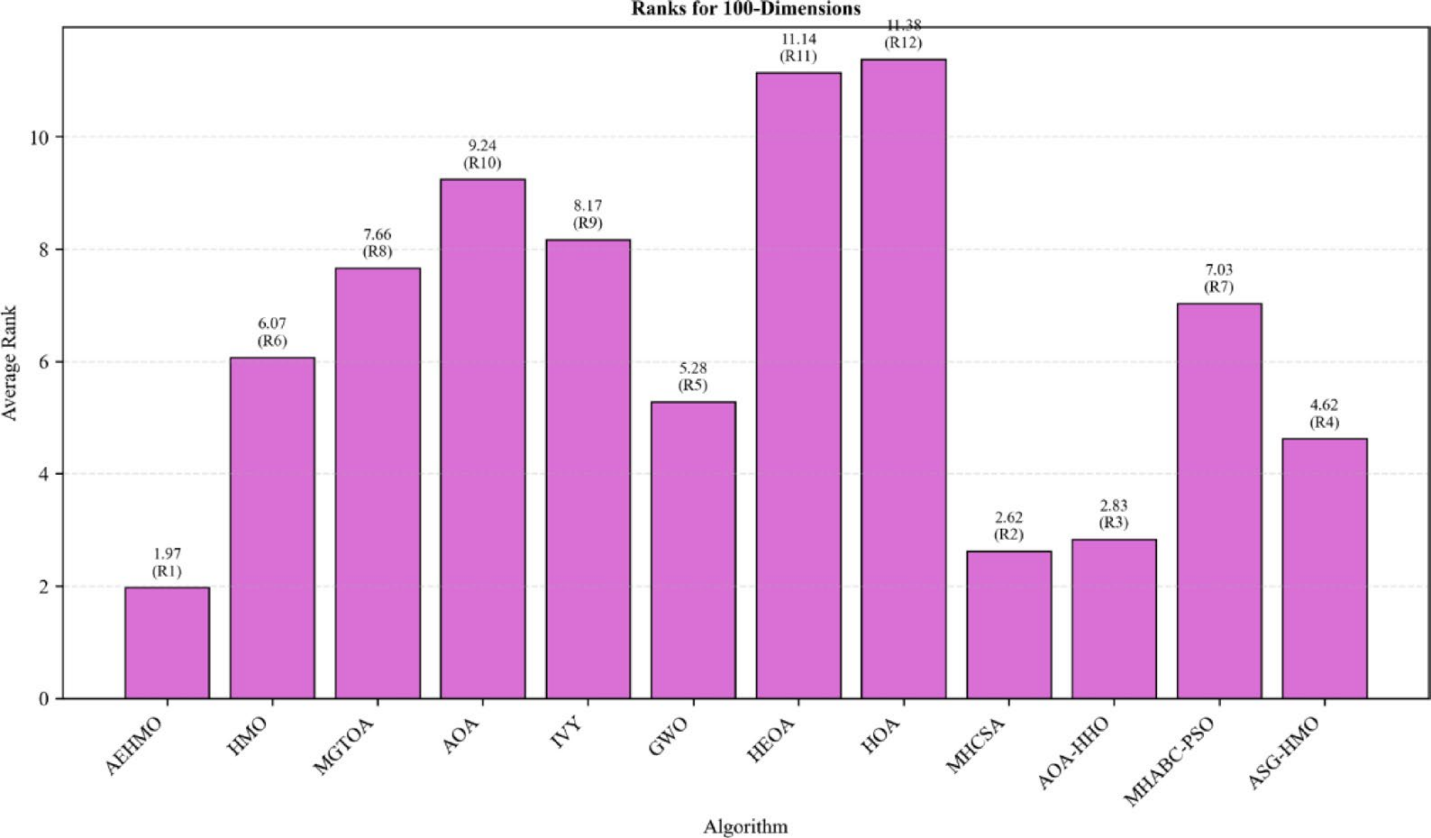
Average ranks of various algorithms using CEC2017, 100D.

AEHMO’s overall average rank of 1.97 in 100 dimensions as shown in Fig. 7, while slightly higher than 1.72 found in 50D and 1.45 found in 30D, confirms good scalability to high dimensional optimization with competitive advantages over most comparison algorithms. The dimensional scaling analysis shows the presence of important performance limitations approaching dimensionality 100. In unimodal function F1, the significant decline of function value from first rank in 10D, 30D to fifth rank in 100D (2.87E + 09) in which MHCSA (6.37E + 04) and AOA-HHO (8.95E + 04) obtained fitness values four orders of magnitude better, implies the adaptive parameter mechanism of AEHMO cannot manage to effectively balance exploration and exploitation when searching for a solution in high dimensional smooth landscapes, whose high standard deviation of 1.04E + 09 implies high variance. In hybrid function F11, the third-rank position (1.46E + 04) and relatively high standard deviation (4.68E + 03) in comparison with MHCSA (2.45E + 03, SD: 1.25E + 02) and AOA-HHO (2.59E + 03, SD: 2.76E + 02) shows the consistency problems in 100-dimensional compositions. The composite function failures in F20, F23 and F28 in the third, fifth and fourth ranks respectively indicates the systematic difficulties. The F23 fifth-rank result (3.90E + 03) which is behind AOA-HHO (3.33E + 03), MHCSA (3.40E + 03), ASG-HMO (3.57E + 03) and GWO (3.69E + 03) shows that the 100-dimensional compositional landscapes are exploration challenges in which the chaotic mapping intensity is not enough to traverse the exponentially expanded search space. The F28 fourth-rank position (4.45E + 03) where AOA-HHO (3.46E + 03), MHCSA (3.46E + 03), and HMO (4.01E + 03) scored the better than AEHMO suggests the existence of certain 100-dimensional compositional structures that paradoxically favor the simpler fixed parameter approach of the original HMO, and also reveals how adaptive mechanisms designed for lower dimensions, sometimes introduce unnecessary complexity or computational overhead for high-dimensional spaces. These systematic failures in unimodal, multimodal, hybrid and composite categories suggest that the current parameter settings of AEHMO especially the fixed chaotic mapping iterations and mutation step sizes and drift intensity coefficients need to have explicit dimensional-adaptive scaling mechanisms to automatically adapt the intensities of strategies based on the problem dimensionality to ensure optimal performance when moving from 50D to 100D and higher since the current linear or sigmoid based parameter adaptations become increasingly inadequate when dealing with the exponential increase in complexity of the search space and the curse of dimensionality effects.

#### Convergence analysis

Convergence behavior analysis across all CEC2017 function classes and dimensions (Figs. D1–D4) demonstrates AEHMO’s consistent ability to balance early exploration with late-stage exploitation throughout the 30,000 function evaluations. For unimodal functions F1 and F3, AEHMO exhibits the steepest initial descent across all dimensions, driven by the DDS strategy enabling rapid identification of promising basins in the first 5,000 FEs, followed by smooth transition to exploitation via adaptive parameter regulation around 10,000 FEs. For multimodal functions, AEHMO consistently falls below all competitors by 8,000–12,000 FEs in F4, F6, F7, and F9 across all dimensions, with the step-like descent patterns in F8 and F10 confirming that the CRL chaotic opposition mechanism successfully escapes successive local optima where competitor algorithms stagnate. Hybrid functions reveal AEHMO’s most sustained convergence behavior, with uninterrupted descent maintained beyond 15,000 FEs in F12, F13, F14, F15, F18, and F19, a phase where all competing algorithms exhibit visible plateauing. Composite functions demonstrate the strongest convergence advantage, with AEHMO maintaining a continuously widening gap over competitors in F22, F24, F25, F26, F27, F29, and F30 throughout the full evaluation budget. The primary convergence limitations emerge at 100D, where F1, F5, F10, F23, and F28 exhibit oscillatory or premature flattening behavior, suggesting that the current adaptive parameter settings encounter dimensional scaling challenges in specific high-dimensional unimodal and composite landscapes. Full convergence curves for all functions and dimensions are provided in Supplementary Appendix D.

#### Boxplot analysis

Boxplot analysis across all CEC2017 function classes and dimensions (Figs. E1–E4) confirms the high solution consistency and robustness of AEHMO across 30 independent runs. For unimodal and multimodal functions, AEHMO produces highly compact distributions with near-zero interquartile ranges in 10D, 30D, and 50D, outperforming competitors such as IVY, AOA, HEOA, and HOA which exhibit significantly larger box heights, heavy-tailed distributions, and frequent outliers. For hybrid functions, AEHMO consistently achieves the smallest interquartile ranges across F12, F13, F15, F18, and F19 in all tested dimensions, confirming that the MDMS elite mutation strategy effectively maintains solution quality consistency without population-wide perturbation. Composite functions reveal AEHMO’s strongest robustness characteristics, with distributions for F22, F24, F25, F26, F27, F29, and F30 collapsing to near-horizontal lines in 10D-50D, reflecting stable convergence across all runs regardless of initialization. The only notable variance increases occur at 100D for F1, F5, F10, F23, and F28, where higher dimensionality introduces initialization-dependent convergence behavior that AEHMO’s current adaptive mechanisms do not fully suppress. A detailed per-function boxplot analysis across all dimensions is provided in Supplementary Appendix E.

#### Statistical significance analysis

**Fig. 8:**
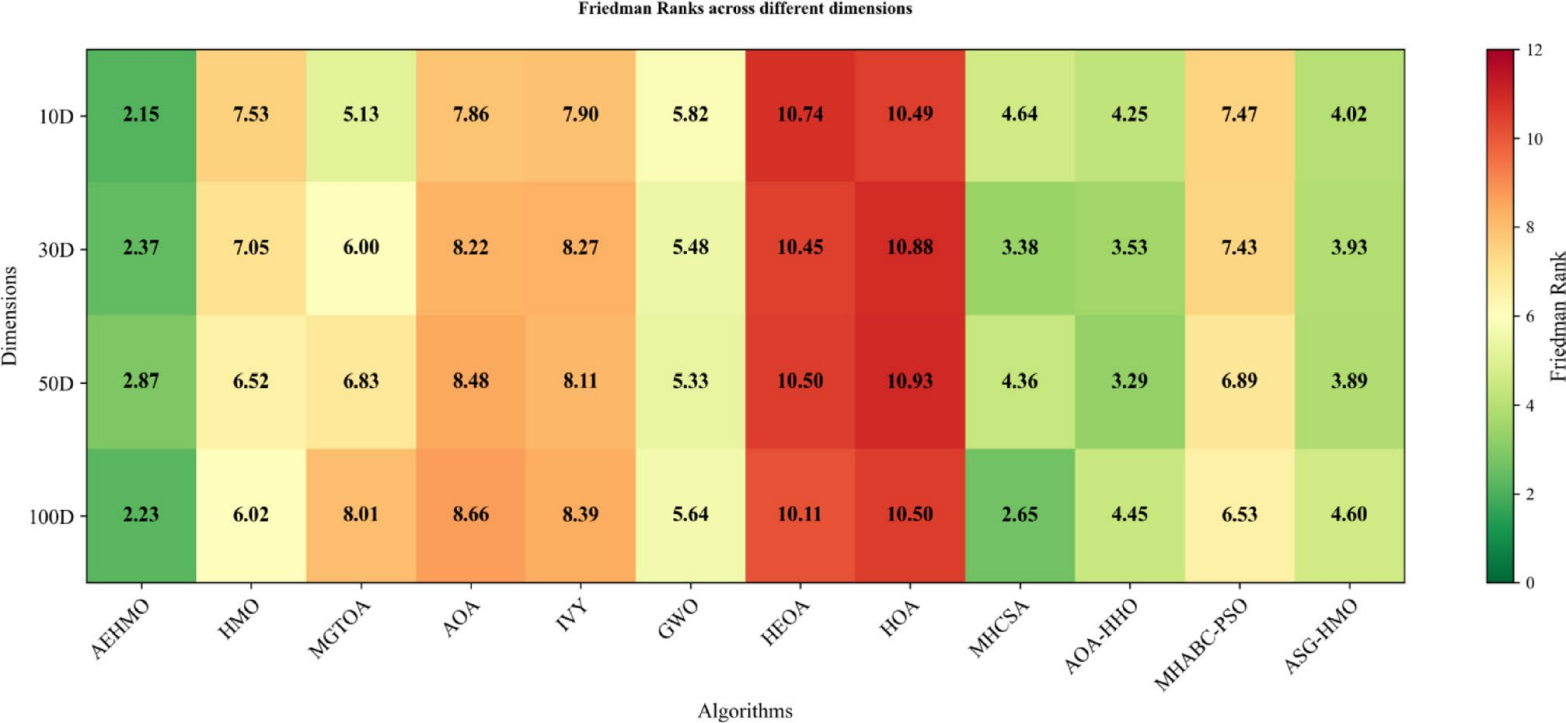
The Friedman ranks of various algorithms across various dimensions.

**Fig. 9:**
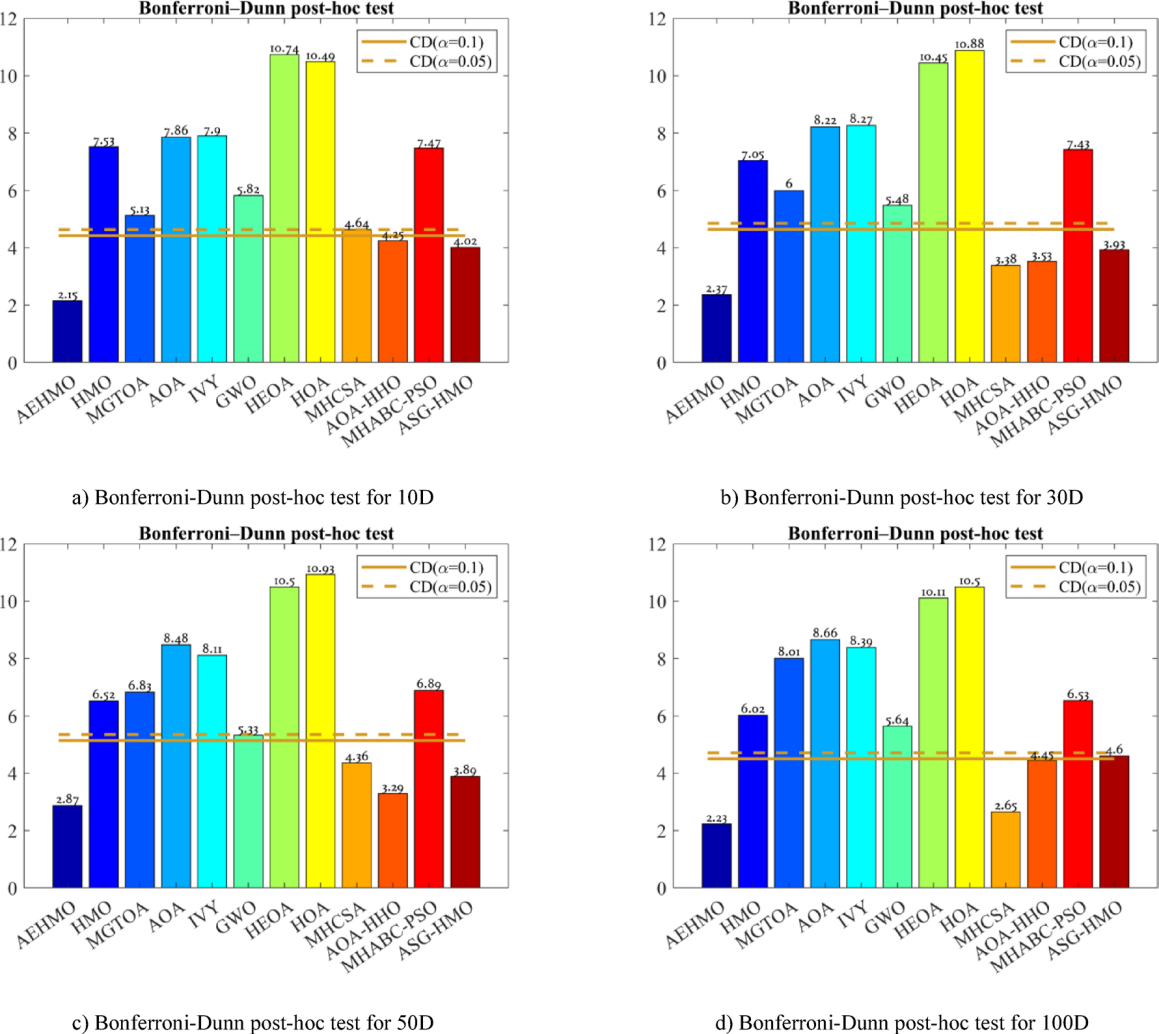
Bonferroni-Dunn post-hoc test of various algorithms across various dimensions.

The result of the Friedman test in all the dimensional scenarios consistently indicate the statistical competitiveness of AEHMO over the comparison algorithms with the algorithm having the best mean ranks in the 10D, 30D, 50D and 100D as shown in Fig. 8. In 10-dimension optimization, AEHMO had a Friedman rank of 2.15 which is far better than all the competitors, the other best competitors were ASG-HMO (4.02), AOA-HHO (4.25) and MHCSA (4.64), while traditional algorithms such as HMO (7.53), HEOA (10.74) and HOA (10.49) showed much weaker overall performance. This ranking pattern indicates that AEHMO is consistently better than other algorithms in the low dimensional spaces of the diverse CEC2017 benchmark suite by having top or near top ranks in most of the 29 test functions. The results of the Bonferroni-Dunn post-hoc test shown in Fig. 9 indicate that the critical difference (CD) values of AEHMO algorithm at both $\:\alpha\:$=0.1 and $\:\alpha\:$=0.05 significance levels place the algorithm well below the threshold lines, showing statistically significant improvement over HMO, MGTOA, AOA, IVY, GWO, HEOA, HOA and MHABC-PSO. The dramatic performance gap between AEHMO and algorithms such as HEOA 10.74 and HOA 10.49 suggests that it is challenging for recent nature-inspired algorithms without adaptive parameter mechanisms to effectively balance exploration and exploitation in the benchmark suite.

As the dimensionality is increased to 30D, 50D and 100D AEHMO maintains its statistical advantage with Friedman ranks of 2.37, 2.87 and 2.23, respectively as shown in Fig. 9. The 30D Friedman ranks show AEHMO continuing to outperform all competing algorithms, with MHCSA (3.38), AOA-HHO (3.53) and ASG-HMO (3.93) forming the next level of competition, with HEOA (10.45) and HOA (10.88) still falling at the bottom of the rankings. The post-hoc test for 30D using the Bonferroni-Dunn confirms the statistical significance of AEHMO’s bar that is well below both CD thresholds, indicating that the observed performance differences are statistically significant. Interestingly, the 50D Friedman ranks have AEHMO with a rank of 2.87, which is slightly higher than the 30D rank (2.37), suggesting a modest reduction in relative performance with increasing dimensionality, but again, AEHMO still has significant advantages over most of its contenders. The Bonferroni-Dunn results for 50D still show AEHMO to be below CD thresholds, with AOA-HHO (3.29) and ASG-HMO (3.89) having the nearest competitive performance. A notable observation emerges in the 100D case where AEHMO achieves a surprisingly competitive Friedman rank of 2.23, which is comparable to its 10D rank of 2.15, suggesting that AEHMO scales effectively to very high-dimensional problems without significant performance degradation with MHCSA (2.65) the closest competitor with a minimum difference of 0.42 rank points. The Bonferroni-Dunn post-hoc test for 100D tells us that AEHMO and MHCSA fall within the critical difference (CD, $\:\alpha\:$ = 0.05), indicating no statistically significant difference between them. The coexistence of AEHMO and MHCSA performance in 100D, and AOA-HHO (4.45) and ASG-HMO (4.60) preserving competitive ranks implies that high-dimensional optimization provides conditions where several well-designed algorithms with different underlying mechanisms are able to achieve similar overall performance for different function types. Despite this convergence, algorithms like HEOA (10.11–10.74), HOA (10.49–10.88), AOA (7.86–8.66) and IVY (7.90–8.39) consistently score in the bottom half in all the dimensions with their bars in all Bonferroni-Dunn charts placed significantly above both CD thresholds, indicating statistically inferior performance relative to AEHMO in all dimensional scenarios tested and that not all nature-inspired metaheuristics perform similarly on complex optimization benchmarks.

**Fig. 10:**
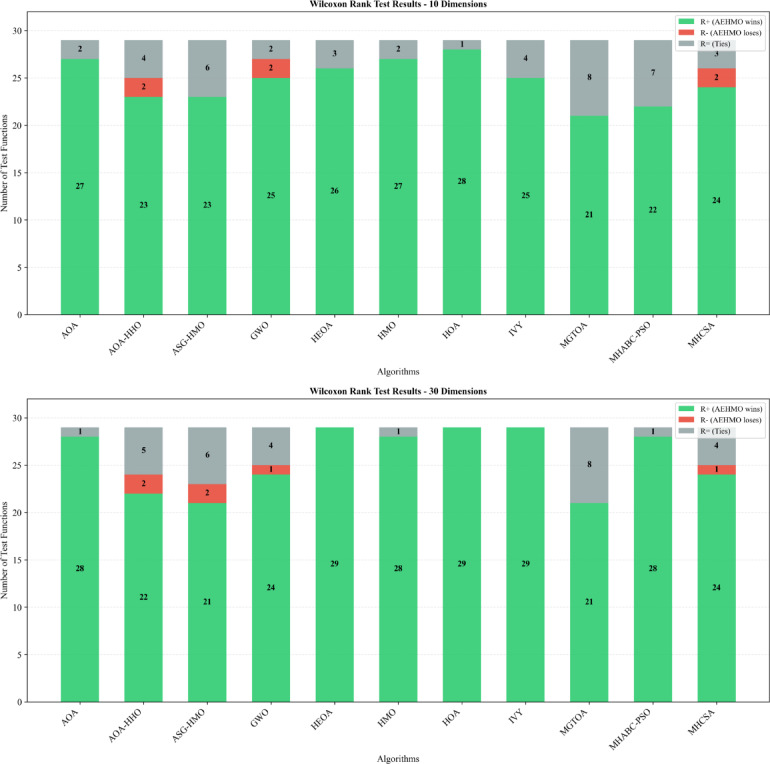

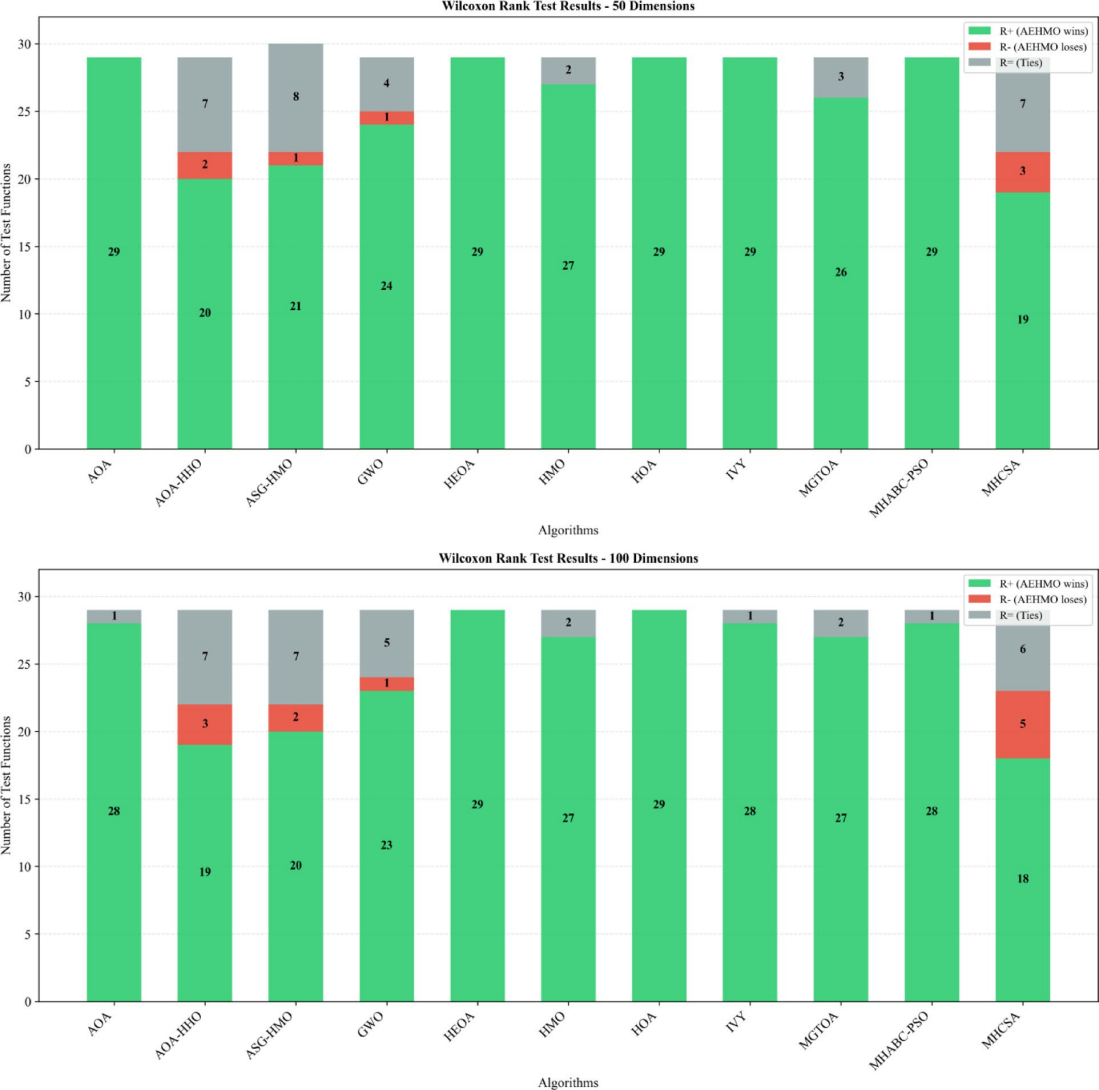
Wilcoxon test of AEHMO versus other algorithms using CEC2017.

The results of the Wilcoxon rank test are used to provide pairwise statistical comparisons between AEHMO and all its competing algorithms for all the dimensional scenarios to confirm the dominance of AEHMO with overwhelmingly positive win-loss ratios as presented in Fig. 10. In optimization of 10-dimension AEHMO was found to be consistently superior to AOA (R + = 27, R-=0, R = = 2), HMO (R + = 27, R-=0, R = = 2), HEOA (R + = 26, R-=0, R = = 3), HOA (R + = 28, R-=0, R = = 1), IVY (R + = 25, R-=0, R = = 4), and MHABC-PSO (R + = 22, R-=0, R = = 7) and showed statistically significant dominance. As the dimensionality increased to 30D, the domination of AEHMO increased, exhibiting perfect or near-perfect dominance over most algorithms: AOA (R + = 28, R-=0), HEOA (R + = 29, R-=0), HMO (R + = 28, R-=0), HOA (R + = 29, R-=0), IVY (R + = 29, R-=0), and MHABC-PSO (R + = 28, R-=0), with good advantages against competitive algorithms with MGTOA (R + = 21, R-=2). The consistent pattern for all dimensions shows that AEHMO is reporting R+ values from 18 to 29 (out of maximum 29 functions) in comparison with all the competitors with R- values never exceeding 5 and mostly staying at 0–2, while algorithms such as HEOA, HOA, AOA and IVY are consistently reporting R− = 0 for all dimensions, indicating that AEHMO does not underperform relative to these methods for each function and validates the statistical significance and robustness of AEHMO’s superior performance for the entire CEC2017 benchmark suite across all tested dimensions.

The vast statistical analysis of AEHMO at various dimensional scales (10D, 30D, 50D and 100D) on the CEC2017 benchmark suite shows an outstanding algorithmic scalability and performance advantage. The 95% confidence interval analysis shown in Table F1 shows a high level of consistency in the accuracy of algorithms with AEHMO having much smaller margins of error than competitor algorithms where notable stability in the performance of the algorithm going from low dimensions to extreme high dimensions search spaces. This continued precision in spite of exponential growth in complexity of search space with increasing dimensionality provides evidence that AEHMO’s enhancement mechanisms are effective in mitigating the curse of dimensionality. The Wilcoxon signed-rank test is applied to check statistical superiority in all the dimensional scales and all pairwise comparisons are statistically significant ($\:p$ < 0.001), indicating that AEHMO is statistically better than competing algorithms regardless of the problem’s dimensionality.

Moreover, the results of effect size analysis presented in Table F2 reveal that there is substantial practical significance in all dimensions with mean effect size $\:r$ ranging between 0.853 and 0.873, which indicates that the effect sizes are large and performance differences are not only statistically significant but also have a large practical significance. AEHMO demonstrated superior performance compared to the established algorithms, AOA, HEOA, HMO, HOA, IVY, MGTOA and MHABC-PSO for all the dimensional levels while maintaining good performance advantages against the most competitive algorithms such as GWO, MHCSA, AOA-HHO and ASG-HMO in the whole dimensional range. The narrow CIs and consistently large effect size in all dimensional scales gives strong statistical evidence that AEHMO’s integrated enhancement strategies (adaptive parameter regulation, multi-directional mutation, DDS and CRL) exhibit strong scalability properties, supporting the robustness and reliability of AEHMO for complex real-world optimization problems involving varying dimensionality, multimodality, and challenging fitness landscapes.

#### Computational overhead analysis

Computational efficiency is a critical factor for the practical deployment of metaheuristic algorithms, particularly in resource-constrained scenarios such as WSN CH selection where optimization must operate under strict computational budgets. Computational time analysis across different dimensional settings (10D, 30D, 50D and 100D) on the CEC2017 benchmark suite demonstrates a balanced trade-off achieved by AEHMO in terms of computational efficiency and optimization performance. As reported in Table 4, AEHMO has a moderate execution time (0.5603, 0.9399, 1.2934 and 2.9225 s) for 10D, 30D, 50D and 100D problems, respectively, placing it within the mid-range of the compared algorithms. While AEHMO incurs higher runtime than lightweight single-strategy algorithms such as ASG-HMO, MGTOA, GWO, and HOA, its execution times remain moderate across all tested dimensions, as shown in Table 4. Notably, AEHMO exhibits significantly lower computational overhead compared to MHABC-PSO across all tested dimensions, while maintaining comparable execution times to AOA-HHO and MHCSA, while delivering substantially superior optimization performance as reflected by lower Friedman ranks.

**Table 4:** Computational overhead of various algorithms at various dimensions.

	AEHMO	HMO	MGTOA	AOA	IVY	GWO	HEOA	HOA	MHCSA	AOA-HHO	MHABC-PSO	ASG-HMO
10D	0.5603	0.3940	0.1912	0.4872	0.3019	0.2186	0.4936	0.2163	1.1673	1.1288	0.9161	0.1871
30D	0.9399	0.6354	0.3647	0.9101	0.5343	0.4589	0.7138	0.3310	1.4179	1.4474	1.9126	0.3739
50D	1.2934	0.8719	0.5144	1.1770	0.6868	0.6724	0.8638	0.4368	1.6348	1.6635	2.5695	0.5280
100D	2.9225	1.9446	1.1539	2.2551	1.3445	1.4383	1.5627	0.9708	2.9693	2.7688	4.8058	1.1889

### AEHMO for engineering applications

In this section, the performance of the proposed AEHMO algorithm is investigated under realistic engineering design conditions, which include eight widely used structural and mechanical design problems: Piston Lever Design, Tension–Compression Spring Design, Tubular Column Design, Cantilever Beam Design, Corrugated Bulkhead Design, I-Beam Design, Speed Reducer Design, and Pressure Vessel Design. Constraint handling of these design tasks is performed by means of a static penalty formulation provided in Eq. (29)^61^:

29 $$\:\begin{array}{c}\boldsymbol{\zeta\:}\left(\boldsymbol{z}\right)\\\:=\boldsymbol{f}\left(\boldsymbol{z}\right)\pm\:\left[\sum\:_{i=1}^{m}\:\:{\boldsymbol{l}}_{i}\cdot\:\boldsymbol{m}\boldsymbol{a}\boldsymbol{x}{\left(0,{\boldsymbol{t}}_{i}\left(\boldsymbol{z}\right)\right)}^{\alpha\:}+\sum\:_{j=1}^{n}\:\:{\boldsymbol{o}}_{j}{\left|{\boldsymbol{U}}_{j}\left(\boldsymbol{z}\right)\right|}^{\beta\:}\right]\end{array}$$

Here, $\:z$ is the penalized objective function where $\:{o}_{j}$, $\:{l}_{j}$ are positive constant penalty. In this formulation, the constraints are denoted by $\:{U}_{j}\left(z\right)$ and $\:{T}_{i}\left(z\right)$, and the penalty effect is governed by the parameters $\:\alpha\:$ and $\:\beta\:$, which respectively assume values 1 or 2. All the test problems are solved using population size 50, maximum iterations 500, and 30 independent runs to achieve a good statistical analysis.

#### Piston Lever Design (PLD) problem

The PLD problem is a minimization problem to minimize the amount of oil used when the piston lever changes its angle from 0^o^ to 45^[o 62^. The effect of geometrical parameters $\:H$, $\:B$, $\:D$ and $\:V$ is considered for the optimization problem which has a great influence on the mechanical efficiency. This problem can be mathematically described as in the Supplementary Appendix.

AEHMO performs the best among all the other algorithms on all the statistical measures as shown in the evaluation of the PLD problem. Table 5 indicates the minimum cost with a low standard deviation for AEHMO is 8.645928 and 0.807653 respectively, meaning that there is high consistency and reliability of finding the best solutions. The best performance (global optimum) of the problem is 8.416798 and the worst-case performance is 12.058200 which is a significant improvement over all other competing algorithms. The fact that the difference between optimal and worst scores is small suggests that the algorithm is robust and less prone to be stuck in local optima and/or not converging.

**Table 5:** The statistical results of various algorithms for PLD problem.

Algorithm	Mean Cost	Std Cost	Best Score	Worst score
AEHMO	8.645928	0.807653	8.416798	12.058200
HMO	111.856559	91.146574	8.435053	278.385039
HOA	200.143511	276.709927	33.156730	830.312182
HEOA	1127.929302	1040.832753	310.434324	4721.277015
AOA	397.772499	111.694767	212.203019	522.444955
IVY	16.403635	35.622673	8.417480	167.747581
MGTOA	229.415770	98.875139	167.472730	461.316707
GWO	88.118756	81.758231	8.417944	168.081983
MHABC-PSO	224.662151	135.152572	8.948950	422.354716
MHCSA	56.130708	74.784038	8.412698	167.472730

#### Tension and compression spring design (TCSD) problem

The TCSD is concerned with the minimization of the weight of a spring subject to a number of engineering constraints^63^. The optimization involves three key design parameters, namely wire diameter $\:d\left({x}_{1}\right)$, mean coil diameter $\:D\left({x}_{2}\right)$ and number of active coils $\:N\left({x}_{3}\right)$. To maximize the performance, these variables need to be tuned to meet allowable stress, permissible deflection, and structural integrity constraints along with the provision of reliable performance under both tensile and compressive loads to ensure material efficiency and durability. This problem is a standard example in mechanical design, and it is often used for testing the robustness of algorithms under nonlinear and constrained loads. This problem can be mathematically described as in the Supplementary Appendix.

The design optimization of the TCSD problem highlights the strong optimization performance of AEHMO, as shown in Table 6. AEHMO exhibits excellent convergence with a mean fitness value of 0.012665233 and a standard deviation numerically equal to zero. This high level of stability provides strong evidence for the reliability and robustness of the proposed improvements. The best and the worst fitness values of the algorithm are equal (0.012665233), which proves that there is no performance variability, which is an important requirement for engineering applications where the optimization result should be predictable. As for the comparison of the competing algorithms, the IVY and MGTOA are relatively competitive algorithms with the mean fitness value of 0.012738194 and 0.012711070, respectively, with some fluctuation. The mean fitness of HMO is 0.014047824 and the standard deviation is 0.001622293, indicating that the convergence consistency still needs to be improved.

**Table 6:** The statistical results of various algorithms for TCSD problem.

Algorithm	Mean Fit	Std Fit	Best Fit	Worst Fit
AEHMO	0.012665233	0.000000000	0.012665233	0.012665233
HMO	0.014047824	0.001622293	0.012667564	0.017783713
HOA	0.013631616	0.000899345	0.012707751	0.016122584
HEOA	0.015989949	0.002509437	0.013057824	0.022125319
AOA	0.013206527	0.000062959	0.013009399	0.013380081
IVY	0.012738194	0.000051036	0.012706276	0.012951421
MGTOA	0.012711070	0.000098801	0.012665233	0.013047499
GWO	0.012714615	0.000013814	0.012680463	0.012725769
MHABC-PSO	0.013360846	0.000836978	0.012722417	0.015260720

#### Tubular column design (TCD) problem

The TCD problem is to find the best design of a uniform column which will support a given compressive load $\:P$ (pounds/inch^2^) with minimum total cost of manufacture^64^. Design variables are the external diameter $\:{t}_{1}$, and the wall thickness $\:{t}_{2}$. The column is made of material with an elastic modulus of 0.85 × 10^6^ kgf/cm^2^ and yield stress limit of 500 kgf/cm^2^. The goal is to find optimal $\:{t}_{1}$ and $\:{t}_{2}$ which ensure the structural stability under compression without yielding and instability. Moreover, it is a representative problem involving a trade-off between mechanical performance and resource efficiency, as designers must balance cost-effectiveness, strength and stiffness requirements, and material savings. This problem can be mathematically described as in the Supplementary Appendix.

The results of the TCD problem, summarized in Table 7, demonstrate the strong optimization performance of AEHMO. The algorithm exhibits highly consistent convergence, with a mean cost of 26.4863615 and a numerically negligible standard deviation of 3.6450110E-15, meaning the best and worst scores were identical across all 30 independent runs. This level of consistency indicates highly stable convergence and suggests improved reliability and accuracy of the proposed enhancements for constrained structural design problems with mixed continuous and boundary variables. Performance comparisons show that despite the other algorithms might show competitive results from time to time, none can compete with the consistency of AEHMO.

**Table 7:** The statistical results of various algorithms for TCD problem.

Algorithm	Mean Cost	Std Cost	Best Score	Worst score
AEHMO	26.4863615	3.64501E-15	26.4863615	26.4863615
HMO	26.4923143	0.004502627	26.4868365	26.5030053
HOA	26.5236547	0.046938111	26.4864750	26.6614788
HEOA	27.1388694	0.564430148	26.5431719	28.6124023
AOA	27.7888622	0.594661813	26.7039852	28.5803989
IVY	26.4966147	0.005760624	26.4886873	26.5104257
MGTOA	26.6011517	0.448478196	26.4863615	28.4858053
GWO	26.4904312	0.002234758	26.4883592	26.4953295
MHABC-PSO	26.4897804	0.002139351	26.4871803	26.4957765

#### Cantilever beam design (CBDP) problem

The CBDP is intended to optimally minimize the total weight of a cantilever suspension arm subject to structural constraints^65^. The widths of these five segments are the optimization variables and the cross-sectional thickness is uniform. This issue is of great importance for structural optimization, which is a problem of reducing material usage without losing the mechanical performance to support loads and moments applied at the free end. It is used as a test problem for optimizing algorithms in multi-variable constrained and weight-sensitive design problems. This problem can be mathematically described as in the Supplementary Appendix.

The effectiveness of CBDP problem is analyzed and excellent optimization performance of AEHMO is presented in Table 8. AEHMO showed near-identical results across runs, with a mean cost of 1.33995636 and a very low standard deviation of 4.55626E-16, meaning that the best and worst scores were the same value in all independent runs. This high level of consistency suggests that the algorithm repeatedly converges to the same high-quality solution. These results highlight the enhanced reliability and accuracy of the proposed strategies for solving multi-variable constrained beam design problems with complex stress and deflection constraints. Comparative analysis shows performance gaps that are significant compared to competitors. The best performance is obtained by GWO with the cost value of 1.34005715 and small standard deviation value of 6.54247E-5 and IVY with the cost value of 1.34020248 is the second-best performance.

**Table 8:** The statistical results of various algorithms for CBDP problem.

Algorithm	Mean Cost	Std Cost	Best Score	Worst score
AEHMO	1.33995636	4.55626E-16	1.33995636	1.33995636
HMO	1.39169817	0.035926493	1.34990649	1.51589519
HOA	1.34534178	0.005103276	1.34007850	1.35686688
HEOA	3.26390871	0.889777854	1.59447941	5.36119627
AOA	2.20779108	0.887194939	1.42452907	5.39635503
IVY	1.34020248	0.000191639	1.33997108	1.34071530
MGTOA	1.35605597	0.046723261	1.33995636	1.49761460
GWO	1.34005715	6.54247E-05	1.33997655	1.34021590
MHABC-PSO	1.34329207	0.003586179	1.34048199	1.35739688

#### Corrugated bulkhead design (CBD) problem

The CBD problem is to reduce the weight of a structural bulkhead made by deforming a pressed steel plate, and not requiring the use of external stiffeners^66^. In this formulation, the stiffness is geometrically introduced into the plate through a corrugation structure. The optimization has four primary design variables: corrugation width $\:{x}_{1}$, depth $\:{x}_{2}$, length $\:{x}_{3}$ and plate thickness $\:{x}_{4}$. The design has to satisfy six constraint conditions for structural adequacy and performance in the various load cases. This problem is a representative form-optimization task for improving structural efficiency and has been widely used to evaluate algorithm performance on multi-constraint, multi-variable engineering design problems. This problem can be mathematically described as in the Supplementary Appendix. Table 9 illustrates the excellent optimization capability of the proposed AEHMO algorithm over other methods for CBD Problem. AEHMO converged with very high consistency, with a mean cost of 6.84295801 and a numerically negligible standard deviation of 1.8225E-15, indicating that there was effectively no variation across the optimization runs. The performance of AEHMO is compared with that of competing algorithms in this challenging structural optimization problem revealing significant gaps in performance. The original HMO solution had a decent solution (mean cost = 6.88164711, std = 0.033774446), the best solution of 6.84618290 is not that far from the optimal. In contrast, the performances of HEOA and AOA were significantly worse with mean costs of 8.79781954 and 7.92043225, respectively and high standard deviations which signifies high inconsistency and convergence difficulties.

**Table 9:** The statistical results of various algorithms for CBD problem.

Algorithm	Mean Cost	Std Cost	Best Score	Worst score
AEHMO	6.84295801	1.8225E-15	6.84295801	6.84295801
HMO	6.88164711	0.033774446	6.84618290	6.95924857
HOA	7.24255394	0.207050892	6.98343698	7.81776010
HEOA	8.79781954	1.058492364	6.99392534	10.65211231
AOA	7.92043225	0.516783383	7.24799472	8.89285190
IVY	6.94727234	0.311100335	6.84858253	8.26175227
MGTOA	6.89904636	0.180498244	6.84295801	7.62976335
GWO	6.89463249	0.196121797	6.84432915	7.72762413
MHABC-PSO	6.91029864	0.074522007	6.85135172	7.11350843

## Application of AEHMO for cluster CH selection in cluster-based routing for wireless sensor networks (WSN)

The strong global optimization performance of AEHMO demonstrated in the section “AEHMO for global optimization” provides a solid foundation for its application to WSN cluster head selection. The CH selection problem is naturally a high-dimensional and multimodal combinatorial optimization problem where the solution space is exponentially large in network size and where there are multiple locally optimal CH configurations. The same capabilities that lead to AEHMO consistently outperforming competing algorithms on CEC2017, namely, adaptive exploration-exploitation regulation, multi-directional mutation for escaping local optima, and chaotic opposition-based diversity preservation, lead to superior CH selection performance. Specifically, the success of avoiding premature convergence in benchmark landscapes of high dimension is directly related to the success of the algorithm navigating the complex CH fitness landscape and finding the energy-efficient configurations consistently for different network sizes and topologies. In this section, the AEHMO algorithm is applied to the Cluster Head (CH) selection problem in cluster-based routing of WSN. The efficient CH selection is crucial to optimize energy consumption and maximize the overall network lifetime. AEHMO is applied to optimize CH selection and thereby enhance overall network performance. This section describes the network model, the energy consumption model for communication, and the performance metrics used to evaluate network efficiency.

### The utilized network model

WSNs have emerged as a foundational technology for interconnected digital systems and smart applications. WSNs play a vital role in enhancing the design of internet-connected applications. The WSN is a multi-hop, self-configured network that comprises many sensor nodes; each node has the potential for wireless communication. These networks integrate several technologies, including distributed data processing, sensing systems, embedded computing, wireless communication, and modern networking, to accomplish complex monitoring and control tasks. Sensor nodes are deployed to gather environmental data, converting physical signals into electrical signals and transmitting them to a central node or sink via wireless multi-hop communication. Applications that can use WSNs include environmental observation, industrial processes, military observation, and healthcare, among many others.

**Fig. 11:**
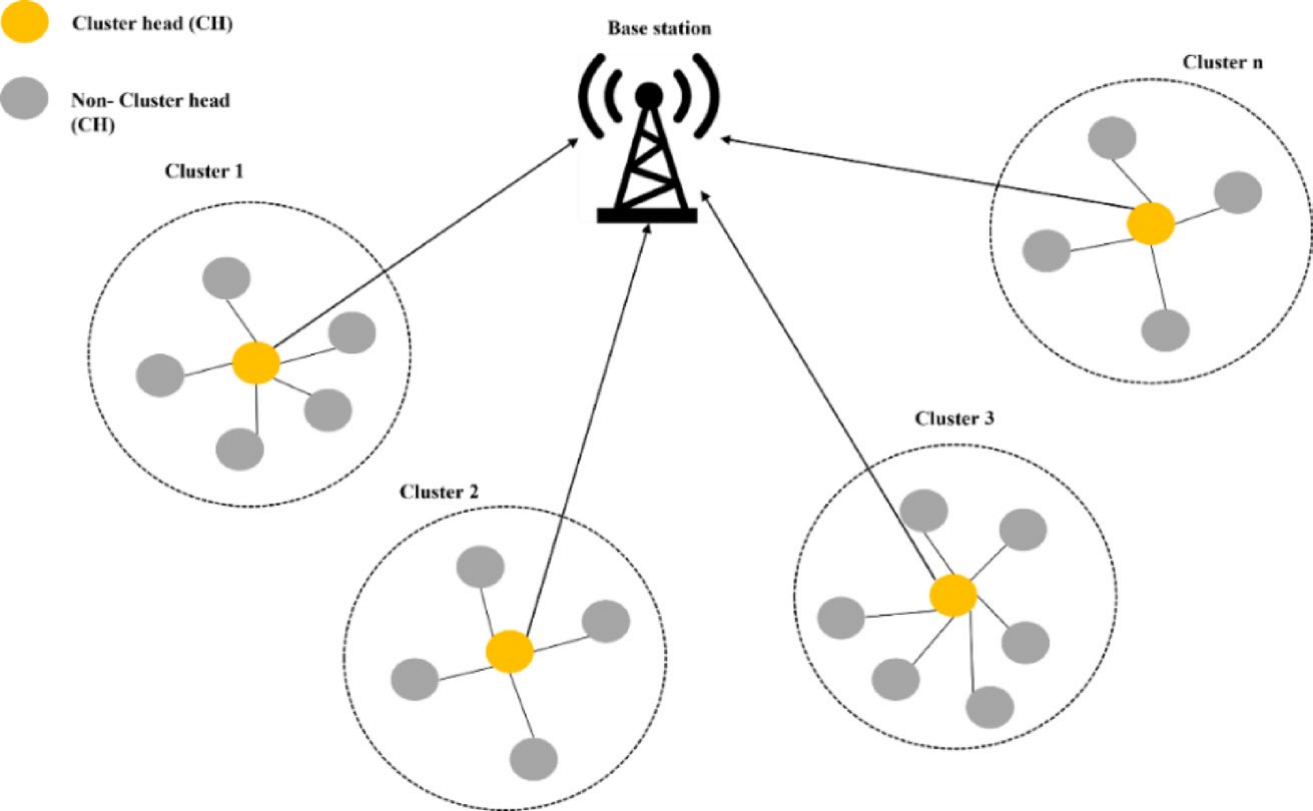
The network model utilized with its components.

The WSN network model consists of several sensors and a sink node. Sensor nodes are uniformly distributed over $\:D\times\:D$ area. Communication between any two nodes takes place either directly via one-hop transmission or through multi-hop routes. Sensor nodes can further be divided into general nodes and source routing nodes. Each node has a unique network identifier and is deployed at a fixed location, while the sink node is positioned at the center of the network. The deployed network requires no interference from human beings afterward. All nodes have the same storage, computing power, and energy capacity. These sensor nodes can identify where they are attached within the network. Since the sink node is not energy-constrained, it possesses greater computational resources than regular sensor nodes. The sensor nodes monitor their residual energy and calculate their distances to neighboring nodes. The node can communicate with the sink node, aggregate data, and change its transmission power according to the distance for its target or recipient. Consequently, the communication links between nodes are symmetric. Each node dynamically adjusts its transmission power according to the distance to the intended recipient. Sensor nodes periodically measure the environment and transmit their data to CH. The network model, including the BS placed within the communication range of all nodes, is illustrated in Fig. 11.

Therefore, the assumptions used in the network model can be summarized as follows:

- All communications are wireless.
- Nodes are scattered randomly in the network.
- Each node has a very limited energy capacity.
- The sink node is also immobile and also has unlimited energy resources.
- CHs can directly communicate with the sink.
- A first-order radio communication model is employed to compute the radio energy dissipation for each node in each round.
- The network operates in rounds.
- Roles of the nodes switch between CH and non-CH nodes at each round start.
- TDMA is used for scheduling the communications between CHs and member nodes’ communications.

### The basic energy model

The energy model is a key factor in analyzing and optimizing power consumption for each node of a network. The whole process depends on the amount of energy taken by the quantity of energy used, for example, transmission, reception, and data aggregation. The components of the energy model are described below:

- Energy consumption for data transmission:

30 $$\:{E}_{\mathrm{T}\mathrm{X}}(p,d)=\left\{\begin{array}{ll}p\times\:{E}_{\mathrm{c}\mathrm{o}\mathrm{m}\mathrm{m}}+p\times\:{\eta\:}_{fs}\times\:{d}^{2}&\:\mathrm{\:if\:}d\le\:{d}_{0}\\\:p\times\:{E}_{\mathrm{c}\mathrm{o}\mathrm{m}\mathrm{m}}+p\times\:{\eta\:}_{mp}\times\:{d}^{4}&\:\mathrm{\:if\:}d>{d}_{0}\end{array}\right.$$

where $\:{E}_{TX}\left(p,d\right)$ is the energy required to transmit $\:p$ bits over distance $\:d$. The input parameter is $\:{E}_{comm}$, the energy consumed per bit for transmission/reception; it is set to 50 nJ/bit. $\:{\eta\:}_{fs}$ represents the energy loss per bit per square meter in free space, and it is set to 0.0015pJ/bit. The second term, $\:{\eta\:}_{mp}$, accounts for the multipath fading loss per bit per meter to the fourth power when d exceeds $\:{d}_{0}$ which is defined as follows:

31 $$\:{d}_{0}=\sqrt{\frac{{\eta\:}_{fs}}{{\eta\:}_{mp}}}$$

- Energy Consumption for Data Reception:

32 $$\:{E}_{\mathrm{R}\mathrm{X}}\left(p\right)={E}_{\mathrm{c}\mathrm{o}\mathrm{m}\mathrm{m}}\times\:p$$

where $\:{E}_{RX}\left(p\right)\:$ is the energy used to receive a message containing $\:p$ bits.

- Energy Consumption for Data Aggregation:

33 $$\:{E}_{\mathrm{D}\mathrm{A}}\left(p\right)={E}_{\mathrm{a}\mathrm{g}\mathrm{g}}\times\:p$$

where $\:{E}_{DA}\left(p\right)$ is the energy consumed to aggregate $\:p$ bits of data, and $\:{E}_{agg}$ is the energy dissipated per bit for data aggregation, set to 10 nJ/bit.

### Cluster head selection in AEHMO

**Fig. 12:**
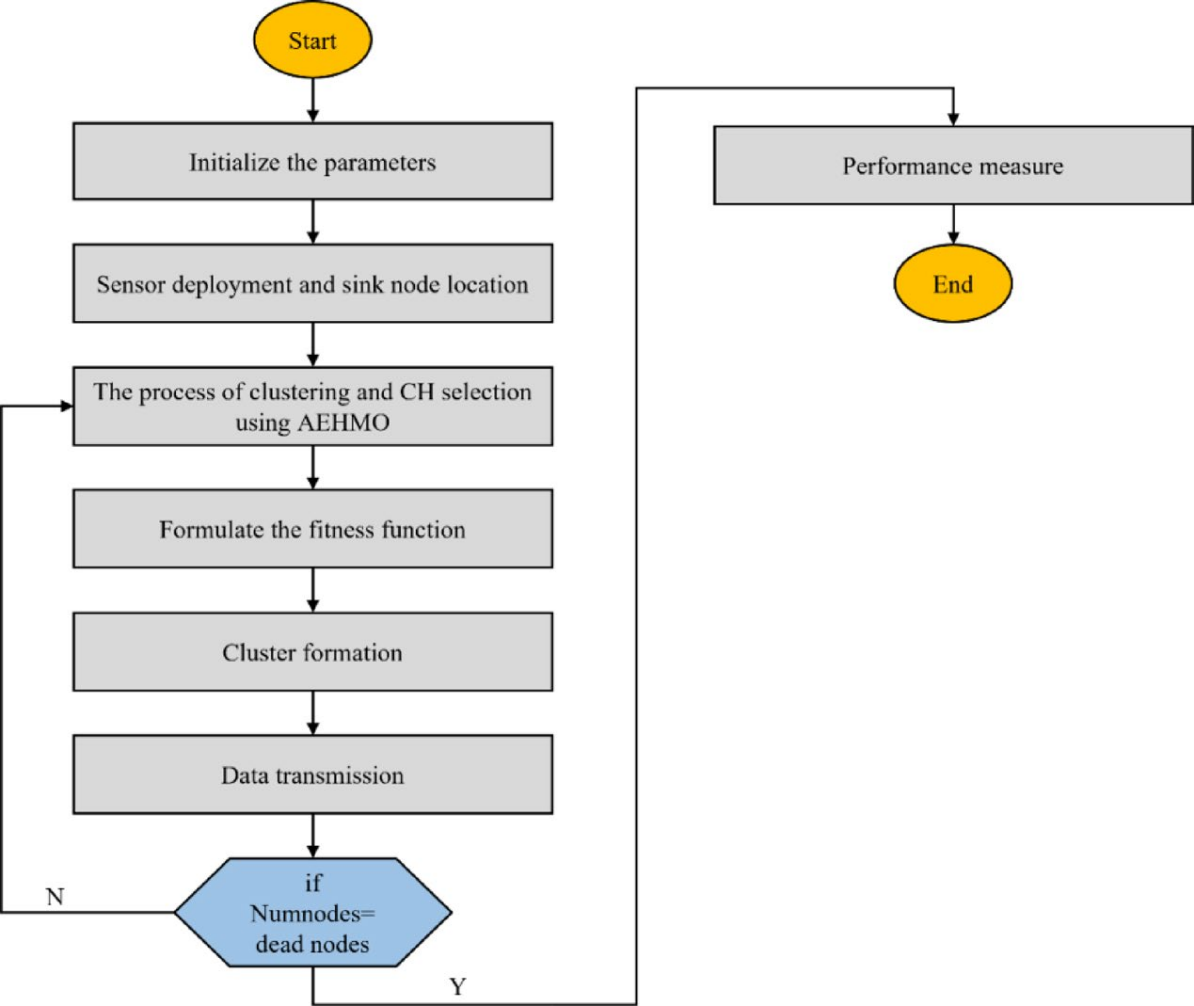
The process of CH selection using AEHMO.

In the proposed scheme for WSNs, the novel AEHMO algorithm is applied for selecting the optimal CHs to enhance communication efficiency between sensor nodes and base stations. The primary objective of this method is to optimize energy management in the routing process. AEHMO selects CHs through an optimization process that accounts for energy consumption, distance to the BS, node-to-CH distance, node degree, and node centrality. The selection of CHs using AEHMO is illustrated in Fig. 12.

The process determines the optimal number of CHs, where the candidate solution size ranges from 1 to the total number of sensor nodes. The AEHMO algorithm optimizes this selection by addressing an objective function, which factors in the energy consumption, distance to BS, packet delivery ratio (PDR), and throughput.

### Objective function for AEHMO-based CH selection

The fitness function employed in AEHMO serves as the core mechanism for selecting optimal CHs from the set of sensor nodes. To maintain consistency across homogeneous and heterogeneous WSN scenarios, the objective function uses the same normalized fitness components and aggregation structure throughout. In homogeneous deployments, all nodes share identical capabilities, so the heterogeneity-aware terms become constant and do not affect the optimization. In heterogeneous deployments, these terms become active while the underlying framework remains unchanged.

Let $\:S=\left\{{s}_{1},{s}_{2},\dots\:,{s}_{m}\right\}$ denote a candidate CH configuration, where $\:m$ represents the number of selected CHs, and let $\:{C}_{j}$ be the set of sensor nodes assigned to $\:\mathrm{C}\mathrm{H}{s}_{j}$. The fitness evaluation is performed over the candidate CH set and the induced cluster assignments rather than globally over all nodes. Four fitness parameters are considered: residual energy suitability, CH-to-base-station proximity, intra-cluster compactness, and a capability-aware bias term.

- The first fitness parameter, $\:F{P}_{1}$, represents the residual energy suitability of the selected CHs. Since CHs perform energy-intensive operations including data collection, aggregation, and forwarding, nodes with higher residual energy are prioritized for CH selection. To ensure applicability to both homogeneous and heterogeneous networks, residual energy is expressed in normalized form as:

34 $$\:F{P}_{1}=\sum\:_{j=1}^{m}\:\frac{{E}_{R}\left({s}_{j}\right)}{{E}_{\mathrm{m}\mathrm{a}\mathrm{x}}\left({s}_{j}\right)}$$

where $\:{E}_{R}\left({s}_{j}\right)$ denotes the residual energy of the $\:j$-th CH and $\:{E}_{\mathrm{m}\mathrm{a}\mathrm{x}}\left({s}_{j}\right)$ denotes its initial energy capacity. In homogeneous networks, $\:{E}_{\mathrm{max\:}}$ is identical for all nodes, whereas in heterogeneous networks it varies across node tiers, allowing the same formulation to naturally capture capability differences.

- The second fitness parameter, $\:F{P}_{2}$, captures the communication cost between CHs and the BS. Longer transmission distances lead to higher energy dissipation according to the adopted radio energy model. This parameter is defined as:

35 $$\:F{P}_{2}=\sum\:_{j=1}^{m}\:\left(1-\frac{\mathrm{d}\mathrm{i}\mathrm{s}\mathrm{t}\left({s}_{j},BS\right)}{{D}_{F}}\right)$$

where $\:\mathrm{d}\mathrm{i}\mathrm{s}\mathrm{t}\left({s}_{j},BS\right)$ denotes the Euclidean distance between $\:\mathrm{C}\mathrm{H}{s}_{j}$ and the base station, and $\:{D}_{F}$ represents the maximum possible communication distance in the deployment area. This normalized form is applicable to both fixed and mobile sink scenarios.

- The third fitness parameter, $\:F{P}_{3}$, reflects intra-cluster compactness and accounts for the average distance between sensor nodes and their associated CH. Since intra-cluster communication occurs frequently, compact clusters significantly reduce energy consumption. This parameter is formulated as:

36 $$\:F{P}_{3}=\sum\:_{j=1}^{m}\:\left(1-\frac{1}{\left|{C}_{j}\right|}\sum\:_{{s}_{i}\in\:{C}_{j}}\:\:\frac{\mathrm{d}\mathrm{i}\mathrm{s}\mathrm{t}\left({s}_{i},{s}_{j}\right)}{{D}_{F}}\right)$$

where $\:\left|{C}_{j}\right|$ is the number of nodes assigned to $\:\mathrm{C}\mathrm{H}{s}_{j}$, and $\:\mathrm{d}\mathrm{i}\mathrm{s}\mathrm{t}\left({s}_{i},{s}_{j}\right)$ denotes the distance between sensor node $\:{s}_{i}$ and its corresponding CH. Higher values of $\:F{P}_{3}$ indicate denser and more energy-efficient clusters.

- The fourth fitness parameter, $\:F{P}_{4}$, represents a capability-aware bias term. In homogeneous networks, where all nodes have identical initial energy and capabilities, this term becomes constant and does not influence the optimization process. In heterogeneous deployments, however, it explicitly promotes the selection of higher-capability nodes as CH s to prevent inefficient configurations where low-capability nodes are selected despite the presence of more suitable alternatives. The bias term is computed for each candidate CH as:

37 $$\:F{P}_{4}=\sum\:_{j=1}^{m}\:\mathrm{B}\mathrm{i}\mathrm{a}\mathrm{s}\left({s}_{j}\right)$$

38 $$\:F=\frac{1}{\alpha\:F{P}_{1}+\beta\:F{P}_{2}+\gamma\:F{P}_{3}+F{P}_{4}}$$

where $\:\mathrm{B}\mathrm{i}\mathrm{a}\mathrm{s}\left({s}_{j}\right)$ is determined according to the capability level of node $\:{s}_{j}$.

The overall fitness function minimized by AEHMO is defined as:

where $\:\alpha\:,\beta\:$, and $\:\gamma\:$ are weighting coefficients satisfying $\:\alpha\:+\beta\:+\gamma\:=1$ and $\:\alpha\:,\beta\:,\gamma\:\in\:\left(\mathrm{0,1}\right)$. In homogeneous scenarios, the weights are set to $\:\alpha\:=0.40,\beta\:=0.25$, and $\:\gamma\:=0.35$, reflecting the dominant importance of residual energy and communication efficiency, while the bias term remains inactive. Lower values of $\:F$ correspond to superior CH configurations in terms of energy sustainability, communication cost, cluster compactness, and-when applicable-tier-aware node selection.

### Cluster formation with potential function

After AEHMO identifies the optimal CH set, the remaining sensor nodes are assigned to their respective CHs using an energy- and load-aware cluster formation mechanism. At this stage, cluster formation is treated as a localized decision process rather than a global optimization procedure to ensure scalability and reduce the control overhead. Each sensor node independently determines its most suitable cluster assignment using a potential function that is a combination of several factors that are directly related to intra-cluster communication cost and cluster sustainability. The potential of assigning sensor node $\:{s}_{i}$ to cluster head $\:C{H}_{j}$, denoted as $\:S{N}_{p}$, is defined as:

39 $$\:S{N}_{p}=\frac{{\lambda\:}_{1}\cdot\:{E}_{C{H}_{j}}+{\lambda\:}_{2}\cdot\:{E}_{{s}_{i}}}{{\lambda\:}_{3}\cdot\:d\left({s}_{i},C{H}_{j}\right)+{\lambda\:}_{4}\cdot\:{L}_{C{H}_{j}}}$$

where $\:{E}_{C{H}_{j}}$ represents the residual energy of the cluster head, $\:{E}_{{s}_{i}}$ is the residual energy of the sensor node, $\:d\left({s}_{i},C{H}_{j}\right)$ denotes the Euclidean distance between the sensor node and the CH, and $\:{L}_{C{H}_{j}}$ is the current load of the CH measured as the number of nodes already associated with it. The parameters $\:{\lambda\:}_{1},{\lambda\:}_{2},{\lambda\:}_{3},{\lambda\:}_{4}$ are normalized weighting coefficients satisfying $\:\sum\:_{k=1}^{4}\:{\lambda\:}_{k}=1$. The weights used in this work are $\:{\lambda\:}_{1}$=0.40, $\:{\lambda\:}_{2}$= 0.20, $\:{\lambda\:}_{3}$=0.30, and $\:{\lambda\:}_{4}$=0.10. These values are selected based on the relative significance of each factor to the network energy consumption, stability and lifetime, and are consistent with the optimization priorities used in AEHMO-based CH selection. The CH residual energy is assigned the highest weight because CHs execute energy-intensive activities, including continuous reception of data from member nodes, aggregation and transmission of data over a long distance to the base station. Routing disruption and energy holes caused by prematurely drained CHs significantly accelerate overall network degradation. Network stability and reduced re-clustering rates are therefore direct outcomes of prioritization of CH residual energy.

Intra-cluster communication occurs far more frequently than the CH-to-BS transmission, therefore, node-to-CH distance is assigned the second highest weight. Reducing the distance between sensor nodes and their CHs significantly lowers transmission energy consumption with the first-order radio model, and per-round energy efficiency. Emphasizing this factor promotes spatially compact clusters and this improves the coverage quality and reduces the likelihood of the packet loss. The rest of the energy of the sensor node itself is assigned a moderate weight to prevent low-energy nodes from being assigned to distant or congested CHs, which could cause premature node failure. This term improves fairness in energy dissipation across the network without interfering with the membership of the cluster. The CH load is given the minimal weight since it is not a major energy driver, it is rather a correcting factor. Even when excessive CH load can increase the cost of aggregation and reception, its impact is secondary to the distance of communication and residual energy. This factor is allowed to have a relatively low weight to avoid frequent cluster reshuffling caused by minor load variations, without falling into the extreme imbalance in cluster sizes.

Each sensor node computes the potential value of each of the available CHs and selects the CH that maximizes $\:S{N}_{p}$. When two CHs yield identical potential values, the node is assigned to the one with greater residual energy, thereby further extending cluster lifetime. By integrating energy awareness, communication efficiency, and load balancing in a single lightweight association rule, the proposed cluster formation mechanism can be seen as an enhancement of the global optimization that is adopted by AEHMO, while maintaining low computational complexity and adaptability to dynamic WSN environments.

### Simulation settings and performance metrics

This section presents the experimental setup and the performance analysis of the proposed AEHMO-based CH selection approach. Various performance metrics are used to evaluate the cluster-based routing approach, including alive nodes, average energy consumption, total packets transmitted to the BS, FND, HND, and LND. A description of each metric is provided below:

- **FND (First Node Death)**: FND refers to the number of rounds that elapse before the first node in the network dies. It indicates how long all nodes in the network are *fully* functional.
- **HND (Half Node Death)**: HND represents the number of rounds at which 50% of the nodes in the network have died. Even after half of the nodes are no longer operational, the network continues to perform data transmission.
- **LND (Last Node Death)**: LND measures the number of rounds that pass before all nodes in the network are dead, signifying the point at which the network becomes entirely inoperative.
- **Alive Nodes**: This metric tracks the number of nodes that remain alive during network operation. A higher number of alive nodes generally correlates with better network performance.
- **Average Energy Consumption**: This refers to the average energy used by each node at the termination of each round in the case of simulation. Lower energy consumption reflects more efficient use of the limited power budget, directly contributing to extended network lifetime.
- **Total Packets Transmitted to the BS**: The total number of packets transmitted to the BS is directly related to the number of alive nodes and their remaining energy. A higher number of alive nodes typically leads to a higher number of packets reaching the BS.

In this work, several different network scenarios are simulated by varying the number of sensor nodes in the WSN: scenario 1 with 100 nodes, and scenario 2 with 150 nodes. This enables evaluation of AEHMO’s performance across networks of varying sizes. The simulation parameters are listed in Table 10.

**Table 10:** The experiment design parameters of the proposed AEHMO-based CH model in WSN.

Parameter	Value
Amplifier for large distances	0.0013 × $\:{10}^{-12}$
Energy for receiving $\:\left({E}_{RX}\right)$	50 × $\:{10}^{-9}$
Transmission energy ($\:{E}_{TX}$)	50 × $\:{10}^{-9}$
Field Size	100 m × 100 m
Transmission for free-space model	10 × $\:{10}^{-12}$
Initial Energy	0.5 J
Probability of CH election	0.1
Total number of iterations	4000
Sensor quantity	[100, 150]
Packet dimensions	2000 bits
Control packet size	80 bits
Population count	10
Basic routing method	LEACH
Network communication range	80 m
Wireless standard	Phy/wireless
Number of iterations	10
Idle node power	0.05 W

### Ablation study

An ablation study was conducted to quantify the individual contribution of each enhancement strategy to WSN performance, evaluating each strategy in isolation across 100-node and 150-node scenarios using FND, HND, LND, and average energy consumption as metrics. The full AEHMO configuration achieved the best results across all metrics, reaching FND of 752/1150 rounds and energy consumption of 0.381/0.395 J for 100/150 nodes respectively, representing improvements of 56.3%/38.1% in FND and 7.5%/6.8% in energy reduction over the baseline HMO. The 10.9%/7.4% FND improvement of full AEHMO over the best single-strategy variant confirms clear synergistic interaction among the four strategies. Full ablation results for all variants and both network scenarios are provided in Supplementary Appendix G, Table G1.

### Parameter sensitivity analysis for WSN Application

Before evaluating the performance of AEHMO in WSN cluster head selection, a sensitivity analysis was conducted to identify the most effective parameter configuration for the 150-node scenario. The analysis examined four key parameters: the chaotic iteration depth ($\:z$) in the CRL strategy, the MDMS mutation percentage, the MDMS scaling factor ($\:c$), and the fitness function weight combinations ($\:\alpha\:,\:\beta\:,\:\gamma\:$), using FND, HND, and LND as evaluation metrics. As shown in Table H1, the results confirmed $\:z$ = 20 as the optimal chaotic iteration depth, achieving the best FND of 1150 rounds, HND of 1853 rounds, and LND of 3136 rounds among all tested values. Table H2 presents the effect of varying the MDMS mutation percentage, where a value of 25% yielded the best overall performance, reflecting the need for more conservative elite refinement to preserve cluster formation stability in WSN environments. Table H3 summarizes the impact of the scaling factor ($\:c$), where $\:c$ = 0.7 achieved the highest network lifetime metrics. Table H4 presents the sensitivity analysis of five fitness function weight combinations, confirming that the configuration ($\:\alpha\:$=0.40, $\:\beta\:$=0.25, $\:\gamma\:$=0.35) strictly satisfying the physically grounded hierarchy $\:\alpha\:\:>\:\gamma\:\:>\:\beta\:$ achieves the best performance with FND of 840 rounds, HND of 1509 rounds, and LND of 2997 rounds, while progressive violations of this ordering produce monotonically decreasing network lifetime. Across all four parameters, AEHMO demonstrated stable and robust behavior over wide parameter ranges. Full sensitivity results and discussion are provided in Supplementary Appendix H, Tables H1, H2, H3, and H4.

### Alive nodes results analysis

We compare the proposed AEHMO algorithm with several state-of-the-art algorithms such as AOA, IVY, MGTOA, HMO, HEOA, GWO, HOA, MHCSA, AOA-H HO, MHABC-PSO and ASG-HMO in two different node density scenarios i.e., 100 and 150 nodes. The location of the BS is fixed at (50, 50) and performance is evaluated over up to 4000 rounds for each algorithm to evaluate each algorithm’s ability to maintain node survivability through efficient energy management.

**Fig. 13:**
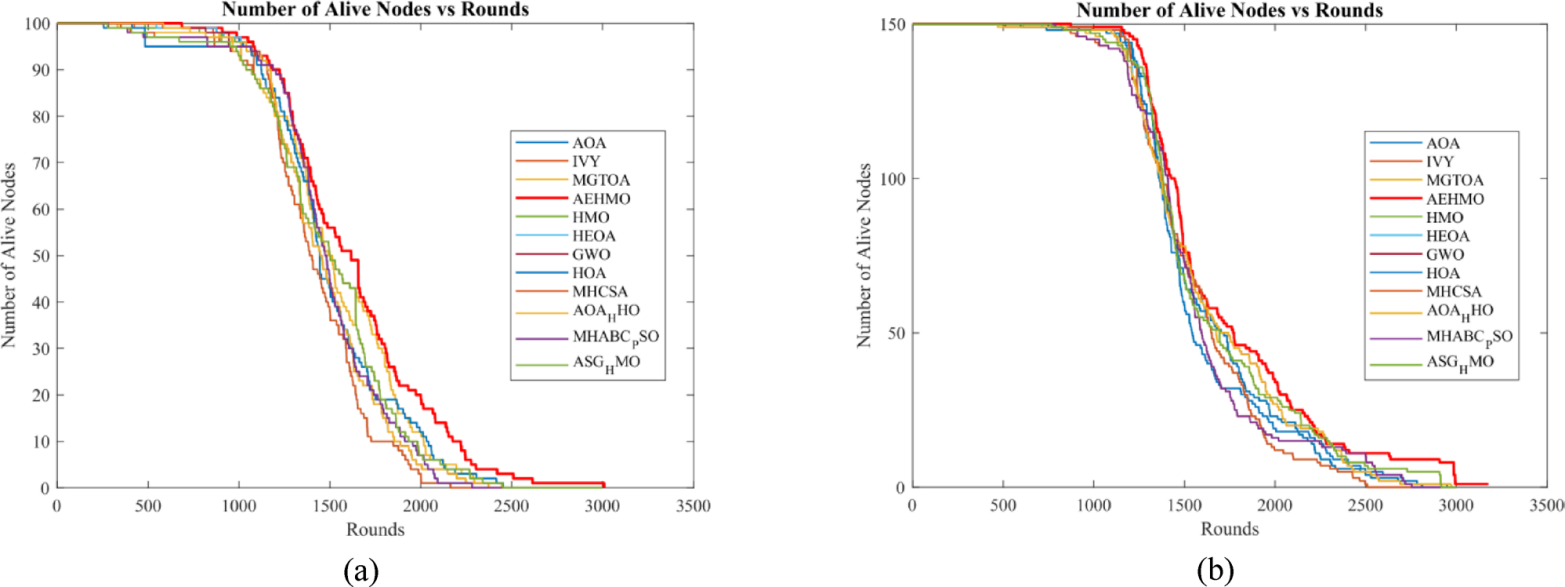
Alive nodes within different scenarios.

In the case of the 100-node scenario shown in Fig. 13(a), AEHMO shows superior performance as it keeps the largest number of alive nodes during the simulation period. AEHMO keeps all 100 nodes alive over 800 rounds which is much better than its competitors (like IVY, MGTOA, HMO) which begin to experience node failures around 600–700 rounds. The red curve representing AEHMO shows a significantly slower decline than other algorithms. This increased survivability can be attributed to AEHMO’s adaptive CH selection mechanism which efficiently distributes the energy consumption within the network by dynamically choosing CHs based on residual energy, distance between nodes and CHs, distance between CHs and the BS and degree of nodes. The combination of MDMS and DDS strategies enables AEHMO to avoid premature depletion of energy in individual nodes, which can lead to premature fragmentation of the network and shorten the operational lifetime of the network.

In the 150-node scenario shown in Fig. 13(b), AEHMO demonstrates strong scalability, maintaining efficient energy management even in dense network configurations. The algorithm keeps all 150 nodes alive for more than 1000 rounds, whereas competing algorithms such as AOA, MGTOA and GWO start having nodes dying much earlier, around 800–900 rounds. More impressively, AEHMO’s performance curve demonstrates an impressive longevity with nodes surviving up to the full 3200 rounds that the simulation lasts, significantly outlasting competing algorithms that experience complete network failure before 2500 rounds. This improved performance in larger networks demonstrates AEHMO’s scalability, and the successful use of its four enhancement strategies, working in synergy. The CRL strategy avoids convergence to suboptimal CH configurations while adaptive parameters $\:{w}_{1}$ and $\:{w}_{2}$ ensure the balanced exploration–exploitation transitions that adapt to increasing network complexity. The balanced energy consumption mechanism in AEHMO prevents the formation of hotspots and overload of individual nodes, which is a typical failure mode in algorithms such as GWO and HEOA which do not have adaptive load balancing. These results strongly demonstrate that AEHMO provides a robust and highly scalable solution to maximize the network lifetime for different network densities in which nodes in the 150-node case were kept in operation during the entire simulation time.

### Total packets transmitted to the BS result analysis

**Fig. 14:**
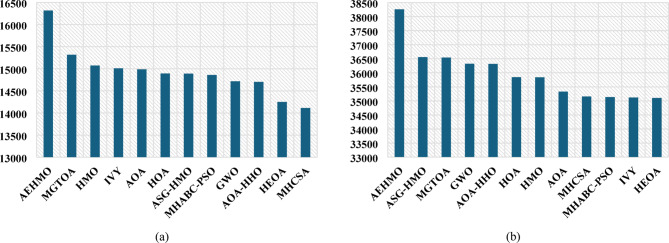
Total number of packets transmitted within different scenarios.

In order to measure the performance of AEHMO and other algorithms in terms of total number of packets sent to BS, two different scenarios with different number of nodes (100 and 150 nodes) were considered. The results for total packets sent to the BS show the efficiency of each algorithm to keep the communication of the network at a sustained level over the whole simulation period. Figure 14 gives a complete comparison of AEHMO with other state-of-the-art algorithms including ASG-HMO, MGTOA, GWO, AOA-HHO, HOA, HMO, AOA, MHCSA, MHABC-PSO, IVY and HEOA.

In the case with 100 nodes shown in Fig. 14(a), AEHMO has the best packet delivery performance with 16,316 successfully transmitted packets to the BS, which is about 6.5% better than the second-best algorithm MGTOA with 15,317 successfully transmitted packets. This substantial difference demonstrates the superior capability of AEHMO for sustained node operation and reliable communication based on its adaptive CH selection mechanism based on MDMS and DDS strategies. In the 150-node scenario in Fig. 14(b), AEHMO is very scalable, and achieves 38,257 packets delivered to the BS, an improvement of 4.7% over ASG-HMO (36557 packets) and 4.69% over MGTOA (36543 packets). The gap in performance is much higher if compared with lower performing algorithms like HEOA (35104 packets) and IVY (35122 packets) with AEHMO having about 9% more packets delivered. This superior performance in larger networks is mainly attributed to the CRL strategy of AEHMO, which introduces structured randomness to escape suboptimal CH configurations, and adaptive parameters $\:{w}_{1}$ and $\:{w}_{2}$, which dynamically change the exploration-exploitation balance during the optimization process. Unlike algorithms such as GWO (36320 packets), AOA-HHO (36315 packets), HMO (35837 packets) that use static parameter configurations, the adaptive mechanism of AEHMO helps maintain efficient routing paths throughout the network lifetime and prevents the occurrence of energy hotspots and premature network partitioning. These results clearly indicate that AEHMO extends the network lifetime and maximizes the data communication efficiency on different scales of the network, and hence is the most suitable solution for the applications that require reliable and long-term data transmission to the base station.

### Network lifetime result analysis

This section discusses the network lifetime of the proposed AEHMO algorithm, which is compared against other state-of-the-art algorithms using three critical metrics: FND, HND and LND. These metrics were tested in two different scenarios with 100 and 150 node densities to fully measure the capabilities of the algorithms in prolonging the operational lifetime of the network by means of effective energy management strategies.

**Fig. 15:**
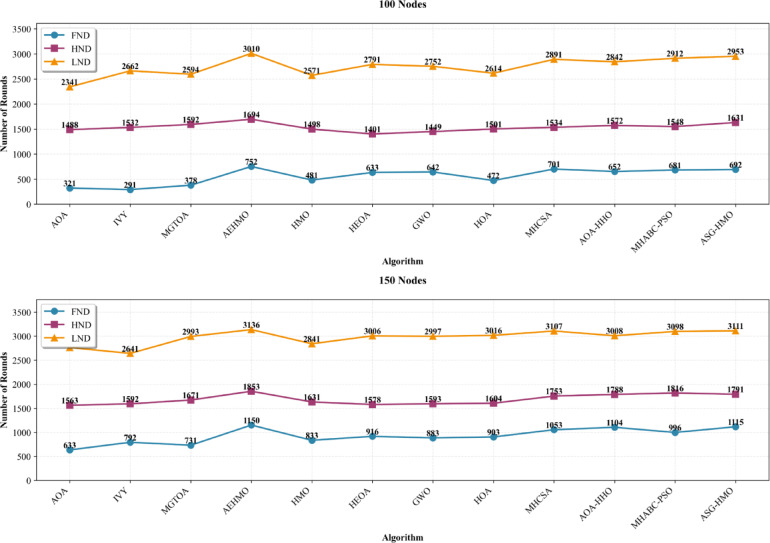
FND results within different scenarios.

In the case of 100 nodes shown in Fig. 15, AEHMO achieves strong performance with FND of 752 rounds, HND of 1694 rounds and LND of 3010 rounds, which are much better than all its competitors. For FND, AEHMO, as compared to the second-best algorithm MHCSA (701 rounds), is able to delay the first node death and more than double that of weaker algorithms like IVY (291 rounds) and AOA (321 rounds). This substantial delay in FND is attributed to the DDS and MDMS strategies, which allow AEHMO to investigate the optimal choice of cluster heads, and to evenly distribute energy consumption throughout the network, which prevents any premature energy depletion of any node in the network. For HND, AEHMO’s 1694 rounds is greater than ASG-HMO (1631 rounds) and is far greater than algorithms like HEOA (1401 rounds) and GWO (1449 rounds), indicating sustained network stability. The adaptive parameters $\:{w}_{1}$ and $\:{w}_{2}$ play an important role in this phase as they dynamically modulate the exploration-exploitation balance in order to maintain the efficient utilization of energy over time and a higher number of operating nodes for longer periods.

In the case of 150 nodes, the scalability and robustness of AEHMO is even more pronounced with FND at 1150 rounds, 9.2% more than ASG-HMO (1115 rounds) and 81.7% more than AOA (633 rounds). The HND metric achieves 1853 rounds which is better than MHABC-PSO (1816 rounds) and AOA-HHO (1788 rounds) in terms of network stability by keeping the CH selection balanced longer. Most notably, AEHMO achieves LND at 3136 rounds, the highest for any of the algorithms and 0.8% higher than ASG-HMO (3111 rounds) and far higher than MGTOA (2993 rounds) and IVY (2641 rounds). The CRL strategy plays a key role in this accomplishment because it introduces structured randomness through the use of chaotic mapping to avoid convergence to suboptimal CH configurations which may result in rapid depletion of energy or partitioning of the network. The consistent superiority of AEHMO in all three-lifetime metrics in both network densities validates the synergistic effect of AEHMO four enhancement strategies, namely adaptive parameters, MDMS, DDS and CRL, which together ensure balanced energy distribution and prolonged network lifetime, making AEHMO well-suited for enhancing WSN sustainability.

### Average energy consumption results analysis

This section presents the average energy consumption of AEHMO across various network scenarios, compared against state-of-the-art algorithms. Energy consumption is a critical metric for evaluating algorithmic efficiency of managing the available energy resources of the nodes within the network. Lower energy consumption directly translates to longer operational lifetime of the nodes, and longer network sustainability.

**Fig. 16:**
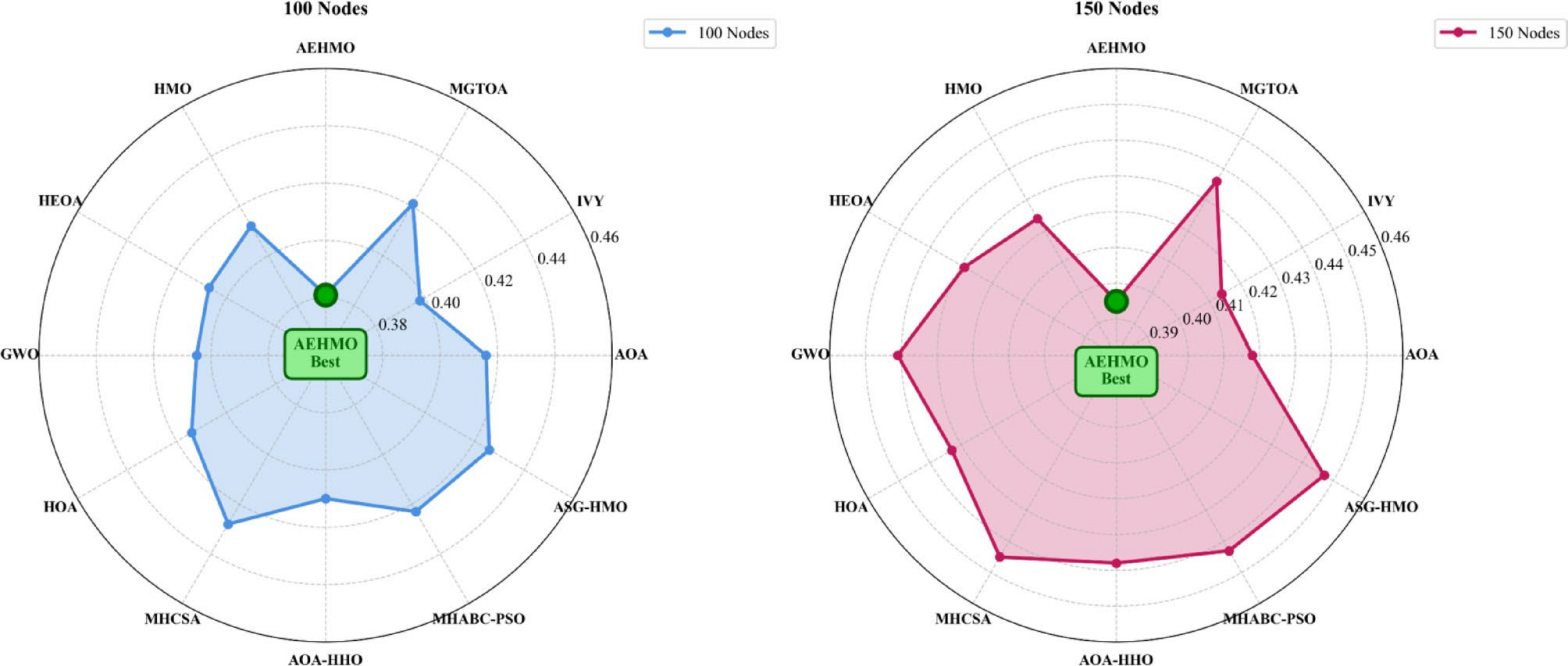
Average energy consumption within scenario 1.

In the case of 100 nodes as shown in Fig. 16(a), AEHMO achieves the lowest average energy consumption at 0.381 J compared to all the other competing algorithms, and hence it is the most energy efficient algorithm. AEHMO is 4.3% lower energy consumption than the 2nd best algorithm IVY (0.398 J) and 5.9% lower energy consumption than GWO (0.405 J). The performance gap is more significant when compared to less efficient algorithms like MHCSA (0.428 J), ASG-HMO (0.426 J) and MHABC-PSO (0.423 J) where AEHMO achieves an energy consumption reduction of about 11.0%, 10.6% and 9.9% respectively. Even against the baseline HMO (0.412 J) AEHMO has shown a 7.5% improvement in energy efficiency. The DDS strategy further aids by enabling extensive searches of the solution space in early iterations that would otherwise cause the algorithm to converge to energy inefficient CH configurations that could lead to the formation of hotspots and rapid depletion of energy.

In the 150-node case shown in Fig. 16(b), AEHMO maintains its leadership in terms of the lowest average energy consumption at 0.395 J, which indicates that it has an excellent scalability as the network density increases. AEHMO achieves 4.6% less energy consumption when compared with IVY (0.414 J) and 5.5% less energy consumption when compared with AOA (0.418 J) and this difference in performance increases to 11.6% and 11.2% when compared with ASG-HMO (0.447 J) and MHCSA (0.445 J), respectively. It is worth noting that AEHMO consumes 6.8% less energy than the baseline HMO (0.424 J), for this larger network configuration. This superiority in the larger networks over a long time validates the importance of the adaptive parameters $\:{w}_{1}$ and $\:{w}_{2}$ used by AEHMO, which dynamically adjust the balance between exploration and exploitation throughout the optimization process, ensuring efficient energy utilization even as the complexity of networks is increasing. The CRL strategy plays an important role by introducing structured randomness through chaotic mapping, AEHMO can break out of suboptimal CH arrangements that can lead to uneven energy distribution and premature node failures, a common drawback of static algorithms such as HMO and HEOA (0.429 J). The sustained achievement of the lowest energy consumption across both network densities confirms that AEHMO’s synergistic combination of four enhancement strategies is able to offer the most energy efficient solution for the problem of WSN cluster head selection.

### Result analysis for AEHMO using BS at the corner

To further evaluate the robustness of AEHMO under asymmetric network topology, an additional experiment was conducted with the BS placed at the corner of the sensing field at coordinates (100, 100) using a 100-node deployment. Under this more demanding scenario, AEHMO achieved the best performance across all compared algorithms with FND = 785, HND = 1420, and LND = 2815 rounds, outperforming the closest competitor HMO by 5.8%, 5.3%, and 4.7% in FND, HND, and LND respectively. Full results and comparative analysis are provided in Supplementary Appendix I, Table I1.

### Real world heterogeneous WSN scenario validation using mobile sink strategy

To address the limitations of the scalability problems and to validate the performance of AEHMO under the real-world deployment condition, this subsection presents a comprehensive evaluation of AEHMO using a large-scale heterogeneous network scenario with 1200 sensor nodes. In contrast to the homogeneous scenarios which are simplified in previous subsections, this evaluation incorporates multi-tier heterogeneity, strategic spatial deployment, a mobile sink strategy, and complex network topologies to accurately reflect real-world WSN deployments in industrial, environmental and smart city applications.

#### Three tier heterogeneous network model

The heterogeneous network model generalizes the basic WSN model by adding three different levels of node capability that produce a realistic model of practical sensor deployments in which nodes have different levels of computational power, energy reserve and transmission capability. This three-tier heterogeneity (3-Tier) model consists of advanced, intermediate, and normal nodes with initial energy levels and deployment strategies.

The network with 1200 nodes is divided into three hierarchical categories based on some established principles in designing heterogeneous WSN. Let $\:n$ = 1200 be the total number of deployed sensor nodes that are spread in the monitoring field. The inequality of the distribution is according to the inequality:

40 $$\:{n}_{nrm}\:>\:{n}_{int}\:>\:{n}_{adv}$$

This inequality ensures that normal nodes form the majority, providing full area coverage; intermediate nodes offer supplementary support; and advanced nodes constitute a smaller but critical subset with enhanced capabilities. For the case of 3-Tier configuration, the network composition is specified with help of proportion factors $\:k$ and $\:{k}_{o}$:

41 $$\:{n}_{adv}\:=\:n\:\times\:\:k$$

42 $$\:{n}_{int}\:=\:n\:\times\:\:{k}_{o}$$

43 $$\:{n}_{nrm}\:=\:n\:\times\:\:(1\:-\:k\:-\:{k}_{o})$$

where $\:k$ is the proportion of advanced nodes, and $\:{k}_{o}$ is the proportion of intermediate nodes in the network. For the 1200-node deployment, $\:k$ is set to 0.167 and $\:{k}_{o}$ is set to 0.25 are used resulting in:

• Advanced nodes ($\:{n}_{adv}$) = 200 nodes (16.7%).

• Intermediate nodes ($\:{n}_{int}$) = 300 nodes (25%).

• Normal nodes ($\:{n}_{nrm}$) = 700 nodes (58.3%).

#### Energy configuration of heterogeneous tier

The allocation of energy is hierarchically organized according to the following basic inequality:

44 $$\:{E}_{nrm}\:<\:{E}_{int}\:<\:{E}_{adv}$$

This inequality reflects the realistic case where more able nodes have access to higher capacity power sources to support their extended role of CH and data aggregation point. The energy of each tier is determined as follows:

45 $$\:{E}_{nrm}\:=\:{E}_{o}\:\times\:\:{n}_{nrm}$$

46 $$\:{E}_{int}\:=\:{E}_{o}\:\times\:\:(1\:+\:\varPsi\:)\:\times\:\:{n}_{int}$$

47 $$\:{E}_{adv}\:=\:{E}_{o}\:\times\:\:(1\:+\:\omega\:)\:\times\:\:{n}_{adv}$$

where $\:{E}_{o}$ is the base energy assigned to normal nodes and $\:\varPsi\:$ is the energy fraction by which intermediate nodes exceed normal nodes and $\:\omega\:$ is the energy fraction by which advanced nodes exceed normal nodes. For the above deployment, $\:{E}_{o}$ = 0.5 J, $\:\varPsi\:$ = 1.0 (intermediate nodes have 100% more energy as compared to normal nodes) and $\:\omega\:$ = 2.0 (advanced nodes have 200% more energy as compared to normal nodes). This arrangement gives a value of 0.5 J for individual node energies for normal nodes, 1.0 J for intermediate nodes, and 1.5 J for advanced nodes. The total of the network energy is computed using:.

48 $$\:{E}_{T}\:=\:{E}_{adv}\:+\:{E}_{int}\:+\:{E}_{nrm}$$

which can be simplified to:

49 $$\:{E}_{T}\:=\:{E}_{o}\:\times\:\:n\:\times\:\:(1\:+\:\varPsi\:\:\times\:\:{k}_{o}\:+\:k\:\times\:\:\omega\:)$$

This hierarchical distribution of energy ensures that sophisticated nodes can be used for sustaining the CH tasks for much longer durations, intermediate nodes can act as important backups and supplementary CH services, and normal nodes are mostly sensing and data collection units with less processing overheads.

#### Spatial deployment topology

Unlike the homogeneous random deployment used in the previous section, this heterogeneous network uses a spatial distribution strategy that considers realistic deployment constraint, network optimization principles and practical infrastructure considerations in real-world WSN applications. The monitoring area is set up as a 300$\:\times\:$300 square area which represents a realistic coverage area for industrial monitoring, precision agriculture or environmental sensing applications. The position of BS is central coordinates:

50 $$\:({c}_{x},\:{c}_{y})$$

This central positioning ensures the maximum transmission distance from any sensor node towards the BS is minimum and hence the energy consumption is less, and network connectivity is improved. However, in order to resolve the hotspot problem of the centralized BS architectures, moveable multiple sinks schemes are integrated, which is described in next subsection.

The deployment is based on a hierarchical zonal strategy with the aim to maximize the energy efficiency by locating more capable nodes close to the critical infrastructure and ensuring the whole area is covered through the distributed normal nodes. Advanced nodes are located in a strategic manner in a circular subsection with the BS location (150, 150) as the center. The radius of such circular zone is calculated in accordance with network density requirements and optimum CH distribution with $\:{r}_{adv}$ is 40 m. This central location ensures that the most able nodes with the most energy reserves are located near the BS, reducing the distances over which long distance transmissions occur when inter-cluster communication is necessary, and ensuring that they will have the greatest utility as primary cluster heads throughout the operational lifetime of the network. The density of advanced nodes in circular subsection is given as:

51 $$\:Do{N}_{Adv}\:=\frac{Number\:of\:advanced\:nodes}{Circular\:subsection\:area}$$

52 $$\:Do{N}_{Adv}\:=\:{n}_{adv}/(\pi\:\:\times\:\:{r}_{adv}^{2})$$

Intermediate nodes are deployed strategically all over the sensor field area except the circular subsection which is reserved for advanced nodes. A critical constraint controls their placement in such a way that intermediate nodes have to be deployed in the vicinity of randomly selected normal nodes so that each normal node has access to at least one higher-capacity neighbor in order to provide CH services. The deployment approach makes sure that no normal node is assigned to more than two intermediate nodes which will prevent clustering imbalances and ensure energy is distributed equally. The density of the intermediate nodes is as follows:

53 $$\:Do{N}_{Int}=\frac{Number\:of\:intermediate\:nodes}{Sensor\:field\:area\:-\:Circular\:subsection\:area}$$

54 $$\:Do{N}_{Int}\:=\:{n}_{int}/(Fiel{d}_{area}\:-\:\pi\:\:\times\:\:{r}_{adv}^{2})$$

For the deployment scenario, where $\:Fiel{d}_{area}$ is 300$\:\times\:$300m^2^. Normal nodes are randomly distributed all over the 300$\:\times\:$300 monitoring area (not the central circular subsection which will be used only for advanced nodes). This distribution gives high coverage of the areas with still preserving the hierarchy energy structure where normal nodes are known as the primary sensing layer that is responsible for forwards the data to their designated cluster heads. The normal nodes density is as follows:

55 $$\:Do{N}_{Nrm}=\frac{Number\:of\:Normal\:nodes}{Sensor\:field\:area\:-\:Circular\:subsection\:area}$$

56 $$\:Do{N}_{Nrm}\:=\:{n}_{nrm}/(Fiel{d}_{area}\:-\:\pi\:\:\times\:\:{r}_{adv}^{2})$$

This spatial arrangement generates realistic topology, which emulates the real-life deployments of having high-capacity nodes near the critical infrastructures (base stations, gateways, edge servers) while providing wide pervasive coverage with distributed sensing nodes. The zonal deployment strategy naturally sets up energy-efficient communication patterns because the high-capacity advanced nodes that are close to the BS can aggregate and relay data from several clusters without consuming their energy reserves too early.

#### Movable multiple sink strategy

One of the important problems in WSN with centralized BS architecture is the hotspot problem where the CHs near the BS get drained out of energy rapidly due to intensive relay traffic from the far clusters. To overcome this simple limitation and to enhance the network performance, movable multiple sink strategy is proposed in the deployment scenario.

In static single sink configurations, energy consumption of sensor nodes which are nearest to the BS are too high since they have to relay data from the more distant nodes, leading to premature death of nodes and network partitioning. Movable sinks in the network strategically positioned outside the network field can enhance the performance of the network in terms of reducing communication distances between the CHs and the sinks, balancing the relay load on the network, reducing congestion around the static base stations, extending the lifetime of the network by balancing the energy consumption and efficiency of data collection in large-scale deployments.

In this deployment scenario, there are four movable sinks outside of the network field, and circular trajectories. The sinks are moving on their own without any human intervention. The sinks traverse outside the network area in circular patterns. The middle of this virtual circle is the point of the center of the network $\:Center\:=\:({c}_{x},\:{c}_{y})$. The radius of the circular trajectory is taken such that the sinks are not placed on the boundaries of the network, and the distances of communication are reasonable:

57 $$r_{c} \:> \:\sqrt {((Field_{{width}} /2)^{2} \: + \:(Field_{{height}} /2)^{2} )}$$

For the 300$\:\times\:$300 field, $\:{r}_{c}$ is made large enough to place sinks outside of the network area. The sink locations at any instant of time are generated by means of parametric equations:

58 $$\:x\:=\:{r}_{c}\:\times\:\:cos\left(\theta\:\right)\:+\:{c}_{x}$$

59 $$\:y\:=\:{r}_{c}\:\times\:\:sin\left(\theta\:\right)\:+\:{c}_{y}$$

where $\:\theta\:$ is the angular position parameter and depending on the mobility requirements and constraints of the application different rotation strategies can be used. For complete circular rotation, the parameter, $\:\theta\:$, varies $\:0\le\:\theta\:\le\:2\pi\:,0\le\:\theta\:\le\:\pi\:$, and $\:0\le\:\theta\:\le\:\pi\:/2$, so the sinks can move all around the perimeter of the network. All sinks are free to move along their circular paths in either clockwise or counter-clockwise. As the network operation continues for several rounds, the sinks move to their next position as per a predefined movement schedule and collect the data from different regions of the network in turn. In the mobile sink case, it is assumed that sensor nodes and CHs know the location of sinks by periodic beacon messages transmitted by each sink at the start of each round, which is consistent with the round-based operation of LEACH like protocols. Each beacon contains the identifier of the sink and the actual coordinates in the predefined trajectory for the CHs to estimate the distances to the beacons based on RSSI or localization services and select the sink that is closest to be able to forward the data. Beaconing is performed once per round to balance location accuracy against control overhead. The additional energy costs added by sink mobility are explicitly considered in the energy model by receiving beacon packets and exchanging scarce control messages, so as to ensure that the benefits of mobility are not overestimated.

#### Extending AEHMO fitness function for heterogenous CH selection

In heterogeneous WSN deployments, the AEHMO fitness function defined in the section “Objective function for AEHMO-based CH selection is extended to explicitly account for node capability differences arising from the multi-tier network structure. The core fitness components-residual energy suitability ($\:F{P}_{1}$), CH -to-sink proximity ($\:F{P}_{2}$), and intra-cluster compactness ($\:F{P}_{3}$)-remain unchanged and are evaluated using the unified formulation.

To prevent inefficient CH configurations in which low-capability nodes are selected despite the presence of more capable alternatives, an additional capability-aware bias term $\:F{P}_{4}$ is activated. This term explicitly incorporates node-tier information into the CH selection process and is defined as:

60 $$\:F{P}_{4}=\sum\:_{j=1}^{m}\:\mathrm{B}\mathrm{i}\mathrm{a}\mathrm{s}\left({s}_{j}\right)$$

where $\:\mathrm{B}\mathrm{i}\mathrm{a}\mathrm{s}\left({s}_{j}\right)$ assigns higher values to nodes belonging to higher-capability tiers. In homogeneous deployments, where all nodes share identical initial energy and capability, this term reduces to a constant and does not influence the optimization outcome. The heuristic nature of metaheuristic optimization algorithms may result in suboptimal CH selection with no consideration of inherent capability difference between node tiers. For example, a normal node could be selected as CH in presence of an advanced node or an intermediate node in the same cluster, it could just happen that the optimization process has generated that configuration with good fitness based on $\:F{P}_{1}$, $\:F{P}_{2}$ and $\:F{P}_{3}$ alone. However, the selection of normal node as CH in case of advanced node is fundamentally inefficient as the normal node will get exhausted much faster so that more frequent re-clustering operations are required, and network stability is low. The capability-aware bias parameter addresses this limitation by explicitly favoring higher-tier nodes in the candidate CH set:

61 $$\:Bias\left({s}_{j}\right)=\left\{\begin{array}{c}1-\frac{{n}_{adv}}{n}\:\:\:\:\:\:\:\:\:\:\:\:\:\:\:\:\:\:\:\:\:\:\:\:\:\:\:if\:{s}_{j}\:is\:advanced\:node\\\:1-\frac{{n}_{int}}{n}\:\:\:\:\:\:\:\:\:\:\:\:\:\:\:\:\:\:\:\:\:\:if\:{s}_{j}\:is\:intermediate\:node\\\:1-\frac{{n}_{nrm}}{n}\:\:\:\:\:\:\:\:\:\:\:\:\:\:\:\:\:\:\:\:\:\:\:\:\:\:\:\:\:\:\:\:\:if\:{s}_{j}\:is\:normal\:node\end{array}\right.$$

For the 1200 node deployment with 200 advanced, 300 intermediate and 700 normal nodes, the bias values are 0.833 for advanced nodes, 0.750 for intermediate nodes and 0.417 for normal nodes. This parameter assigns higher bias values to advanced and intermediate nodes, encouraging the algorithm to leverage the energy advantage of capable nodes from the outset and not wait until energy differentiation becomes visible through $\:F{P}_{1}$ only. Thus, the four fitness parameters are aggregated in one objective function by weighted aggregation:

62 $$\:F\:=\:1\:/\:(\alpha\:\:\times\:\:F{P}_{1}\:+\:\beta\:\:\times\:\:F{P}_{2}\:+\:\gamma\:\:\times\:\:F{P}_{3}\:+\:F{P}_{4})\:$$

where the weight coefficients must satisfy the normalization condition that $\:\alpha\:\:+\:\beta\:\:+\:\gamma\:\:=\:1$. And each of the coefficients are bounded in such that $\:\alpha\:$, $\:\beta\:$, $\:\gamma\:$ are in 0 to 1. For this heterogeneous scenario, the weights are assigned in accordance with the relative importance of each factor in large-scale networks with mobile sinks. Specifically, the value for $\:\alpha\:$= 0.40 (residual energy weight having the highest priority in terms of sustainability), $\:\beta\:$= 0.25 (CH-sink distance weight being critical for movable sink scenarios) and $\:\gamma\:$= 0.35 (intra-cluster compactness weight being important for communication efficiency). These weights satisfy the constraint of being a vector that adds up to 1.00, i.e., satisfy the constraint of being properly normalized. During its optimization process, AEHMO minimizes the fitness function $\:F$, with lower values of $\:F$ corresponding to better CH configurations in terms of energy efficiency, communications distance, clusters compactness and tier-appropriate node selection.

#### Multi hop communication strategies

To further enhance the energy efficiency in this large-scale heterogeneous network, AEHMO integrates with the intra-cluster and inter-cluster multi-hop routing strategies. These strategies are based on the understanding that direct long-distance transmissions are energy inefficient compared to multi-hop relaying via the intermediate nodes, especially for large 300$\:\times\:$300 fields where node to sink or node to CH distances can easily exceed 200 m. Despite the addition of mobile sinks and multi-hop extensions, the underlying communication and clustering structure is still LEACH-based with AEHMO only replacing the CH selection mechanism while preserving the standard setup and steady-state phases, TDMA scheduling and data aggregation model:

- **Intra-cluster multi hop strategy**: within a cluster, the energy consumption for communication between the cluster members (CMs) and the CH is proportional to the square of the distance for the communications below the threshold $\:{r}_{o}$ (free-space propagation). Since, only a small fraction of nodes in a cluster are physically close to center of the cluster, many CHs elected through rotation strategies can be far from the geometric center of the cluster, this leads to increasing intra-cluster communication distances, and aggravates energy consumption for CMs. To solve this inefficiency, intra-cluster multi-hop strategy is taken. A threshold distance $\:{d}_{intra}$ is specified in order to classify the members of the clusters:
  - **Inner layer nodes**: CMs whose distance from the CH is $\:\le\:\:{d}_{intra}$.
  - **Outer layer nodes**: CMs which are further than $\:{d}_{intra}$ from the CH.

The working principle of the communication protocol is as follows

 Inner layer nodes communicate directly with CH. Outer layer nodes select the nearest inner layer node to which they send their data to CH. For an outer layer node $\:{S}_{out}$ sending $\:k$ bits of data to the CH through an inner layer node $\:{S}_{in}$ where $\:{d}_{{S}_{out}-\:{S}_{in}}$ is the distance between the two nodes and no greater than $\:{r}_{o}$, the total energy consumption is.

63 $$\begin{aligned} _{{2\_{\mathrm{hop}}\:}} = & E_{T} \left( {\underbrace {{k,d_{{s_{{{\mathrm{out}}\_{\kern 1pt} {\mathrm{s}}_{{{\mathrm{in}}{\kern 1pt} }} }} }} }}_{{s_{{{\mathrm{out}}{\kern 1pt} }} }}} \right) + \underbrace {{E_{R} \left( k \right) + E_{D} \left( k \right)}}_{{s_{{{\mathrm{in}}{\kern 1pt} }} }} \\ = & kE_{{{\mathrm{elec}}\:}} + k_{{\varepsilon _{{fs}} }} d_{{s_{{{\mathrm{out}}\_{\kern 1pt} {\mathrm{s}}_{{{\mathrm{in}}{\kern 1pt} }} }}^{2} }} + kE_{{{\mathrm{elec}}\:}} + kE_{{{\mathrm{da}}\:}} \\ = & k\left( {2E_{{{\mathrm{elec}}\:}} + E_{{{\mathrm{da}}\:}} } \right) + k_{{f_{f} }} d_{{s_{{{\mathrm{out}}\_{\kern 1pt} }} s_{{{\mathrm{in}}{\kern 1pt} }} }}^{2} \\ \end{aligned}$$

The magnitude of $\:{E}_{2-hop}$ is a function primarily of $\:{d}_{{S}_{out}-\:{S}_{in}}$. Therefore, for intra-cluster communication, it is energy-efficient that the outer layer nodes select the inner layer node that is closest to them as a relay. This minimizes $\:{d}_{{S}_{out}-\:{S}_{in}}$, and hence the quadratic energy term. The value of $\:{d}_{intra}$ which is decided by network environment and algorithm designing. Larger cluster structures have larger $\:{d}_{intra}$ value for balancing the tradeoff between overhead of relay and reduction of transmission distance. For 1200 node scenario in 300$\:\times\:$300 field $\:{d}_{intra}$ is calculated using empirical optimization.

- **Inter-cluster multi hop strategy**: In the inter-cluster communication phase CHs need to route their aggregated data to the BS. For CHs placed at a large distance from sinks (beyond the threshold $\:{d}_{inter}$), direct transmission would result in an excessive energy consumption, because of the $\:{d}^{4}$ relation from multipath propagation. Multi-hop forwarding through intermediate CHs is a more energy efficient solution. CHs select the most energy efficient relay node by measuring the distances to neighboring CHs by using received signal strength indicator (RSSI) measurement. Multi-hop forwarding is performed only if the distance between the CH and the BS (or nearest sink) is more than the inter-cluster threshold $\:{d}_{inter}$ otherwise the CH sends the data directly. Based on distance relationship between $\:C{H}_{i}$ and BS, and distance relationship between $\:C{H}_{i}$ and its neighboring CHs, routing metric $\:{R}_{t}$ for each of neighboring CHs is computed as follows:

64 $$\:Rt\left(i\right)=\frac{d(i,BS{)}^{2}}{d(i,j{)}^{2}+d(j,BS{)}^{2}},j=\mathrm{1,2},\dots\:,{N}_{CH}$$

where $\:Rt\left(i\right)$ is the array of routing metric of neighboring CHs of $\:C{H}_{i}$, $\:d(i,\:BS)$ is the distance between $\:C{H}_{i}$ and the base station (or nearest sink), $\:d(i,\:j)$ is the distance between $\:C{H}_{i}$ and neighboring $\:C{H}_{j}$, $\:d(j,\:BS)$ is the distance between neighboring $\:C{H}_{j}$ and the base station and $\:j$ is the index of the neighboring CH ranging from 1 to $\:{N}_{CH}$, where $\:{N}_{CH}$ is the total number of CHs. Based on calculated $\:Rt$ values, $\:C{H}_{i}$ selects the next hop node based on:

65 $$\:\mathrm{N}\mathrm{e}\mathrm{x}\mathrm{t}\left(i\right)=\left\{\begin{array}{l}j\:\:\:\:\:\:\:\:\:\:\:\mathrm{\:if\:}\:\left(\mathrm{m}\mathrm{a}\mathrm{x}\right(\mathrm{R}\mathrm{t}\left(i\right)>1)\:\mathrm{\:and\:}\\\:\:\:\:\:\:\:\:\:\:\:\:\:\:\:\:\:\:\:\:\:\:\:\:\:\:\:\:\:\:\mathrm{m}\mathrm{a}\mathrm{x}\left(\mathrm{R}\mathrm{t}\right(i\left)\right)=Rt(i,j))\\\:0\:\mathrm{\:\:\:\:\:\:\:\:\:\:\:\:\:\:\:\:if\:}\:\mathrm{m}\mathrm{a}\mathrm{x}\left(Rt\right(i\left)\right)\le\:1\end{array}\right.$$

where $\:Next\left(i\right)\:=\:j$ means that $\:C{H}_{j}$ is selected as the relay node and $\:Next\left(i\right)\:=\:0$ means direct transmission to the BS without any relaying. The condition $\:max\left(Rt\right(i\left)\right)>1$ ensures that relaying through $\:C{H}_{j}$ is geometrical favorable in comparison to direct transmission. When $\:Rt\:>\:1$, the square distance from $\:C{H}_{i}$ to the BS will be more than the sum of square distances via relay path so multi hop transmission will be more energy efficient.

Furthermore, the inter-cluster multi-hop strategy does not take the residual energy of relay CHs explicitly into consideration when the next hop is selected. This choice of design is justified by the strategy of CH rotation used in the network. In each round, CHs gather residual energy information from their CMs and compare their own residual energy to the initial cluster average residual energy ($\:{C}_{initial}$) to make sure that energy factors are well considered during CH selection. In the case of inter-cluster multi-hop routing, the relay node with the largest $\:Rt$ is able to guarantee the most energy-efficient relay scheme from a geometric point of view. If the residual energy was further considered at this point, the approach might choose geometrically suboptimal relay paths to conserve low energy nodes which might lead to a higher overall network energy consumption. Therefore, not considering the energy aspect in relay selection is helpful to minimize information exchange overhead between CHs, without compromising the energy efficiency. For the field size of 300 m $\:\times\:$ 300 m with movable sinks, the value of $\:{d}_{inter}$ is obtained according to the threshold of the propagation model and network dimensions. CHs outside this threshold use multi-hop routing and CHs inside this range communicate directly with the sinks.

#### Results analysis for the heterogeneous WSN scenario

This subsection presents a detailed analysis of the performance of AEHMO in the large-scale heterogeneous WSN scenario (1200 nodes) with a mobile-sink strategy. The heterogeneous three-tier architecture and mobile sinks allow for a realistic testing environment to closely approximate practical WSN deployments for industrial monitoring, smart cities and environmental sensing applications.

**Fig. 17:**
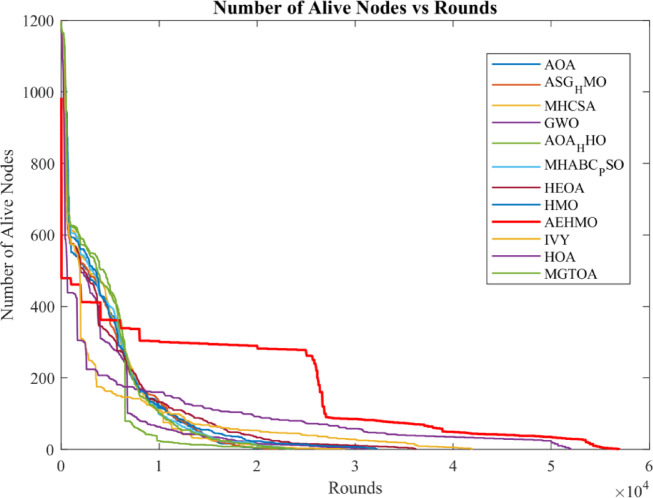
Number of alive nodes versus number of rounds for the complex scenario.

The network survivability analysis as shown in Fig. 17 shows AEHMO’s superior performance in keeping operational nodes alive during the network lifetime. AEHMO exhibits strong node preservation, where all the 1200 nodes remain alive for a much longer time than competing algorithms. The graph shows that AEHMO maintains roughly 300 nodes in operation after 25,000 rounds and most of the competitors have network-wide node depletion before 40,000 rounds. This long survivability is attributed to AEHMO’s intelligent CH selection mechanism that considers the three-tier heterogeneity through the comparative bias parameter ($\:F{P}_{4}$) so that advanced nodes with higher energy reserves (1.5 J) are selected as cluster heads during the critical phases of the network. The slowly decreasing alive nodes curve of AEHMO is in sharp contrast with the rapidly declining alive-node curves of the algorithms like AOA, ASG-HMO and MHABC-PSO which indicates balanced energy consumption for the heterogeneous network topology. Notably, HEOA and HMO are relatively better than other competitors in terms of keeping nodes alive until about 36,000 and 32,000 rounds respectively, although they are still significantly inferior to AEHMO. The better performance is attributed to the synergistic combination of the adaptive parameters $\:{w}_{1}$ and $\:{w}_{2}$ in AEHMO which dynamically control the balance of exploration and exploitation, DDS for thorough exploration of the spatial search space for CH selection, MDMS for CH candidate refinement, and CRL for escaping suboptimal configurations which would otherwise lead to premature energy depletion in the critical areas of the network.

**Fig. 18:**
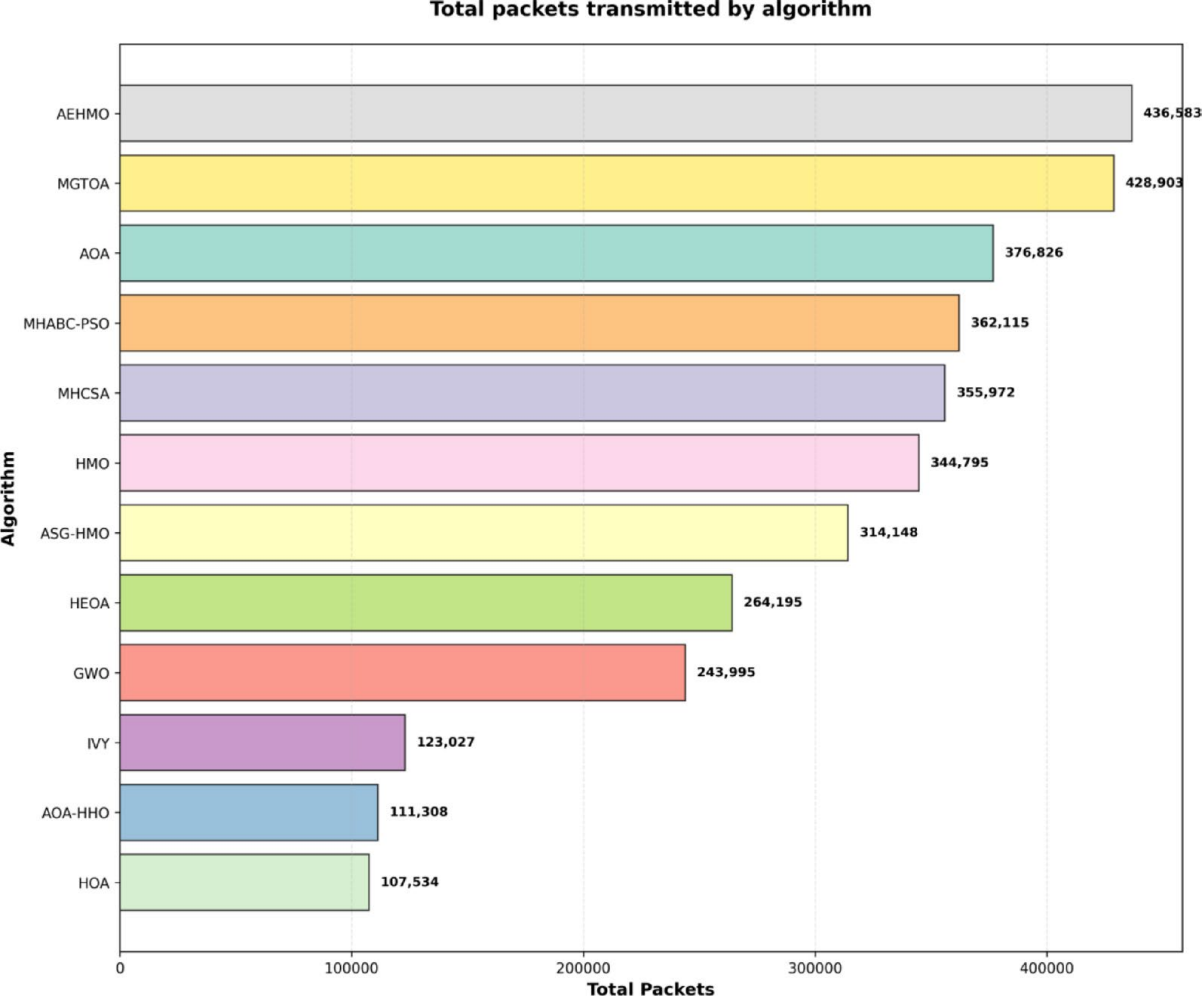
Number of total packets for each algorithm for the complex scenario.

The total packet transmission analysis, as shown in Fig. 18, indicates that AEHMO demonstrates strong data-delivery capability in the heterogeneous mobile sink situation. AEHMO gets the highest packet delivery with 436,583 packets successfully transmitted to the base station which is closely followed by MGTOA with 428,903 packets. This represents a substantial improvement over several baseline algorithms, with AEHMO sending around 16% more packets than AOA (376,826 packets), 27% more than GWO (243,995 packets) and nearly four times that of the lowest-performing methods (HOA (107,534 packets) and AOA-HHO (111,308 packets)). Higher packet delivery is directly correlated with AEHMO’s extended network lifetime and efficient energy management as nodes that remain operational for longer periods of time can continuously sense and transmit data to the mobile sinks. The multi-hop routing strategies integrated with AEHMO’s CH selection further improves the packet delivery by optimizing the transmission paths based on the dynamic sink positions, reducing packet loss caused by long multi-hop transmission distances. The competitive performance of MGTOA implies that nature inspired algorithms with good exploration mechanisms can achieve high packet throughput in mobile sink situations, but AEHMO’s adaptive mechanisms are slightly stronger and more consistent. The middle-tier performers (MHABC-PSO: 362,115; MHCSA: 355,972; HMO: 344,795; ASG-HMO: 314,148) suggest that hybrid metaheuristic approaches provide enhanced performances over the single-strategy algorithms, but not as comprehensive enhancement mechanisms as AEHMO. The poor performance of algorithms like IVY (123,027), AOA-HHO (111,308), HOA (107,534) can be explained by the fact that they deplete energy rapidly and they are not able to adapt their CH selection strategy to the energy heterogeneous distribution and mobile sink trajectories resulting in early network partitioning and communication failures.

**Fig. 19:**
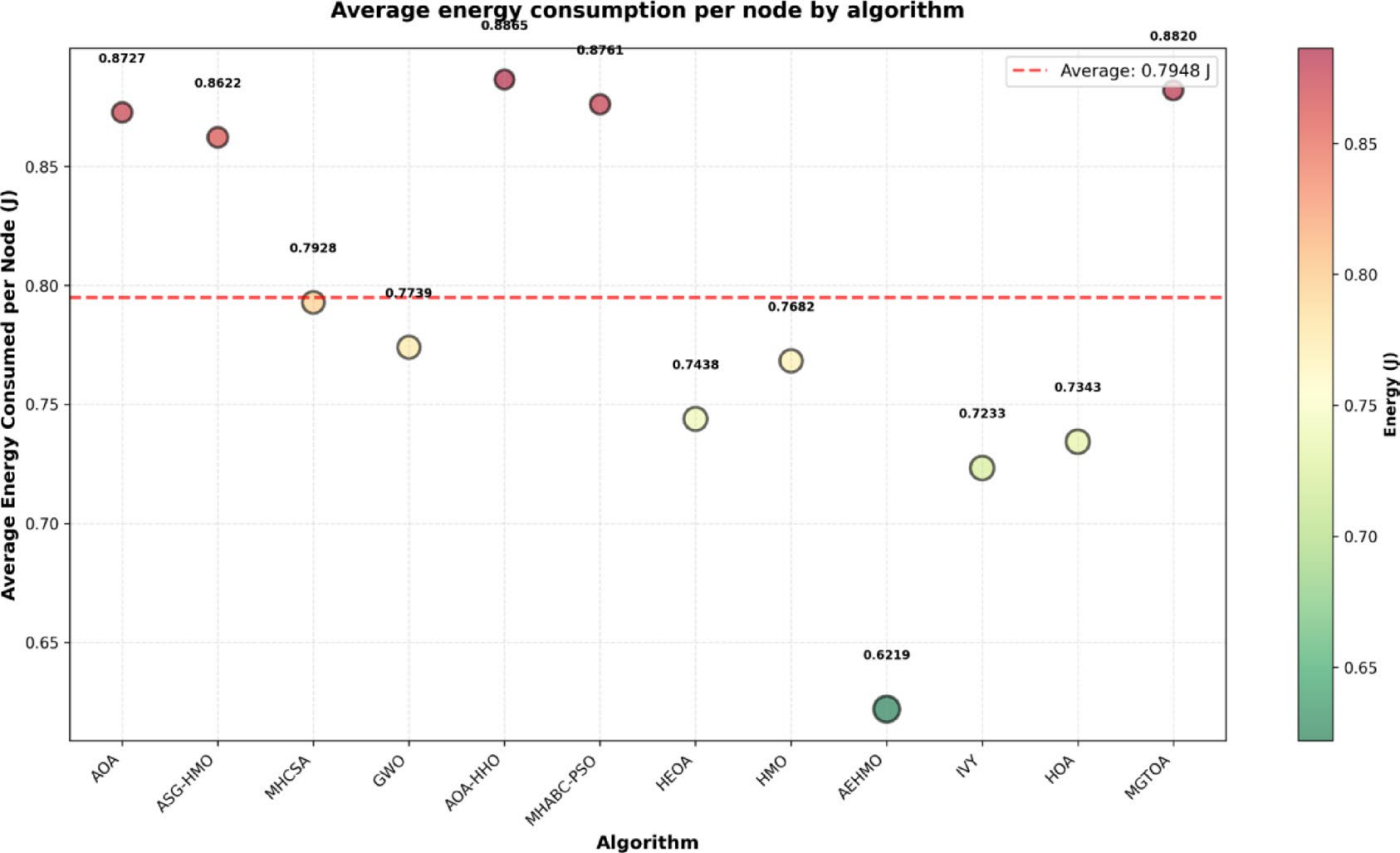
Energy consumption of each algorithm for the complex scenario.

The average energy consumption per node analysis, as shown in Fig. 19, demonstrates strong energy efficiency of AEHMO in the large-scale heterogeneous network. AEHMO has the lowest average energy consumption at 0.6219 J per node, which is significantly better than all the other competing algorithms and is well below the overall average (0.7948 J), as indicated by the dashed reference line. This corresponds to ~ 21.8% lower energy consumption than the competitor average and shows significant improvements over high consuming algorithms such as MGTOA (0.8820 J), AOA (0.8727 J), AOA-HHO (0.8761 J) and ASG-HMO (0.8622 J). The advantage in terms of energy efficiency is more significant when it is compared to algorithms such as MHABC-PSO (0.8761 J) and HEOA (0.7438 J) with AEHMO consuming 29% and 16.4% less energy, respectively. The superior energy performance is due to several complementary mechanisms: (1) Through FP4 in the fitness function, advanced (1.5 J) and intermediate (1.0 J) nodes are preferentially selected as cluster heads, and normal nodes (0.5 J) will not be prematurely depleted of energy; (2) the intra-cluster and inter-cluster multi-hop routing schemes reduce energy costly long distance transmissions by intelligently routing data through intermediate nodes; (3) The mobile-sink strategy minimizes average CH-to-sink distance, reinforced by FP2 in the fitness function. The large gap of energy consumption between the best (AEHMO: 0.6219 J) and worst (MGTOA: 0.8820 J) performers, which is about 41.8% highlights the importance of intelligent CH selection and routing optimization for prolonging network lifetime in large-size heterogeneous WSN deployment.

The comprehensive network lifetime analysis in terms of FND, HND and LND metrics presented in Table 11 proves the outstanding performance of AEHMO in the heterogeneous mobile sink scenario. For FND, AEHMO achieves 362 rounds before the first node death, representing a substantial improvement of 87.6% as compared to the second-best performers GWO and MHCSA (both with 193 rounds), 85.6% as compared to HMO (195 rounds) and 209.4% as compared to worst performers AOA-HHO (117 rounds) and MHABC-PSO (127 rounds). This high FND improvement is a direct validation of the effectiveness of the comparative bias parameter (FP4) and the hierarchical energy configuration strategy as AEHMO manages to prevent premature depletion of low-capacity normal nodes by giving priority to advanced and intermediate nodes to perform CH duties in the critical early operation phases. The HND metric goes on to prove AEHMO’s superiority, with 1903 rounds (the point where half of the network is still operating) compared to the second-best performer AOA-HHO with 1873 rounds, MGTOA with 1791 rounds, and MHABC-PSO with 1514 rounds. The minor difference between AEHMO and AOA-HHO at HND (1.6% difference) is in stark contrast to the large FND advantage (209% improvement), suggesting that although the performance of both algorithms will eventually be similar, the early-stage energy management advantage of AEHMO gives it important operational advantages. Most importantly, the LND metric, which is total network lifetime, AEHMO managed to achieve 56,946 rounds, which is an extraordinary improvement over the second-best performer IVY (41,928 rounds), 49.3% better than HOA (38,147 rounds), 57.3% better than HEOA (36,212 rounds), and 76.8% longer than HMO (32,214 rounds). When compared to the worst performers in the large-scale scenario, AEHMO shows even more dramatic advantages: 146.5% longer LND than MGTOA (23,096 rounds), 139.3% longer than AOA-HHO (23,804 rounds) and longer than the lowest-performing AOA (21,374 rounds).

**Table 11:** Network lifetime for each algorithm for the complex scenario.

Algorithm	FND	HND	LND
AOA	139	867	21,374
ASG-HMO	133	759	22,075
MHCSA	193	1493	28,911
GWO	193	799	31,146
AOA-HHO	117	1873	23,804
MHABC-PSO	127	1514	23,152
HEOA	127	799	36,212
HMO	195	935	32,214
AEHMO	362	1903	56,946
IVY	147	799	41,928
HOA	161	395	38,147
MGTOA	188	1791	23,096

### Communication overhead analysis

A communication overhead analysis was conducted across network scales ranging from 50 to 1000 nodes to evaluate the scalability of AEHMO in terms of control message exchange required for CH selection and network maintenance. AEHMO consistently achieved the lowest overhead across all network sizes, reaching 2.5%, 4.0%, 8.5%, and 12.0% at 100, 150, 500, and 1000 nodes respectively — representing reductions of up to 46.7% compared to the worst-performing competitor IVY. This efficiency is attributable to the memory-based recall mechanism that reduces redundant fitness evaluations, the DDS strategy that accelerates convergence in early iterations, and the MDMS selective mutation applied only to the top 20–30% elite solutions. Full overhead comparisons across all algorithms and network scales are provided in Supplementary Appendix J, Table J1.

### Comparison between AEHMO and other state-of-the-art approaches

This section presents a comparison of the proposed AEHMO algorithm with the recent and state-of-the-art algorithms in terms of some key performance metrics: FND, HND, LND, Average Energy Consumption, Number of Transmitted Packets to BS. The comparison underlines the superiority of AEHMO in terms of prolonging network lifetime, reducing energy consumption, and increasing the number of data packets transmitted to BS. Table 12 describes the results for different metrics.

FND is an important metric that reflects the network’s early-stage energy management effectiveness. In Table 12, the FND of the proposed algorithm AEHMO has reached 1150 rounds, whereas the algorithms PSOECSM and AROA have achieved only 698 and 834 rounds respectively. These improvements in FND are mainly rooted in the fact that AEHMO’s DDS and MDMS make the selection of cluster heads wiser, while energy is distributed among nodes more evenly to delay the death of the first node. Moreover, AEHMO has also recorded the highest value of 1853 rounds regarding the HND metric, as reflected in Table 12. Other algorithms such as PSOECSM, GAPSO-H, and SWARAM recorded HND values as low as 1392 and 1412 rounds due to their inefficient energy management techniques. Regarding the LND metric, AEHMO reaches 3136 rounds, which outperforms the performance achieved by other algorithms such as PSOECSM with 2933 rounds and SWARAM with 2591 rounds. This denotes the potential of the AEHMO algorithm to extend the overall network lifetime.

**Table 12:** Comparison between AEHMO and other state-of-the-art works.

	FND	HND	LND
PSOECSM^67^	698	1392	2933
GAPSO-H^68^	767	1412	2718
MOCRAW^69^	816	1183	2639
AROA^70^	834	1481	2874
SWARAM^29^	633	1392	2591
SHO-OBL^36^	604	1101	2431
EHCR-FCM^71^	752	1342	2531
Proposed	1150	1853	3136

These results clearly demonstrate that AEHMO outperforms all compared state-of-the-art algorithms across all metrics, and it is a highly efficient solution to extend network lifetime, reduce energy consumption, and enhance data transmission. The proposed strategies, DDS and MDMS, along with adaptive parameters and CRL play an important role in energy optimization and ensuring better performance in general.

## Conclusion and future directions

This paper presented an Adaptive Enhanced Human Memory Optimization algorithm (AEHMO) for global optimization and energy-efficient CH selection in WSNs. AEHMO incorporates adaptive parameters $\:\boldsymbol{w}1$ and $\:\boldsymbol{w}2$, MDMS, DDS, and CRL, which collectively enhance its exploration-exploitation balance and accelerate convergence. AEHMO was rigorously evaluated on the CEC2017 benchmark suite, demonstrating strong performance on complex optimization problems. In terms of global optimization performance, AEHMO achieved the best Friedman ranks of 2.15, 2.37, 2.87, and 2.23 across 10D, 30D, 50D, and 100D problem dimensions respectively on the CEC2017 benchmark suite, consistently outperforming eleven competing algorithms including GWO, AOA, HMO, HEOA, HOA, IVY, MGTOA, MHCSA, AOA-HHO, MHABC-PSO, and ASG-HMO. The Wilcoxon rank-sum test confirmed statistically significant superiority ($\:p$ < 0.001) across all dimensional scales, with a mean effect size r ranging between 0.853 and 0.873, validating both the statistical and practical significance of the improvements. Furthermore, AEHMO demonstrated strong engineering optimization capability, achieving perfect or near-perfect convergence consistency across five classical engineering design problems including Tension-Compression Spring, Tubular Column, Cantilever Beam, and Corrugated Bulkhead designs. Regarding CH selection in homogeneous WSN scenarios, AEHMO achieved an FND of 752 rounds, HND of 1694 rounds, LND of 3010 rounds, and the lowest average energy consumption of 0.381 J in the 100-node scenario, while in the 150-node scenario it achieved an FND of 1150 rounds, HND of 1853 rounds, LND of 3136 rounds, and average energy consumption of 0.395 J, representing improvements of up to 81.7% in FND and 4.6% lower energy consumption compared to competing algorithms. In terms of data delivery, AEHMO successfully transmitted 16,316 packets to the BS in the 100-node scenario, approximately 6.5% more than the second-best algorithm MGTOA (15,317 packets), and 38,257 packets in the 150-node scenario, approximately 4.7% more than ASG-HMO (36,557 packets).

Most notably, validation under a large-scale real-world heterogeneous WSN scenario comprising 1200 sensor nodes with a three-tier node architecture and a mobile sink strategy demonstrated AEHMO’s exceptional scalability, achieving an FND of 362 rounds, HND of 1903 rounds, and LND of 56,946 rounds. These results represent improvements in total network lifetime over the second-best performer IVY (41,928 rounds), 76.8% improvement over HMO (32,214 rounds), and 146.5% improvement over MGTOA (23,096 rounds). Additionally, AEHMO achieved the lowest average energy consumption of 0.6219 J per node, approximately 21.8% below the competitor average, and the highest packet delivery of 436,583 packets to the base station. The ablation study further confirmed that all four enhancement strategies contribute synergistically, with the full AEHMO configuration achieving 56.3% and 38.1% FND improvement over baseline HMO in the 100-node and 150-node scenarios respectively.

Despite these achievements, several limitations persist in the current study. For instance, AEHMO’s adaptability to highly dynamic and real-time network environments has not been thoroughly investigated, and parameter adaptation mechanisms may require further refinement to ensure optimal performance under diverse network conditions. Future work will focus on enhancing the scalability and adaptability of AEHMO for deployment in real-time and dynamic WSN environments. The integration of self-adjusting mechanisms and dynamic parameter tuning will improve AEHMO’s responsiveness to fluctuating network conditions and mobile WSN deployments. Another promising direction is the integration of machine learning or deep learning models with AEHMO. Such hybrid models could enhance AEHMO’s adaptability by enabling data-driven CH selection decisions that respond to real-time network dynamics. Further validation across real-world applications such as environmental monitoring, smart cities, and industrial automation will demonstrate the broader applicability and effectiveness of AEHMO. Additionally, extending AEHMO to cooperative multi-agent network designs — where node interactions are explicitly modeled — could further improve packet delivery ratios and overall energy efficiency. Furthermore, physical deployment and validation of AEHMO on real sensor mote platforms, such as Raspberry Pi or Arduino-based nodes, **represent** a valuable future direction that would confirm the algorithm’s practical applicability beyond simulation. Finally, applying AEHMO to broader domains such as image processing, robotics, and smart agriculture will be explored to further demonstrate its versatility as a general-purpose global optimizer.

## Supplementary Information

Below is the link to the electronic supplementary material.

Supplementary Material 1

## Data Availability

All data generated or analyzed during this study are included directly in the text of this submitted manuscript.
