# Supplementary material for: An improved human memory algorithm with multi-directional and chaotic approaches for global optimization and energy-efficient cluster head selection in WSNs
Source: Sci Rep. 2026 Jul 16;16:22414. doi: 10.1038/s41598-026-61131-3 (PMC13377191; doi:10.1038/s41598-026-61131-3)
Supplement: Supplementary file 1 — Supplementary Material 1 [file 41598_2026_61131_MOESM1_ESM.pdf]

Appendix A: Sensitivity Analysis of AEHMO Parameters (CEC2017)

Effect of the Depth of Chaotic Iteration (z) in CRL

The depth of chaotic iteration z controls the degree of chaotic sequences to be generated in the CRL strategy and thus controls the degree of structured randomness to be introduced in the opposition-based learning process. Table A1 presents the results of the Friedman ranks obtained for different values of z. The results in Table A1 show that small values of z yield poor performance, indicating that too few chaotic iterations fail to provide sufficient diversity for escaping local optima. Increasing z results in increasing exploring capability with the best Friedman rank being achieved at $z = 20$. This is a good way of confirming that $z = 20$ is sufficient to add good amounts of chaotic depth to the search without losing the convergence stability. Although values that are close to those ($z = 15$ and $z = 25$) produce competitive rankings, increasing z to 30 degrades performance because of unnecessary repetition of chaotic mappings and increases in computation effort without proportional increase in performance. Therefore, $z = 20$ is not arbitrarily selected; it represents a balanced midpoint that maximizes diversity enhancement while minimizing computational overhead, and the stable performance of the range 15-25 is a testament to AEHMO's robustness to this parameter.

Table A1 Sensitivity analysis of chaotic iteration depth (z) on CEC2017.

z value	Friedman Rank (FR)
10	3.92
15	2.41
**20**	**1.47**
25	1.69
30	2.88

Effect of MDMS Mutation Percentage

The mutation percentage in the MDMS strategy **controls** the proportion of elite solutions to be refined at each iteration and is also used to influence directly the trade-off between exploitation and population stability. Table A2 presents the Friedman ranks that were obtained for values of mutation percentage ranging from 10% to 50%.

As shown in Table A2, mutating only 10% of the population yields poor performance due to insufficient exploitation of high-quality solutions. Increasing the mutation percentage to 20%-30% gives considerable improvement of the Friedman rank, with the best result obtained for 30%, suggesting that moderate elite refinement is an effective method to increase the rate of convergence. When percentage of mutation is greater than 30%, it is poor because too much mutation breaks continuity of elite solution and injects instability in the search process. At 50%, the algorithm is more like random perturbation and the convergence efficiency is compromised. These results provide an explicit support for the choice of the 20%-30% range as this provides the best balance between intensification of promising areas and conservation of global diversity. Moreover, the steady performance degradation outside this range confirms that AEHMO does not depend on a finely tuned mutation rate, demonstrating robust behavior across a wide parameter interval.

Table A2 Sensitivity analysis of MDMS mutation percentage on CEC2017

Mutation Percentage	Friedman Rank (FR)
10%	4.26
20%	2.31
**30%**	**1.52**
40%	2.94

Mutation Percentage	Friedman Rank (FR)
50%	4.11

Effect of Scaling Factor (c) of MDMS

The scaling factor c is used to control the amplitude of Gaussian and Cauchy mutations in the MDMS strategy and has a direct effect on the step size during the solution refinement. Table A3 summarizes the Friedman ranks that were found for the range of c values from 0.1 to 0.9.

Table A3 Sensitivity analysis of scaling factor (c) on CEC2017

c value	Friedman Rank (FR)
0.1	4.63
0.3	3.74
0.5	2.69
0.7	1.98
**0.8**	**1.44**
0.9	2.37

The results in Table A3 show that small values of c (0.1–0.3) result in overly conservative mutations, limiting the algorithm's ability to escape local optima and causing it to converge more slowly. As c increases, performance is steadily improved, and the best Friedman rank is achieved at c = 0.8, where mutation steps are sufficiently large to explore promising neighborhoods while remaining stable enough for effective exploitation. Increasing c to a value of 0.9 further results in a slight performance decrease due to too-aggressive perturbations that destabilize local refinement. These observations give explicit justification to choose c = 0.8 since this is the best possible compromise between exploration strength and exploitation precision. Additionally, the smooth trend of performance over 0.7-0.9 reinforces that AEHMO is stable over a wide parameter range reinforcing that the chosen value is principled rather than arbitrary.

The parameter sensitivity analysis demonstrates that AEHMO maintains stable and competitive behavior across wide parameter ranges, and each chosen value (z = 20, mutation percentage = 20%–30%, c = 0.8) has direct empirical justification. The observed trends confirm the choice of these parameters in consideration of the balanced performance aspects rather than fine-tuning that will ensure the robustness and generalization of AEHMO across a variety of optimization landscapes.

Appendix B: CEC2017 Ablation Full Results

Table B1 Statistical measurements for the ablation study using CEC2017.

Function	Metric	AEHMO	HMO	AHMO	CHMO	DHMO	MHMO
F1	AVG	6.66E+03	3.41E+06	2.89E+06	5.09E+06	5.21E+03	1.15E+08
	STD	4.59E+03	4.72E+06	5.50E+06	1.03E+07	3.80E+03	6.19E+08
	RANK	2	4	3	5	1	6
F3	AVG	3.00E+02	8.30E+02	4.54E+02	4.90E+02	3.00E+02	7.17E+02
	STD	4.44E-02	1.06E+03	1.27E+02	3.96E+02	9.83E-02	9.34E+02
	RANK	1	6	3	4	2	5
F4	AVG	4.04E+02	4.10E+02	4.27E+02	4.13E+02	4.04E+02	4.24E+02
	STD	2.47E+00	1.71E+01	3.63E+01	2.90E+01	2.24E+00	4.13E+01
	RANK	1	3	6	4	2	5
F5	AVG	5.20E+02	5.46E+02	5.45E+02	5.46E+02	5.18E+02	5.46E+02
	STD	7.88E+00	1.72E+01	1.60E+01	1.86E+01	8.15E+00	1.68E+01
	RANK	2	5	3	6	1	4
F6	AVG	6.00E+02	6.29E+02	6.25E+02	6.26E+02	6.00E+02	6.21E+02
	STD	5.80E-01	1.11E+01	1.17E+01	1.11E+01	8.06E-01	9.01E+00
	RANK	1	6	4	5	2	3
F7	AVG	7.34E+02	7.76E+02	7.68E+02	7.83E+02	7.26E+02	7.64E+02
	STD	8.66E+00	2.50E+01	1.97E+01	1.96E+01	6.47E+00	2.35E+01
	RANK	2	5	4	6	1	3
F8	AVG	8.23E+02	8.37E+02	8.36E+02	8.34E+02	8.22E+02	8.35E+02
	STD	8.70E+00	1.33E+01	1.38E+01	9.11E+00	1.08E+01	1.28E+01
	RANK	2	6	5	3	1	4
F9	AVG	9.00E+02	1.25E+03	1.21E+03	1.30E+03	9.00E+02	1.09E+03
	STD	2.47E-01	2.65E+02	2.06E+02	2.07E+02	2.95E-01	1.16E+02
	RANK	1	5	4	6	2	3
F10	AVG	1.56E+03	1.88E+03	1.91E+03	1.90E+03	1.54E+03	1.77E+03
	STD	2.60E+02	2.80E+02	2.86E+02	2.67E+02	1.55E+02	3.05E+02
	RANK	2	4	6	5	1	3
F11	AVG	1.12E+03	1.18E+03	1.18E+03	1.18E+03	1.12E+03	1.18E+03
	STD	2.63E+01	5.28E+01	7.35E+01	4.44E+01	9.09E+00	6.47E+01
	RANK	2	5	4	3	1	6
F12	AVG	7.36E+04	5.11E+06	4.75E+06	4.20E+06	8.16E+04	4.35E+06
	STD	1.22E+05	3.52E+06	4.12E+06	3.32E+06	1.51E+05	3.94E+06
	RANK	1	6	5	3	2	4
F13	AVG	1.11E+04	1.52E+04	1.51E+04	1.43E+04	1.31E+04	1.78E+04
	STD	1.04E+04	1.07E+04	9.43E+03	9.67E+03	1.10E+04	1.35E+04
	RANK	1	5	4	3	2	6
F14	AVG	1.44E+03	1.60E+03	1.68E+03	1.57E+03	1.45E+03	1.59E+03
	STD	2.99E+01	1.47E+02	3.39E+02	1.10E+02	4.48E+01	1.45E+02
	RANK	1	5	6	3	2	4
F15	AVG	1.69E+03	8.05E+03	8.24E+03	5.29E+03	2.66E+03	4.52E+03
	STD	3.31E+02	2.99E+03	4.16E+03	3.13E+03	1.17E+03	2.27E+03
	RANK	1	5	6	4	2	3
F16	AVG	1.74E+03	1.93E+03	1.95E+03	1.89E+03	1.80E+03	1.82E+03
	STD	1.09E+02	1.75E+02	1.56E+02	1.27E+02	1.31E+02	1.28E+02
	RANK	1	5	6	4	2	3
F17	AVG	1.75E+03	1.84E+03	1.80E+03	1.77E+03	1.80E+03	1.79E+03
	STD	2.60E+01	8.32E+01	3.86E+01	2.53E+01	7.57E+01	4.33E+01
	RANK	1	6	4	2	5	3
F18	AVG	1.30E+04	1.88E+04	2.57E+04	2.43E+04	1.93E+04	3.02E+04
	STD	1.01E+04	1.27E+04	1.42E+04	1.64E+04	1.22E+04	1.77E+04
	RANK	1	2	5	4	3	6
F19	AVG	2.85E+03	7.69E+04	6.59E+04	4.40E+04	3.03E+03	1.87E+04
	STD	1.49E+03	1.42E+05	1.77E+05	7.01E+04	1.94E+03	3.92E+04
	RANK	1	6	5	4	2	3
F20	AVG	2.03E+03	2.16E+03	2.16E+03	2.16E+03	2.03E+03	2.13E+03
	STD	3.47E+01	7.43E+01	6.99E+01	7.79E+01	4.24E+01	6.15E+01
	RANK	1	4	5	6	2	3
F21	AVG	2.26E+03	2.34E+03	2.28E+03	2.26E+03	2.32E+03	2.30E+03
	STD	6.47E+01	3.12E+01	5.71E+01	6.53E+01	3.99E+01	5.62E+01
	RANK	1	6	3	2	5	4
F22	AVG	2.30E+03	2.43E+03	2.31E+03	2.32E+03	2.30E+03	2.31E+03
	STD	2.01E+01	4.40E+02	1.32E+01	7.70E+01	1.24E+01	1.55E+01
	RANK	1	6	4	5	2	3
F23	AVG	2.62E+03	2.66E+03	2.66E+03	2.65E+03	2.63E+03	2.64E+03
	STD	7.19E+00	2.33E+01	2.19E+01	1.85E+01	9.43E+00	1.67E+01
	RANK	1	5	6	4	2	3
F24	AVG	2.72E+03	2.79E+03	2.76E+03	2.74E+03	2.76E+03	2.76E+03
	STD	1.01E+02	3.50E+01	8.69E+01	1.13E+02	5.16E+01	5.05E+01
	RANK	1	6	4	2	3	5
F25	AVG	2.94E+03	2.95E+03	2.94E+03	2.95E+03	2.92E+03	2.94E+03
	STD	1.84E+01	3.44E+01	2.67E+01	3.64E+01	2.36E+01	2.88E+01
	RANK	2	5	3	6	1	4
F26	AVG	2.95E+03	3.59E+03	3.52E+03	3.29E+03	3.05E+03	3.18E+03
	STD	2.07E+02	4.97E+02	4.57E+02	4.00E+02	3.83E+02	2.34E+02
	RANK	1	6	5	4	2	3
F27	AVG	3.10E+03	3.16E+03	3.14E+03	3.17E+03	3.10E+03	3.10E+03
	STD	1.13E+01	5.41E+01	4.06E+01	6.11E+01	1.97E+01	4.88E+00
	RANK	1	5	4	6	3	2
F28	AVG	3.32E+03	3.59E+03	3.55E+03	3.47E+03	3.47E+03	3.39E+03
	STD	1.24E+02	1.33E+02	1.54E+02	1.88E+02	1.58E+02	1.31E+02
	RANK	1	6	5	3	4	2
F29	AVG	3.22E+03	3.30E+03	3.32E+03	3.31E+03	3.25E+03	3.25E+03
	STD	4.63E+01	1.17E+02	1.10E+02	9.35E+01	6.83E+01	6.33E+01
	RANK	1	4	6	5	2	3
F30	AVG	8.50E+04	5.94E+05	5.07E+05	8.35E+05	7.50E+04	5.54E+05
	STD	2.26E+05	7.83E+05	4.70E+05	1.69E+06	2.66E+05	7.91E+05
	RANK	2	5	3	6	1	4
Average rank	Average rank	1.28	5.07	4.52	4.24	2.10	3.79
Final rank	Final rank	1	6	5	4	2	3

Appendix C: CEC2017 Full Results

Table C1 Statistical analysis of AEHMO and other algorithms using CEC2017, 10D

F		AEHMO	HMO	MGTOA	AOA	IVY	GWO	HEOA	HOA	MHCSA	AOA-HHO	MHABC-PSO	ASG-HMO
F1	AVG	1.00E+02	4.68E+02	2.42E+03	8.32E+07	1.50E+08	3.42E+06	8.06E+08	7.47E+09	3.61E+03	4.15E+03	3.61E+03	2.49E+03
	STD	2.15E-06	3.22E+02	1.37E+03	1.41E+08	2.12E+08	8.09E+06	5.50E+08	2.54E+09	1.25E+03	2.78E+03	1.31E+03	2.65E+03
	RANK	1	2	3	9	10	8	11	12	6	7	5	4
F3	AVG	3.00E+02	3.00E+02	3.00E+02	3.90E+03	4.60E+03	1.68E+03	1.15E+04	1.17E+04	3.00E+02	3.00E+02	4.14E+02	3.00E+02
	STD	0.00E+00	9.02E-03	6.18E-02	2.16E+03	2.97E+03	1.76E+03	2.11E+03	2.91E+03	7.19E-10	3.81E-09	8.64E+01	1.10E+00
	RANK	1	4	5	9	10	8	11	12	2	3	7	6
F4	AVG	4.00E+02	4.03E+02	4.04E+02	4.36E+02	4.45E+02	4.29E+02	4.57E+02	1.01E+03	4.01E+02	4.01E+02	4.04E+02	4.04E+02
	STD	4.93E-03	2.63E+00	3.28E+00	1.97E+01	3.53E+01	2.01E+01	4.31E+01	4.26E+02	6.33E-01	6.14E-01	3.27E+00	2.29E+00
	RANK	1	4	6	9	10	8	11	12	3	2	7	5
F5	AVG	5.24E+02	5.41E+02	5.31E+02	5.33E+02	5.28E+02	5.16E+02	5.79E+02	5.58E+02	5.23E+02	5.19E+02	5.56E+02	5.17E+02
	STD	8.36E+00	1.10E+01	1.38E+01	8.67E+00	8.63E+00	8.39E+00	9.97E+00	1.10E+01	6.10E+00	6.59E+00	1.30E+01	4.67E+00
	RANK	5	9	7	8	6	1	12	11	4	3	10	2
F6	AVG	6.00E+02	6.25E+02	6.01E+02	6.14E+02	6.08E+02	6.01E+02	6.55E+02	6.35E+02	6.02E+02	6.01E+02	6.33E+02	6.01E+02
	STD	4.12E-01	1.47E+01	1.14E+00	8.15E+00	6.01E+00	7.71E-01	8.77E+00	8.08E+00	3.13E+00	1.82E+00	1.32E+01	1.07E+00
	RANK	1	9	5	8	7	3	12	11	6	4	10	2
F7	AVG	7.34E+02	7.85E+02	7.52E+02	7.62E+02	7.66E+02	7.24E+02	8.11E+02	7.68E+02	7.55E+02	7.56E+02	7.85E+02	7.30E+02
	STD	9.04E+00	2.57E+01	1.88E+01	1.13E+01	1.48E+01	6.00E+00	2.21E+01	1.91E+01	1.56E+01	1.73E+01	1.35E+01	1.46E+01
	RANK	3	11	4	7	8	1	12	9	5	6	10	2
F8	AVG	8.14E+02	8.33E+02	8.20E+02	8.25E+02	8.26E+02	8.15E+02	8.52E+02	8.31E+02	8.27E+02	8.28E+02	8.24E+02	8.16E+02
	STD	4.92E+00	1.36E+01	7.93E+00	4.64E+00	9.26E+00	4.46E+00	9.75E+00	5.25E+00	5.90E+00	1.93E+00	4.42E+00	5.29E+00
	RANK	1	11	4	6	7	2	12	10	8	9	5	3
F9	AVG	9.01E+02	1.17E+03	9.34E+02	1.06E+03	9.90E+02	9.14E+02	1.63E+03	1.21E+03	9.46E+02	9.38E+02	1.50E+03	9.08E+02
	STD	1.17E+00	1.64E+02	7.04E+01	1.77E+02	9.72E+01	1.58E+01	3.46E+02	1.47E+02	7.15E+01	6.44E+01	1.94E+02	5.14E+00
	RANK	1	9	4	8	7	3	12	10	6	5	11	2
F10	AVG	1.55E+03	1.90E+03	1.68E+03	1.69E+03	1.78E+03	1.57E+03	2.55E+03	2.44E+03	1.59E+03	1.58E+03	1.93E+03	1.42E+03
	STD	3.23E+02	4.24E+02	3.31E+02	4.20E+02	3.01E+02	4.30E+02	3.06E+02	1.63E+02	2.05E+02	6.99E+01	2.78E+02	2.54E+02
	RANK	2	9	6	7	8	3	12	11	5	4	10	1
F11	AVG	1.11E+03	1.16E+03	1.12E+03	1.13E+03	1.21E+03	1.13E+03	1.33E+03	4.02E+03	1.12E+03	1.13E+03	1.12E+03	1.11E+03
	STD	5.65E+00	1.89E+01	1.06E+01	1.43E+01	8.72E+01	9.67E+00	1.62E+02	1.86E+03	2.68E+00	8.32E+00	9.72E+00	9.34E+00
	RANK	2	9	3	7	10	8	11	12	4	6	5	1
F12	AVG	1.41E+03	3.36E+05	8.82E+03	8.06E+05	9.44E+05	8.63E+05	4.85E+06	2.01E+07	7.60E+03	1.00E+04	2.93E+06	1.51E+04
	STD	7.62E+01	3.33E+05	5.56E+03	8.03E+05	9.47E+05	1.19E+06	4.01E+06	1.75E+07	2.60E+03	5.76E+03	3.12E+06	7.27E+03
	RANK	1	6	3	7	9	8	11	12	2	4	10	5
F13	AVG	1.31E+03	1.31E+04	8.81E+03	6.06E+03	1.33E+04	6.59E+03	6.60E+04	3.28E+04	7.88E+03	8.30E+03	1.66E+04	8.37E+03
	STD	3.06E+00	7.80E+03	4.05E+03	1.84E+03	8.49E+03	1.66E+03	4.32E+04	4.62E+04	6.33E+03	5.59E+03	1.18E+04	7.55E+03
	RANK	1	8	7	2	9	3	12	11	4	5	10	6
F14	AVG	1.42E+03	1.49E+03	1.47E+03	3.66E+03	2.14E+03	3.30E+03	2.60E+03	2.96E+03	1.88E+03	1.91E+03	4.66E+03	1.51E+03
	STD	2.39E+00	3.85E+01	2.39E+01	1.36E+03	1.48E+03	1.99E+03	1.09E+03	1.36E+03	3.45E+02	5.06E+02	2.96E+03	2.74E+01
	RANK	1	3	2	11	7	10	8	9	5	6	12	4
F15	AVG	1.50E+03	1.66E+03	1.70E+03	2.98E+03	4.43E+03	4.29E+03	1.19E+04	9.42E+03	2.02E+03	2.10E+03	4.49E+03	1.90E+03
	STD	5.17E-01	1.57E+02	9.59E+01	1.29E+03	1.53E+03	1.66E+03	1.54E+03	2.64E+03	5.61E+02	4.47E+02	1.45E+03	2.03E+02
	RANK	1	2	3	7	9	8	12	11	5	6	10	4
F16	AVG	1.62E+03	1.86E+03	1.78E+03	1.85E+03	1.82E+03	1.70E+03	2.08E+03	1.99E+03	1.80E+03	1.80E+03	1.92E+03	1.72E+03
	STD	4.91E+01	1.38E+02	1.11E+02	1.11E+02	1.31E+02	5.62E+01	1.70E+02	1.39E+02	1.36E+02	9.33E+01	7.24E+01	1.24E+02
	RANK	1	9	4	8	7	2	12	11	6	5	10	3
F17	AVG	1.72E+03	1.77E+03	1.79E+03	1.75E+03	1.76E+03	1.76E+03	1.86E+03	1.79E+03	1.76E+03	1.75E+03	1.77E+03	1.76E+03
	STD	1.40E+01	2.57E+01	3.24E+01	8.21E+00	1.82E+01	1.95E+01	5.05E+01	1.44E+01	2.02E+01	2.81E+01	1.48E+01	2.34E+01
	RANK	1	9	10	2	6	4	12	11	7	3	8	5
F18	AVG	1.81E+03	6.89E+03	5.83E+03	1.87E+04	3.71E+04	3.11E+04	2.18E+05	1.71E+08	1.51E+04	9.71E+03	7.28E+03	4.47E+03
	STD	1.06E+00	3.84E+03	3.79E+03	9.93E+03	3.94E+03	1.18E+04	1.15E+05	3.61E+08	8.02E+03	7.58E+03	4.25E+03	2.58E+03
	RANK	1	4	3	8	10	9	11	12	7	6	5	2
F19	AVG	1.90E+03	8.49E+03	1.99E+03	9.05E+03	1.28E+04	9.57E+03	1.54E+05	8.84E+04	9.89E+03	9.59E+03	8.31E+03	2.51E+03
	STD	4.30E-01	4.43E+03	1.19E+02	4.91E+03	5.89E+03	8.32E+03	2.41E+05	1.08E+05	5.04E+03	5.41E+03	6.67E+03	8.74E+02
	RANK	1	5	2	6	10	7	12	11	9	8	4	3
F20	AVG	2.01E+03	2.19E+03	2.07E+03	2.12E+03	2.12E+03	2.07E+03	2.32E+03	2.10E+03	2.11E+03	2.04E+03	2.17E+03	2.04E+03
	STD	8.87E+00	7.93E+01	5.05E+01	5.73E+01	7.38E+01	5.34E+01	9.08E+01	6.25E+01	4.41E+01	8.01E+00	6.26E+01	2.85E+01
	RANK	1	11	4	8	9	5	12	6	7	3	10	2
F21	AVG	2.26E+03	2.29E+03	2.32E+03	2.33E+03	2.33E+03	2.31E+03	2.38E+03	2.33E+03	2.22E+03	2.22E+03	2.29E+03	2.30E+03
	STD	6.36E+01	6.86E+01	8.00E+00	1.49E+01	8.50E+00	4.42E+00	2.73E+01	5.33E+01	5.24E+01	4.56E+01	6.58E+01	4.82E+01
	RANK	3	4	8	10	9	7	12	11	2	1	5	6
F22	AVG	2.30E+03	2.32E+03	2.25E+03	2.35E+03	2.34E+03	2.31E+03	2.33E+03	2.89E+03	2.29E+03	2.30E+03	2.30E+03	2.30E+03
	STD	1.15E+00	1.63E+01	4.06E+01	2.26E+01	4.31E+01	5.41E+00	4.10E+01	1.42E+02	3.28E+01	1.29E+00	3.24E+01	9.09E-01
	RANK	3	8	1	11	10	7	9	12	2	4	6	5
F23	AVG	2.62E+03	2.65E+03	2.66E+03	2.65E+03	2.64E+03	2.61E+03	2.69E+03	2.76E+03	2.62E+03	2.62E+03	2.64E+03	2.62E+03
	STD	7.19E+00	1.82E+01	3.10E+01	7.74E+00	1.21E+01	6.17E+00	1.69E+01	4.19E+01	5.78E+00	5.38E+00	1.88E+01	9.93E+00
	RANK	3	8	10	9	6	1	11	12	5	2	7	4
F24	AVG	2.66E+03	2.73E+03	2.68E+03	2.78E+03	2.77E+03	2.75E+03	2.84E+03	2.74E+03	2.71E+03	2.75E+03	2.76E+03	2.75E+03
	STD	1.24E+02	1.16E+02	1.41E+02	1.91E+01	1.13E+01	8.23E+00	3.60E+01	1.41E+02	1.03E+02	5.71E+00	1.38E+01	7.64E+00
	RANK	1	4	2	11	10	8	12	5	3	6	9	7
F25	AVG	2.93E+03	2.95E+03	2.94E+03	3.00E+03	2.94E+03	2.94E+03	2.98E+03	3.13E+03	2.92E+03	2.92E+03	2.93E+03	2.92E+03
	STD	2.33E+01	2.20E+00	9.58E-01	1.28E+02	3.58E+01	1.13E+01	3.96E+01	1.38E+02	2.45E+01	2.46E+01	3.89E+01	2.54E+01
	RANK	5	9	7	11	8	6	10	12	3	1	4	2
F26	AVG	3.09E+03	3.55E+03	2.96E+03	3.33E+03	3.09E+03	3.17E+03	3.41E+03	3.91E+03	2.94E+03	2.94E+03	3.16E+03	3.35E+03
	STD	1.07E+02	6.74E+02	1.56E+02	4.76E+02	2.19E+02	3.92E+02	3.54E+02	4.65E+02	1.12E+02	1.13E+02	2.13E+02	4.01E+02
	RANK	4	11	3	8	5	7	10	12	2	1	6	9
F27	AVG	3.10E+03	3.16E+03	3.14E+03	3.13E+03	3.10E+03	3.10E+03	3.17E+03	3.25E+03	3.09E+03	3.09E+03	3.10E+03	3.11E+03
	STD	4.87E+00	3.87E+01	4.95E+01	2.82E+01	1.37E+01	2.02E+01	3.85E+01	4.39E+01	2.93E+00	2.05E+00	4.50E+00	3.77E+00
	RANK	4	10	9	8	5	6	11	12	1	2	3	7
F28	AVG	3.21E+03	3.37E+03	3.37E+03	3.42E+03	3.38E+03	3.36E+03	3.58E+03	3.77E+03	3.41E+03	3.35E+03	3.37E+03	3.36E+03
	STD	1.64E+02	1.59E+02	6.26E+01	1.63E+02	8.95E+01	9.62E+01	7.60E+01	7.85E+01	9.30E-05	1.23E+02	1.35E+02	9.66E+01
	RANK	1	5	7	10	8	3	11	12	9	2	6	4
F29	AVG	3.16E+03	3.37E+03	3.22E+03	3.25E+03	3.21E+03	3.22E+03	3.41E+03	3.35E+03	3.19E+03	3.21E+03	3.29E+03	3.20E+03
	STD	1.34E+01	1.02E+02	2.84E+01	2.94E+01	2.93E+01	6.54E+01	1.08E+02	6.47E+01	3.39E+01	6.47E+01	4.60E+01	3.50E+01
	RANK	1	11	7	8	4	6	12	10	2	5	9	3
F30	AVG	3.43E+03	9.88E+05	5.55E+03	2.92E+06	4.67E+05	7.80E+05	1.55E+06	2.29E+06	1.95E+04	2.06E+04	2.42E+05	1.29E+04
	STD	2.41E+01	1.86E+06	2.50E+03	5.67E+06	6.29E+05	1.02E+06	1.27E+06	3.14E+06	1.03E+04	1.18E+04	3.11E+05	6.07E+03
	RANK	1	9	2	12	7	8	10	11	4	5	6	3
Average rank	Average rank	1.83	7.34	4.86	8.10	7.97	5.52	11.24	10.79	4.62	4.28	7.59	3.86
Final rank	Final rank	1	7	5	10	9	6	12	11	4	3	8	2

Table C2 Statistical analysis of AEHMO and other algorithms using CEC2017, 30D

F		AEHMO	HMO	MGTOA	AOA	IVY	GWO	HEOA	HOA	MHCSA	AOA-HHO	MHABC-PSO	ASG-HMO
F1	AVG	2.35E+03	3.18E+05	1.97E+09	1.94E+10	1.41E+10	2.13E+09	2.25E+10	4.58E+10	3.05E+03	6.40E+03	3.00E+09	2.92E+05
	STD	1.78E+03	1.26E+05	1.24E+09	5.14E+09	6.35E+09	1.46E+09	5.24E+09	8.90E+09	2.05E+03	6.44E+03	1.28E+09	3.95E+05
	RANK	1	5	6	10	9	7	11	12	2	3	8	4
F3	AVG	8.52E+03	4.36E+04	2.70E+04	6.65E+04	5.92E+04	5.16E+04	8.64E+04	8.03E+04	3.02E+03	3.96E+03	6.68E+04	7.14E+04
	STD	1.84E+03	9.43E+03	9.70E+03	6.32E+03	1.11E+04	1.30E+04	4.52E+03	8.61E+03	1.33E+03	1.29E+03	6.14E+03	1.35E+04
	RANK	3	5	4	8	7	6	12	11	1	2	9	10
F4	AVG	5.05E+02	5.36E+02	5.25E+02	2.59E+03	1.64E+03	6.15E+02	2.15E+03	9.00E+03	4.97E+02	4.91E+02	6.95E+02	5.13E+02
	STD	1.82E+01	1.05E+01	3.33E+01	1.14E+03	1.42E+03	4.89E+01	8.55E+02	1.35E+03	2.14E+01	2.03E+01	1.15E+02	2.97E+01
	RANK	3	6	5	11	9	7	10	12	2	1	8	4
F5	AVG	6.56E+02	7.63E+02	7.21E+02	7.57E+02	7.22E+02	6.24E+02	8.95E+02	8.43E+02	6.95E+02	6.82E+02	7.69E+02	5.70E+02
	STD	3.48E+01	4.33E+01	2.94E+01	2.99E+01	3.61E+01	4.00E+01	3.92E+01	2.97E+01	4.79E+01	5.18E+01	1.64E+01	1.09E+01
	RANK	3	9	6	8	7	2	12	11	5	4	10	1
F6	AVG	6.12E+02	6.62E+02	6.30E+02	6.41E+02	6.37E+02	6.22E+02	6.89E+02	6.74E+02	6.33E+02	6.29E+02	6.61E+02	6.14E+02
	STD	5.74E+00	5.27E+00	1.03E+01	5.48E+00	8.24E+00	7.34E+00	4.86E+00	5.54E+00	8.82E+00	8.67E+00	8.84E+00	5.43E+00
	RANK	1	10	5	8	7	3	12	11	6	4	9	2
F7	AVG	8.59E+02	1.23E+03	1.02E+03	1.12E+03	1.04E+03	9.79E+02	1.39E+03	1.27E+03	9.21E+02	9.79E+02	1.12E+03	8.74E+02
	STD	3.36E+01	1.38E+02	5.20E+01	5.50E+01	2.97E+01	5.47E+01	6.73E+01	4.07E+01	3.30E+01	9.60E+01	4.95E+01	2.75E+01
	RANK	1	10	6	8	7	4	12	11	3	5	9	2
F8	AVG	9.02E+02	9.73E+02	9.44E+02	9.94E+02	9.55E+02	9.15E+02	1.10E+03	1.08E+03	9.27E+02	9.45E+02	9.90E+02	8.92E+02
	STD	1.97E+01	2.54E+01	3.59E+01	5.01E+01	2.49E+01	2.28E+01	3.98E+01	1.57E+01	1.72E+01	1.84E+01	9.47E+00	2.43E+01
	RANK	2	8	5	10	7	3	12	11	4	6	9	1
F9	AVG	1.86E+03	5.14E+03	4.40E+03	6.26E+03	4.94E+03	3.19E+03	1.25E+04	7.41E+03	3.61E+03	3.99E+03	5.74E+03	2.04E+03
	STD	8.33E+02	8.18E+02	1.44E+03	9.26E+02	8.40E+02	8.60E+02	6.49E+02	1.56E+03	9.53E+02	3.34E+02	3.35E+02	6.53E+02
	RANK	1	8	6	10	7	3	12	11	4	5	9	2
F10	AVG	3.78E+03	5.23E+03	5.40E+03	5.44E+03	7.88E+03	4.72E+03	9.03E+03	7.79E+03	5.36E+03	4.21E+03	5.60E+03	7.19E+03
	STD	9.45E+02	4.76E+02	8.87E+02	6.27E+02	1.35E+03	6.19E+02	5.05E+02	2.39E+02	1.07E+03	9.87E+02	1.69E+03	1.42E+03
	RANK	1	4	6	7	11	3	12	10	5	2	8	9
F11	AVG	1.18E+03	1.28E+03	1.29E+03	3.08E+03	4.39E+03	2.02E+03	7.45E+03	7.61E+03	1.23E+03	1.19E+03	1.64E+03	1.32E+03
	STD	2.74E+01	9.54E+01	4.37E+01	8.47E+02	1.28E+03	7.06E+02	2.39E+03	2.26E+03	5.38E+01	2.11E+01	2.40E+02	5.68E+01
	RANK	1	4	5	9	10	8	11	12	3	2	7	6
F12	AVG	5.30E+04	3.50E+07	6.34E+06	1.16E+09	8.80E+08	1.59E+08	1.97E+09	9.63E+09	7.09E+05	1.34E+06	7.61E+07	1.26E+06
	STD	2.40E+04	2.02E+07	3.12E+06	8.04E+08	6.96E+08	1.32E+08	1.00E+09	2.20E+09	6.30E+05	6.10E+05	3.44E+07	6.39E+05
	RANK	1	6	5	10	9	8	11	12	2	4	7	3
F13	AVG	9.61E+03	1.28E+05	8.01E+04	8.37E+07	8.06E+07	2.82E+05	1.11E+09	4.21E+09	1.35E+04	1.39E+04	3.47E+04	2.96E+04
	STD	9.36E+03	1.09E+05	2.74E+04	1.82E+08	1.17E+08	3.45E+05	9.76E+08	1.97E+09	9.71E+03	9.97E+03	1.68E+04	2.14E+04
	RANK	1	7	6	10	9	8	11	12	2	3	5	4
F14	AVG	1.51E+03	3.72E+04	1.01E+05	6.30E+05	2.10E+06	6.73E+05	2.20E+06	1.97E+06	1.76E+04	2.74E+04	9.35E+05	3.55E+04
	STD	1.43E+01	4.10E+04	1.22E+05	6.07E+05	2.01E+06	8.43E+05	1.02E+06	1.07E+06	1.03E+04	1.62E+04	5.98E+05	3.08E+04
	RANK	1	5	6	7	11	8	12	10	2	3	9	4
F15	AVG	1.66E+03	3.99E+04	7.87E+03	5.94E+05	2.13E+06	8.06E+05	1.91E+07	1.80E+08	3.28E+03	4.37E+03	1.53E+04	8.66E+03
	STD	5.75E+01	4.22E+04	3.15E+03	8.96E+05	1.52E+06	1.20E+06	1.72E+07	3.15E+08	1.82E+03	1.71E+03	1.64E+03	6.18E+03
	RANK	1	7	4	8	10	9	11	12	2	3	6	5
F16	AVG	2.42E+03	3.60E+03	2.69E+03	2.83E+03	2.90E+03	2.31E+03	4.26E+03	5.92E+03	2.62E+03	2.90E+03	3.36E+03	2.52E+03
	STD	1.54E+02	5.02E+02	2.48E+02	2.36E+02	1.59E+02	2.09E+02	5.35E+02	1.92E+03	2.31E+02	2.59E+02	5.29E+02	1.89E+02
	RANK	2	10	5	6	7	1	11	12	4	8	9	3
F17	AVG	1.92E+03	2.42E+03	2.39E+03	2.21E+03	2.43E+03	2.00E+03	2.81E+03	4.63E+03	2.17E+03	2.24E+03	2.54E+03	2.25E+03
	STD	1.35E+02	2.01E+02	2.56E+02	1.84E+02	3.02E+02	1.73E+02	3.47E+02	1.70E+03	1.88E+02	3.63E+02	2.89E+02	9.38E+01
	RANK	1	8	7	4	9	2	11	12	3	5	10	6
F18	AVG	2.63E+03	1.11E+05	6.50E+05	9.37E+05	1.85E+06	3.76E+06	1.73E+07	2.49E+07	1.32E+05	1.02E+05	3.84E+05	6.03E+05
	STD	3.73E+02	1.96E+04	8.70E+05	3.37E+05	1.28E+06	3.48E+06	1.93E+07	2.09E+07	5.74E+04	4.86E+04	6.45E+04	6.65E+05
	RANK	1	3	7	8	9	10	11	12	4	2	5	6
F19	AVG	1.96E+03	1.89E+06	2.49E+04	7.21E+05	2.33E+07	7.16E+05	1.93E+07	7.35E+07	5.17E+03	4.29E+03	1.05E+06	2.23E+04
	STD	1.41E+01	6.23E+05	3.06E+04	6.36E+05	4.64E+07	7.10E+05	9.56E+06	1.16E+08	7.65E+02	1.24E+03	1.16E+05	1.57E+04
	RANK	1	9	5	7	11	6	10	12	3	2	8	4
F20	AVG	2.36E+03	2.71E+03	2.57E+03	2.67E+03	2.40E+03	2.44E+03	3.09E+03	2.64E+03	2.45E+03	2.56E+03	2.68E+03	2.46E+03
	STD	1.16E+02	3.15E+02	1.70E+02	9.83E+01	1.63E+02	1.32E+02	2.17E+02	1.54E+02	1.40E+02	1.18E+02	8.42E+01	1.61E+02
	RANK	1	11	7	9	2	3	12	8	4	6	10	5
F21	AVG	2.43E+03	2.54E+03	2.48E+03	2.48E+03	2.51E+03	2.40E+03	2.67E+03	2.62E+03	2.43E+03	2.41E+03	2.54E+03	2.40E+03
	STD	5.99E+01	4.07E+01	3.59E+01	1.65E+01	6.49E+01	1.69E+01	3.71E+01	4.26E+01	2.94E+01	4.27E+01	4.50E+01	2.44E+01
	RANK	5	10	7	6	8	2	12	11	4	3	9	1
F22	AVG	2.30E+03	3.25E+03	7.34E+03	6.37E+03	5.62E+03	5.73E+03	7.80E+03	8.40E+03	2.86E+03	2.91E+03	4.03E+03	4.86E+03
	STD	9.67E-01	2.31E+03	2.58E+03	7.97E+02	1.90E+03	6.77E+02	1.99E+03	8.34E+02	1.36E+03	1.49E+03	1.02E+03	1.32E+03
	RANK	1	4	10	9	7	8	11	12	2	3	5	6
F23	AVG	2.73E+03	3.06E+03	2.92E+03	2.99E+03	2.91E+03	2.80E+03	3.29E+03	3.65E+03	2.80E+03	2.75E+03	2.95E+03	2.79E+03
	STD	2.21E+01	1.04E+02	4.86E+01	4.81E+01	6.52E+01	5.93E+01	3.94E+01	1.96E+02	2.86E+01	2.16E+01	5.50E+01	1.18E+01
	RANK	1	10	7	9	6	4	11	12	5	2	8	3
F24	AVG	2.92E+03	3.23E+03	3.05E+03	3.29E+03	3.13E+03	2.96E+03	3.37E+03	4.09E+03	2.94E+03	3.06E+03	3.05E+03	2.92E+03
	STD	2.90E+01	1.27E+02	9.94E+01	5.94E+01	2.58E+01	7.74E+01	1.06E+02	2.25E+02	4.54E+01	6.69E+01	4.54E+01	2.38E+01
	RANK	2	9	6	10	8	4	11	12	3	7	5	1
F25	AVG	2.89E+03	2.92E+03	2.98E+03	3.42E+03	3.18E+03	2.98E+03	3.42E+03	4.02E+03	2.90E+03	2.90E+03	3.01E+03	2.90E+03
	STD	7.07E+00	2.80E+01	1.11E+01	1.93E+02	1.32E+02	4.09E+01	1.36E+02	2.21E+02	1.23E+01	1.77E+01	1.45E+01	8.41E+00
	RANK	1	5	6	11	9	7	10	12	3	4	8	2
F26	AVG	3.91E+03	7.63E+03	5.09E+03	7.21E+03	5.94E+03	4.71E+03	7.47E+03	1.04E+04	3.96E+03	6.75E+03	7.38E+03	5.58E+03
	STD	8.91E+02	6.97E+02	1.74E+03	1.06E+03	7.01E+02	2.10E+02	1.00E+03	1.15E+03	1.90E+03	8.48E+02	7.79E+02	4.14E+02
	RANK	1	11	4	8	6	3	10	12	2	7	9	5
F27	AVG	3.20E+03	3.51E+03	3.29E+03	3.55E+03	3.34E+03	3.29E+03	3.59E+03	4.66E+03	3.25E+03	3.25E+03	3.49E+03	3.28E+03
	STD	2.33E-04	1.40E+02	5.04E+01	6.10E+01	8.20E+01	4.67E+01	7.18E+01	4.22E+02	1.55E+01	2.70E+01	1.40E+02	1.90E+01
	RANK	1	9	5	10	7	6	11	12	2	3	8	4
F28	AVG	3.18E+03	3.30E+03	3.33E+03	4.31E+03	3.74E+03	3.43E+03	4.31E+03	6.22E+03	3.19E+03	3.23E+03	3.57E+03	3.29E+03
	STD	3.84E+01	3.89E+01	6.23E+01	5.92E+02	1.87E+02	8.72E+01	5.72E+02	5.19E+02	2.49E+01	3.00E+01	1.27E+02	1.77E+01
	RANK	1	5	6	11	9	7	10	12	2	3	8	4
F29	AVG	3.80E+03	4.77E+03	3.85E+03	4.28E+03	4.18E+03	3.90E+03	5.92E+03	7.10E+03	3.89E+03	3.84E+03	4.94E+03	4.02E+03
	STD	5.26E+01	1.57E+02	3.02E+02	1.54E+02	2.15E+02	2.17E+02	9.79E+02	8.90E+02	1.39E+02	1.43E+02	3.88E+02	1.87E+02
	RANK	1	9	3	8	7	5	11	12	4	2	10	6
F30	AVG	7.36E+03	5.17E+06	3.43E+05	1.18E+07	3.30E+07	5.59E+06	2.37E+08	8.89E+08	1.77E+04	1.42E+04	5.49E+06	8.35E+04
	STD	1.46E+03	2.70E+06	7.96E+05	1.00E+07	2.25E+07	4.04E+06	2.26E+08	5.55E+08	1.23E+04	2.35E+03	2.04E+06	8.43E+04
	RANK	1	6	5	9	10	8	11	12	3	2	7	4
Average rank	Average rank	1.45	7.34	5.69	8.59	8.10	5.34	11.17	11.48	3.14	3.66	8.00	4.03
Final rank	Final rank	1	7	6	10	9	5	11	12	2	3	8	4

Table C3 Statistical analysis of AEHMO and other algorithms using CEC2017, 50D

F		AEHMO	HMO	MGTOA	AOA	IVY	GWO	HEOA	HOA	MHCSA	AOA-HHO	MHABC-PSO	ASG-HMO
F1	AVG	2.80E+06	9.52E+06	4.72E+09	6.29E+10	3.72E+10	9.79E+09	6.25E+10	9.32E+10	8.21E+03	7.80E+03	2.20E+10	8.28E+06
	STD	2.35E+06	3.48E+06	4.30E+09	1.03E+10	5.57E+09	2.43E+09	8.33E+09	1.12E+10	3.46E+03	2.89E+03	3.64E+09	3.46E+06
	RANK	3	5	6	11	9	7	10	12	2	1	8	4
F3	AVG	3.23E+04	6.62E+04	1.22E+05	1.29E+05	1.28E+05	1.45E+05	1.97E+05	1.59E+05	3.78E+04	4.19E+04	1.46E+05	1.57E+05
	STD	7.42E+03	1.47E+04	1.80E+04	1.28E+04	1.78E+04	2.73E+04	4.39E+03	1.89E+04	5.15E+03	6.32E+03	1.27E+04	9.52E+03
	RANK	1	4	5	7	6	8	12	11	2	3	9	10
F4	AVG	5.28E+02	6.93E+02	1.29E+03	8.86E+03	6.31E+03	1.89E+03	1.18E+04	2.75E+04	6.23E+02	5.36E+02	3.06E+03	6.62E+02
	STD	6.29E+01	3.75E+01	4.86E+02	2.33E+03	1.49E+03	4.06E+02	3.31E+03	3.54E+03	4.62E+01	9.52E+01	7.07E+02	6.80E+01
	RANK	1	5	6	10	9	7	11	12	3	2	8	4
F5	AVG	7.01E+02	9.05E+02	8.66E+02	9.84E+02	8.74E+02	7.12E+02	1.19E+03	1.10E+03	8.27E+02	8.27E+02	8.63E+02	8.12E+02
	STD	2.92E+01	5.29E+01	3.81E+01	3.24E+01	3.27E+01	2.61E+01	5.96E+01	3.92E+01	2.00E+01	3.98E+01	2.62E+01	2.53E+01
	RANK	1	9	7	10	8	2	12	11	4	5	6	3
F6	AVG	6.19E+02	6.67E+02	6.63E+02	6.63E+02	6.53E+02	6.21E+02	6.98E+02	6.83E+02	6.43E+02	6.46E+02	6.68E+02	6.44E+02
	STD	4.15E+00	4.33E+00	4.28E+00	3.62E+00	3.89E+00	4.97E+00	5.80E+00	8.50E+00	6.81E+00	6.76E+00	4.19E+00	1.60E+01
	RANK	1	9	8	7	6	2	12	11	3	5	10	4
F7	AVG	1.04E+03	1.75E+03	1.47E+03	1.70E+03	1.52E+03	1.12E+03	2.03E+03	1.87E+03	1.27E+03	1.30E+03	1.56E+03	1.31E+03
	STD	4.55E+01	1.15E+02	2.31E+02	3.93E+01	6.57E+01	8.59E+01	4.62E+01	5.58E+01	1.01E+02	1.65E+02	5.10E+01	1.16E+02
	RANK	1	10	6	9	7	2	12	11	3	4	8	5
F8	AVG	1.11E+03	1.18E+03	1.20E+03	1.27E+03	1.20E+03	1.02E+03	1.57E+03	1.43E+03	1.11E+03	1.14E+03	1.18E+03	1.00E+03
	STD	6.51E+01	4.23E+01	3.82E+01	5.60E+01	3.82E+01	2.68E+01	4.32E+01	5.39E+01	1.80E+01	3.79E+01	3.06E+01	3.87E+01
	RANK	3	6	8	10	9	2	12	11	4	5	7	1
F9	AVG	4.99E+03	1.66E+04	1.92E+04	1.85E+04	2.01E+04	1.07E+04	3.75E+04	3.11E+04	1.05E+04	1.06E+04	2.01E+04	2.26E+04
	STD	3.12E+03	9.75E+02	5.56E+03	1.94E+03	5.65E+03	5.93E+03	2.56E+03	2.29E+03	1.02E+03	1.04E+03	7.48E+03	5.42E+03
	RANK	1	5	7	6	8	4	12	11	2	3	9	10
F10	AVG	5.94E+03	9.30E+03	1.14E+04	9.14E+03	1.20E+04	7.46E+03	1.44E+04	1.40E+04	7.13E+03	8.08E+03	8.00E+03	1.18E+04
	STD	4.50E+02	9.17E+02	1.57E+03	1.26E+03	2.12E+03	1.28E+03	1.09E+03	8.82E+02	1.37E+03	8.60E+02	6.10E+02	3.24E+03
	RANK	1	7	8	6	10	3	12	11	2	5	4	9
F11	AVG	1.36E+03	1.52E+03	1.04E+06	1.05E+04	7.28E+03	6.75E+03	1.68E+04	2.09E+04	1.30E+03	1.27E+03	3.63E+03	1.84E+03
	STD	5.15E+01	7.61E+01	1.60E+06	2.26E+03	2.78E+03	2.45E+03	1.74E+03	2.25E+03	5.02E+01	4.86E+01	5.09E+02	1.85E+02
	RANK	3	4	12	9	8	7	10	11	2	1	6	5
F12	AVG	4.93E+06	1.64E+08	1.56E+09	1.59E+10	8.38E+09	1.17E+09	1.98E+10	6.44E+10	4.49E+06	4.23E+06	1.58E+09	3.21E+07
	STD	4.36E+06	1.09E+08	2.36E+09	1.41E+10	2.22E+09	1.44E+09	4.85E+09	1.79E+10	1.44E+06	1.80E+06	1.06E+09	3.02E+07
	RANK	3	5	7	10	9	6	11	12	2	1	8	4
F13	AVG	6.03E+03	8.31E+04	8.36E+06	7.43E+09	1.74E+09	1.25E+08	3.43E+09	4.22E+10	1.17E+04	1.37E+04	8.79E+05	8.94E+04
	STD	2.44E+03	5.72E+04	1.28E+07	5.96E+09	2.04E+09	1.38E+08	3.08E+09	1.04E+10	5.66E+03	9.23E+03	2.03E+06	5.13E+04
	RANK	1	4	7	11	9	8	10	12	2	3	6	5
F14	AVG	1.79E+03	1.80E+05	9.15E+05	5.02E+06	3.91E+06	2.07E+06	1.97E+07	8.84E+07	1.12E+05	4.65E+04	1.54E+06	2.63E+05
	STD	6.01E+01	1.55E+05	5.30E+05	3.53E+06	4.33E+06	1.09E+06	1.25E+07	6.01E+07	9.55E+04	3.03E+04	1.12E+06	2.04E+05
	RANK	1	4	6	10	9	8	11	12	3	2	7	5
F15	AVG	1.16E+04	3.50E+04	4.59E+04	8.96E+08	5.77E+07	7.02E+07	1.63E+09	6.46E+09	1.31E+04	1.75E+04	3.20E+04	1.95E+04
	STD	8.45E+03	1.36E+04	2.41E+04	6.52E+08	5.57E+07	1.66E+08	6.97E+08	2.13E+09	6.34E+03	4.83E+03	6.41E+03	1.82E+04
	RANK	1	6	7	10	8	9	11	12	2	3	5	4
F16	AVG	2.72E+03	4.91E+03	3.31E+03	4.17E+03	4.15E+03	3.24E+03	7.16E+03	8.44E+03	3.13E+03	2.93E+03	3.58E+03	3.49E+03
	STD	1.73E+02	5.55E+02	4.70E+02	3.30E+02	5.43E+02	3.62E+02	7.48E+02	1.14E+03	2.54E+02	1.92E+02	2.32E+02	3.43E+02
	RANK	1	10	5	9	8	4	11	12	3	2	7	6
F17	AVG	3.01E+03	3.87E+03	3.71E+03	3.45E+03	3.48E+03	2.98E+03	4.71E+03	6.55E+03	3.13E+03	3.11E+03	3.52E+03	3.14E+03
	STD	1.91E+02	2.38E+02	6.07E+02	3.66E+02	3.56E+02	3.56E+02	3.29E+02	1.01E+03	3.62E+02	1.52E+02	3.84E+02	4.13E+02
	RANK	2	10	9	6	7	1	11	12	4	3	8	5
F18	AVG	8.12E+04	2.24E+06	3.08E+06	5.95E+06	1.32E+07	1.32E+07	5.94E+07	1.12E+08	5.38E+05	5.00E+05	2.74E+06	3.30E+06
	STD	7.99E+04	1.34E+06	1.29E+06	4.62E+06	8.07E+06	2.33E+07	3.02E+07	2.62E+07	2.29E+05	9.16E+04	1.07E+06	1.48E+06
	RANK	1	4	6	8	10	9	11	12	3	2	5	7
F19	AVG	1.73E+04	1.03E+06	2.01E+05	1.99E+08	3.41E+08	1.13E+07	3.48E+08	2.19E+09	2.61E+04	3.09E+04	4.21E+05	3.02E+04
	STD	8.94E+03	1.02E+06	3.17E+05	2.24E+08	4.70E+08	1.76E+07	1.78E+08	1.23E+09	1.41E+04	1.23E+04	2.44E+04	1.14E+04
	RANK	1	7	5	9	10	8	11	12	2	4	6	3
F20	AVG	2.98E+03	3.40E+03	3.23E+03	3.04E+03	3.42E+03	2.98E+03	4.23E+03	3.65E+03	2.96E+03	3.29E+03	3.24E+03	2.97E+03
	STD	8.34E+01	2.49E+02	1.68E+02	1.99E+02	3.53E+02	1.18E+02	3.91E+02	2.77E+02	1.86E+02	4.47E+02	1.59E+02	1.47E+02
	RANK	4	9	6	5	10	3	12	11	1	8	7	2
F21	AVG	2.50E+03	2.76E+03	2.65E+03	2.83E+03	2.75E+03	2.51E+03	3.11E+03	3.01E+03	2.58E+03	2.57E+03	2.75E+03	2.53E+03
	STD	2.85E+01	9.12E+01	7.05E+01	2.90E+01	6.20E+01	3.44E+01	9.54E+01	2.09E+01	5.67E+01	5.22E+01	6.53E+01	3.85E+01
	RANK	1	9	6	10	8	2	12	11	5	4	7	3
F22	AVG	7.86E+03	1.05E+04	1.49E+04	1.24E+04	1.35E+04	9.14E+03	1.64E+04	1.56E+04	1.35E+04	9.44E+03	1.17E+04	8.78E+03
	STD	1.06E+03	1.29E+03	1.28E+03	7.33E+02	2.56E+03	1.05E+03	7.89E+02	6.14E+02	2.66E+03	1.23E+03	4.66E+02	1.09E+03
	RANK	1	5	10	7	8	3	12	11	9	4	6	2
F23	AVG	3.15E+03	3.53E+03	3.36E+03	3.60E+03	3.29E+03	3.00E+03	4.04E+03	4.64E+03	3.00E+03	3.05E+03	3.40E+03	3.08E+03
	STD	1.51E+02	1.17E+02	8.29E+01	1.26E+02	7.46E+01	3.80E+01	2.36E+02	1.80E+02	8.39E+01	5.84E+01	7.56E+01	5.25E+01
	RANK	5	9	7	10	6	2	11	12	1	3	8	4
F24	AVG	3.08E+03	3.81E+03	3.71E+03	4.03E+03	3.55E+03	3.20E+03	4.12E+03	5.50E+03	3.09E+03	3.35E+03	3.42E+03	3.20E+03
	STD	2.15E+01	1.32E+02	3.29E+02	7.55E+01	7.08E+01	1.41E+02	2.55E+02	1.23E+02	4.75E+01	1.49E+02	1.04E+02	8.56E+01
	RANK	1	9	8	10	7	3	11	12	2	5	6	4
F25	AVG	3.08E+03	3.16E+03	3.61E+03	7.32E+03	5.83E+03	3.86E+03	8.37E+03	1.27E+04	3.14E+03	3.09E+03	4.37E+03	3.16E+03
	STD	4.73E+01	8.94E+01	2.57E+02	1.16E+03	3.76E+02	2.78E+02	1.50E+03	1.46E+03	5.92E+01	3.71E+01	2.68E+02	6.61E+01
	RANK	1	5	6	10	9	7	11	12	3	2	8	4
F26	AVG	6.75E+03	1.21E+04	8.77E+03	1.31E+04	9.26E+03	9.99E+03	1.47E+04	1.68E+04	9.73E+03	8.84E+03	1.17E+04	7.66E+03
	STD	5.50E+02	1.33E+03	3.20E+03	1.06E+03	1.01E+03	3.20E+03	9.91E+02	9.91E+02	3.60E+03	2.57E+03	1.49E+03	7.11E+02
	RANK	1	9	3	10	5	7	11	12	6	4	8	2
F27	AVG	3.20E+03	5.19E+03	3.75E+03	4.58E+03	4.27E+03	3.69E+03	5.55E+03	7.24E+03	3.53E+03	3.63E+03	4.33E+03	3.72E+03
	STD	2.50E-04	9.33E+02	2.15E+02	2.75E+02	1.98E+02	6.58E+01	5.90E+02	5.56E+02	1.37E+02	1.45E+02	2.97E+02	9.10E+01
	RANK	1	10	6	9	7	4	11	12	2	3	8	5
F28	AVG	3.51E+03	3.40E+03	4.17E+03	7.21E+03	5.90E+03	4.76E+03	8.66E+03	1.18E+04	3.31E+03	3.31E+03	5.53E+03	3.68E+03
	STD	5.97E+01	3.95E+01	4.53E+02	9.82E+02	9.08E+02	1.12E+02	5.93E+02	1.16E+03	1.02E+01	1.35E+01	5.52E+02	1.46E+02
	RANK	4	3	6	10	9	7	11	12	2	1	8	5
F29	AVG	4.51E+03	6.31E+03	4.68E+03	7.93E+03	5.84E+03	4.69E+03	1.40E+04	5.49E+04	4.60E+03	4.95E+03	7.41E+03	4.98E+03
	STD	4.44E+02	5.16E+02	8.81E+02	1.05E+03	3.78E+02	3.90E+02	7.50E+03	1.77E+04	5.22E+02	3.10E+02	5.33E+02	2.97E+02
	RANK	1	8	3	10	7	4	11	12	2	5	9	6
F30	AVG	1.26E+06	1.01E+08	1.78E+06	4.01E+08	4.06E+08	1.34E+08	8.00E+08	5.49E+09	9.84E+05	1.08E+06	1.81E+08	7.14E+06
	STD	2.57E+05	2.58E+07	2.79E+06	2.87E+08	2.24E+08	2.57E+07	3.02E+08	1.92E+09	1.67E+05	1.27E+05	2.35E+07	4.15E+06
	RANK	3	6	4	9	10	7	11	12	1	2	8	5
Average rank	Average rank	1.72	6.76	6.55	8.90	8.14	5.03	11.24	11.62	2.83	3.28	7.24	4.69
Final rank	Final rank	1	7	6	10	9	5	11	12	2	3	8	4

Table C4 Statistical analysis of AEHMO and other algorithms using CEC2017, 100D

F		AEHMO	HMO	MGTOA	AOA	IVY	GWO	HEOA	HOA	MHCSA	AOA-HHO	MHABC-PSO	ASG-HMO
F1	AVG	2.87E+09	6.71E+08	5.30E+10	1.88E+11	1.33E+11	5.17E+10	2.05E+11	2.43E+11	6.37E+04	8.95E+04	1.08E+11	7.39E+08
	STD	1.04E+09	1.02E+08	7.30E+09	1.36E+10	1.76E+10	9.17E+09	1.47E+10	9.79E+09	2.13E+04	6.08E+04	9.20E+09	2.92E+08
	RANK	5	3	7	10	9	6	11	12	1	2	8	4
F3	AVG	2.44E+05	3.28E+05	3.09E+05	3.20E+05	3.38E+05	4.47E+05	3.64E+05	3.20E+05	2.51E+05	2.46E+05	3.26E+05	3.35E+05
	STD	2.12E+04	1.44E+04	4.31E+04	1.49E+04	3.11E+04	6.38E+04	1.09E+04	1.19E+04	2.35E+04	9.50E+03	4.55E+03	3.32E+04
	RANK	1	8	4	6	10	12	11	5	3	2	7	9
F4	AVG	7.35E+02	1.22E+03	8.04E+03	3.66E+04	2.04E+04	5.76E+03	4.16E+04	8.44E+04	7.47E+02	1.47E+03	1.58E+04	1.30E+03
	STD	3.97E+01	9.20E+01	3.11E+03	7.20E+03	5.26E+03	9.96E+02	6.96E+03	1.27E+04	5.73E+01	1.30E+02	1.49E+03	8.99E+01
	RANK	1	3	7	10	9	6	11	12	2	5	8	4
F5	AVG	1.11E+03	1.42E+03	1.51E+03	1.76E+03	1.58E+03	1.19E+03	2.10E+03	1.96E+03	1.31E+03	1.31E+03	1.38E+03	1.21E+03
	STD	6.28E+01	6.29E+01	9.12E+01	2.97E+01	8.66E+01	6.80E+01	6.53E+01	5.82E+01	2.30E+01	5.70E+01	2.19E+01	5.51E+01
	RANK	1	7	8	10	9	2	12	11	5	4	6	3
F6	AVG	6.43E+02	6.77E+02	6.71E+02	6.82E+02	6.73E+02	6.56E+02	7.15E+02	6.98E+02	6.59E+02	6.59E+02	6.74E+02	6.45E+02
	STD	4.66E+00	3.00E+00	6.95E+00	3.76E+00	5.60E+00	5.05E+00	3.21E+00	2.21E+00	2.51E+00	1.75E+00	2.54E+00	2.70E+00
	RANK	1	9	6	10	7	3	12	11	5	4	8	2
F7	AVG	1.87E+03	3.48E+03	3.26E+03	3.42E+03	2.93E+03	2.14E+03	4.03E+03	3.76E+03	2.63E+03	2.56E+03	3.14E+03	2.73E+03
	STD	1.41E+02	2.07E+02	5.92E+02	1.28E+02	9.43E+01	1.90E+02	4.20E+01	1.11E+02	1.28E+02	2.71E+02	1.07E+02	1.13E+02
	RANK	1	10	8	9	6	2	12	11	4	3	7	5
F8	AVG	1.53E+03	1.96E+03	1.97E+03	2.13E+03	1.87E+03	1.68E+03	2.62E+03	2.45E+03	1.68E+03	1.72E+03	1.84E+03	1.37E+03
	STD	4.51E+01	7.20E+01	8.79E+01	4.65E+01	1.36E+02	7.40E+01	1.11E+02	5.46E+01	9.20E+01	5.44E+01	7.31E+01	5.89E+01
	RANK	2	8	9	10	7	3	12	11	4	5	6	1
F9	AVG	2.02E+04	4.08E+04	5.27E+04	4.65E+04	5.65E+04	3.37E+04	8.10E+04	6.48E+04	2.33E+04	2.26E+04	3.41E+04	6.23E+04
	STD	2.32E+03	3.15E+03	7.30E+03	2.62E+03	1.35E+04	1.15E+04	4.62E+03	5.39E+03	5.08E+02	7.66E+02	2.36E+03	7.71E+03
	RANK	1	6	8	7	9	4	12	11	3	2	5	10
F10	AVG	2.41E+04	1.94E+04	2.54E+04	2.44E+04	2.36E+04	1.72E+04	3.19E+04	2.93E+04	1.50E+04	1.61E+04	1.81E+04	2.80E+04
	STD	5.96E+03	1.78E+03	2.72E+03	1.18E+03	4.32E+03	1.33E+03	8.32E+02	1.10E+03	1.01E+03	1.41E+03	1.25E+03	5.58E+03
	RANK	7	5	9	8	6	3	12	11	1	2	4	10
F11	AVG	1.46E+04	2.63E+04	1.68E+05	9.92E+04	1.05E+05	7.76E+04	2.12E+05	1.84E+05	2.45E+03	2.59E+03	1.08E+05	9.24E+04
	STD	4.68E+03	5.75E+03	4.10E+04	1.47E+04	9.99E+03	1.93E+04	2.14E+04	2.63E+04	1.25E+02	2.76E+02	1.64E+04	2.97E+04
	RANK	3	4	10	7	8	5	12	11	1	2	9	6
F12	AVG	2.61E+07	8.46E+08	1.66E+10	9.24E+10	5.88E+10	1.11E+10	9.53E+10	1.66E+11	2.78E+07	2.78E+08	3.59E+10	1.91E+08
	STD	7.10E+06	3.51E+08	1.21E+10	8.93E+09	8.18E+09	8.45E+09	1.77E+10	2.09E+10	1.42E+07	1.58E+08	7.04E+09	1.47E+08
	RANK	1	5	7	10	9	6	11	12	2	4	8	3
F13	AVG	1.20E+04	1.81E+05	9.33E+08	1.66E+10	8.82E+09	1.17E+09	1.57E+10	3.94E+10	1.18E+04	2.27E+04	2.35E+09	5.10E+05
	STD	4.30E+03	9.50E+04	1.32E+09	4.61E+09	2.90E+09	6.38E+08	2.85E+09	6.42E+09	2.15E+03	3.57E+03	4.15E+08	3.80E+05
	RANK	2	4	6	11	9	7	10	12	1	3	8	5
F14	AVG	2.48E+05	1.38E+06	5.77E+06	1.53E+07	1.00E+07	7.90E+06	2.14E+07	4.76E+07	4.57E+05	3.54E+05	5.80E+06	6.30E+06
	STD	1.48E+05	5.66E+05	1.94E+06	4.15E+06	3.40E+06	4.00E+06	4.33E+06	2.00E+07	1.20E+05	7.64E+04	1.93E+06	2.97E+06
	RANK	1	4	5	10	9	8	11	12	3	2	6	7
F15	AVG	5.04E+03	4.18E+04	1.72E+07	8.15E+09	2.54E+09	2.61E+08	6.03E+09	1.78E+10	5.22E+03	5.35E+03	2.87E+05	2.40E+05
	STD	4.36E+02	1.13E+04	9.25E+06	4.81E+09	1.27E+09	3.02E+08	1.32E+09	3.51E+09	2.12E+03	1.13E+03	3.34E+05	3.04E+05
	RANK	1	4	7	11	9	8	10	12	2	3	6	5
F16	AVG	5.42E+03	9.20E+03	8.35E+03	1.13E+04	1.03E+04	7.14E+03	1.68E+04	2.05E+04	5.88E+03	5.84E+03	1.01E+04	5.84E+03
	STD	1.34E+03	1.47E+03	8.28E+02	9.28E+02	8.99E+02	1.16E+03	8.92E+02	1.94E+03	6.46E+02	5.00E+02	7.77E+02	1.07E+03
	RANK	1	7	6	10	9	5	11	12	4	3	8	2
F17	AVG	4.92E+03	6.30E+03	6.80E+03	2.87E+04	3.23E+04	5.38E+03	4.46E+05	2.44E+06	5.08E+03	5.11E+03	7.39E+03	5.55E+03
	STD	6.79E+02	6.71E+02	9.14E+02	2.05E+04	4.13E+04	5.16E+02	5.51E+05	1.85E+06	2.67E+02	6.33E+02	5.12E+02	3.11E+02
	RANK	1	6	7	9	10	4	11	12	2	3	8	5
F18	AVG	6.35E+05	1.48E+06	7.86E+07	1.15E+07	1.64E+07	9.29E+06	4.60E+07	1.22E+08	9.04E+05	8.36E+05	2.95E+06	5.56E+06
	STD	2.76E+05	6.08E+05	3.70E+07	5.22E+06	8.57E+06	7.74E+06	6.07E+06	5.06E+07	3.17E+05	3.55E+05	1.22E+06	1.67E+06
	RANK	1	4	11	8	9	7	10	12	3	2	5	6
F19	AVG	6.56E+03	1.08E+07	4.62E+07	7.04E+09	1.17E+09	2.45E+08	8.00E+09	2.06E+10	8.35E+03	8.67E+03	1.85E+06	4.04E+05
	STD	4.50E+03	6.63E+06	9.06E+07	5.14E+09	5.49E+08	2.05E+08	3.87E+09	5.23E+09	5.69E+03	2.74E+03	1.59E+05	4.36E+05
	RANK	1	6	7	10	9	8	11	12	2	3	5	4
F20	AVG	5.77E+03	6.15E+03	9.18E+03	6.20E+03	6.33E+03	6.12E+03	7.73E+03	7.52E+03	5.12E+03	5.16E+03	6.48E+03	6.08E+03
	STD	1.01E+03	6.54E+02	1.74E+03	3.39E+02	1.33E+03	1.70E+03	2.46E+02	2.69E+02	4.50E+02	4.19E+02	8.33E+02	1.31E+03
	RANK	3	6	12	7	8	5	11	10	1	2	9	4
F21	AVG	2.96E+03	3.62E+03	3.67E+03	3.77E+03	3.47E+03	3.05E+03	4.32E+03	4.57E+03	2.97E+03	2.90E+03	3.40E+03	3.15E+03
	STD	1.08E+02	1.46E+02	2.31E+02	6.10E+01	8.84E+01	6.30E+01	1.97E+02	1.58E+02	7.18E+01	8.81E+01	7.54E+01	1.22E+02
	RANK	2	8	9	10	7	4	11	12	3	1	6	5
F22	AVG	1.94E+04	2.37E+04	3.12E+04	2.75E+04	2.68E+04	2.02E+04	3.48E+04	3.20E+04	2.01E+04	2.46E+04	2.28E+04	2.36E+04
	STD	1.56E+03	1.84E+03	3.34E+03	1.55E+03	3.76E+03	1.94E+03	6.69E+02	9.89E+02	1.73E+03	6.98E+03	9.98E+02	7.01E+03
	RANK	1	6	10	9	8	3	12	11	2	7	4	5
F23	AVG	3.90E+03	4.92E+03	8.06E+03	4.87E+03	4.47E+03	3.69E+03	5.96E+03	7.93E+03	3.40E+03	3.33E+03	4.22E+03	3.57E+03
	STD	1.50E+02	2.42E+02	8.95E+02	2.94E+02	1.41E+02	1.24E+02	5.19E+02	3.63E+02	5.17E+01	8.03E+01	2.52E+02	5.22E+01
	RANK	5	9	12	8	7	4	10	11	2	1	6	3
F24	AVG	3.95E+03	7.51E+03	6.29E+03	7.44E+03	5.96E+03	4.40E+03	7.52E+03	1.27E+04	4.00E+03	5.45E+03	5.64E+03	4.61E+03
	STD	9.94E+01	9.26E+02	6.11E+02	3.40E+02	1.43E+02	2.41E+02	5.15E+02	1.97E+02	6.26E+01	5.05E+02	2.05E+02	1.89E+02
	RANK	1	10	8	9	7	3	11	12	2	5	6	4
F25	AVG	3.29E+03	3.86E+03	7.49E+03	1.60E+04	1.32E+04	6.82E+03	1.78E+04	2.48E+04	4.14E+03	3.32E+03	9.56E+03	4.09E+03
	STD	9.27E+01	9.28E+01	1.27E+03	1.37E+03	2.07E+03	3.60E+02	1.36E+03	2.75E+03	2.02E+02	7.24E+01	5.85E+02	2.45E+02
	RANK	1	3	7	10	9	6	11	12	5	2	8	4
F26	AVG	1.63E+04	3.05E+04	2.75E+04	3.94E+04	2.69E+04	2.84E+04	4.37E+04	5.28E+04	2.25E+04	2.55E+04	3.20E+04	1.88E+04
	STD	1.42E+03	8.61E+02	2.70E+03	2.02E+03	1.95E+03	1.45E+03	3.87E+03	3.45E+03	2.49E+03	1.55E+03	1.45E+03	1.64E+03
	RANK	1	8	6	10	5	7	11	12	3	4	9	2
F27	AVG	3.79E+03	5.68E+03	4.84E+03	6.90E+03	5.41E+03	4.23E+03	8.93E+03	1.44E+04	3.20E+03	3.82E+03	7.08E+03	4.12E+03
	STD	7.69E+01	3.77E+02	2.21E+02	7.40E+02	2.59E+02	2.20E+02	1.21E+03	1.40E+03	2.74E-04	9.78E+01	4.05E+02	1.40E+02
	RANK	2	8	6	9	7	5	11	12	1	3	10	4
F28	AVG	4.45E+03	4.01E+03	1.51E+04	2.33E+04	1.76E+04	8.90E+03	2.56E+04	3.24E+04	3.46E+03	3.46E+03	1.09E+04	5.68E+03
	STD	2.34E+02	1.36E+02	9.40E+03	1.16E+03	2.09E+03	8.36E+02	1.58E+03	3.18E+03	3.93E+01	2.86E+01	8.17E+02	1.23E+03
	RANK	4	3	8	10	9	6	11	12	2	1	7	5
F29	AVG	7.72E+03	1.37E+04	9.12E+03	3.52E+04	1.57E+04	8.63E+03	4.64E+04	2.51E+05	8.73E+03	7.57E+03	1.62E+04	8.37E+03
	STD	5.90E+02	1.66E+03	2.18E+03	3.79E+04	3.10E+03	8.68E+02	3.25E+04	8.81E+04	6.62E+02	3.62E+02	1.50E+03	2.93E+02
	RANK	2	7	6	10	8	4	11	12	5	1	9	3
F30	AVG	2.58E+06	1.65E+08	1.15E+09	1.51E+10	5.35E+09	1.24E+09	1.70E+10	3.44E+10	2.89E+05	2.27E+05	5.04E+09	5.15E+06
	STD	1.51E+06	1.02E+08	2.15E+09	3.86E+09	3.59E+09	8.03E+08	3.55E+09	4.35E+09	1.29E+05	1.10E+05	2.24E+09	2.99E+06
	RANK	3	5	6	10	9	7	11	12	2	1	8	4
Average rank	Average rank	1.97	6.07	7.66	9.24	8.17	5.28	11.14	11.38	2.62	2.83	7.03	4.62
Final rank	Final rank	1	6	8	10	9	5	11	12	2	3	7	4

Appendix D: Convergence curves

The convergence behavior of AEHMO for unimodal and multimodal functions is shown to be consistently strong in both exploration efficiency and exploitation precision over the 30,000 function evaluations as presented in Figs. D1-D4. In unimodal functions F1 and F3, AEHMO exhibits rapid early decrease in fitness in the first 5000 FEs, which reflects intensive exploration in the early stage with the help of the DDS strategy that helps the algorithm to find promising basins quickly. Across all tested dimensions (10D, 30D, 50D, 100D), AEHMO shows the steepest convergence slope in F1 with a transition from exploration to exploitation around 10,000 FEs via dynamic adaptation of control parameters to shift the search focus. However, in F3 in higher dimensions, especially 30D and 50D, AEHMO exhibits a slower, more gradual convergence pattern after 15,000 FEs, suggesting that the function requires longer exploitation phases, where MDMS will improve the solutions by selective mutations. In 100D F1, there is more oscillation in the convergence curve than for lower dimensions, which indicates that fixed parameter settings may be less effective in sustaining steady exploitation in high-dimensional smooth landscapes. For multimodal functions F4-F10, AEHMO demonstrates consistently favorable exploration–exploitation behavior with especially interesting performance in F4, F6, F7 and F9 where the AEHMO curve falls below all competitors by 8000-12000 FEs for all dimensions. The convergence pattern in these functions illustrates a two-phase behavior: intensive exploration phase (0-7000 FEs) where AEHMO samples rapidly different regions by chaotic drift movements, followed by intensive exploitation phase (7,000-30,000 FEs) where

the curve flattens indicating solution refinement by Gaussian mutations from MDMS. In F5 in 30D, 50D and 100D, AEHMO has competitive but not dominant behavior, where GWO and ASG-HMO have a slightly better final convergence after 30,000 FEs, which may be because of the regular periodic structure of the function, where AEHMO continues to explore widely for a longer time than necessary and therefore delays the transfer to fine-grained exploitation. The F8 and F10 convergence curves in 50D and 100D exhibit step-like descent curves of AEHMO indicating discrete improvements of the algorithm leaving successive local basins by CRL's chaotic opposition mechanism, whereas, competitor algorithms exhibit smoother, but slower convergence curves.

For hybrid functions, AEHMO has exceptional convergence behavior showing sustained descent over the whole 30,000 FEs, which is indicative of the ability for AEHMO to maintain momentum in the search in complex compositional landscapes. In F12, F13, F14, F15, F18 and F19 in all dimensions, AEHMO's optimization path is consistently lower than any of the competitors in the entire optimization process and this is especially dramatic after 15,000 FEs where the competitor algorithms plateau and AEHMO continues to improve steadily. This behavior indicates that the CRL strategy was able to avoid premature convergence by introducing structured randomness to find new promising regions even in late iterations. The descent behavior reveals that AEHMO finds a good balance between exploration and exploitation using a three-step approach: global search supported by DDS for 0-8000 FEs, transient refinement supported by DDS for 8000-18000 FEs where the adaptive parameters are gradually changing the search intensity and fine-tuning for 18000-30000 FEs where MDMS uses selective mutations to the elite solutions. In F16 and F17 for 30D and 50D, AEHMO's fitness trends show stable monotonic decreasing without the stagnation that is clearly shown in competitor algorithms around 20,000 FEs, which verifies the success of the adaptive parameter mechanism to avoid the exploration-exploitation imbalance. However, in F11 across 50D and 100D AEHMO's progress profile exhibits slight performance degradation compared to MHCSA and AOA-HHO after 20,000 FEs with the convergence curves converging to similar final values indicating that some hybrid composite functions form landscapes in which memory-based recall mechanisms offer diminishing returns in high dimensions. The F20 convergence behavior in 100D shows the performance to be competitive rather than dominant with multiple algorithms showing similar convergence behaviors suggesting the expanded rotated hybrid functions in 100 dimensions introduce challenges to the search in which the benefits of AEHMO are less pronounced.

The composite functions exhibit AEHMO's most remarkable convergence properties, where the algorithm keeps improving steadily over the entire optimization budget, while most of the competitors keep stagnating after 15,000-20,000 FEs. In F22, F24, F25, F26, F27, F29, F30 in 10D, 30D and 50D, the fitness trends of AEHMO show an uninterrupted downward trend without any apparent plateaus, indicating that the synergistic combination of DDS, CRL, and MDMS sustains improvement throughout the evaluation budget. The convergence behavior is characterized particularly well in F27 in all dimensions where AEHMO has been able to achieve near-vertical initial descent in the 0-3,000 FEs followed by sustained steady improvement showing strong performance in finding and exploiting optimal regions in a short time. In F30 for 30D and 50D, the trajectories reveal AEHMO maintaining the steepest slope of descent throughout the 30,000 FE budget with AEHMO and the competitors having a larger and larger gap, suggesting that the diversity maintenance mechanisms of AEHMO prevents the convergence stagnation of other methods. The F21, F23 and F24 convergence patterns in 30D reveal a smooth and constant descent without the oscillations we see with competitor algorithms, that is, AEHMO handles population diversity in a stable manner thanks to balanced mutation strategies that do not prematurely lead to convergence and exploration. However, in 100D composite functions, particularly F23, F28 and F20, AEHMO's convergence curves show a more gradual convergence than those for the lower dimensions with other competing algorithms, such as MHCSA and AOA-HHO, showing similar or slightly better convergence curves at 20,000 FEs, implying that high-dimensional compositional spaces are difficult to handle for AEHMO's parameter adaptation mechanisms.

The failure patterns of convergence indicate some systematic limitations in the search behavior of AEHMO under certain dimensional and functional circumstances. In F1 at 100D we can see that AEHMO's trajectory is much slower than MHCSA and AOA-HHO, where you can see the visible occurrence of

stagnation around 15,000 FEs where the curve flattens prematurely and suggests that the adaptive parameters go into exploitation too aggressively in high-dimensional unimodal spaces, reducing population diversity before well sampling the expanded search space. The oscillating convergence pattern in 100D F1 between 10,000-25,000 FEs indicates that AEHMO has difficulty in sustaining momentum of the search direction, the algorithm is oscillating between exploration and exploitation without stable convergence, which is probably due to insufficient stability of the fixed chaotic mapping intensity for 100 dimensional smooth landscapes. In F5 in 30D, 50D, and 100D, the progress profiles have shown that AEHMO has a more extensive exploration time than the competitors, the AEHMO curve has a slower declining rate in 5,000-20,000 FEs compared to GWO and ASG-HMO, which indicates that the local optima of the function are distributed regularly, triggering too much DDS activity, delaying the convergence to the global optimum. The F10 convergence failure in 100D is shown where the AEHMO's curve shows dramatic performance degradation with AEHMO's curve descending in a competitive way during 8,000 FEs but exhibits erratic behavior with multiple plateaus and oscillations during 8,000-30,000 FEs, suggesting that the 100-dimensional rotated function creates deceptive landscape features that disorganize the ability of adaptive parameter mechanism to regulate the balance of exploration and exploitation. In composite function F23 at 100D, the trajectory of AEHMO has premature flattening around 12000 FEs while the convergence curves of the competitor algorithms keep descending steadily, which reveals that some compositional functions in high dimension cause the memory-based recall mechanism to concentrate on suboptimal regions of the landscape several times, causing search bias preventing the discovery of better solutions despite the continuous evaluations of the function. The F28 and F20 convergence patterns in 100D indicate that AEHMO sometimes exhibits slower convergence speeds than the original HMO for 20,000 FEs with the red curve still above HMO's curve in the final iterations, indicating the existence of paradoxical scenarios where the simpler fixed parameter approach achieves better late stage exploitation by avoiding the computational overhead and possible over-adaptation of AEHMO's dynamic mechanisms in certain high-dimensional compositional structures, confirming the need to design current parameter adaptation strategies with mechanisms for dimensional scaling to maintain their effectiveness in high-dimensional settings.

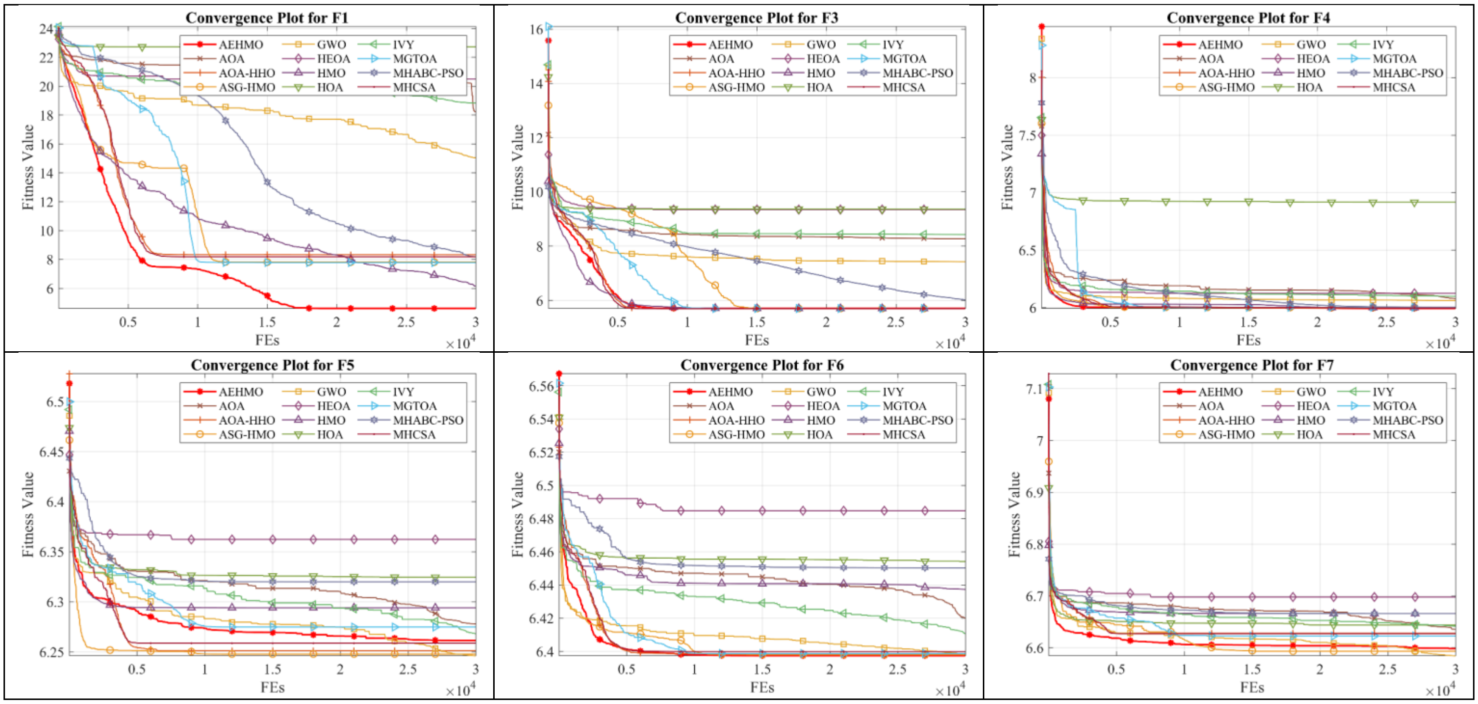

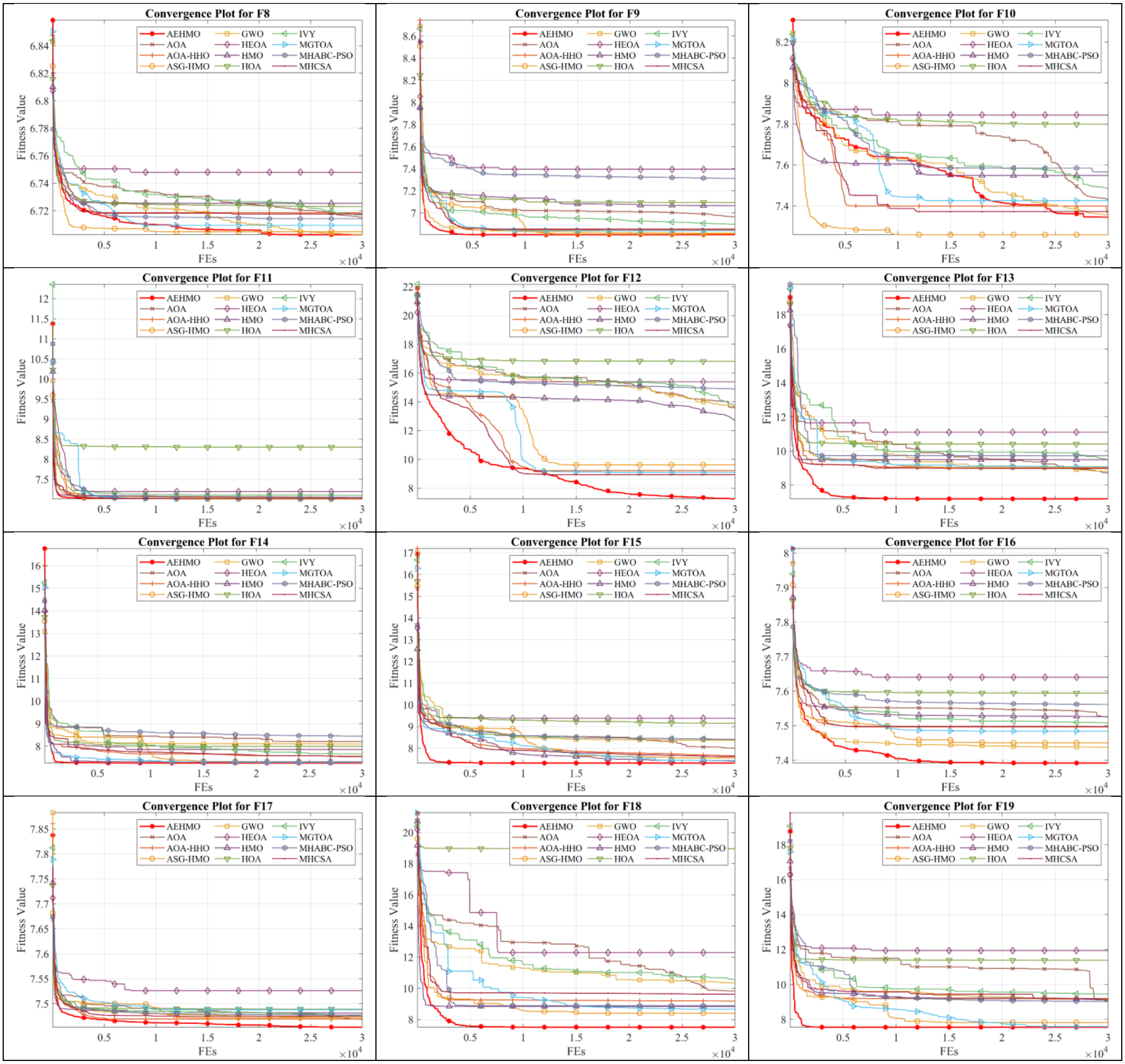
Convergence Plot for F8
Convergence Plot for F9
Convergence Plot for F10
Convergence Plot for F11
Convergence Plot for F12
Convergence Plot for F13
Convergence Plot for F14
Convergence Plot for F15
Convergence Plot for F16
Convergence Plot for F17
Convergence Plot for F18
Convergence Plot for F19
AEHMO
AOA
AOA-HHO
ASG-HMO
GWO
HEOA
HMO
HOA
IVY
MGTOA
MHABC-PSO
MHCSA
Fitness Value
FEs
$\times 10^4$

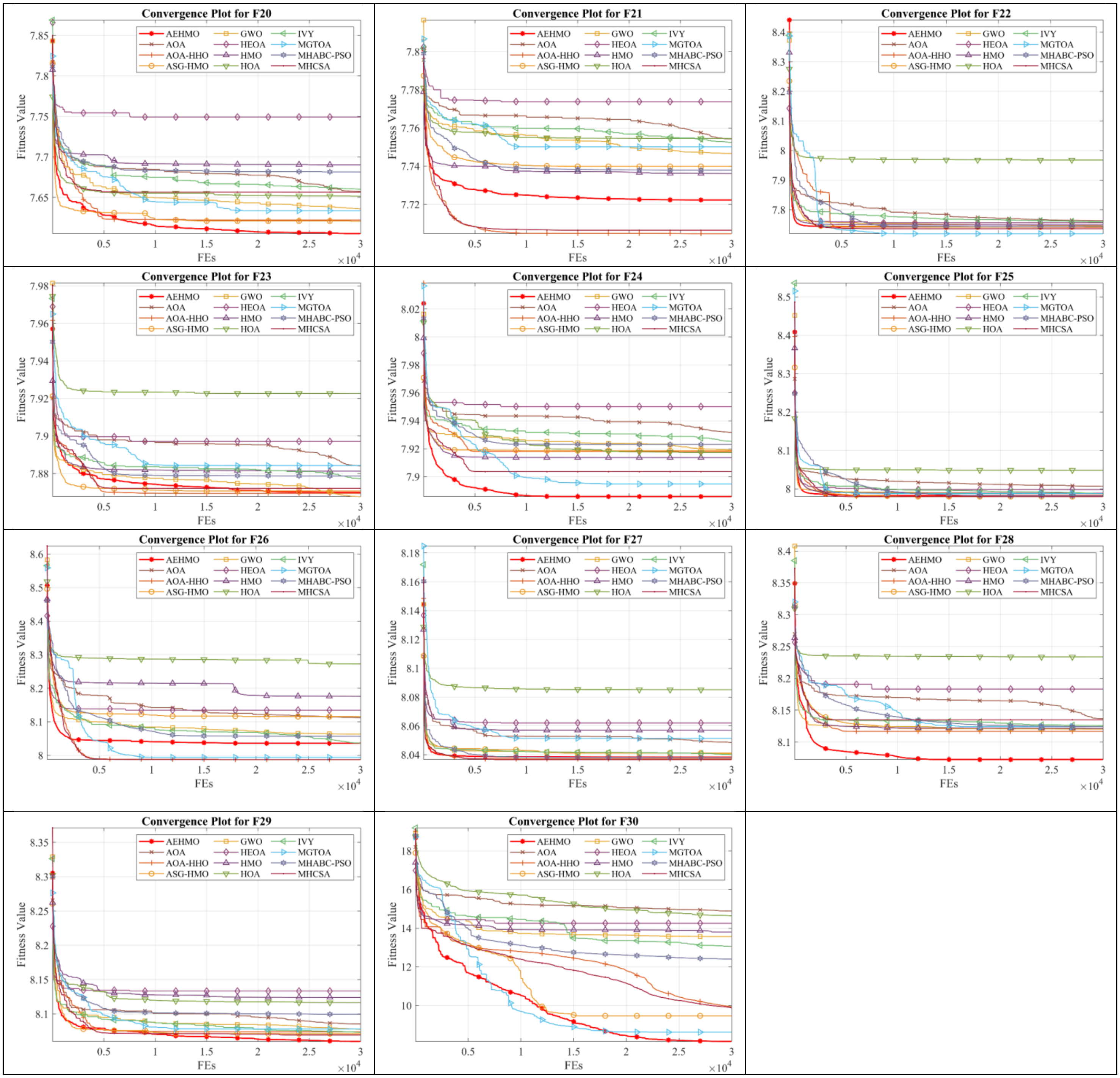

Figure D1 convergence curves of various algorithms using CEC2017, 10D.

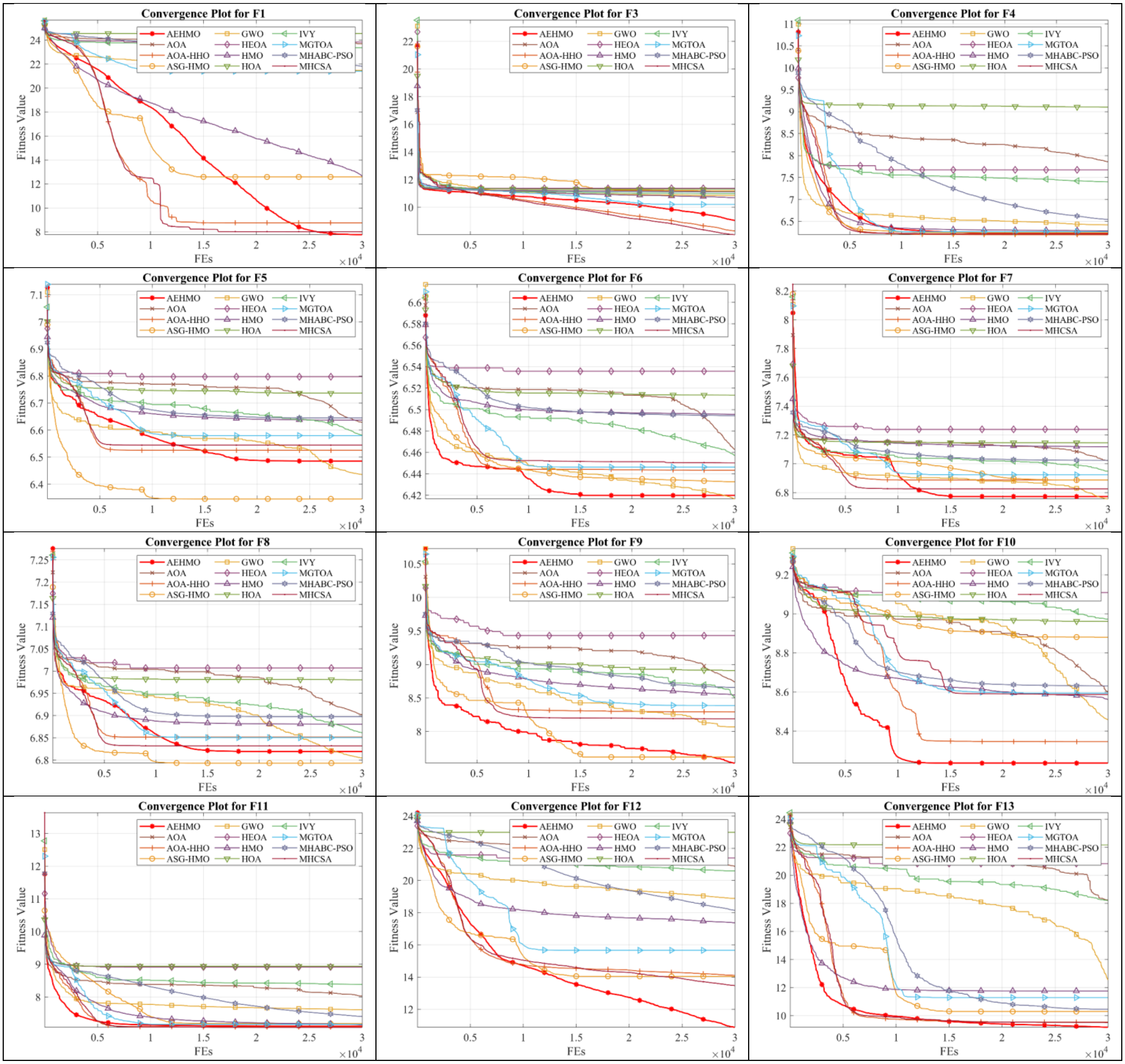
Convergence Plot for F1
Convergence Plot for F3
Convergence Plot for F4
Convergence Plot for F5
Convergence Plot for F6
Convergence Plot for F7
Convergence Plot for F8
Convergence Plot for F9
Convergence Plot for F10
Convergence Plot for F11
Convergence Plot for F12
Convergence Plot for F13
AEHMO
AOA
AOA-HHO
ASG-HMO
GWO
HEOA
HMO
HOA
IVY
MGTOA
MHABC-PSO
MHCSA
Fitness Value
FEs
×10^4

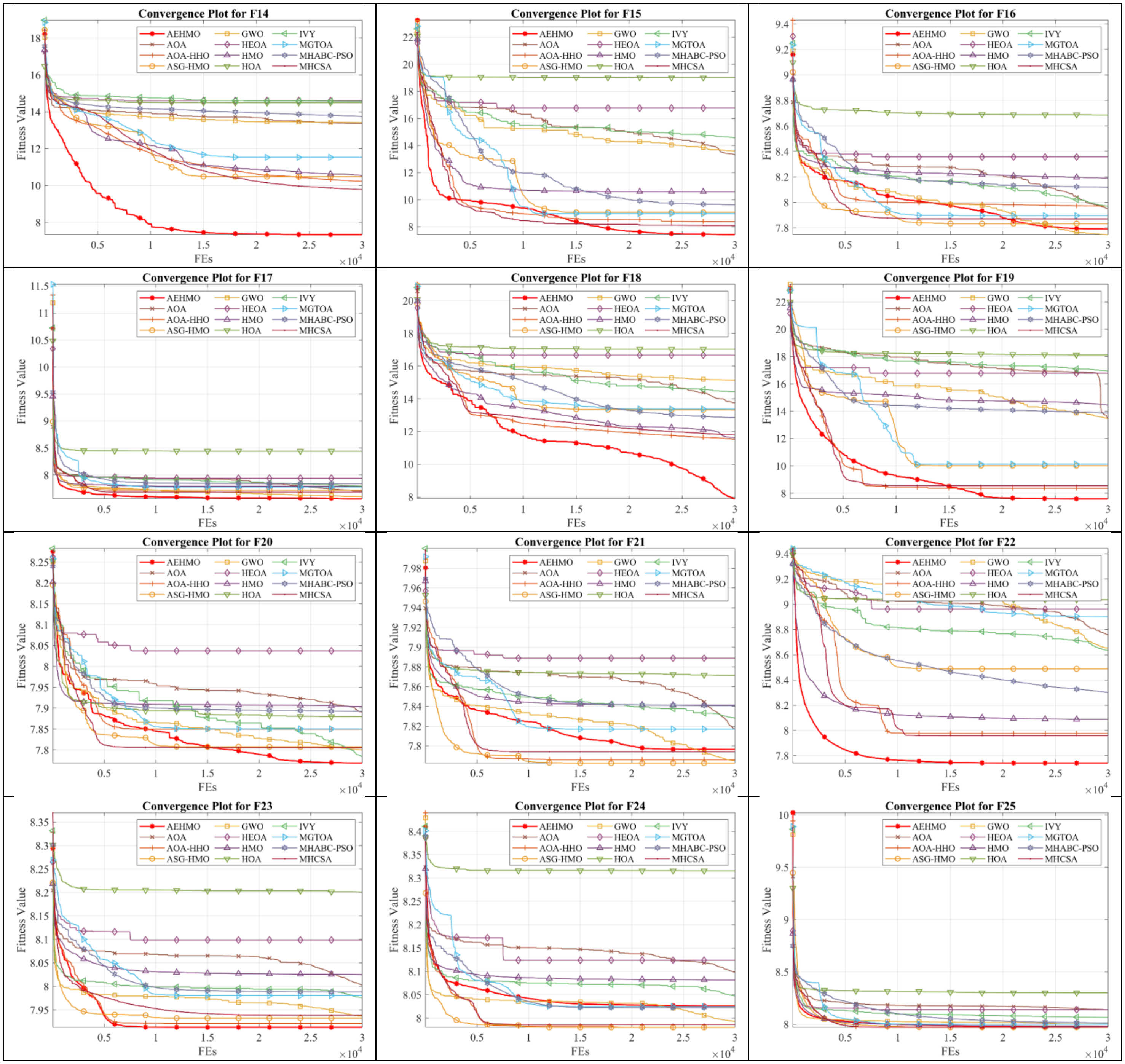
Convergence Plot for F14
Convergence Plot for F15
Convergence Plot for F16
Convergence Plot for F17
Convergence Plot for F18
Convergence Plot for F19
Convergence Plot for F20
Convergence Plot for F21
Convergence Plot for F22
Convergence Plot for F23
Convergence Plot for F24
Convergence Plot for F25
AEHMO
AOA
AOA-HHO
ASG-HMO
GWO
HEOA
HMO
HOA
IVY
MGTOA
MHABC-PSO
MHCSA
Fitness Value
FEs
×10^4

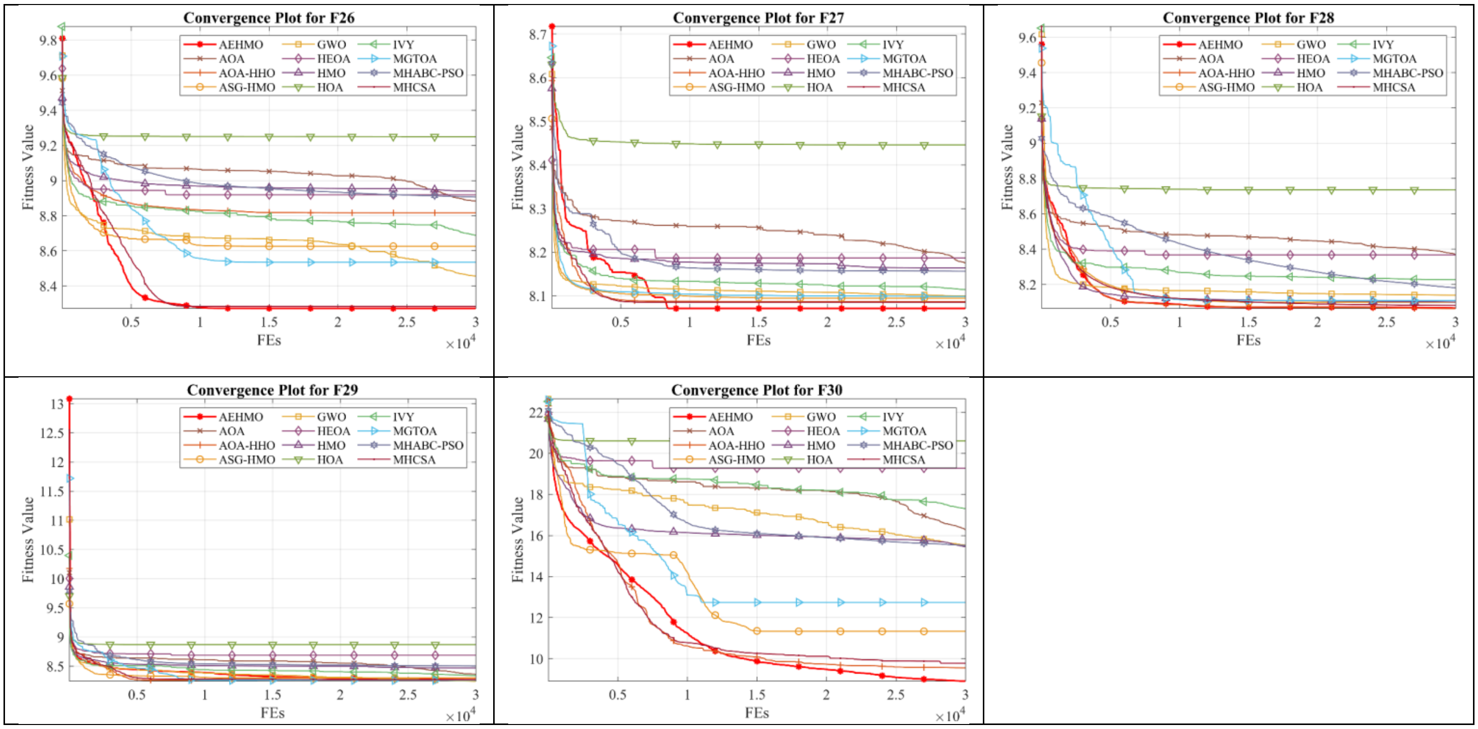

Figure D2 convergence curves of various algorithms using CEC2017, 30D.

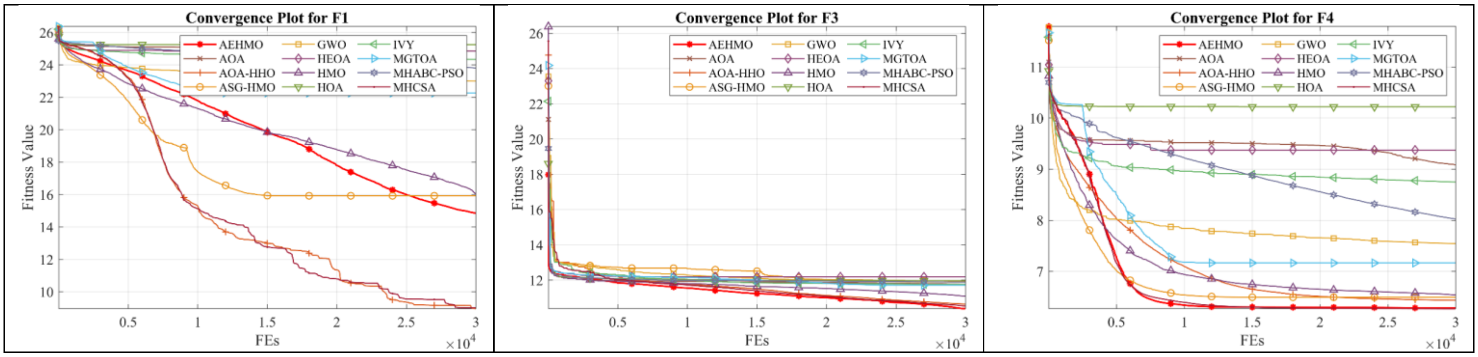

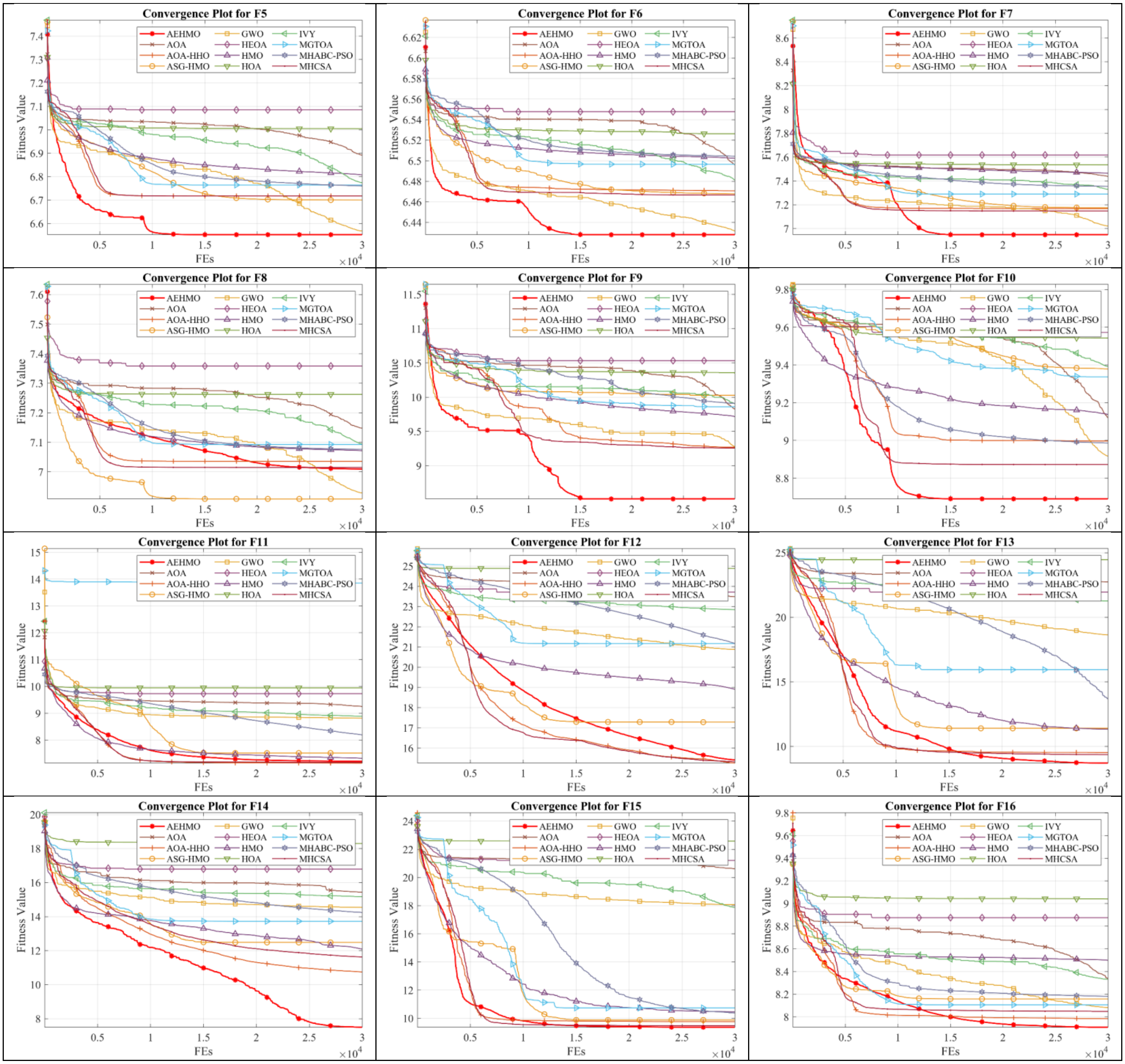
Convergence Plot for F5
Convergence Plot for F6
Convergence Plot for F7
Convergence Plot for F8
Convergence Plot for F9
Convergence Plot for F10
Convergence Plot for F11
Convergence Plot for F12
Convergence Plot for F13
Convergence Plot for F14
Convergence Plot for F15
Convergence Plot for F16
AEHMO
AOA
AOA-HHO
ASG-HMO
GWO
HEOA
HMO
HOA
IVY
MGTOA
MHABC-PSO
MHCSA
Fitness Value
FEs
$\times 10^4$

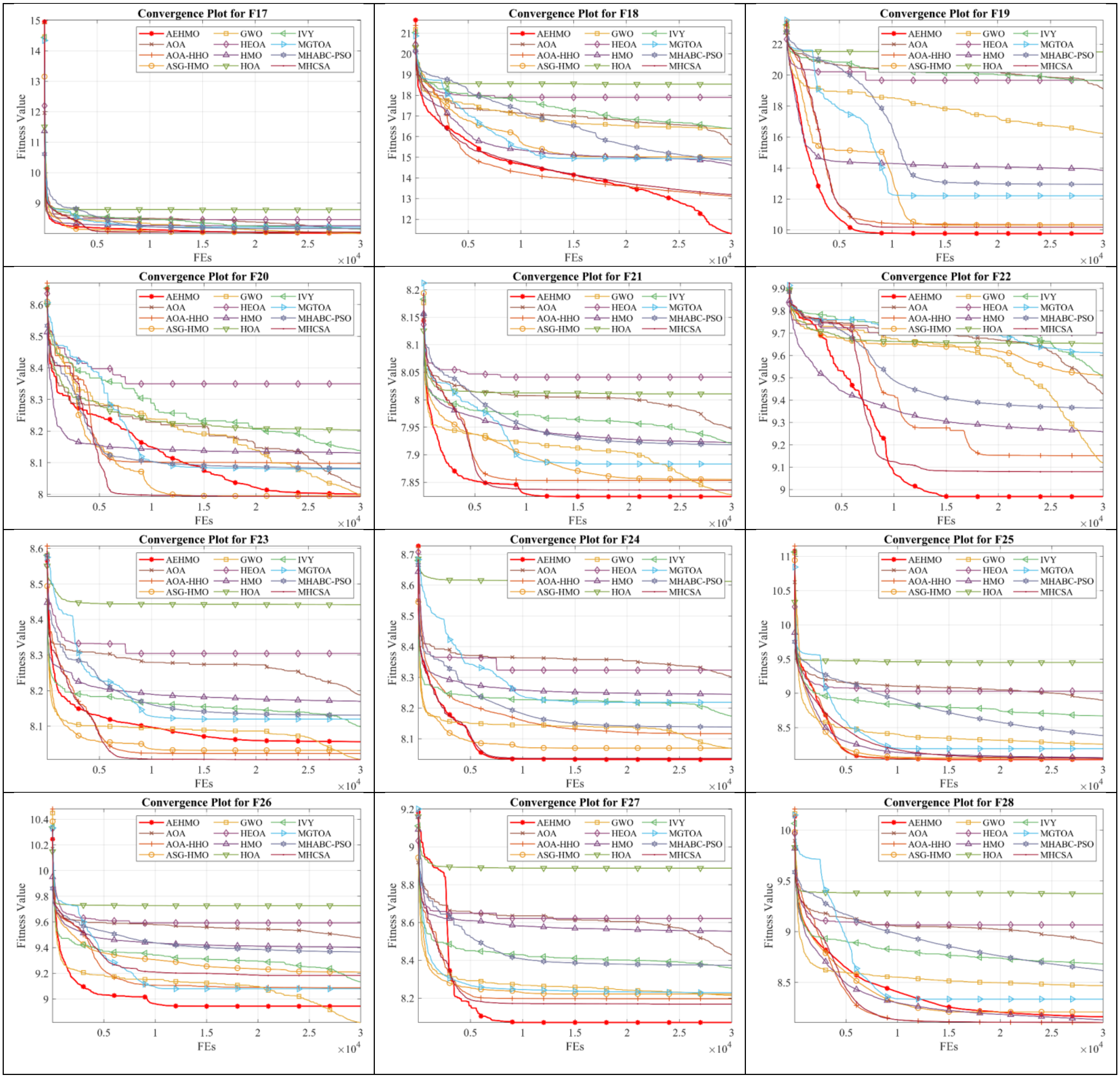

Convergence Plot for F17
Convergence Plot for F18
Convergence Plot for F19
Convergence Plot for F20
Convergence Plot for F21
Convergence Plot for F22
Convergence Plot for F23
Convergence Plot for F24
Convergence Plot for F25
Convergence Plot for F26
Convergence Plot for F27
Convergence Plot for F28
AEHMO
AOA
AOA-HHO
ASG-HMO
GWO
HEOA
HMO
HOA
IVY
MGTOA
MHABC-PSO
MHCSA
Fitness Value
FEs
×10^4

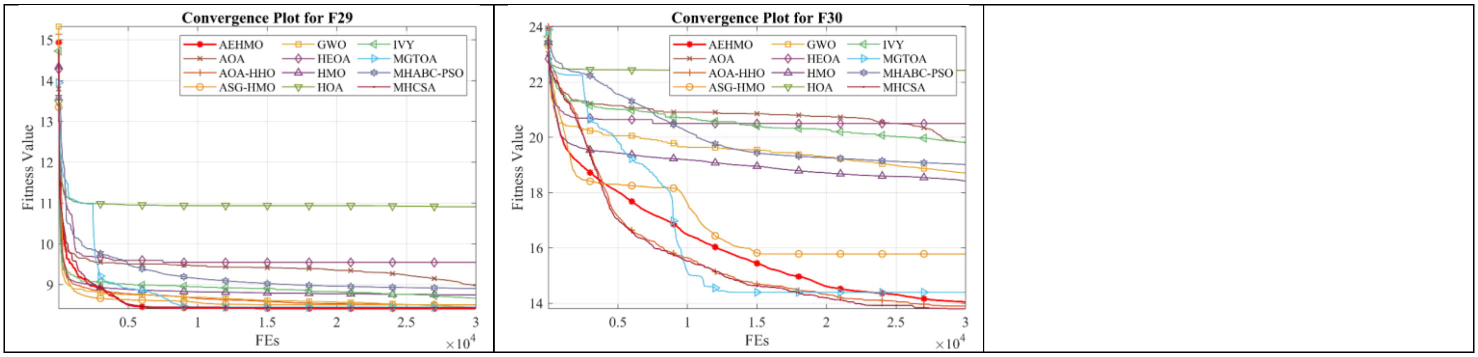

Figure D3 convergence curves of various algorithms using CEC2017, 50D.

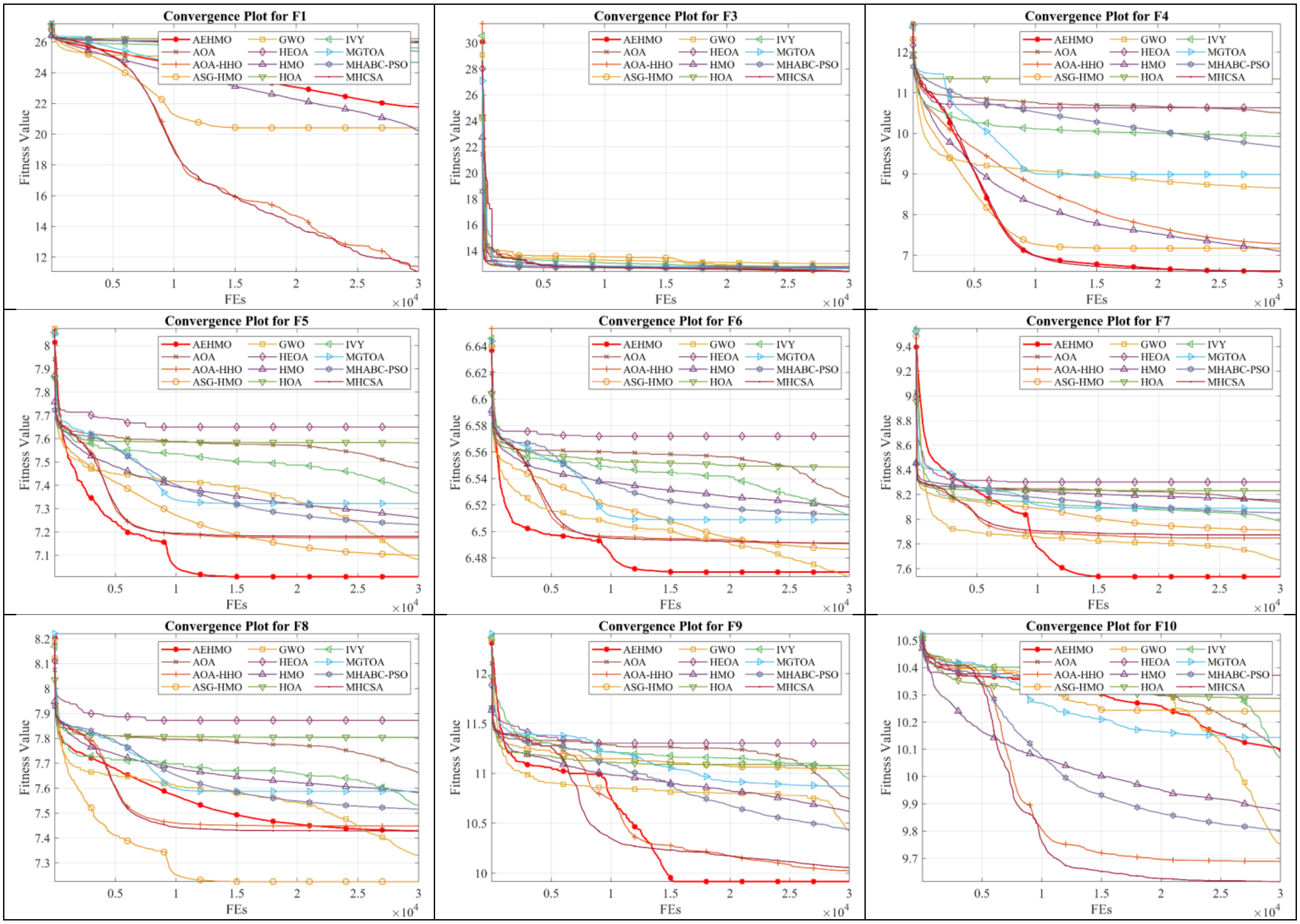

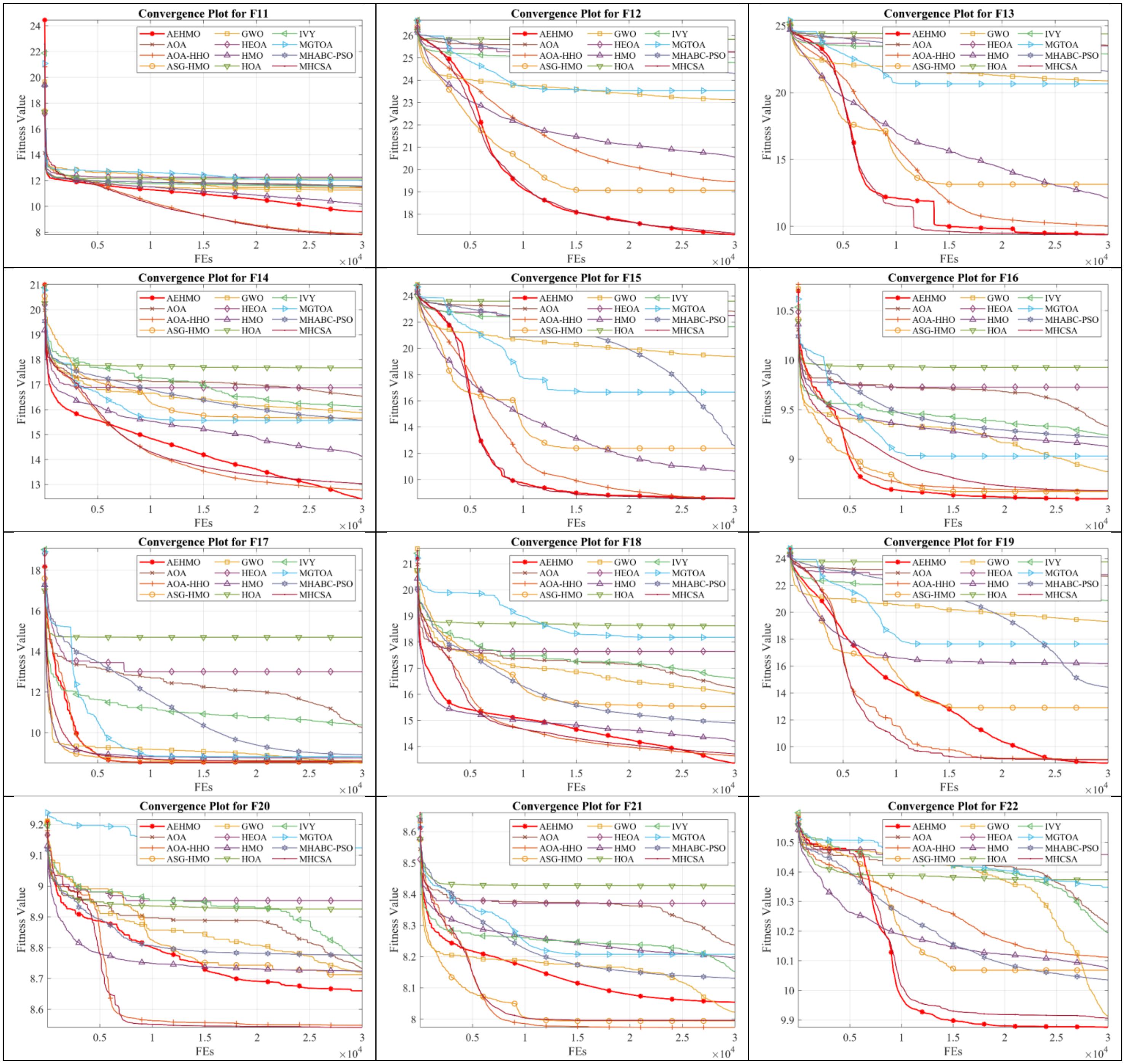
Convergence Plot for F11
Convergence Plot for F12
Convergence Plot for F13
Convergence Plot for F14
Convergence Plot for F15
Convergence Plot for F16
Convergence Plot for F17
Convergence Plot for F18
Convergence Plot for F19
Convergence Plot for F20
Convergence Plot for F21
Convergence Plot for F22
AEHMO
AOA
AOA-HHO
ASG-HMO
GWO
HEOA
HMO
HOA
IVY
MGTOA
MHABC-PSO
MHCSA
Fitness Value
FEs
×10^4

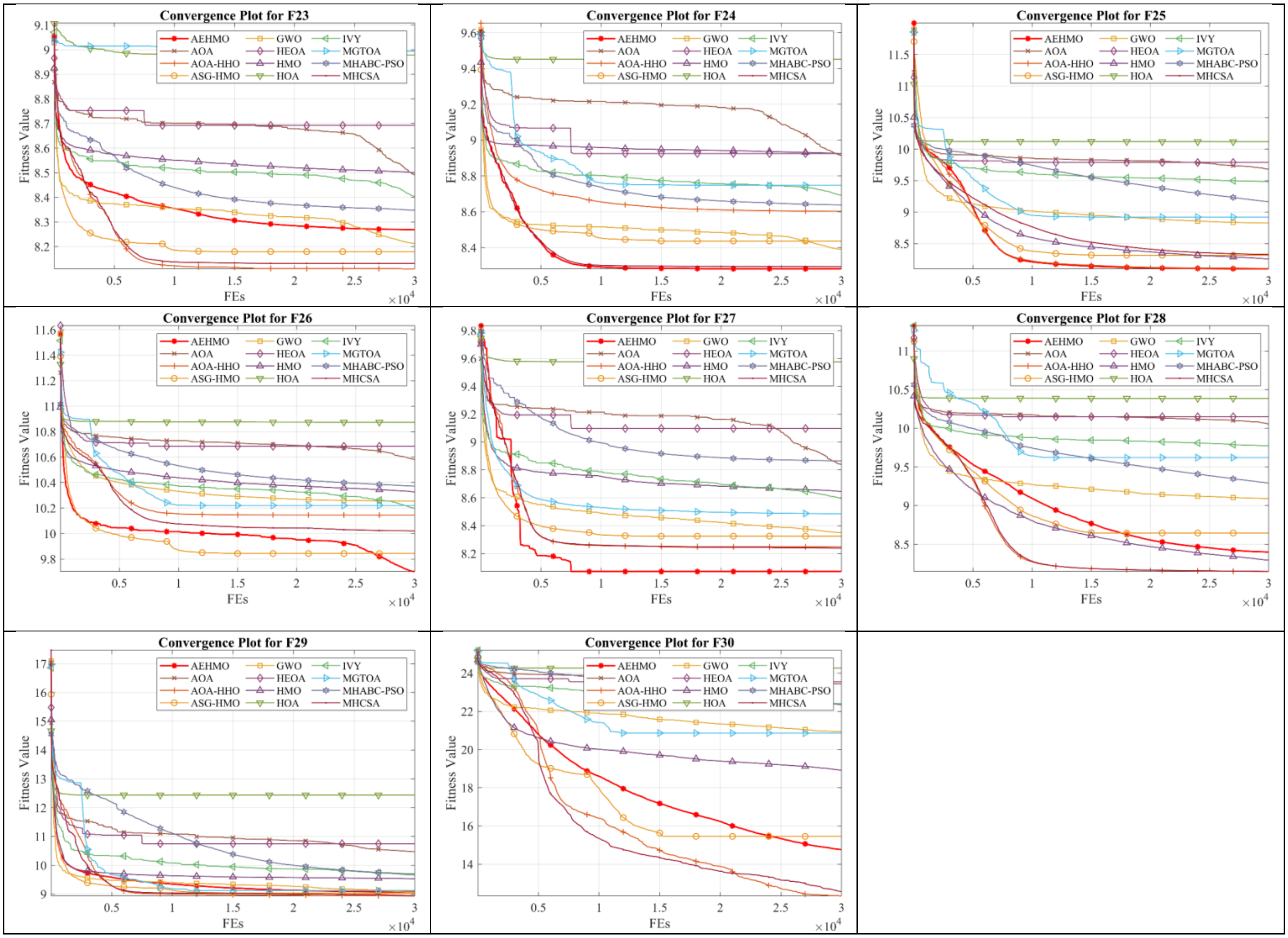

Figure D4 convergence curves of various algorithms using CEC2017, 100D.

Appendix E: Boxplots

Unimodal and multimodal function boxplot distributions indicate the high consistency of AEHMO's solutions and robustness across all tested dimensions shown in Figs. E1-E4. In unimodal functions F1 and F3, AEHMO has highly compact boxplot distributions on all dimensions, with very narrow interquartile ranges and low variance across 30 independent runs. For F1 in 10D, AEHMO exhibits a near-zero interquartile range with the median, first quartile and third quartile as a single line which confirms near-perfect convergence consistency with standard deviation approaching zero. This tight distribution still exists in 30D and 50D but F1 in 100D, shows more box height, as a result of the above-mentioned dimensional scaling issues, where AEHMO was ranked 5th with higher variance. In F3 in all dimensions, AEHMO has tight distributions with no outliers in the 10D, 30D and 50D, which shows the effectiveness of the adaptive parameters and CRL strategy in maintaining population diversity and guaranteeing consistent convergence to high-quality solutions. For multimodal functions, AEHMO shows quite uniform boxplots for most functions and most dimensions, with interesting stability for F4, F6, F7, and F9. The F4 boxplot in 10D has

a narrow distribution similar to the unimodal function performance suggesting that AEHMO handles the rotated function with exceptional consistency. In F5 across 30D, 50D and 100D, the boxplots of AEHMO have slightly larger interquartile ranges than in F4 and F6 which correlate to the performance of these dimensions as observed, confirming the introduction of the variability of convergence due to the periodic structure of the functions. The F8 boxplots for every dimension show moderate box heights, but no significant outliers, which is a good sign that although different runs may converge to a bit different local optima in the expanded function, the algorithm is not going out of bounds of bounded variance with catastrophic failures. Comparing AEHMO's distributions against competitors shows systematic benefits where the distributions of algorithms such as IVY, AOA, HEOA and HOA show significantly larger box heights with heavy-tailed distributions and many outliers, suggesting erratic performance across independent runs while AEHMO, MHCSA and AOA-HHO show the most compact distributions suggesting better algorithmic stability.

For hybrid functions, the characteristics of AEHMO's boxplots exhibit excellent robustness with small variance in an overwhelming majority of test cases and dimensions. In F12, F13, F14, F15, F18 and F19 in 10D, 30D and 50D, AEHMO routinely has smallest boxplots compared to other competing algorithms, with interquartile range visually smaller than those of competitors by large margins. The F12 boxplot in 10D shows AEHMO with essentially negligible variance, collapsing to a near-degenerate distribution with competitors like IVY and HOA. This pattern repeats in F13 and F18 in all dimensions where the boxplots of AEHMO are compressed at the low end of the fitness scale, but the distributions of competitors are widely spread out, confirming that the MDMS strategy is successful in terms of maintaining the consistency of solution quality, since mutations are focused on the elite individuals in the population and not the entire population. In F14 and F15 in 30D and 50D, AEHMO's boxplots show somewhat larger interquartile ranges than 10D, as is to be expected with more complexity in higher dimensional hybrid spaces but are much more compact than most competitors. The F16 boxplots in 30D and 50D reveal some interesting dimensional dependencies, where AEHMO is exhibiting tight distributions in lower dimensions, but with increased variances in 50D and 100D, with visible expansion of box heights, and sometimes with upper whisker extensions, correlating with the performance in these situations, suggesting that some rotated expanded compositions pose a challenge to AEHMO consistency mechanisms. In F11 on 50D and 100D, AEHMO's boxplots have moderate interquartile ranges comparable to MHCSA and AOA-HHO, competitive but not dominant stability in hybrid compositions with high dimensions. The F20 boxplots in 100D show AEHMO with relatively compact distribution in spite of performance and shows that while the median solution quality may not be able to achieve top rank, the algorithm has acceptable variance bounds without extreme outliers which would indicate algorithmic instability or susceptibility to initialization sensitivity.

The composite functions show AEHMO's most notable robustness characteristics with the algorithm giving extremely compact boxplot distributions for nearly all functions and dimensions. For the cases F22, F24, F25, F26, F27, F29 and F30 with 10D, 30D, and 50D, AEHMO has consistently the lowest interquartile ranges among all the compared algorithms, many boxplots being essentially horizontal lines. F27 boxplot across all dimensions is a good example of this, which shows AEHMO with almost zero box height in 10D, 30D, and 50D which confirms that the algorithm converges to essentially identical solutions across all runs regardless of random initialization. This uniformity is consistent with the stabilizing effect of CRL, which promotes structured exploration and reduces run-to-run variance. In F30 in 10D, 30D and 50D, AEHMO has compact boxplots with much lower fitness values than all the competitors, and the box central of the closest competitors are greater, confirming the substantial advantages in performance in the statistical results. Across all dimensions, the F24 boxplots suggest that AEHMO has small distribution with no outliers which demonstrates that these functions do not cause convergence instability even though it has a complex multi-basin structure. In F21, F23 and F28 in 30D, 50D and 100D, AEHMO's boxplots show a slightly larger interquartile range than those of other composite functions, most noticeably in 100D where the box heights increase quite significantly, which correlates with the performance degradations in these combinations of function dimension. The F23 boxplot in 100D shows AEHMO with moderately sized box with visible extension of whiskers, variance in solution quality across the runs being one of the contributions

to the fifth-rank performance, suggesting that these composite functions in high dimensions generate landscapes where the memory-based recall mechanism generates solution quality variability depending on the trajectory of search followed during the early exploration phases.

Despite the robustness of AEHMO under most circumstances, some boxplot patterns indicate important limitations of variance and stability under some circumstances. In F1 at 100D, AEHMO's boxplot shows a drastically increased interquartile range compared to the lower dimensions, where the height of the boxes has increased, and the distribution is no longer centered around the compact patterns seen in 10D-50D, suggesting that the high dimensional sphere function introduces initialization dependent convergence behavior, where different starting populations will converge to substantially different final solutions. The presence of long-lasting upper whiskers in F1 at 100D with no visible outliers indicates that some runs had slower convergence, which justifies the absence of convergence consistency in the curve analysis in which oscillations had been shown in the optimization process. In F3 at 100D, AEHMO's boxplot is reasonably compact but with a larger box height than at 50D with the distribution center higher than several competitors including MHCSA and AOA-HHO. The multimodal function F5 boxplots across 30D, 50D and 100D consistently show AEHMO with moderate to large interquartile ranges compared to its performance in other multimodal functions with visible whisker extensions in both directions, indicating that individual runs converge to substantially different solution qualities, confirming that this function structure introduces variance which AEHMO's diversity mechanisms cannot fully suppress. In F10 at 100D, AEHMO's boxplot has significant box height and long whiskers, which suggest high variance across runs that contributed to the performance of the seventh rank, and the distribution has some data points near the upper whisker boundary that suggest that multiple runs had convergence difficulties in the 100-dimensional.

For composite functions, F21 boxplots for 50D and 100D depict AEHMO with considerably larger boxes compared to 10D-30D with the 100D boxplot depicting a distribution that overlaps quite a bit with the boxes of the competitor algorithms, which visually confirms the second-rank rather than first-rank performance, and the result of less consistency in high dimensional composition functions with rotated hybrid components. The F28 boxplot in 100D is a relatively compact distribution even with fourth-rank performance and interestingly AEHMO is consistent around a suboptimal fitness level in comparison to highly variable performance, which implies that the algorithm converges to non-optimal basins reliably, but to non-optimal basins in certain 100D composite structures. These failure patterns in variance and stability indicate that while AEHMO is excellent in ensuring robustness in most optimization scenarios, the combination of high-dimensional spaces (100D) and certain function characteristics introduce the new problem of initialization sensitivity and trajectory-dependent convergence that the current adaptive parameter settings cannot fully control. This results in solution quality variability across independent runs that appear as expanded boxplot distributions, extended whiskers and the occasional outliers that represent rare convergence failures.

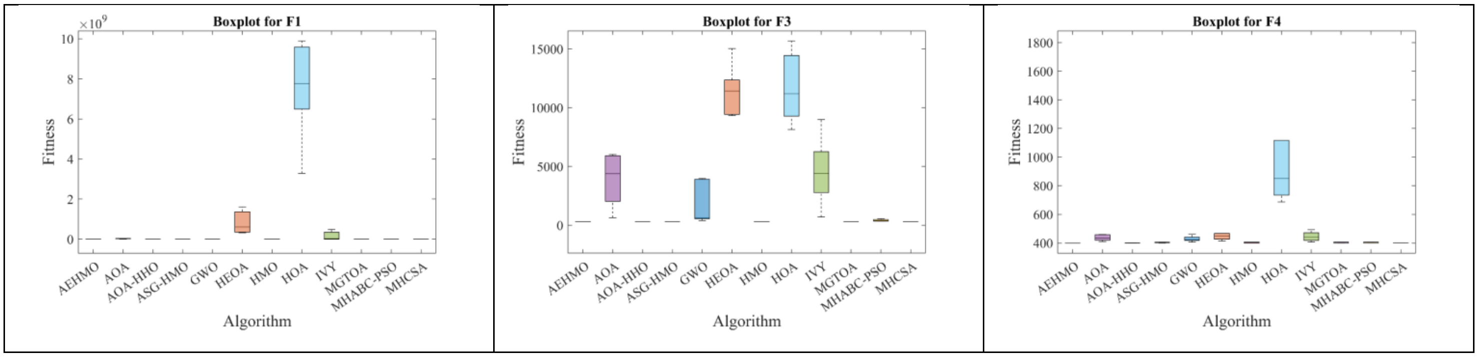

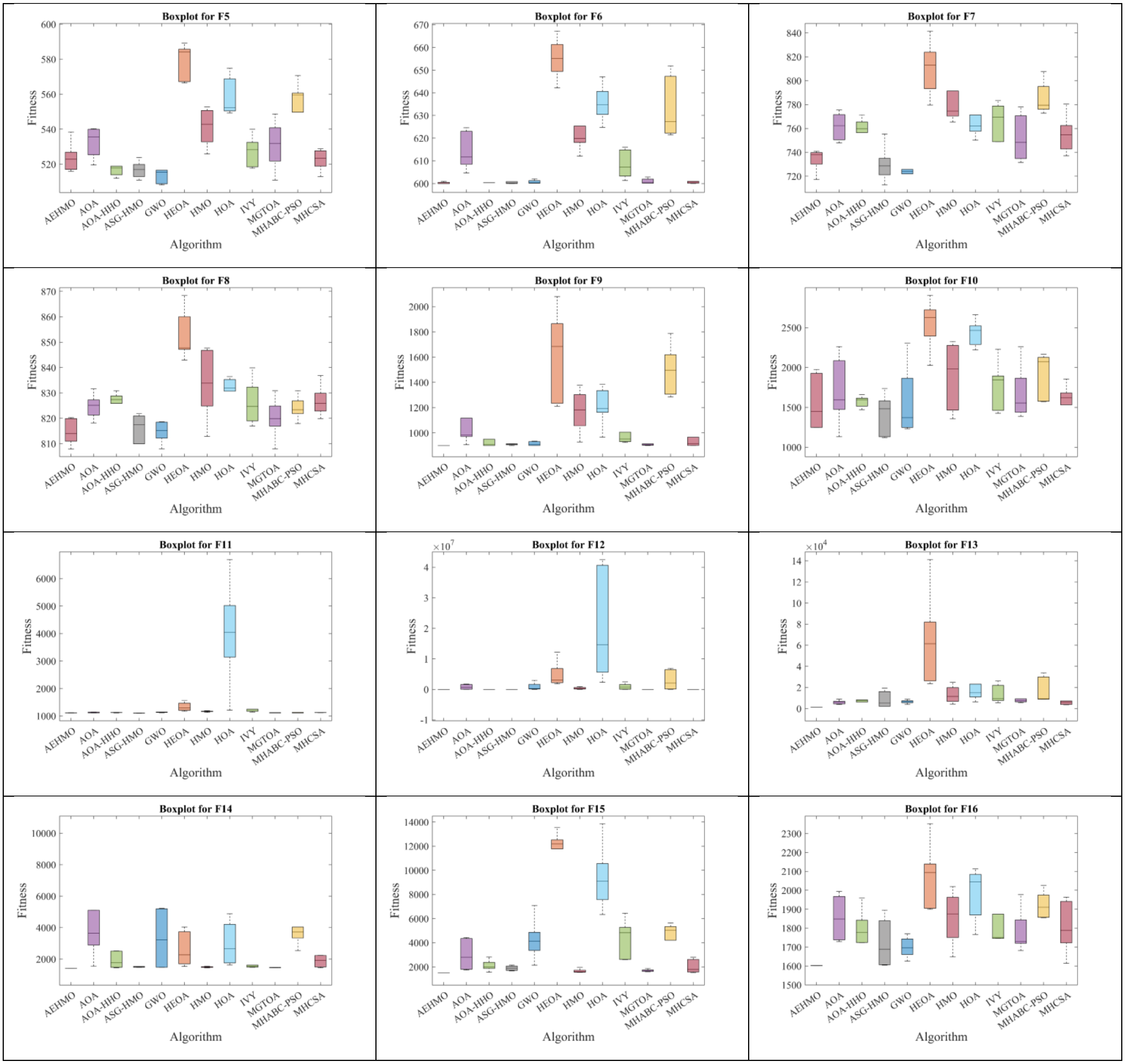
Boxplot for F5
Boxplot for F6
Boxplot for F7
Boxplot for F8
Boxplot for F9
Boxplot for F10
Boxplot for F11
Boxplot for F12
Boxplot for F13
Boxplot for F14
Boxplot for F15
Boxplot for F16
Fitness
Algorithm
AEHMO
AOA
AOA-HHO
ASG-HMO
GWO
HEOA
HMO
HOA
IVY
MGTOA
MHABC-PSO
MHCSA
×10^7
×10^4

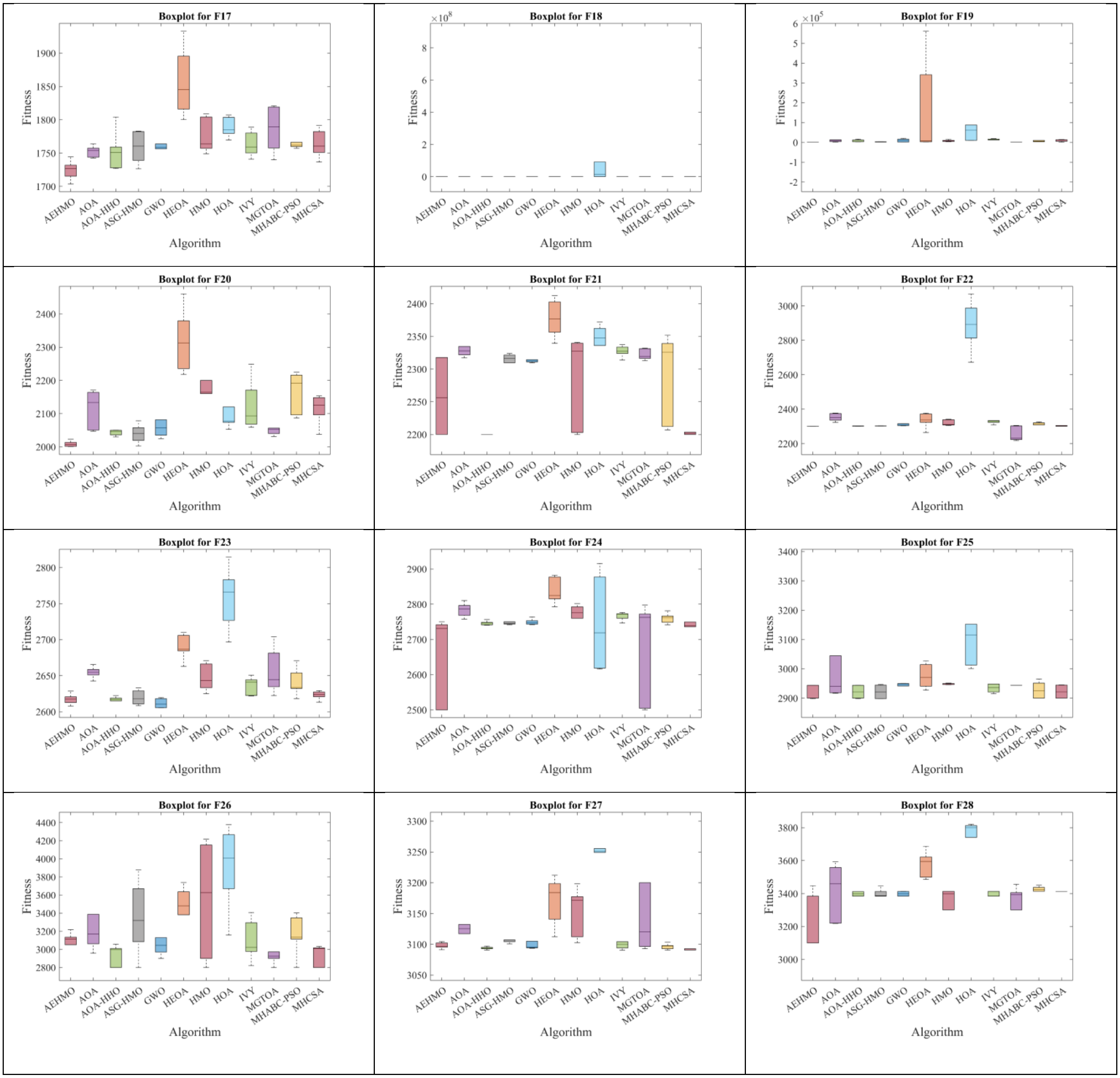
Boxplot for F17
Boxplot for F18
Boxplot for F19
Boxplot for F20
Boxplot for F21
Boxplot for F22
Boxplot for F23
Boxplot for F24
Boxplot for F25
Boxplot for F26
Boxplot for F27
Boxplot for F28
Fitness
Algorithm
AEHMO
AOA
AOA-HHO
ASG-HMO
GWO
HEOA
HMO
HOA
IVY
MGTOA
MHABC-PSO
MHCSA

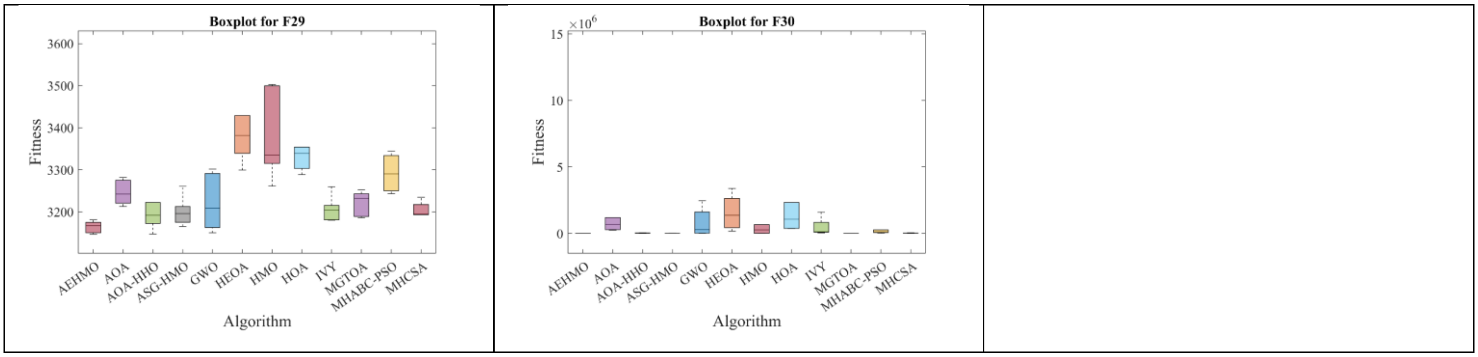

Figure E1 Boxplots of various algorithms using CEC2017, 10D.

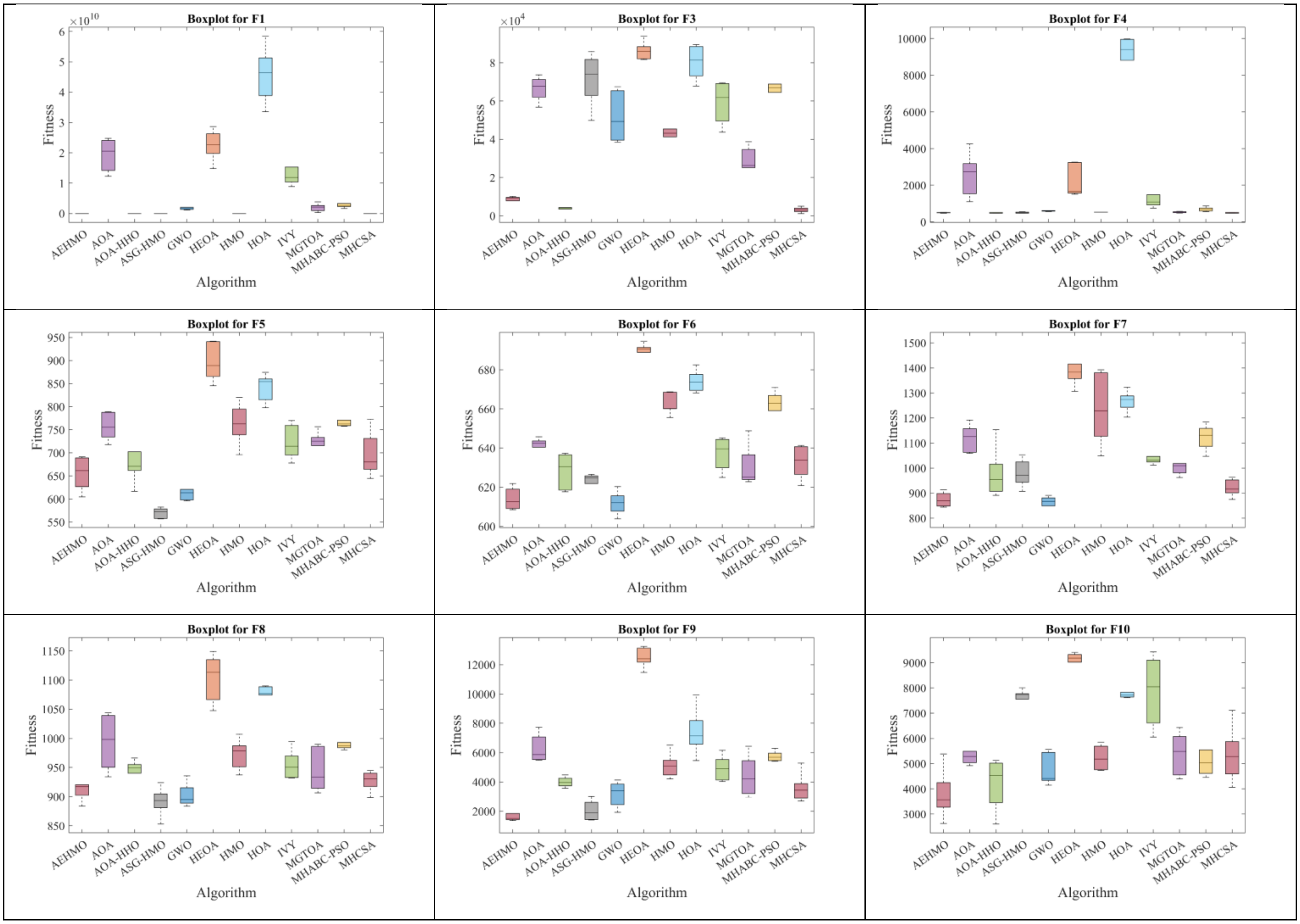

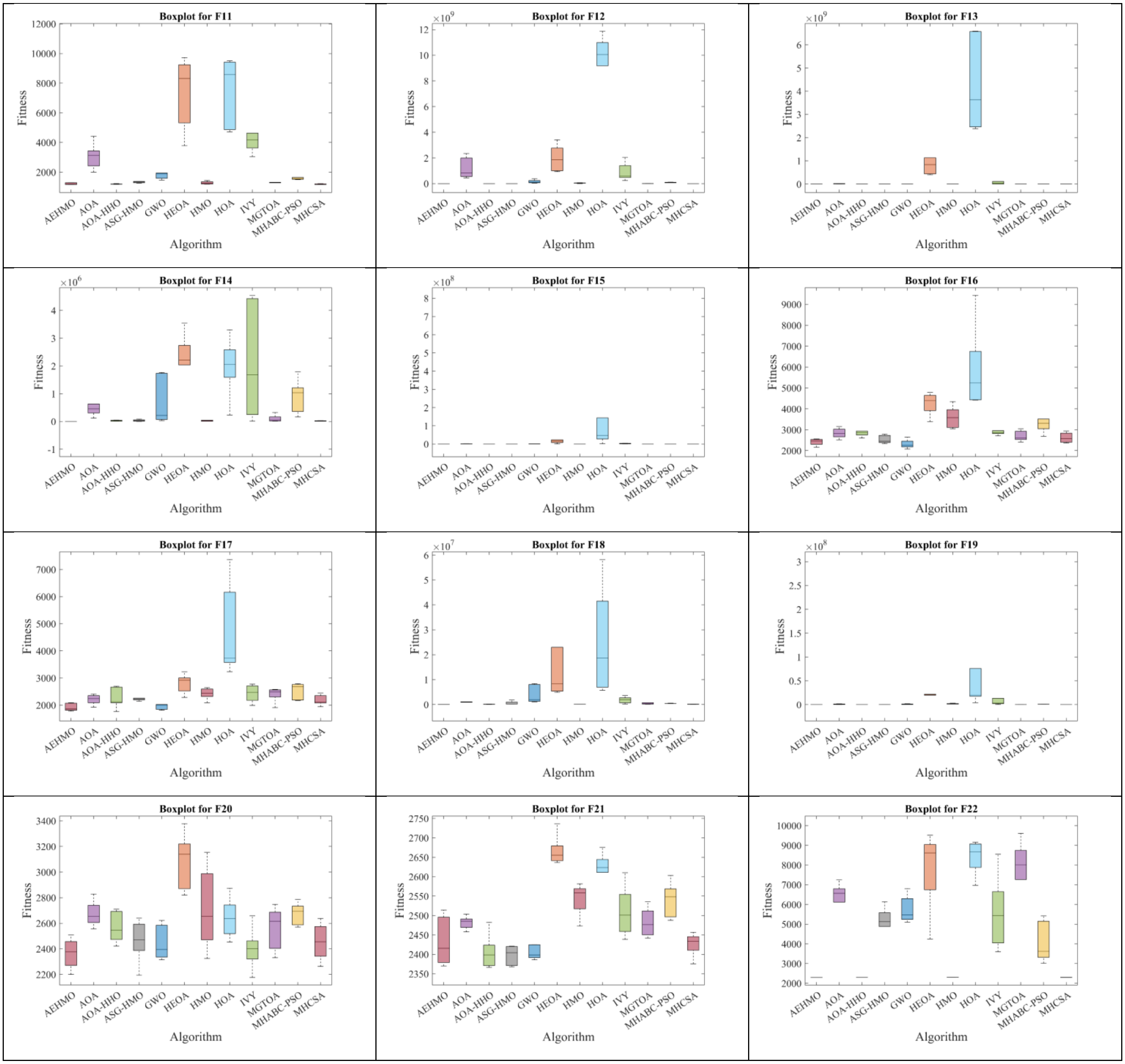

Boxplot for F11
Boxplot for F12
Boxplot for F13
Boxplot for F14
Boxplot for F15
Boxplot for F16
Boxplot for F17
Boxplot for F18
Boxplot for F19
Boxplot for F20
Boxplot for F21
Boxplot for F22
Fitness
Algorithm
AEHMO
AOA
AOA-HHO
ASG-HMO
GWO
HEOA
HMO
HOA
IVY
MGTOA
MHABC-PSO
MHCSA

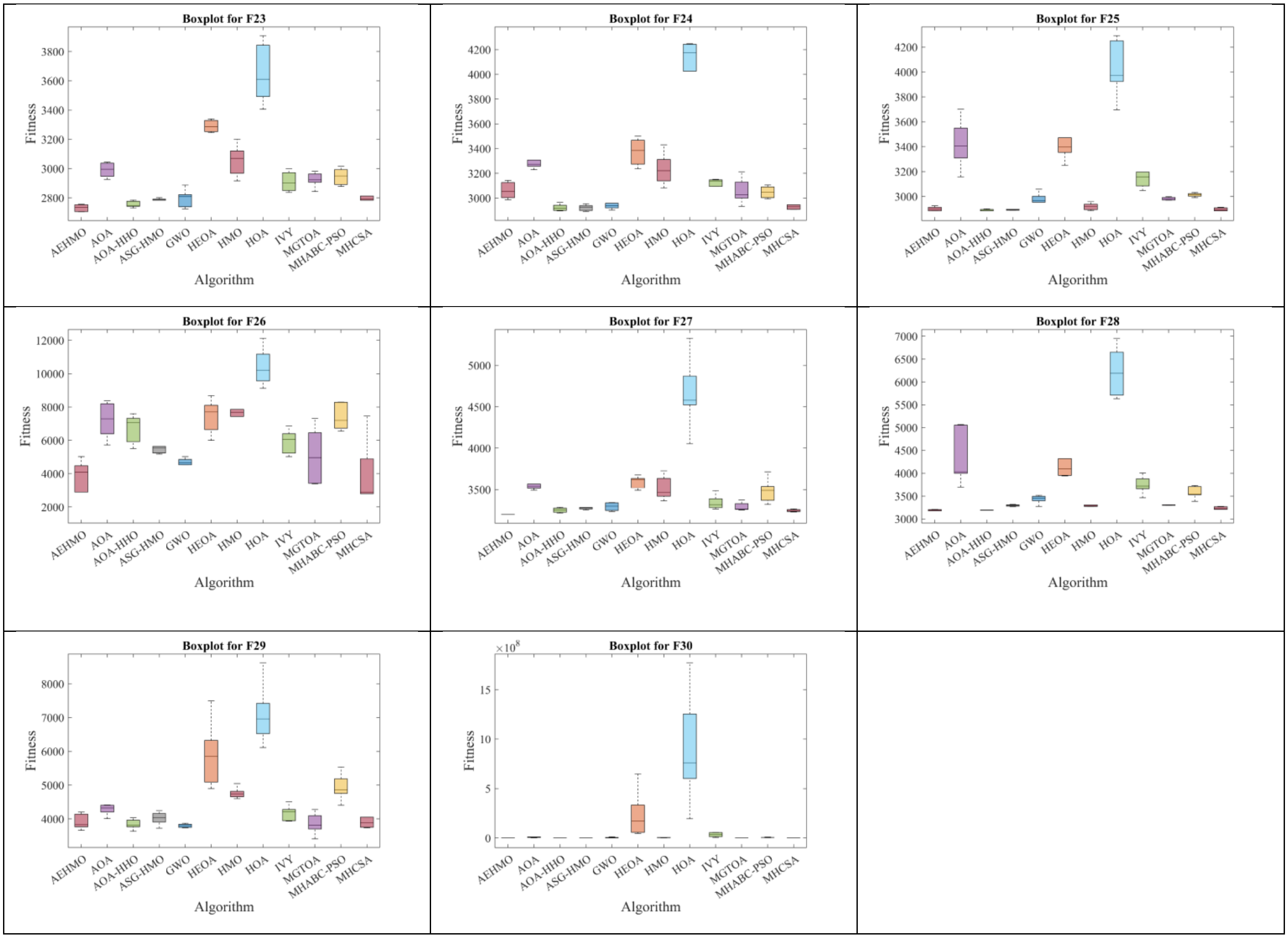

Figure E2 Boxplots of various algorithms using CEC2017, 30D.

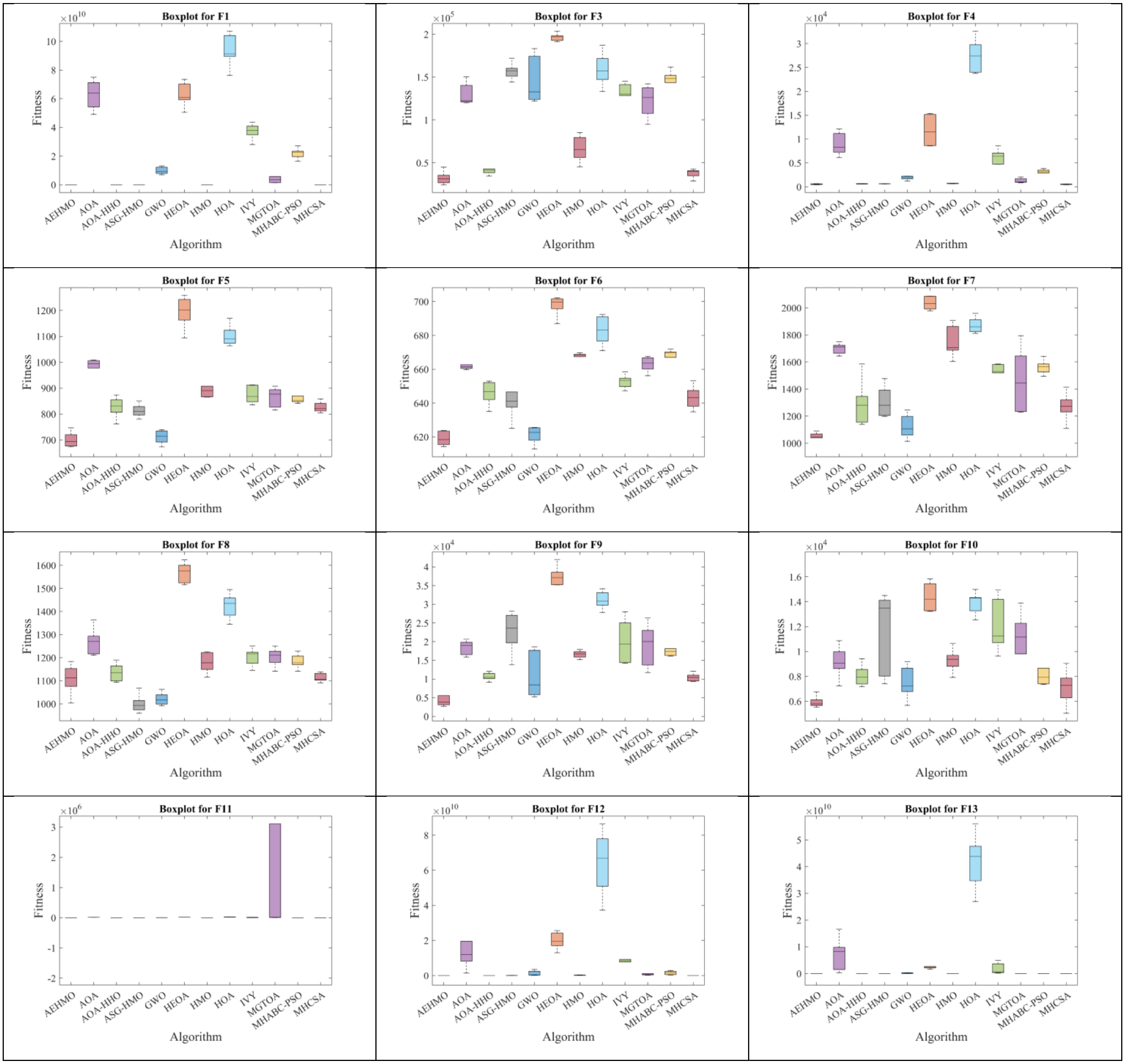
Boxplot for F1
Boxplot for F3
Boxplot for F4
Boxplot for F5
Boxplot for F6
Boxplot for F7
Boxplot for F8
Boxplot for F9
Boxplot for F10
Boxplot for F11
Boxplot for F12
Boxplot for F13
Fitness
Algorithm
AEHMO
AOA
AOA-HHO
ASG-HMO
GWO
HEOA
HMO
HOA
IVY
MGTOA
MHABC-PSO
MHCSA

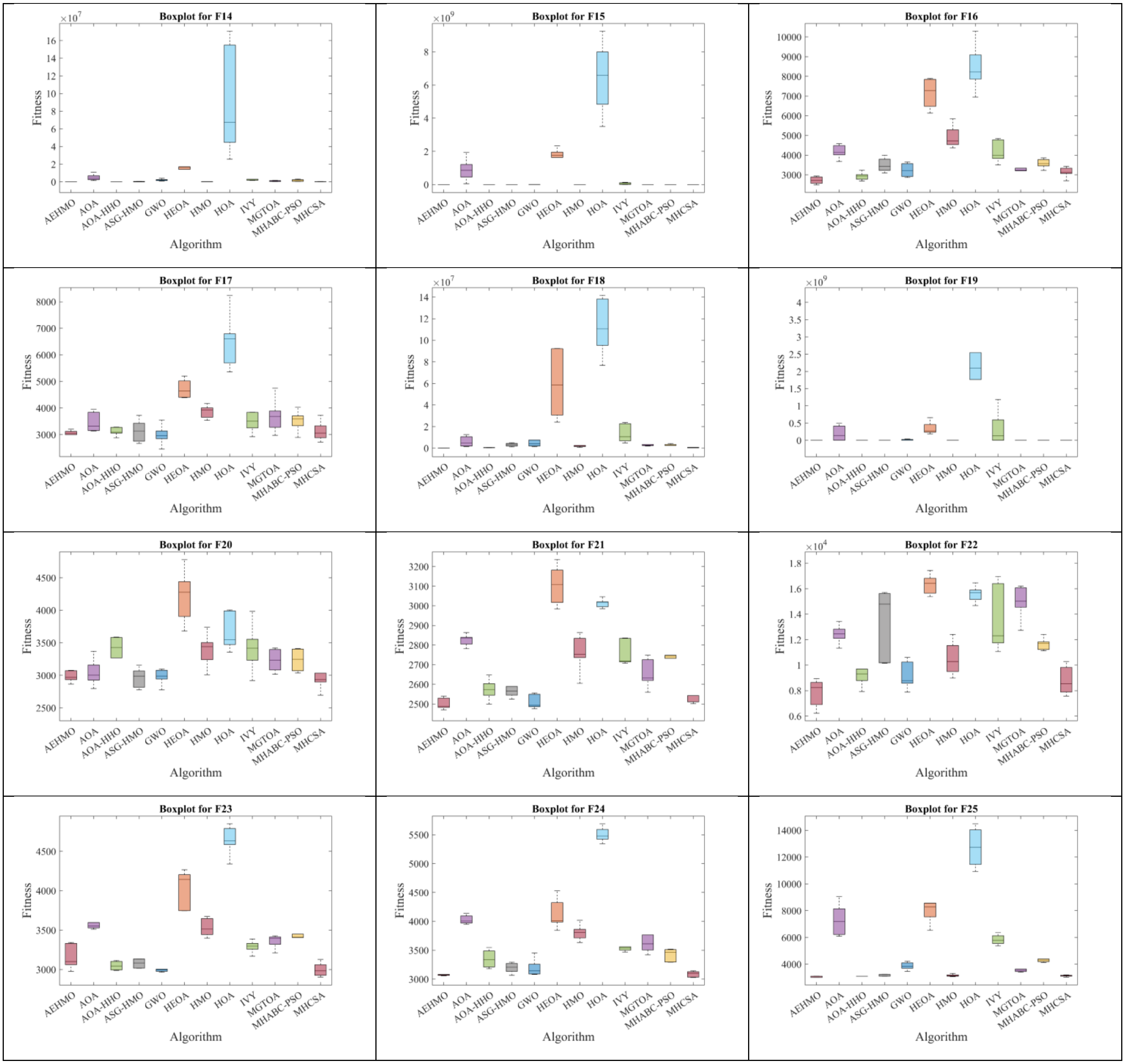
Boxplot for F14
Boxplot for F15
Boxplot for F16
Boxplot for F17
Boxplot for F18
Boxplot for F19
Boxplot for F20
Boxplot for F21
Boxplot for F22
Boxplot for F23
Boxplot for F24
Boxplot for F25
Fitness
Algorithm
AEHMO
AOA
AOA-HHO
ASG-HMO
GWO
HEOA
HMO
HOA
IVY
MGTOA
MHABC-PSO
MHCSA

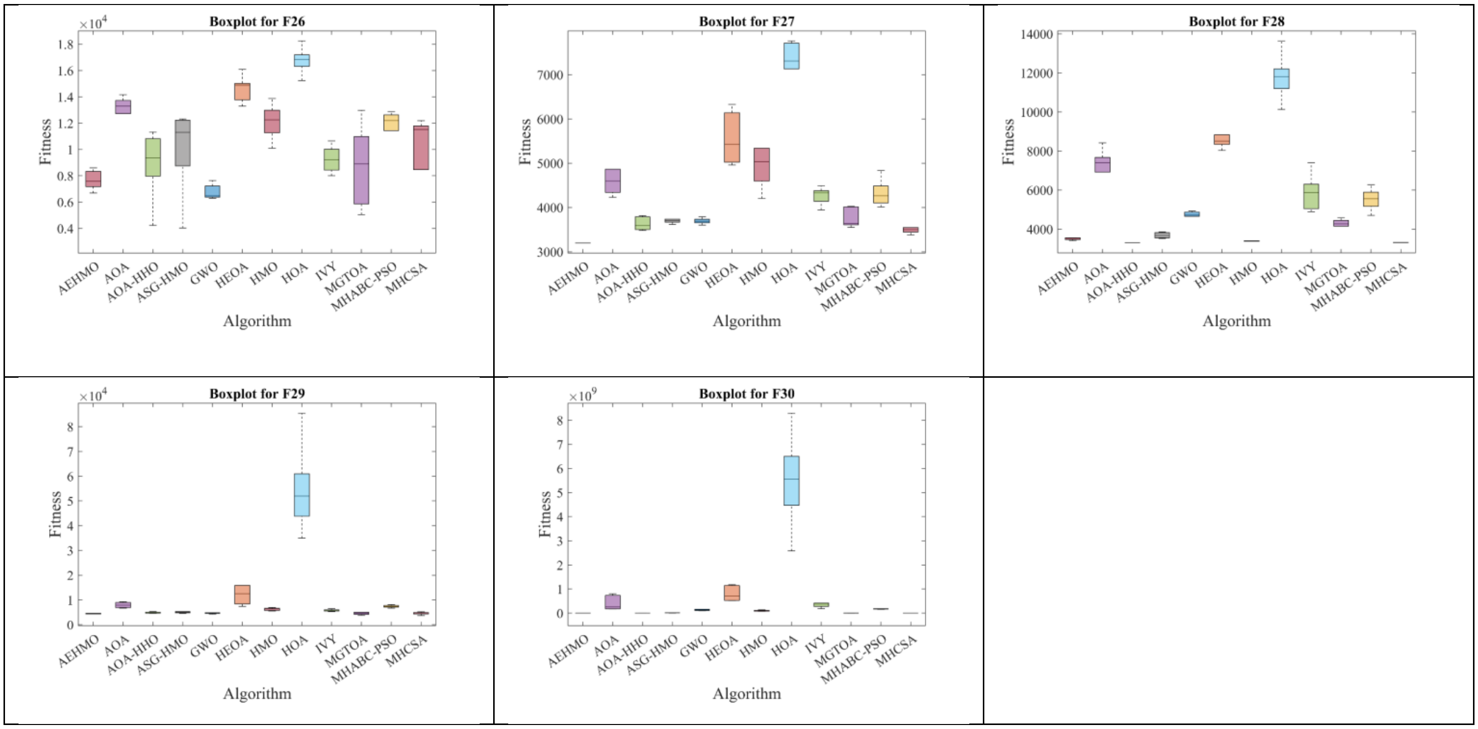

Figure E3 Boxplots of various algorithms using CEC2017, 50D.

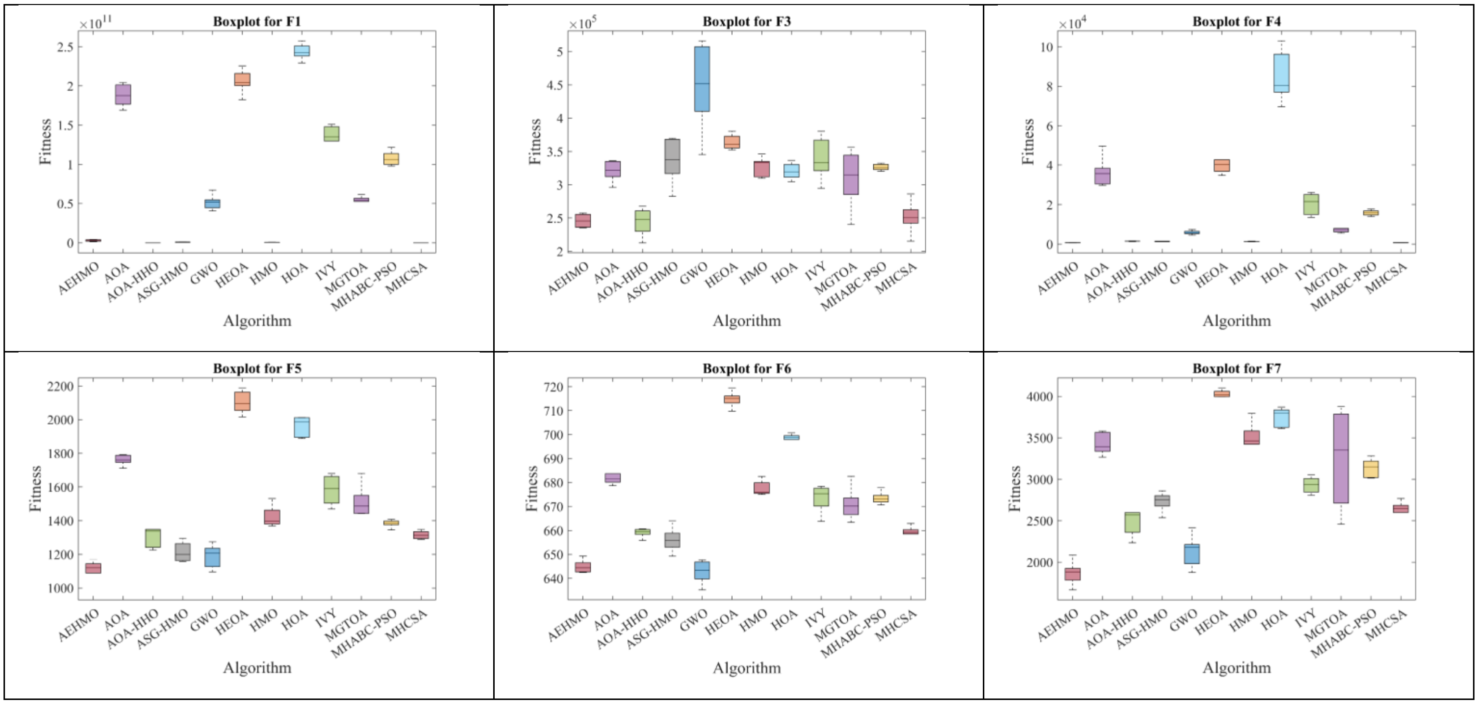

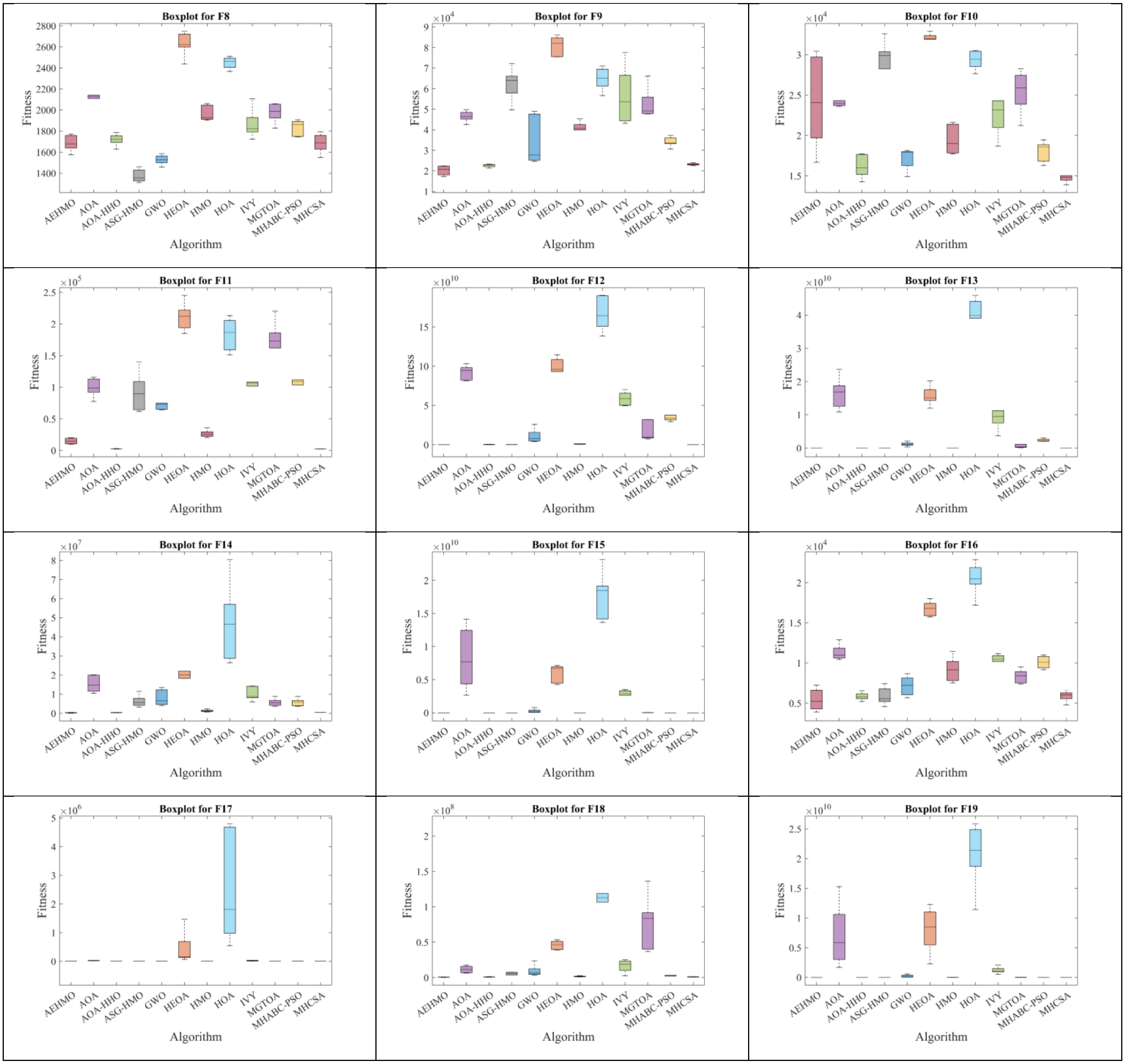
Boxplot for F8
Boxplot for F9
Boxplot for F10
Boxplot for F11
Boxplot for F12
Boxplot for F13
Boxplot for F14
Boxplot for F15
Boxplot for F16
Boxplot for F17
Boxplot for F18
Boxplot for F19
Fitness
Algorithm
AEHMO
AOA
AOA-HHO
ASG-HMO
GWO
HEOA
HMO
HOA
IVY
MGTOA
MHABC-PSO
MHCSA

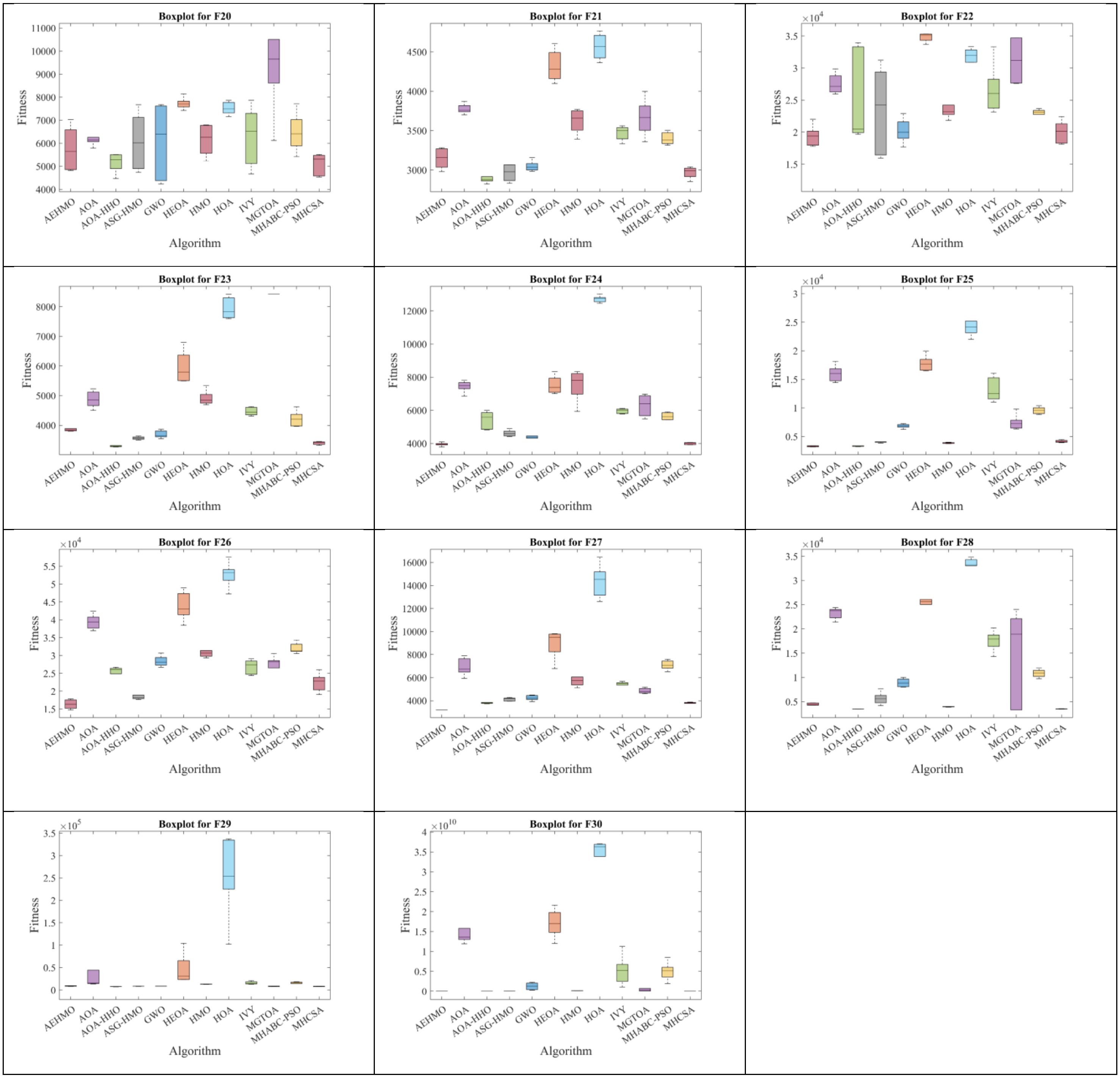

Figure E4 Boxplots of various algorithms using CEC2017, 100D.

Appendix F: Statistical CI and Effect Size

Table F1 Confidence intervals at various dimensions. (Part 1/8)

F	D	M	AEHMO	HMO	MGTOA	AOA	IVY	GWO	HEOA	HOA	MHCSA	AOA-HHO	MHABC-PSO	ASG-HMO
F1	10D	CI_Lower	1.00E+02	3.53E+02	1.93E+03	3.27E+07	7.44E+07	5.21E+05	6.10E+08	6.56E+09	3.17E+03	3.15E+03	3.14E+03	1.54E+03
F1	10D	CI_Upper	1.00E+02	5.83E+02	2.91E+03	1.34E+08	2.26E+08	6.31E+06	1.00E+09	8.38E+09	4.06E+03	5.14E+03	4.07E+03	3.44E+03
F1	10D	Margin_of_Error	7.69E-07	1.15E+02	4.90E+02	5.04E+07	7.60E+07	2.90E+06	1.97E+08	9.09E+08	4.46E+02	9.95E+02	4.68E+02	9.50E+02
F1	30D	CI_Lower	1.72E+03	2.73E+05	1.52E+09	1.76E+10	1.18E+10	1.61E+09	2.06E+10	4.26E+10	2.32E+03	4.10E+03	2.54E+09	1.51E+05
F1	30D	CI_Upper	2.99E+03	3.63E+05	2.41E+09	2.13E+10	1.64E+10	2.65E+09	2.44E+10	4.90E+10	3.78E+03	8.70E+03	3.46E+09	4.33E+05
F1	30D	Margin_of_Error	6.35E+02	4.50E+04	4.44E+08	1.84E+09	2.27E+09	5.22E+08	1.87E+09	3.18E+09	7.32E+02	2.30E+03	4.59E+08	1.41E+05
F1	50D	CI_Lower	1.96E+06	8.28E+06	3.19E+09	5.92E+10	3.52E+10	8.92E+09	5.96E+10	8.92E+10	6.97E+03	6.76E+03	2.07E+10	7.04E+06
F1	50D	CI_Upper	3.64E+06	1.08E+07	6.26E+09	6.66E+10	3.92E+10	1.07E+10	6.55E+10	9.72E+10	9.45E+03	8.83E+03	2.33E+10	9.52E+06
F1	50D	Margin_of_Error	8.39E+05	1.25E+06	1.54E+09	3.67E+09	1.99E+09	8.70E+08	2.98E+09	3.99E+09	1.24E+03	1.03E+03	1.30E+09	1.24E+06
F1	100D	CI_Lower	2.50E+09	5.84E+08	5.04E+10	1.83E+11	1.27E+11	4.84E+10	2.00E+11	2.40E+11	5.61E+04	6.77E+04	1.04E+11	6.35E+08
F1	100D	CI_Upper	3.24E+09	6.58E+08	5.56E+10	1.93E+11	1.40E+11	5.50E+10	2.11E+11	2.47E+11	7.14E+04	1.11E+05	1.11E+11	8.44E+08
F1	100D	Margin_of_Error	3.71E+08	3.66E+07	2.61E+09	4.87E+09	6.28E+09	3.28E+09	5.26E+09	3.50E+09	7.64E+03	2.18E+04	3.29E+09	1.04E+08
F3	10D	CI_Lower	3.00E+02	3.00E+02	3.00E+02	3.13E+03	3.53E+03	1.05E+03	1.08E+04	1.06E+04	3.00E+02	3.00E+02	3.83E+02	3.00E+02
F3	10D	CI_Upper	3.00E+02	3.00E+02	3.00E+02	4.68E+03	5.66E+03	2.31E+03	1.23E+04	1.27E+04	3.00E+02	3.00E+02	4.45E+02	3.01E+02
F3	10D	Margin_of_Error	0.00E+00	3.23E-03	2.21E-02	7.72E+02	1.06E+03	6.29E+02	7.54E+02	1.04E+03	2.57E-10	1.36E-09	3.09E+01	3.92E-01
F3	30D	CI_Lower	7.86E+03	4.03E+04	2.35E+04	6.43E+04	5.53E+04	4.69E+04	8.48E+04	7.72E+04	2.54E+03	3.50E+03	6.46E+04	6.66E+04
F3	30D	CI_Upper	9.18E+03	4.70E+04	3.04E+04	6.88E+04	6.32E+04	5.62E+04	8.80E+04	8.34E+04	3.49E+03	4.42E+03	6.90E+04	7.63E+04
F3	30D	Margin_of_Error	6.59E+02	3.38E+03	3.47E+03	2.26E+03	3.96E+03	4.66E+03	1.62E+03	3.08E+03	4.77E+02	4.62E+02	2.20E+03	4.83E+03
F3	50D	CI_Lower	2.96E+04	6.09E+04	1.16E+05	1.25E+05	1.22E+05	1.35E+05	1.95E+05	1.52E+05	3.60E+04	3.97E+04	1.42E+05	1.53E+05
F3	50D	CI_Upper	3.49E+04	7.14E+04	1.29E+05	1.34E+05	1.35E+05	1.54E+05	1.98E+05	1.66E+05	3.96E+04	4.42E+04	1.51E+05	1.60E+05
F3	50D	Margin_of_Error	2.65E+03	5.24E+03	6.46E+03	4.57E+03	6.35E+03	9.78E+03	1.57E+03	6.76E+03	1.84E+03	2.26E+03	4.56E+03	3.41E+03
F3	100D	CI_Lower	2.37E+05	3.23E+05	2.94E+05	3.15E+05	3.27E+05	4.24E+05	3.60E+05	3.16E+05	2.43E+05	2.42E+05	3.25E+05	3.24E+05
F3	100D	CI_Upper	2.52E+05	3.34E+05	3.25E+05	3.26E+05	3.49E+05	4.70E+05	3.67E+05	3.25E+05	2.60E+05	2.49E+05	3.28E+05	3.47E+05
F3	100D	Margin_of_Error	7.57E+03	5.16E+03	1.54E+04	5.34E+03	1.11E+04	2.28E+04	3.92E+03	4.27E+03	8.40E+03	3.40E+03	1.63E+03	1.19E+04
F4	10D	CI_Lower	4.00E+02	4.02E+02	4.03E+02	4.29E+02	4.32E+02	4.22E+02	4.41E+02	8.56E+02	4.01E+02	4.01E+02	4.03E+02	4.03E+02
F4	10D	CI_Upper	4.00E+02	4.04E+02	4.05E+02	4.43E+02	4.58E+02	4.36E+02	4.72E+02	1.16E+03	4.02E+02	4.01E+02	4.06E+02	4.05E+02
F4	10D	Margin_of_Error	1.76E-03	9.42E-01	1.17E+00	7.05E+00	1.26E+01	7.21E+00	1.54E+01	1.52E+02	2.26E-01	2.20E-01	1.17E+00	8.21E-01
F4	30D	CI_Lower	4.99E+02	5.32E+02	5.13E+02	2.18E+03	1.13E+03	5.97E+02	1.85E+03	8.52E+03	4.89E+02	4.84E+02	6.53E+02	5.03E+02
F4	30D	CI_Upper	5.12E+02	5.40E+02	5.37E+02	3.00E+03	2.15E+03	6.32E+02	2.46E+03	9.48E+03	5.04E+02	4.98E+02	7.36E+02	5.24E+02
F4	30D	Margin_of_Error	6.52E+00	3.74E+00	1.19E+01	4.09E+02	5.10E+02	1.75E+01	3.06E+02	4.84E+02	7.64E+00	7.28E+00	4.12E+01	1.06E+01
F4	50D	CI_Lower	5.06E+02	6.79E+02	1.12E+03	8.03E+03	5.78E+03	1.75E+03	1.06E+04	2.62E+04	6.07E+02	5.02E+02	2.80E+03	6.37E+02
F4	50D	CI_Upper	5.51E+02	7.06E+02	1.47E+03	9.69E+03	6.84E+03	2.04E+03	1.30E+04	2.88E+04	6.40E+02	5.70E+02	3.31E+03	6.86E+02
F4	50D	Margin_of_Error	2.25E+01	1.34E+01	1.74E+02	8.33E+02	5.32E+02	1.45E+02	1.18E+03	1.27E+03	1.65E+01	3.41E+01	2.53E+02	2.43E+01
F4	100D	CI_Lower	7.21E+02	1.19E+03	6.93E+03	3.41E+04	1.86E+04	5.40E+03	3.91E+04	7.98E+04	7.27E+02	1.42E+03	1.53E+04	1.27E+03
F4	100D	CI_Upper	7.50E+02	1.25E+03	9.16E+03	3.92E+04	2.23E+04	6.12E+03	4.41E+04	8.89E+04	7.68E+02	1.51E+03	1.64E+04	1.34E+03
F4	100D	Margin_of_Error	1.42E+01	3.29E+01	1.11E+03	2.58E+03	1.88E+03	3.57E+02	2.49E+03	4.55E+03	2.05E+01	4.64E+01	5.35E+02	3.22E+01
F5	10D	CI_Lower	5.21E+02	5.37E+02	5.26E+02	5.30E+02	5.24E+02	5.13E+02	5.76E+02	5.54E+02	5.20E+02	5.16E+02	5.51E+02	5.15E+02
F5	10D	CI_Upper	5.27E+02	5.45E+02	5.36E+02	5.36E+02	5.31E+02	5.19E+02	5.83E+02	5.62E+02	5.25E+02	5.21E+02	5.60E+02	5.19E+02
F5	10D	Margin_of_Error	2.99E+00	3.94E+00	4.93E+00	3.10E+00	3.09E+00	3.00E+00	3.57E+00	3.93E+00	2.18E+00	2.36E+00	4.64E+00	1.67E+00
F5	30D	CI_Lower	6.43E+02	7.47E+02	7.10E+02	7.46E+02	7.09E+02	6.10E+02	8.81E+02	8.32E+02	6.78E+02	6.64E+02	7.63E+02	5.66E+02
F5	30D	CI_Upper	6.68E+02	7.78E+02	7.31E+02	7.67E+02	7.35E+02	6.38E+02	9.09E+02	8.53E+02	7.13E+02	7.01E+02	7.75E+02	5.74E+02
F5	30D	Margin_of_Error	1.25E+01	1.55E+01	1.05E+01	1.07E+01	1.29E+01	1.43E+01	1.40E+01	1.06E+01	1.71E+01	1.85E+01	5.86E+00	3.89E+00
F5	50D	CI_Lower	6.90E+02	8.86E+02	8.53E+02	9.73E+02	8.62E+02	7.02E+02	1.17E+03	1.09E+03	8.19E+02	8.12E+02	8.53E+02	8.03E+02
F5	50D	CI_Upper	7.11E+02	9.24E+02	8.80E+02	9.96E+02	8.86E+02	7.21E+02	1.22E+03	1.12E+03	8.34E+02	8.41E+02	8.72E+02	8.22E+02
F5	50D	Margin_of_Error	1.05E+01	1.89E+01	1.36E+01	1.16E+01	1.17E+01	9.33E+00	2.13E+01	1.40E+01	7.17E+00	1.43E+01	9.37E+00	9.05E+00
F5	100D	CI_Lower	1.08E+03	1.40E+03	1.48E+03	1.75E+03	1.55E+03	1.17E+03	2.08E+03	1.94E+03	1.31E+03	1.29E+03	1.37E+03	1.19E+03

Table F1 Confidence intervals at various dimensions. (Part 2/8)

F	D	M	AEHMO	HMO	MGTOA	AOA	IVY	GWO	HEOA	HOA	MHCSA	AOA-HHO	MHABC-PSO	ASG-HMO
F5	100D	CI_Upper	1.13E+03	1.44E+03	1.55E+03	1.77E+03	1.61E+03	1.22E+03	2.13E+03	1.98E+03	1.32E+03	1.33E+03	1.39E+03	1.23E+03
F5	100D	Margin_of_Error	2.25E+01	2.25E+01	3.26E+01	1.06E+01	3.10E+01	2.43E+01	2.34E+01	2.08E+01	8.22E+00	2.04E+01	7.85E+00	1.97E+01
F6	10D	CI_Lower	6.00E+02	6.20E+02	6.01E+02	6.11E+02	6.06E+02	6.00E+02	6.52E+02	6.32E+02	6.01E+02	6.00E+02	6.28E+02	6.00E+02
F6	10D	CI_Upper	6.00E+02	6.30E+02	6.01E+02	6.17E+02	6.10E+02	6.01E+02	6.58E+02	6.38E+02	6.03E+02	6.02E+02	6.38E+02	6.01E+02
F6	10D	Margin_of_Error	1.47E-01	5.25E+00	4.08E-01	2.92E+00	2.15E+00	2.76E-01	3.14E+00	2.89E+00	1.12E+00	6.53E-01	4.74E+00	3.84E-01
F6	30D	CI_Lower	6.10E+02	6.60E+02	6.27E+02	6.39E+02	6.34E+02	6.19E+02	6.88E+02	6.72E+02	6.30E+02	6.25E+02	6.58E+02	6.12E+02
F6	30D	CI_Upper	6.14E+02	6.64E+02	6.34E+02	6.43E+02	6.40E+02	6.24E+02	6.91E+02	6.76E+02	6.36E+02	6.32E+02	6.64E+02	6.16E+02
F6	30D	Margin_of_Error	2.06E+00	1.89E+00	3.68E+00	1.96E+00	2.95E+00	2.63E+00	1.74E+00	1.98E+00	3.16E+00	3.10E+00	3.16E+00	1.94E+00
F6	50D	CI_Lower	6.17E+02	6.65E+02	6.61E+02	6.61E+02	6.51E+02	6.19E+02	6.96E+02	6.80E+02	6.41E+02	6.44E+02	6.67E+02	6.38E+02
F6	50D	CI_Upper	6.20E+02	6.68E+02	6.64E+02	6.64E+02	6.54E+02	6.23E+02	7.00E+02	6.86E+02	6.46E+02	6.48E+02	6.70E+02	6.50E+02
F6	50D	Margin_of_Error	1.49E+00	1.55E+00	1.53E+00	1.30E+00	1.39E+00	1.78E+00	2.07E+00	3.04E+00	2.44E+00	2.42E+00	1.50E+00	5.74E+00
F6	100D	CI_Lower	6.41E+02	6.76E+02	6.69E+02	6.81E+02	6.71E+02	6.54E+02	7.14E+02	6.98E+02	6.58E+02	6.58E+02	6.73E+02	6.44E+02
F6	100D	CI_Upper	6.44E+02	6.79E+02	6.74E+02	6.84E+02	6.75E+02	6.58E+02	7.16E+02	6.99E+02	6.60E+02	6.60E+02	6.74E+02	6.46E+02
F6	100D	Margin_of_Error	1.67E+00	1.07E+00	2.49E+00	1.34E+00	2.00E+00	1.81E+00	1.15E+00	7.91E-01	8.98E-01	6.27E-01	9.08E-01	9.67E-01
F7	10D	CI_Lower	7.31E+02	7.76E+02	7.45E+02	7.58E+02	7.61E+02	7.22E+02	8.03E+02	7.61E+02	7.50E+02	7.50E+02	7.80E+02	7.25E+02
F7	10D	CI_Upper	7.37E+02	7.94E+02	7.59E+02	7.66E+02	7.72E+02	7.26E+02	8.19E+02	7.75E+02	7.61E+02	7.62E+02	7.90E+02	7.35E+02
F7	10D	Margin_of_Error	3.23E+00	9.21E+00	6.74E+00	4.04E+00	5.29E+00	2.15E+00	7.92E+00	6.82E+00	5.59E+00	6.19E+00	4.84E+00	5.22E+00
F7	30D	CI_Lower	8.47E+02	1.19E+03	9.97E+02	1.10E+03	1.03E+03	9.59E+02	1.37E+03	1.25E+03	9.09E+02	9.45E+02	1.11E+03	8.64E+02
F7	30D	CI_Upper	8.71E+02	1.28E+03	1.03E+03	1.14E+03	1.05E+03	9.98E+02	1.42E+03	1.28E+03	9.33E+02	1.01E+03	1.14E+03	8.83E+02
F7	30D	Margin_of_Error	1.20E+01	4.95E+01	1.86E+01	1.97E+01	1.06E+01	1.96E+01	2.41E+01	1.46E+01	1.18E+01	3.44E+01	1.77E+01	9.84E+00
F7	50D	CI_Lower	1.03E+03	1.70E+03	1.38E+03	1.69E+03	1.50E+03	1.09E+03	2.02E+03	1.85E+03	1.23E+03	1.24E+03	1.54E+03	1.26E+03
F7	50D	CI_Upper	1.06E+03	1.79E+03	1.55E+03	1.72E+03	1.55E+03	1.15E+03	2.05E+03	1.89E+03	1.31E+03	1.36E+03	1.58E+03	1.35E+03
F7	50D	Margin_of_Error	1.63E+01	4.12E+01	8.26E+01	1.41E+01	2.35E+01	3.07E+01	1.65E+01	2.00E+01	3.62E+01	5.90E+01	1.82E+01	4.15E+01
F7	100D	CI_Lower	1.82E+03	3.41E+03	3.05E+03	3.38E+03	2.90E+03	2.07E+03	4.02E+03	3.72E+03	2.58E+03	2.46E+03	3.10E+03	2.69E+03
F7	100D	CI_Upper	1.92E+03	3.55E+03	3.47E+03	3.47E+03	2.97E+03	2.21E+03	4.05E+03	3.80E+03	2.67E+03	2.66E+03	3.18E+03	2.77E+03
F7	100D	Margin_of_Error	5.04E+01	7.41E+01	2.12E+02	4.57E+01	3.38E+01	6.80E+01	1.50E+01	3.96E+01	4.57E+01	9.71E+01	3.82E+01	4.03E+01
F8	10D	CI_Lower	8.13E+02	8.28E+02	8.17E+02	8.23E+02	8.23E+02	8.13E+02	8.49E+02	8.29E+02	8.25E+02	8.27E+02	8.22E+02	8.14E+02
F8	10D	CI_Upper	8.16E+02	8.38E+02	8.23E+02	8.26E+02	8.30E+02	8.16E+02	8.56E+02	8.33E+02	8.29E+02	8.28E+02	8.26E+02	8.18E+02
F8	10D	Margin_of_Error	1.76E+00	4.87E+00	2.84E+00	1.66E+00	3.31E+00	1.59E+00	3.49E+00	1.88E+00	2.11E+00	6.91E-01	1.58E+00	1.89E+00
F8	30D	CI_Lower	8.95E+02	9.64E+02	9.31E+02	9.76E+02	9.46E+02	9.07E+02	1.09E+03	1.07E+03	9.21E+02	9.39E+02	9.86E+02	8.83E+02
F8	30D	CI_Upper	9.09E+02	9.82E+02	9.57E+02	1.01E+03	9.64E+02	9.24E+02	1.12E+03	1.08E+03	9.33E+02	9.52E+02	9.93E+02	9.00E+02
F8	30D	Margin_of_Error	7.05E+00	9.09E+00	1.29E+01	1.79E+01	8.91E+00	8.14E+00	1.42E+01	5.60E+00	6.17E+00	6.60E+00	3.39E+00	8.69E+00
F8	50D	CI_Lower	1.08E+03	1.16E+03	1.19E+03	1.25E+03	1.19E+03	1.01E+03	1.55E+03	1.41E+03	1.11E+03	1.12E+03	1.17E+03	9.87E+02
F8	50D	CI_Upper	1.13E+03	1.19E+03	1.22E+03	1.29E+03	1.22E+03	1.03E+03	1.58E+03	1.44E+03	1.12E+03	1.15E+03	1.19E+03	1.01E+03
F8	50D	Margin_of_Error	2.33E+01	1.51E+01	1.37E+01	2.00E+01	1.37E+01	9.59E+00	1.55E+01	1.93E+01	6.42E+00	1.36E+01	1.09E+01	1.39E+01
F8	100D	CI_Lower	1.51E+03	1.94E+03	1.94E+03	2.11E+03	1.82E+03	1.66E+03	2.58E+03	2.43E+03	1.65E+03	1.70E+03	1.81E+03	1.35E+03
F8	100D	CI_Upper	1.54E+03	1.99E+03	2.01E+03	2.14E+03	1.91E+03	1.71E+03	2.66E+03	2.47E+03	1.72E+03	1.74E+03	1.86E+03	1.39E+03
F8	100D	Margin_of_Error	1.61E+01	2.57E+01	3.14E+01	1.67E+01	4.87E+01	2.65E+01	3.96E+01	1.96E+01	3.29E+01	1.95E+01	2.61E+01	2.11E+01
F9	10D	CI_Lower	9.00E+02	1.11E+03	9.09E+02	9.95E+02	9.55E+02	9.08E+02	1.50E+03	1.15E+03	9.21E+02	9.15E+02	1.43E+03	9.06E+02
F9	10D	CI_Upper	9.01E+02	1.23E+03	9.59E+02	1.12E+03	1.03E+03	9.19E+02	1.75E+03	1.26E+03	9.72E+02	9.61E+02	1.57E+03	9.09E+02
F9	10D	Margin_of_Error	4.18E-01	5.88E+01	2.52E+01	6.35E+01	3.48E+01	5.67E+00	1.24E+02	5.26E+01	2.56E+01	2.31E+01	6.94E+01	1.84E+00
F9	30D	CI_Lower	1.56E+03	4.85E+03	3.88E+03	5.92E+03	4.64E+03	2.88E+03	1.22E+04	6.85E+03	3.27E+03	3.87E+03	5.62E+03	1.80E+03
F9	30D	CI_Upper	2.16E+03	5.43E+03	4.91E+03	6.59E+03	5.24E+03	3.50E+03	1.27E+04	7.97E+03	3.95E+03	4.11E+03	5.86E+03	2.27E+03
F9	30D	Margin_of_Error	2.98E+02	2.93E+02	5.16E+02	3.31E+02	3.01E+02	3.08E+02	2.32E+02	5.59E+02	3.41E+02	1.19E+02	1.20E+02	2.34E+02
F9	50D	CI_Lower	3.87E+03	1.63E+04	1.72E+04	1.78E+04	1.80E+04	8.60E+03	3.66E+04	3.03E+04	1.01E+04	1.02E+04	1.74E+04	2.07E+04
F9	50D	CI_Upper	6.10E+03	1.70E+04	2.11E+04	1.92E+04	2.21E+04	1.28E+04	3.85E+04	3.19E+04	1.08E+04	1.10E+04	2.28E+04	2.46E+04

Table F1 Confidence intervals at various dimensions. (Part 3/8)

F	D	M	AEHMO	HMO	MGTOA	AOA	IVY	GWO	HEOA	HOA	MHCSA	AOA-HHO	MHABC-PSO	ASG-HMO
F9	50D	Margin_of_Error	1.12E+03	3.49E+02	1.99E+03	6.94E+02	2.02E+03	2.12E+03	9.15E+02	8.20E+02	3.65E+02	3.73E+02	2.67E+03	1.94E+03
F9	100D	CI_Lower	1.94E+04	3.97E+04	5.01E+04	4.55E+04	5.17E+04	2.95E+04	7.94E+04	6.29E+04	2.31E+04	2.23E+04	3.32E+04	5.96E+04
F9	100D	CI_Upper	2.11E+04	4.19E+04	5.53E+04	4.74E+04	6.13E+04	3.78E+04	8.27E+04	6.67E+04	2.35E+04	2.28E+04	3.49E+04	6.51E+04
F9	100D	Margin_of_Error	8.29E+02	1.13E+03	2.61E+03	9.38E+02	4.84E+03	4.12E+03	1.65E+03	1.93E+03	1.82E+02	2.74E+02	8.43E+02	2.76E+03
F10	10D	CI_Lower	1.43E+03	1.75E+03	1.56E+03	1.54E+03	1.67E+03	1.41E+03	2.44E+03	2.38E+03	1.52E+03	1.55E+03	1.83E+03	1.33E+03
F10	10D	CI_Upper	1.66E+03	2.05E+03	1.80E+03	1.84E+03	1.89E+03	1.72E+03	2.66E+03	2.50E+03	1.66E+03	1.60E+03	2.03E+03	1.51E+03
F10	10D	Margin_of_Error	1.16E+02	1.52E+02	1.18E+02	1.50E+02	1.08E+02	1.54E+02	1.09E+02	5.83E+01	7.34E+01	2.50E+01	9.95E+01	9.10E+01
F10	30D	CI_Lower	3.44E+03	5.06E+03	5.08E+03	5.22E+03	7.40E+03	4.50E+03	8.85E+03	7.71E+03	4.98E+03	3.86E+03	5.00E+03	6.68E+03
F10	30D	CI_Upper	4.11E+03	5.40E+03	5.72E+03	5.67E+03	8.36E+03	4.94E+03	9.21E+03	7.88E+03	5.74E+03	4.57E+03	6.21E+03	7.70E+03
F10	30D	Margin_of_Error	3.38E+02	1.70E+02	3.17E+02	2.24E+02	4.83E+02	2.22E+02	1.81E+02	8.57E+01	3.82E+02	3.53E+02	6.04E+02	5.07E+02
F10	50D	CI_Lower	5.78E+03	8.97E+03	1.08E+04	8.69E+03	1.12E+04	7.00E+03	1.40E+04	1.36E+04	6.64E+03	7.77E+03	7.78E+03	1.07E+04
F10	50D	CI_Upper	6.11E+03	9.63E+03	1.19E+04	9.59E+03	1.28E+04	7.92E+03	1.48E+04	1.43E+04	7.62E+03	8.38E+03	8.21E+03	1.30E+04
F10	50D	Margin_of_Error	1.61E+02	3.28E+02	5.62E+02	4.50E+02	7.58E+02	4.60E+02	3.89E+02	3.16E+02	4.89E+02	3.08E+02	2.18E+02	1.16E+03
F10	100D	CI_Lower	2.20E+04	1.88E+04	2.45E+04	2.39E+04	2.21E+04	1.67E+04	3.16E+04	2.90E+04	1.46E+04	1.56E+04	1.76E+04	2.60E+04
F10	100D	CI_Upper	2.62E+04	2.01E+04	2.64E+04	2.48E+04	2.51E+04	1.77E+04	3.22E+04	2.97E+04	1.53E+04	1.66E+04	1.85E+04	3.00E+04
F10	100D	Margin_of_Error	2.13E+03	6.37E+02	9.74E+02	4.23E+02	1.55E+03	4.75E+02	2.98E+02	3.93E+02	3.61E+02	5.04E+02	4.48E+02	2.00E+03
F11	10D	CI_Lower	1.11E+03	1.15E+03	1.12E+03	1.12E+03	1.18E+03	1.13E+03	1.27E+03	3.36E+03	1.12E+03	1.12E+03	1.12E+03	1.11E+03
F11	10D	CI_Upper	1.12E+03	1.17E+03	1.12E+03	1.13E+03	1.24E+03	1.14E+03	1.39E+03	4.69E+03	1.12E+03	1.13E+03	1.13E+03	1.11E+03
F11	10D	Margin_of_Error	2.02E+00	6.77E+00	3.81E+00	5.13E+00	3.12E+01	3.46E+00	5.81E+01	6.64E+02	9.60E-01	2.98E+00	3.48E+00	3.34E+00
F11	30D	CI_Lower	1.17E+03	1.24E+03	1.27E+03	2.78E+03	3.93E+03	1.77E+03	6.59E+03	6.81E+03	1.21E+03	1.19E+03	1.56E+03	1.30E+03
F11	30D	CI_Upper	1.19E+03	1.31E+03	1.31E+03	3.39E+03	4.85E+03	2.28E+03	8.30E+03	8.42E+03	1.24E+03	1.20E+03	1.73E+03	1.34E+03
F11	30D	Margin_of_Error	9.81E+00	3.42E+01	1.56E+01	3.03E+02	4.58E+02	2.53E+02	8.57E+02	8.07E+02	1.92E+01	7.56E+00	8.59E+01	2.03E+01
F11	50D	CI_Lower	1.34E+03	1.49E+03	4.69E+05	9.66E+03	6.29E+03	5.87E+03	1.62E+04	2.01E+04	1.29E+03	1.25E+03	3.45E+03	1.77E+03
F11	50D	CI_Upper	1.37E+03	1.54E+03	1.61E+06	1.13E+04	8.28E+03	7.63E+03	1.74E+04	2.17E+04	1.32E+03	1.28E+03	3.82E+03	1.90E+03
F11	50D	Margin_of_Error	1.84E+01	2.72E+01	5.72E+05	8.11E+02	9.93E+02	8.78E+02	6.21E+02	8.03E+02	1.80E+01	1.74E+01	1.82E+02	6.63E+01
F11	100D	CI_Lower	1.29E+04	2.43E+04	1.54E+05	9.40E+04	1.02E+05	7.07E+04	2.04E+05	1.74E+05	2.41E+03	2.49E+03	1.02E+05	8.17E+04
F11	100D	CI_Upper	1.63E+04	2.84E+04	1.83E+05	1.04E+05	1.09E+05	8.45E+04	2.19E+05	1.93E+05	2.50E+03	2.69E+03	1.14E+05	1.03E+05
F11	100D	Margin_of_Error	1.67E+03	2.06E+03	1.47E+04	5.25E+03	3.57E+03	6.92E+03	7.66E+03	9.41E+03	4.49E+01	9.86E+01	5.87E+03	1.06E+04
F12	10D	CI_Lower	1.38E+03	2.17E+05	6.83E+03	5.19E+05	6.05E+05	4.36E+05	3.41E+06	1.38E+07	6.67E+03	7.95E+03	1.81E+06	1.25E+04
F12	10D	CI_Upper	1.43E+03	4.55E+05	1.08E+04	1.09E+06	1.28E+06	1.29E+06	6.28E+06	2.63E+07	8.53E+03	1.21E+04	4.05E+06	1.77E+04
F12	10D	Margin_of_Error	2.73E+01	1.19E+05	1.99E+03	2.87E+05	3.39E+05	4.28E+05	1.43E+06	6.27E+06	9.30E+02	2.06E+03	1.12E+06	2.60E+03
F12	30D	CI_Lower	4.44E+04	2.78E+07	5.22E+06	8.74E+08	6.31E+08	1.12E+08	1.61E+09	8.84E+09	4.84E+05	1.12E+06	6.38E+07	1.03E+06
F12	30D	CI_Upper	6.16E+04	4.23E+07	7.45E+06	1.45E+09	1.13E+09	2.06E+08	2.33E+09	1.04E+10	9.34E+05	1.56E+06	8.84E+07	1.48E+06
F12	30D	Margin_of_Error	8.60E+03	7.25E+06	1.12E+06	2.88E+08	2.49E+08	4.73E+07	3.58E+08	7.88E+08	2.25E+05	2.18E+05	1.23E+07	2.29E+05
F12	50D	CI_Lower	3.37E+06	1.25E+08	7.10E+08	1.08E+10	7.59E+09	6.56E+08	1.81E+10	5.80E+10	3.98E+06	3.58E+06	1.20E+09	2.12E+07
F12	50D	CI_Upper	6.49E+06	2.04E+08	2.40E+09	2.09E+10	9.17E+09	1.69E+09	2.16E+10	7.08E+10	5.01E+06	4.87E+06	1.96E+09	4.29E+07
F12	50D	Margin_of_Error	1.56E+06	3.90E+07	8.46E+08	5.05E+09	7.94E+08	5.17E+08	1.74E+09	6.42E+09	5.16E+05	6.46E+05	3.80E+08	1.08E+07
F12	100D	CI_Lower	2.35E+07	7.20E+08	1.23E+10	8.92E+10	5.59E+10	8.03E+09	8.90E+10	1.59E+11	2.27E+07	2.22E+08	3.34E+10	1.38E+08
F12	100D	CI_Upper	2.86E+07	9.72E+08	2.09E+10	9.56E+10	6.18E+10	1.41E+10	1.02E+11	1.74E+11	3.28E+07	3.35E+08	3.85E+10	2.43E+08
F12	100D	Margin_of_Error	2.54E+06	1.26E+08	4.33E+09	3.19E+09	2.93E+09	3.02E+09	6.32E+09	7.46E+09	5.08E+06	5.67E+07	2.52E+09	5.26E+07
F13	10D	CI_Lower	1.31E+03	1.04E+04	7.36E+03	5.40E+03	1.03E+04	6.00E+03	5.05E+04	1.63E+04	5.62E+03	6.29E+03	1.24E+04	5.67E+03
F13	10D	CI_Upper	1.32E+03	1.59E+04	1.03E+04	6.72E+03	1.63E+04	7.19E+03	8.14E+04	4.94E+04	1.01E+04	1.03E+04	2.08E+04	1.11E+04
F13	10D	Margin_of_Error	1.09E+00	2.79E+03	1.45E+03	6.60E+02	3.04E+03	5.95E+02	1.55E+04	1.65E+04	2.26E+03	2.00E+03	4.24E+03	2.70E+03
F13	30D	CI_Lower	6.27E+03	8.92E+04	7.03E+04	1.85E+07	3.88E+07	1.58E+05	7.64E+08	3.50E+09	1.00E+04	1.03E+04	2.87E+04	2.19E+04
F13	30D	CI_Upper	1.30E+04	1.67E+05	8.99E+04	1.49E+08	1.22E+08	4.05E+05	1.46E+09	4.91E+09	1.70E+04	1.75E+04	4.07E+04	3.72E+04
F13	30D	Margin_of_Error	3.35E+03	3.89E+04	9.82E+03	6.53E+07	4.18E+07	1.23E+05	3.49E+08	7.07E+08	3.47E+03	3.57E+03	6.00E+03	7.66E+03

Table F1 Confidence intervals at various dimensions. (Part 4/8)

F	D	M	AEHMO	HMO	MGTOA	AOA	IVY	GWO	HEOA	HOA	MHCSA	AOA-HHO	MHABC-PSO	ASG-HMO
F13	50D	CI_Lower	5.16E+03	6.26E+04	3.78E+06	5.30E+09	1.01E+09	7.57E+07	2.33E+09	3.85E+10	9.65E+03	1.04E+04	1.52E+05	7.10E+04
F13	50D	CI_Upper	6.91E+03	1.04E+05	1.29E+07	9.57E+09	2.47E+09	1.74E+08	4.53E+09	4.59E+10	1.37E+04	1.70E+04	1.61E+06	1.08E+05
F13	50D	Margin_of_Error	8.73E+02	2.05E+04	4.58E+06	2.13E+09	7.30E+08	4.93E+07	1.10E+09	3.71E+09	2.02E+03	3.30E+03	7.28E+05	1.84E+04
F13	100D	CI_Lower	1.04E+04	1.47E+05	4.61E+08	1.50E+10	7.78E+09	9.42E+08	1.47E+10	3.71E+10	1.10E+04	2.14E+04	2.20E+09	3.74E+05
F13	100D	CI_Upper	1.35E+04	2.15E+05	1.41E+09	1.83E+10	9.86E+09	1.40E+09	1.67E+10	4.17E+10	1.26E+04	2.40E+04	2.50E+09	6.45E+05
F13	100D	Margin_of_Error	1.54E+03	3.40E+04	4.73E+08	1.65E+09	1.04E+09	2.28E+08	1.02E+09	2.30E+09	7.70E+02	1.28E+03	1.49E+08	1.36E+05
F14	10D	CI_Lower	1.42E+03	1.48E+03	1.46E+03	3.17E+03	1.61E+03	2.59E+03	2.21E+03	2.48E+03	1.75E+03	1.73E+03	3.60E+03	1.50E+03
F14	10D	CI_Upper	1.42E+03	1.51E+03	1.48E+03	4.15E+03	2.67E+03	4.02E+03	2.98E+03	3.45E+03	2.00E+03	2.10E+03	5.72E+03	1.52E+03
F14	10D	Margin_of_Error	8.54E-01	1.38E+01	8.55E+00	4.87E+02	5.29E+02	7.13E+02	3.89E+02	4.88E+02	1.23E+02	1.81E+02	1.06E+03	9.80E+00
F14	30D	CI_Lower	1.50E+03	2.25E+04	5.77E+04	4.13E+05	1.38E+06	3.71E+05	1.84E+06	1.59E+06	1.40E+04	2.16E+04	7.21E+05	2.45E+04
F14	30D	CI_Upper	1.51E+03	5.19E+04	1.45E+05	8.47E+05	2.82E+06	9.74E+05	2.57E+06	2.36E+06	2.13E+04	3.32E+04	1.15E+06	4.66E+04
F14	30D	Margin_of_Error	5.13E+00	1.47E+04	4.36E+04	2.17E+05	7.19E+05	3.02E+05	3.65E+05	3.82E+05	3.69E+03	5.79E+03	2.14E+05	1.10E+04
F14	50D	CI_Lower	1.77E+03	1.25E+05	7.26E+05	3.76E+06	2.36E+06	1.68E+06	1.53E+07	6.69E+07	7.82E+04	3.56E+04	1.14E+06	1.90E+05
F14	50D	CI_Upper	1.82E+03	2.36E+05	1.10E+06	6.28E+06	5.46E+06	2.46E+06	2.42E+07	1.10E+08	1.47E+05	5.73E+04	1.94E+06	3.36E+05
F14	50D	Margin_of_Error	2.15E+01	5.55E+04	1.90E+05	1.26E+06	1.55E+06	3.91E+05	4.46E+06	2.15E+07	3.42E+04	1.08E+04	4.00E+05	7.32E+04
F14	100D	CI_Lower	1.95E+05	1.18E+06	5.08E+06	1.38E+07	8.82E+06	6.47E+06	1.98E+07	4.05E+07	4.14E+05	3.26E+05	5.11E+06	5.24E+06
F14	100D	CI_Upper	3.01E+05	1.58E+06	6.46E+06	1.67E+07	1.13E+07	9.33E+06	2.29E+07	5.48E+07	5.00E+05	3.81E+05	6.49E+06	7.37E+06
F14	100D	Margin_of_Error	5.30E+04	2.03E+05	6.94E+05	1.49E+06	1.22E+06	1.43E+06	1.55E+06	7.17E+06	4.29E+04	2.74E+04	6.91E+05	1.06E+06
F15	10D	CI_Lower	1.50E+03	1.60E+03	1.67E+03	2.52E+03	3.88E+03	3.69E+03	1.13E+04	8.47E+03	1.82E+03	1.94E+03	3.97E+03	1.82E+03
F15	10D	CI_Upper	1.50E+03	1.72E+03	1.74E+03	3.44E+03	4.98E+03	4.88E+03	1.24E+04	1.04E+04	2.22E+03	2.26E+03	5.01E+03	1.97E+03
F15	10D	Margin_of_Error	1.85E-01	5.60E+01	3.43E+01	4.61E+02	5.48E+02	5.95E+02	5.52E+02	9.46E+02	2.01E+02	1.60E+02	5.20E+02	7.26E+01
F15	30D	CI_Lower	1.64E+03	2.48E+04	6.74E+03	2.73E+05	1.59E+06	3.77E+05	1.30E+07	6.79E+07	2.63E+03	3.76E+03	1.47E+04	6.45E+03
F15	30D	CI_Upper	1.68E+03	5.50E+04	8.99E+03	9.15E+05	2.68E+06	1.23E+06	2.53E+07	2.93E+08	3.93E+03	4.98E+03	1.59E+04	1.09E+04
F15	30D	Margin_of_Error	2.06E+01	1.51E+04	1.13E+03	3.21E+05	5.43E+05	4.29E+05	6.14E+06	1.13E+08	6.51E+02	6.11E+02	5.88E+02	2.21E+03
F15	50D	CI_Lower	8.59E+03	3.01E+04	3.73E+04	6.63E+08	3.78E+07	1.09E+07	1.38E+09	5.69E+09	1.08E+04	1.57E+04	2.97E+04	1.30E+04
F15	50D	CI_Upper	1.46E+04	3.99E+04	5.45E+04	1.13E+09	7.76E+07	1.30E+08	1.88E+09	7.22E+09	1.54E+04	1.92E+04	3.43E+04	2.60E+04
F15	50D	Margin_of_Error	3.03E+03	4.88E+03	8.63E+03	2.33E+08	1.99E+07	5.94E+07	2.49E+08	7.64E+08	2.27E+03	1.73E+03	2.29E+03	6.50E+03
F15	100D	CI_Lower	4.89E+03	3.77E+04	1.39E+07	6.43E+09	2.09E+09	1.53E+08	5.55E+09	1.66E+10	4.46E+03	4.95E+03	1.67E+05	1.31E+05
F15	100D	CI_Upper	5.20E+03	4.58E+04	2.05E+07	9.87E+09	3.00E+09	3.69E+08	6.50E+09	1.91E+10	5.98E+03	5.76E+03	4.06E+05	3.48E+05
F15	100D	Margin_of_Error	1.56E+02	4.05E+03	3.31E+06	1.72E+09	4.55E+08	1.08E+08	4.71E+08	1.25E+09	7.57E+02	4.04E+02	1.20E+05	1.09E+05
F16	10D	CI_Lower	1.60E+03	1.81E+03	1.74E+03	1.81E+03	1.78E+03	1.68E+03	2.02E+03	1.94E+03	1.75E+03	1.77E+03	1.90E+03	1.68E+03
F16	10D	CI_Upper	1.64E+03	1.90E+03	1.82E+03	1.89E+03	1.87E+03	1.72E+03	2.14E+03	2.04E+03	1.85E+03	1.83E+03	1.95E+03	1.77E+03
F16	10D	Margin_of_Error	1.76E+01	4.93E+01	3.97E+01	3.98E+01	4.69E+01	2.01E+01	6.07E+01	4.98E+01	4.88E+01	3.34E+01	2.59E+01	4.45E+01
F16	30D	CI_Lower	2.36E+03	3.42E+03	2.60E+03	2.75E+03	2.84E+03	2.24E+03	4.06E+03	5.23E+03	2.54E+03	2.80E+03	3.17E+03	2.45E+03
F16	30D	CI_Upper	2.47E+03	3.78E+03	2.78E+03	2.92E+03	2.95E+03	2.39E+03	4.45E+03	6.61E+03	2.70E+03	2.99E+03	3.55E+03	2.59E+03
F16	30D	Margin_of_Error	5.53E+01	1.80E+02	8.87E+01	8.45E+01	5.67E+01	7.47E+01	1.92E+02	6.86E+02	8.28E+01	9.28E+01	1.89E+02	6.75E+01
F16	50D	CI_Lower	2.66E+03	4.72E+03	3.15E+03	4.05E+03	3.96E+03	3.11E+03	6.89E+03	8.03E+03	3.04E+03	2.86E+03	3.49E+03	3.37E+03
F16	50D	CI_Upper	2.78E+03	5.11E+03	3.48E+03	4.29E+03	4.35E+03	3.37E+03	7.42E+03	8.85E+03	3.22E+03	3.00E+03	3.66E+03	3.62E+03
F16	50D	Margin_of_Error	6.19E+01	1.99E+02	1.68E+02	1.18E+02	1.94E+02	1.29E+02	2.68E+02	4.08E+02	9.08E+01	6.87E+01	8.30E+01	1.23E+02
F16	100D	CI_Lower	4.94E+03	8.67E+03	8.05E+03	1.10E+04	1.00E+04	6.73E+03	1.64E+04	1.98E+04	5.65E+03	5.66E+03	9.81E+03	5.45E+03
F16	100D	CI_Upper	5.90E+03	9.72E+03	8.65E+03	1.16E+04	1.06E+04	7.56E+03	1.71E+04	2.11E+04	6.11E+03	6.02E+03	1.04E+04	6.22E+03
F16	100D	Margin_of_Error	4.80E+02	5.26E+02	2.96E+02	3.32E+02	3.22E+02	4.16E+02	3.19E+02	6.93E+02	2.31E+02	1.79E+02	2.78E+02	3.84E+02
F17	10D	CI_Lower	1.72E+03	1.77E+03	1.77E+03	1.75E+03	1.76E+03	1.75E+03	1.84E+03	1.78E+03	1.76E+03	1.74E+03	1.76E+03	1.75E+03
F17	10D	CI_Upper	1.73E+03	1.78E+03	1.80E+03	1.76E+03	1.77E+03	1.76E+03	1.87E+03	1.79E+03	1.77E+03	1.76E+03	1.77E+03	1.77E+03
F17	10D	Margin_of_Error	5.02E+00	9.19E+00	1.16E+01	2.94E+00	6.52E+00	6.98E+00	1.81E+01	5.15E+00	7.22E+00	1.01E+01	5.28E+00	8.37E+00
F17	30D	CI_Lower	1.87E+03	2.35E+03	2.30E+03	2.14E+03	2.32E+03	1.94E+03	2.69E+03	4.03E+03	2.10E+03	2.12E+03	2.44E+03	2.21E+03

Table F1 Confidence intervals at various dimensions. (Part 5/8)

F	D	M	AEHMO	HMO	MGTOA	AOA	IVY	GWO	HEOA	HOA	MHCSA	AOA-HHO	MHABC-PSO	ASG-HMO
F17	30D	CI_Upper	1.96E+03	2.49E+03	2.48E+03	2.28E+03	2.54E+03	2.06E+03	2.94E+03	5.24E+03	2.24E+03	2.37E+03	2.65E+03	2.28E+03
F17	30D	Margin_of_Error	4.83E+01	7.20E+01	9.14E+01	6.59E+01	1.08E+02	6.19E+01	1.24E+02	6.10E+02	6.73E+01	1.30E+02	1.03E+02	3.36E+01
F17	50D	CI_Lower	2.95E+03	3.78E+03	3.49E+03	3.32E+03	3.35E+03	2.86E+03	4.60E+03	6.19E+03	3.00E+03	3.06E+03	3.38E+03	2.99E+03
F17	50D	CI_Upper	3.08E+03	3.95E+03	3.93E+03	3.58E+03	3.60E+03	3.11E+03	4.83E+03	6.91E+03	3.26E+03	3.16E+03	3.66E+03	3.29E+03
F17	50D	Margin_of_Error	6.84E+01	8.51E+01	2.17E+02	1.31E+02	1.27E+02	1.28E+02	1.18E+02	3.60E+02	1.30E+02	5.44E+01	1.37E+02	1.48E+02
F17	100D	CI_Lower	4.67E+03	6.06E+03	6.48E+03	2.14E+04	1.75E+04	5.20E+03	2.49E+05	1.77E+06	4.99E+03	4.88E+03	7.21E+03	5.44E+03
F17	100D	CI_Upper	5.16E+03	6.54E+03	7.13E+03	3.61E+04	4.70E+04	5.57E+03	6.43E+05	3.10E+06	5.18E+03	5.33E+03	7.57E+03	5.66E+03
F17	100D	Margin_of_Error	2.43E+02	2.40E+02	3.27E+02	7.33E+03	1.48E+04	1.85E+02	1.97E+05	6.62E+05	9.54E+01	2.26E+02	1.83E+02	1.11E+02
F18	10D	CI_Lower	1.81E+03	5.52E+03	4.47E+03	1.51E+04	3.57E+04	2.69E+04	1.77E+05	4.15E+07	1.22E+04	7.00E+03	5.76E+03	3.55E+03
F18	10D	CI_Upper	1.81E+03	8.26E+03	7.18E+03	2.22E+04	3.85E+04	3.54E+04	2.59E+05	3.00E+08	1.79E+04	1.24E+04	8.80E+03	5.39E+03
F18	10D	Margin_of_Error	3.80E-01	1.37E+03	1.36E+03	3.55E+03	1.41E+03	4.24E+03	4.12E+04	1.29E+08	2.87E+03	2.71E+03	1.52E+03	9.22E+02
F18	30D	CI_Lower	2.49E+03	1.04E+05	3.38E+05	8.16E+05	1.39E+06	2.51E+06	1.04E+07	1.75E+07	1.11E+05	8.43E+04	3.61E+05	3.65E+05
F18	30D	CI_Upper	2.76E+03	1.18E+05	9.61E+05	1.06E+06	2.31E+06	5.00E+06	2.43E+07	3.24E+07	1.52E+05	1.19E+05	4.07E+05	8.41E+05
F18	30D	Margin_of_Error	1.33E+02	7.00E+03	3.11E+05	1.21E+05	4.58E+05	1.24E+06	6.91E+06	7.48E+06	2.06E+04	1.74E+04	2.31E+04	2.38E+05
F18	50D	CI_Lower	5.26E+04	1.76E+06	2.62E+06	4.30E+06	1.03E+07	4.84E+06	4.87E+07	1.03E+08	4.56E+05	4.67E+05	2.36E+06	2.77E+06
F18	50D	CI_Upper	1.10E+05	2.71E+06	3.54E+06	7.60E+06	1.61E+07	2.15E+07	7.02E+07	1.22E+08	6.20E+05	5.32E+05	3.13E+06	3.83E+06
F18	50D	Margin_of_Error	2.86E+04	4.79E+05	4.60E+05	1.65E+06	2.89E+06	8.32E+06	1.08E+07	9.39E+06	8.19E+04	3.28E+04	3.84E+05	5.30E+05
F18	100D	CI_Lower	5.37E+05	1.26E+06	6.54E+07	9.59E+06	1.33E+07	6.52E+06	4.38E+07	1.04E+08	7.91E+05	7.09E+05	2.52E+06	4.96E+06
F18	100D	CI_Upper	7.34E+05	1.70E+06	9.18E+07	1.33E+07	1.95E+07	1.21E+07	4.82E+07	1.40E+08	1.02E+06	9.63E+05	3.39E+06	6.15E+06
F18	100D	Margin_of_Error	9.86E+04	2.18E+05	1.32E+07	1.87E+06	3.07E+06	2.77E+06	2.17E+06	1.81E+07	1.14E+05	1.27E+05	4.35E+05	5.98E+05
F19	10D	CI_Lower	1.90E+03	6.90E+03	1.94E+03	7.30E+03	1.07E+04	6.59E+03	6.76E+04	4.98E+04	8.09E+03	7.66E+03	5.92E+03	2.20E+03
F19	10D	CI_Upper	1.90E+03	1.01E+04	2.03E+03	1.08E+04	1.49E+04	1.25E+04	2.40E+05	1.27E+05	1.17E+04	1.15E+04	1.07E+04	2.82E+03
F19	10D	Margin_of_Error	1.54E-01	1.58E+03	4.26E+01	1.76E+03	2.11E+03	2.98E+03	8.62E+04	3.87E+04	1.80E+03	1.94E+03	2.39E+03	3.13E+02
F19	30D	CI_Lower	1.96E+03	1.67E+06	1.40E+04	4.94E+05	6.69E+06	4.62E+05	1.59E+07	3.21E+07	4.90E+03	3.85E+03	1.01E+06	1.67E+04
F19	30D	CI_Upper	1.97E+03	2.12E+06	3.59E+04	9.49E+05	3.99E+07	9.70E+05	2.28E+07	1.15E+08	5.45E+03	4.74E+03	1.09E+06	2.80E+04
F19	30D	Margin_of_Error	5.04E+00	2.23E+05	1.10E+04	2.27E+05	1.66E+07	2.54E+05	3.42E+06	4.14E+07	2.74E+02	4.45E+02	4.15E+04	5.63E+03
F19	50D	CI_Lower	1.42E+04	6.67E+05	8.75E+04	1.19E+08	1.72E+08	4.95E+06	2.85E+08	1.75E+09	2.10E+04	2.65E+04	4.12E+05	2.61E+04
F19	50D	CI_Upper	2.05E+04	1.39E+06	3.14E+05	2.79E+08	5.09E+08	1.76E+07	4.12E+08	2.63E+09	3.11E+04	3.53E+04	4.29E+05	3.42E+04
F19	50D	Margin_of_Error	3.20E+03	3.64E+05	1.13E+05	8.02E+07	1.68E+08	6.31E+06	6.37E+07	4.40E+08	5.04E+03	4.41E+03	8.73E+03	4.07E+03
F19	100D	CI_Lower	4.95E+03	8.42E+06	1.37E+07	5.20E+09	9.75E+08	1.72E+08	6.62E+09	1.87E+10	6.31E+03	7.68E+03	1.79E+06	2.48E+05
F19	100D	CI_Upper	8.17E+03	1.32E+07	7.86E+07	8.88E+09	1.37E+09	3.19E+08	9.39E+09	2.25E+10	1.04E+04	9.65E+03	1.90E+06	5.60E+05
F19	100D	Margin_of_Error	1.61E+03	2.37E+06	3.24E+07	1.84E+09	1.96E+08	7.35E+07	1.38E+09	1.87E+09	2.04E+03	9.82E+02	5.69E+04	1.56E+05
F20	10D	CI_Lower	2.01E+03	2.16E+03	2.05E+03	2.10E+03	2.10E+03	2.05E+03	2.29E+03	2.08E+03	2.10E+03	2.04E+03	2.15E+03	2.03E+03
F20	10D	CI_Upper	2.01E+03	2.22E+03	2.08E+03	2.14E+03	2.15E+03	2.09E+03	2.35E+03	2.13E+03	2.13E+03	2.05E+03	2.19E+03	2.05E+03
F20	10D	Margin_of_Error	3.17E+00	2.84E+01	1.81E+01	2.05E+01	2.64E+01	1.91E+01	3.25E+01	2.24E+01	1.58E+01	2.87E+00	2.24E+01	1.02E+01
F20	30D	CI_Lower	2.32E+03	2.60E+03	2.51E+03	2.64E+03	2.34E+03	2.39E+03	3.02E+03	2.59E+03	2.40E+03	2.52E+03	2.65E+03	2.40E+03
F20	30D	CI_Upper	2.41E+03	2.82E+03	2.63E+03	2.71E+03	2.46E+03	2.49E+03	3.17E+03	2.70E+03	2.50E+03	2.61E+03	2.71E+03	2.52E+03
F20	30D	Margin_of_Error	4.14E+01	1.13E+02	6.08E+01	3.52E+01	5.85E+01	4.71E+01	7.78E+01	5.51E+01	5.02E+01	4.21E+01	3.01E+01	5.78E+01
F20	50D	CI_Lower	2.95E+03	3.31E+03	3.17E+03	2.97E+03	3.30E+03	2.94E+03	4.09E+03	3.55E+03	2.89E+03	3.13E+03	3.18E+03	2.91E+03
F20	50D	CI_Upper	3.01E+03	3.49E+03	3.29E+03	3.11E+03	3.55E+03	3.02E+03	4.37E+03	3.75E+03	3.03E+03	3.45E+03	3.29E+03	3.02E+03
F20	50D	Margin_of_Error	2.98E+01	8.90E+01	6.00E+01	7.12E+01	1.26E+02	4.22E+01	1.40E+02	9.93E+01	6.64E+01	1.60E+02	5.69E+01	5.27E+01
F20	100D	CI_Lower	5.41E+03	5.91E+03	8.55E+03	6.08E+03	5.86E+03	5.51E+03	7.64E+03	7.42E+03	4.96E+03	5.01E+03	6.18E+03	5.61E+03
F20	100D	CI_Upper	6.13E+03	6.38E+03	9.80E+03	6.32E+03	6.81E+03	6.72E+03	7.82E+03	7.62E+03	5.28E+03	5.31E+03	6.77E+03	6.55E+03
F20	100D	Margin_of_Error	3.62E+02	2.34E+02	6.21E+02	1.21E+02	4.75E+02	6.08E+02	8.82E+01	9.62E+01	1.61E+02	1.50E+02	2.98E+02	4.68E+02
F21	10D	CI_Lower	2.24E+03	2.27E+03	2.32E+03	2.33E+03	2.32E+03	2.31E+03	2.37E+03	2.31E+03	2.20E+03	2.20E+03	2.27E+03	2.28E+03
F21	10D	CI_Upper	2.28E+03	2.31E+03	2.32E+03	2.34E+03	2.33E+03	2.32E+03	2.39E+03	2.35E+03	2.24E+03	2.23E+03	2.32E+03	2.32E+03

Table F1 Confidence intervals at various dimensions. (Part 6/8)

F	D	M	AEHMO	HMO	MGTOA	AOA	IVY	GWO	HEOA	HOA	MHCSA	AOA-HHO	MHABC-PSO	ASG-HMO
F21	10D	Margin_of_Error	2.28E+01	2.46E+01	2.86E+00	5.32E+00	3.04E+00	1.58E+00	9.79E+00	1.91E+01	1.88E+01	1.63E+01	2.36E+01	1.72E+01
F21	30D	CI_Lower	2.41E+03	2.53E+03	2.47E+03	2.48E+03	2.49E+03	2.40E+03	2.65E+03	2.61E+03	2.42E+03	2.39E+03	2.53E+03	2.39E+03
F21	30D	CI_Upper	2.45E+03	2.56E+03	2.50E+03	2.49E+03	2.53E+03	2.41E+03	2.68E+03	2.64E+03	2.44E+03	2.42E+03	2.56E+03	2.41E+03
F21	30D	Margin_of_Error	2.14E+01	1.46E+01	1.28E+01	5.91E+00	2.32E+01	6.04E+00	1.33E+01	1.52E+01	1.05E+01	1.53E+01	1.61E+01	8.73E+00
F21	50D	CI_Lower	2.49E+03	2.72E+03	2.63E+03	2.82E+03	2.73E+03	2.50E+03	3.07E+03	3.01E+03	2.56E+03	2.56E+03	2.72E+03	2.52E+03
F21	50D	CI_Upper	2.51E+03	2.79E+03	2.68E+03	2.84E+03	2.78E+03	2.52E+03	3.14E+03	3.02E+03	2.60E+03	2.59E+03	2.77E+03	2.54E+03
F21	50D	Margin_of_Error	1.02E+01	3.26E+01	2.52E+01	1.04E+01	2.22E+01	1.23E+01	3.41E+01	7.48E+00	2.03E+01	1.87E+01	2.34E+01	1.38E+01
F21	100D	CI_Lower	2.93E+03	3.57E+03	3.58E+03	3.75E+03	3.44E+03	3.03E+03	4.25E+03	4.51E+03	2.94E+03	2.87E+03	3.37E+03	3.10E+03
F21	100D	CI_Upper	3.00E+03	3.68E+03	3.75E+03	3.80E+03	3.50E+03	3.07E+03	4.39E+03	4.62E+03	2.99E+03	2.94E+03	3.43E+03	3.19E+03
F21	100D	Margin_of_Error	3.85E+01	5.21E+01	8.26E+01	2.18E+01	3.16E+01	2.25E+01	7.03E+01	5.65E+01	2.57E+01	3.15E+01	2.70E+01	4.38E+01
F22	10D	CI_Lower	2.30E+03	2.31E+03	2.24E+03	2.34E+03	2.33E+03	2.31E+03	2.32E+03	2.84E+03	2.28E+03	2.30E+03	2.29E+03	2.30E+03
F22	10D	CI_Upper	2.30E+03	2.32E+03	2.27E+03	2.36E+03	2.36E+03	2.31E+03	2.35E+03	2.94E+03	2.30E+03	2.30E+03	2.32E+03	2.30E+03
F22	10D	Margin_of_Error	4.10E-01	5.83E+00	1.45E+01	8.07E+00	1.54E+01	1.94E+00	1.47E+01	5.09E+01	1.17E+01	4.63E-01	1.16E+01	3.25E-01
F22	30D	CI_Lower	2.30E+03	2.43E+03	6.42E+03	6.09E+03	4.94E+03	5.48E+03	7.09E+03	8.11E+03	2.37E+03	2.38E+03	3.66E+03	4.38E+03
F22	30D	CI_Upper	2.30E+03	4.08E+03	8.26E+03	6.66E+03	6.30E+03	5.97E+03	8.51E+03	8.70E+03	3.35E+03	3.44E+03	4.39E+03	5.33E+03
F22	30D	Margin_of_Error	3.46E-01	8.27E+02	9.22E+02	2.85E+02	6.79E+02	2.42E+02	7.13E+02	2.98E+02	4.87E+02	5.33E+02	3.65E+02	4.74E+02
F22	50D	CI_Lower	7.48E+03	1.00E+04	1.45E+04	1.22E+04	1.25E+04	8.76E+03	1.61E+04	1.54E+04	1.26E+04	9.00E+03	1.15E+04	8.39E+03
F22	50D	CI_Upper	8.23E+03	1.10E+04	1.54E+04	1.27E+04	1.44E+04	9.52E+03	1.66E+04	1.58E+04	1.45E+04	9.88E+03	1.18E+04	9.17E+03
F22	50D	Margin_of_Error	3.80E+02	4.63E+02	4.57E+02	2.62E+02	9.16E+02	3.77E+02	2.82E+02	2.20E+02	9.51E+02	4.40E+02	1.67E+02	3.91E+02
F22	100D	CI_Lower	1.89E+04	2.30E+04	3.00E+04	2.70E+04	2.54E+04	1.95E+04	3.46E+04	3.16E+04	1.94E+04	2.21E+04	2.25E+04	2.11E+04
F22	100D	CI_Upper	2.00E+04	2.44E+04	3.24E+04	2.81E+04	2.81E+04	2.09E+04	3.51E+04	3.24E+04	2.07E+04	2.71E+04	2.32E+04	2.61E+04
F22	100D	Margin_of_Error	5.58E+02	6.57E+02	1.19E+03	5.56E+02	1.35E+03	6.94E+02	2.39E+02	3.54E+02	6.19E+02	2.50E+03	3.57E+02	2.51E+03
F23	10D	CI_Lower	2.61E+03	2.64E+03	2.64E+03	2.65E+03	2.63E+03	2.61E+03	2.68E+03	2.74E+03	2.62E+03	2.61E+03	2.63E+03	2.62E+03
F23	10D	CI_Upper	2.62E+03	2.65E+03	2.67E+03	2.66E+03	2.64E+03	2.61E+03	2.70E+03	2.77E+03	2.63E+03	2.62E+03	2.65E+03	2.62E+03
F23	10D	Margin_of_Error	2.57E+00	6.51E+00	1.11E+01	2.77E+00	4.32E+00	2.21E+00	6.06E+00	1.50E+01	2.07E+00	1.92E+00	6.71E+00	3.55E+00
F23	30D	CI_Lower	2.72E+03	3.02E+03	2.91E+03	2.97E+03	2.89E+03	2.78E+03	3.28E+03	3.58E+03	2.79E+03	2.75E+03	2.93E+03	2.78E+03
F23	30D	CI_Upper	2.74E+03	3.09E+03	2.94E+03	3.01E+03	2.93E+03	2.82E+03	3.30E+03	3.72E+03	2.81E+03	2.76E+03	2.97E+03	2.79E+03
F23	30D	Margin_of_Error	7.89E+00	3.72E+01	1.74E+01	1.72E+01	2.33E+01	2.12E+01	1.41E+01	7.00E+01	1.02E+01	7.74E+00	1.97E+01	4.23E+00
F23	50D	CI_Lower	3.10E+03	3.49E+03	3.33E+03	3.55E+03	3.26E+03	2.99E+03	3.96E+03	4.57E+03	2.97E+03	3.03E+03	3.37E+03	3.06E+03
F23	50D	CI_Upper	3.21E+03	3.57E+03	3.39E+03	3.64E+03	3.32E+03	3.02E+03	4.13E+03	4.70E+03	3.03E+03	3.07E+03	3.42E+03	3.10E+03
F23	50D	Margin_of_Error	5.41E+01	4.19E+01	2.97E+01	4.52E+01	2.67E+01	1.36E+01	8.46E+01	6.44E+01	3.00E+01	2.09E+01	2.71E+01	1.88E+01
F23	100D	CI_Lower	3.84E+03	4.83E+03	7.74E+03	4.77E+03	4.41E+03	3.64E+03	5.77E+03	7.80E+03	3.38E+03	3.30E+03	4.13E+03	3.55E+03
F23	100D	CI_Upper	3.95E+03	5.00E+03	8.38E+03	4.98E+03	4.52E+03	3.73E+03	6.14E+03	8.06E+03	3.42E+03	3.35E+03	4.31E+03	3.59E+03
F23	100D	Margin_of_Error	5.37E+01	8.68E+01	3.20E+02	1.05E+02	5.03E+01	4.43E+01	1.86E+02	1.30E+02	1.85E+01	2.87E+01	9.01E+01	1.87E+01
F24	10D	CI_Lower	2.61E+03	2.69E+03	2.63E+03	2.78E+03	2.76E+03	2.75E+03	2.82E+03	2.69E+03	2.67E+03	2.74E+03	2.76E+03	2.75E+03
F24	10D	CI_Upper	2.70E+03	2.78E+03	2.73E+03	2.79E+03	2.77E+03	2.75E+03	2.85E+03	2.79E+03	2.74E+03	2.75E+03	2.77E+03	2.75E+03
F24	10D	Margin_of_Error	4.42E+01	4.15E+01	5.04E+01	6.85E+00	4.04E+00	2.94E+00	1.29E+01	5.05E+01	3.67E+01	2.04E+00	4.94E+00	2.74E+00
F24	30D	CI_Lower	2.91E+03	3.19E+03	3.02E+03	3.27E+03	3.12E+03	2.93E+03	3.34E+03	4.01E+03	2.93E+03	3.04E+03	3.03E+03	2.91E+03
F24	30D	CI_Upper	2.93E+03	3.28E+03	3.09E+03	3.31E+03	3.14E+03	2.99E+03	3.41E+03	4.17E+03	2.96E+03	3.08E+03	3.06E+03	2.93E+03
F24	30D	Margin_of_Error	1.04E+01	4.55E+01	3.56E+01	2.13E+01	9.22E+00	2.77E+01	3.81E+01	8.07E+01	1.62E+01	2.39E+01	1.62E+01	8.51E+00
F24	50D	CI_Lower	3.07E+03	3.76E+03	3.59E+03	4.00E+03	3.52E+03	3.14E+03	4.03E+03	5.46E+03	3.07E+03	3.30E+03	3.39E+03	3.17E+03
F24	50D	CI_Upper	3.09E+03	3.86E+03	3.83E+03	4.06E+03	3.57E+03	3.25E+03	4.21E+03	5.55E+03	3.11E+03	3.40E+03	3.46E+03	3.23E+03
F24	50D	Margin_of_Error	7.68E+00	4.72E+01	1.18E+02	2.70E+01	2.53E+01	5.05E+01	9.13E+01	4.42E+01	1.70E+01	5.34E+01	3.73E+01	3.06E+01
F24	100D	CI_Lower	3.91E+03	7.18E+03	6.08E+03	7.32E+03	5.91E+03	4.32E+03	7.34E+03	1.27E+04	3.98E+03	5.27E+03	5.57E+03	4.55E+03
F24	100D	CI_Upper	3.98E+03	7.85E+03	6.51E+03	7.56E+03	6.01E+03	4.49E+03	7.71E+03	1.28E+04	4.02E+03	5.63E+03	5.72E+03	4.68E+03
F24	100D	Margin_of_Error	3.56E+01	3.31E+02	2.19E+02	1.22E+02	5.10E+01	8.62E+01	1.84E+02	7.05E+01	2.24E+01	1.81E+02	7.33E+01	6.77E+01

Table F1 Confidence intervals at various dimensions. (Part 7/8)

F	D	M	AEHMO	HMO	MGTOA	AOA	IVY	GWO	HEOA	HOA	MHCSA	AOA-HHO	MHABC-PSO	ASG-HMO
F25	10D	CI_Lower	2.92E+03	2.95E+03	2.94E+03	2.95E+03	2.93E+03	2.94E+03	2.96E+03	3.08E+03	2.91E+03	2.91E+03	2.92E+03	2.91E+03
F25	10D	CI_Upper	2.94E+03	2.95E+03	2.94E+03	3.05E+03	2.96E+03	2.95E+03	2.99E+03	3.18E+03	2.93E+03	2.93E+03	2.94E+03	2.93E+03
F25	10D	Margin_of_Error	8.33E+00	7.86E-01	3.43E-01	4.58E+01	1.28E+01	4.06E+00	1.42E+01	4.94E+01	8.78E+00	8.79E+00	1.10E+01	9.10E+00
F25	30D	CI_Lower	2.89E+03	2.91E+03	2.98E+03	3.35E+03	3.13E+03	2.97E+03	3.37E+03	3.94E+03	2.89E+03	2.89E+03	3.01E+03	2.89E+03
F25	30D	CI_Upper	2.89E+03	2.93E+03	2.99E+03	3.49E+03	3.23E+03	3.00E+03	3.47E+03	4.10E+03	2.90E+03	2.91E+03	3.02E+03	2.90E+03
F25	30D	Margin_of_Error	2.53E+00	1.00E+01	3.98E+00	6.91E+01	4.73E+01	1.46E+01	4.87E+01	7.92E+01	4.41E+00	6.34E+00	5.17E+00	3.01E+00
F25	50D	CI_Lower	3.06E+03	3.13E+03	3.52E+03	6.90E+03	5.70E+03	3.76E+03	7.83E+03	1.22E+04	3.12E+03	3.08E+03	4.27E+03	3.13E+03
F25	50D	CI_Upper	3.10E+03	3.19E+03	3.70E+03	7.73E+03	5.97E+03	3.96E+03	8.90E+03	1.33E+04	3.16E+03	3.11E+03	4.46E+03	3.18E+03
F25	50D	Margin_of_Error	1.69E+01	3.20E+01	9.21E+01	4.15E+02	1.34E+02	9.94E+01	5.37E+02	5.22E+02	2.12E+01	1.33E+01	9.61E+01	2.37E+01
F25	100D	CI_Lower	3.26E+03	3.83E+03	7.04E+03	1.55E+04	1.24E+04	6.69E+03	1.73E+04	2.38E+04	4.07E+03	3.29E+03	9.35E+03	4.00E+03
F25	100D	CI_Upper	3.33E+03	3.89E+03	7.94E+03	1.65E+04	1.39E+04	6.95E+03	1.83E+04	2.58E+04	4.21E+03	3.35E+03	9.76E+03	4.18E+03
F25	100D	Margin_of_Error	3.32E+01	3.32E+01	4.53E+02	4.89E+02	7.40E+02	1.29E+02	4.85E+02	9.82E+02	7.22E+01	2.59E+01	2.09E+02	8.76E+01
F26	10D	CI_Lower	3.05E+03	3.31E+03	2.91E+03	3.16E+03	3.01E+03	3.03E+03	3.28E+03	3.75E+03	2.90E+03	2.90E+03	3.08E+03	3.20E+03
F26	10D	CI_Upper	3.13E+03	3.79E+03	3.02E+03	3.50E+03	3.17E+03	3.31E+03	3.54E+03	4.08E+03	2.98E+03	2.98E+03	3.23E+03	3.49E+03
F26	10D	Margin_of_Error	3.82E+01	2.41E+02	5.59E+01	1.70E+02	7.83E+01	1.40E+02	1.27E+02	1.66E+02	4.00E+01	4.04E+01	7.62E+01	1.43E+02
F26	30D	CI_Lower	3.59E+03	7.38E+03	4.47E+03	6.83E+03	5.69E+03	4.63E+03	7.12E+03	9.99E+03	3.28E+03	6.44E+03	7.10E+03	5.43E+03
F26	30D	CI_Upper	4.23E+03	7.88E+03	5.71E+03	7.59E+03	6.19E+03	4.78E+03	7.83E+03	1.08E+04	4.64E+03	7.05E+03	7.66E+03	5.73E+03
F26	30D	Margin_of_Error	3.19E+02	2.49E+02	6.23E+02	3.81E+02	2.51E+02	7.51E+01	3.58E+02	4.10E+02	6.79E+02	3.03E+02	2.79E+02	1.48E+02
F26	50D	CI_Lower	6.55E+03	1.16E+04	7.63E+03	1.27E+04	8.90E+03	8.84E+03	1.43E+04	1.64E+04	8.44E+03	7.92E+03	1.12E+04	7.40E+03
F26	50D	CI_Upper	6.94E+03	1.26E+04	9.92E+03	1.34E+04	9.62E+03	1.11E+04	1.50E+04	1.71E+04	1.10E+04	9.76E+03	1.22E+04	7.91E+03
F26	50D	Margin_of_Error	1.97E+02	4.77E+02	1.14E+03	3.78E+02	3.61E+02	1.15E+03	3.55E+02	3.55E+02	1.29E+03	9.19E+02	5.34E+02	2.54E+02
F26	100D	CI_Lower	1.58E+04	3.02E+04	2.65E+04	3.87E+04	2.62E+04	2.79E+04	4.23E+04	5.15E+04	2.16E+04	2.49E+04	3.14E+04	1.82E+04
F26	100D	CI_Upper	1.69E+04	3.09E+04	2.84E+04	4.02E+04	2.76E+04	2.89E+04	4.51E+04	5.40E+04	2.34E+04	2.60E+04	3.25E+04	1.94E+04
F26	100D	Margin_of_Error	5.09E+02	3.08E+02	9.66E+02	7.22E+02	6.99E+02	5.18E+02	1.38E+03	1.23E+03	8.93E+02	5.54E+02	5.19E+02	5.88E+02
F27	10D	CI_Lower	3.10E+03	3.14E+03	3.12E+03	3.12E+03	3.10E+03	3.10E+03	3.16E+03	3.23E+03	3.09E+03	3.09E+03	3.09E+03	3.11E+03
F27	10D	CI_Upper	3.10E+03	3.17E+03	3.16E+03	3.14E+03	3.11E+03	3.11E+03	3.19E+03	3.26E+03	3.09E+03	3.09E+03	3.10E+03	3.11E+03
F27	10D	Margin_of_Error	1.74E+00	1.39E+01	1.77E+01	1.01E+01	4.92E+00	7.23E+00	1.38E+01	1.57E+01	1.05E+00	7.35E-01	1.61E+00	1.35E+00
F27	30D	CI_Lower	3.20E+03	3.46E+03	3.27E+03	3.53E+03	3.31E+03	3.28E+03	3.57E+03	4.51E+03	3.24E+03	3.24E+03	3.44E+03	3.27E+03
F27	30D	CI_Upper	3.20E+03	3.56E+03	3.31E+03	3.57E+03	3.37E+03	3.31E+03	3.62E+03	4.81E+03	3.25E+03	3.26E+03	3.54E+03	3.28E+03
F27	30D	Margin_of_Error	8.34E-05	5.02E+01	1.80E+01	2.18E+01	2.93E+01	1.67E+01	2.57E+01	1.51E+02	5.55E+00	9.65E+00	5.02E+01	6.78E+00
F27	50D	CI_Lower	3.20E+03	4.85E+03	3.67E+03	4.48E+03	4.20E+03	3.67E+03	5.34E+03	7.04E+03	3.48E+03	3.58E+03	4.22E+03	3.69E+03
F27	50D	CI_Upper	3.20E+03	5.52E+03	3.82E+03	4.68E+03	4.34E+03	3.72E+03	5.76E+03	7.44E+03	3.58E+03	3.68E+03	4.44E+03	3.75E+03
F27	50D	Margin_of_Error	8.95E-05	3.34E+02	7.70E+01	9.84E+01	7.08E+01	2.36E+01	2.11E+02	1.99E+02	4.91E+01	5.18E+01	1.06E+02	3.26E+01
F27	100D	CI_Lower	3.76E+03	5.55E+03	4.76E+03	6.64E+03	5.32E+03	4.15E+03	8.50E+03	1.39E+04	3.20E+03	3.78E+03	6.93E+03	4.07E+03
F27	100D	CI_Upper	3.81E+03	5.82E+03	4.92E+03	7.17E+03	5.50E+03	4.31E+03	9.36E+03	1.49E+04	3.20E+03	3.85E+03	7.22E+03	4.17E+03
F27	100D	Margin_of_Error	2.75E+01	1.35E+02	7.92E+01	2.65E+02	9.28E+01	7.88E+01	4.33E+02	5.01E+02	9.80E-05	3.32E+01	1.45E+02	4.99E+01
F28	10D	CI_Lower	3.15E+03	3.31E+03	3.35E+03	3.36E+03	3.35E+03	3.33E+03	3.55E+03	3.74E+03	3.41E+03	3.31E+03	3.33E+03	3.33E+03
F28	10D	CI_Upper	3.26E+03	3.42E+03	3.40E+03	3.48E+03	3.41E+03	3.40E+03	3.61E+03	3.79E+03	3.41E+03	3.39E+03	3.42E+03	3.40E+03
F28	10D	Margin_of_Error	5.87E+01	5.69E+01	2.24E+01	5.84E+01	3.20E+01	3.44E+01	2.72E+01	2.81E+01	3.33E-05	4.42E+01	4.83E+01	3.46E+01
F28	30D	CI_Lower	3.16E+03	3.28E+03	3.30E+03	4.10E+03	3.67E+03	3.40E+03	4.10E+03	6.04E+03	3.18E+03	3.22E+03	3.53E+03	3.29E+03
F28	30D	CI_Upper	3.19E+03	3.31E+03	3.35E+03	4.53E+03	3.81E+03	3.46E+03	4.51E+03	6.41E+03	3.19E+03	3.24E+03	3.62E+03	3.30E+03
F28	30D	Margin_of_Error	1.37E+01	1.39E+01	2.23E+01	2.12E+02	6.70E+01	3.12E+01	2.05E+02	1.86E+02	8.91E+00	1.07E+01	4.55E+01	6.33E+00
F28	50D	CI_Lower	3.49E+03	3.39E+03	4.01E+03	6.86E+03	5.58E+03	4.72E+03	8.45E+03	1.14E+04	3.31E+03	3.31E+03	5.33E+03	3.63E+03
F28	50D	CI_Upper	3.53E+03	3.41E+03	4.33E+03	7.56E+03	6.23E+03	4.80E+03	8.88E+03	1.22E+04	3.32E+03	3.32E+03	5.73E+03	3.73E+03
F28	50D	Margin_of_Error	2.14E+01	1.41E+01	1.62E+02	3.51E+02	3.25E+02	4.02E+01	2.12E+02	4.14E+02	3.66E+00	4.83E+00	1.98E+02	5.22E+01
F28	100D	CI_Lower	4.37E+03	3.96E+03	1.17E+04	2.29E+04	1.68E+04	8.60E+03	2.50E+04	3.13E+04	3.45E+03	3.45E+03	1.06E+04	5.25E+03

Table F1 Confidence intervals at various dimensions (part 8).

F	D	M	AEHMO	HMO	MGTOA	AOA	IVY	GWO	HEOA	HOA	MHCSA	AOA-HHO	MHABC-PSO	ASG-HMO
F29	10D	CI_Lower	3.16E+03	3.34E+03	3.21E+03	3.24E+03	3.20E+03	3.20E+03	3.37E+03	3.33E+03	3.18E+03	3.19E+03	3.28E+03	3.19E+03
F29	10D	CI_Upper	3.17E+03	3.41E+03	3.23E+03	3.26E+03	3.22E+03	3.24E+03	3.44E+03	3.37E+03	3.21E+03	3.23E+03	3.31E+03	3.21E+03
F29	10D	Margin_of_Error	4.81E+00	3.65E+01	1.02E+01	1.05E+01	1.05E+01	2.34E+01	3.88E+01	2.31E+01	1.21E+01	2.31E+01	1.65E+01	1.25E+01
F29	30D	CI_Lower	3.78E+03	4.71E+03	3.74E+03	4.23E+03	4.11E+03	3.83E+03	5.57E+03	6.78E+03	3.84E+03	3.79E+03	4.80E+03	3.95E+03
F29	30D	CI_Upper	3.82E+03	4.82E+03	3.96E+03	4.34E+03	4.26E+03	3.98E+03	6.27E+03	7.42E+03	3.94E+03	3.89E+03	5.07E+03	4.09E+03
F29	30D	Margin_of_Error	1.88E+01	5.62E+01	1.08E+02	5.52E+01	7.68E+01	7.77E+01	3.50E+02	3.19E+02	4.97E+01	5.12E+01	1.39E+02	6.71E+01
F29	50D	CI_Lower	4.35E+03	6.12E+03	4.37E+03	7.55E+03	5.70E+03	4.55E+03	1.13E+04	4.85E+04	4.42E+03	4.84E+03	7.22E+03	4.87E+03
F29	50D	CI_Upper	4.67E+03	6.49E+03	5.00E+03	8.30E+03	5.98E+03	4.83E+03	1.67E+04	6.12E+04	4.79E+03	5.06E+03	7.60E+03	5.09E+03
F29	50D	Margin_of_Error	1.59E+02	1.85E+02	3.15E+02	3.75E+02	1.35E+02	1.39E+02	2.69E+03	6.33E+03	1.87E+02	1.11E+02	1.91E+02	1.06E+02
F29	100D	CI_Lower	7.51E+03	1.31E+04	8.34E+03	2.16E+04	1.46E+04	8.32E+03	3.48E+04	2.20E+05	8.49E+03	7.45E+03	1.57E+04	8.27E+03
F29	100D	CI_Upper	7.93E+03	1.43E+04	9.90E+03	4.88E+04	1.68E+04	8.94E+03	5.81E+04	2.83E+05	8.96E+03	7.70E+03	1.68E+04	8.48E+03
F29	100D	Margin_of_Error	2.11E+02	5.95E+02	7.78E+02	1.36E+04	1.11E+03	3.11E+02	1.16E+04	3.15E+04	2.37E+02	1.30E+02	5.36E+02	1.05E+02
F30	10D	CI_Lower	3.42E+03	3.23E+05	4.66E+03	8.98E+05	2.42E+05	4.16E+05	1.10E+06	1.17E+06	1.58E+04	1.64E+04	1.31E+05	1.07E+04
F30	10D	CI_Upper	3.43E+03	1.65E+06	6.44E+03	4.95E+06	6.93E+05	1.14E+06	2.01E+06	3.42E+06	2.31E+04	2.49E+04	3.54E+05	1.51E+04

F30	10D	Margin_of_Error	8.63E+00	6.64E+05	8.93E+02	2.03E+06	2.25E+05	3.64E+05	4.53E+05	1.12E+06	3.68E+03	4.23E+03	1.11E+05	2.17E+03
F30	30D	CI_Lower	6.84E+03	4.20E+06	5.77E+04	8.19E+06	2.50E+07	4.14E+06	1.56E+08	6.91E+08	1.33E+04	1.33E+04	4.76E+06	5.33E+04
F30	30D	CI_Upper	7.89E+03	6.13E+06	6.28E+05	1.54E+07	4.11E+07	7.03E+06	3.17E+08	1.09E+09	2.22E+04	1.50E+04	6.22E+06	1.14E+05
F30	30D	Margin_of_Error	5.23E+02	9.68E+05	2.85E+05	3.59E+06	8.03E+06	1.44E+06	8.09E+07	1.99E+08	4.41E+03	8.41E+02	7.31E+05	3.02E+04
F30	50D	CI_Lower	1.16E+06	9.13E+07	7.84E+05	2.98E+08	3.26E+08	1.25E+08	6.92E+08	4.80E+09	9.25E+05	1.04E+06	1.73E+08	5.66E+06
F30	50D	CI_Upper	1.35E+06	1.10E+08	2.78E+06	5.04E+08	4.87E+08	1.44E+08	9.08E+08	6.18E+09	1.04E+06	1.13E+06	1.90E+08	8.63E+06
F30	50D	Margin_of_Error	9.19E+04	9.25E+06	9.98E+05	1.03E+08	8.01E+07	9.21E+06	1.08E+08	6.89E+08	5.96E+04	4.54E+04	8.43E+06	1.49E+06
F30	100D	CI_Lower	2.04E+06	1.29E+08	3.78E+08	1.37E+10	4.07E+09	9.53E+08	1.58E+10	3.28E+10	2.42E+05	1.88E+05	4.24E+09	4.08E+06
F30	100D	CI_Upper	3.12E+06	2.02E+08	1.91E+09	1.65E+10	6.64E+09	1.53E+09	1.83E+10	3.59E+10	3.35E+05	2.67E+05	5.84E+09	6.22E+06
F30	100D	Margin_of_Error	5.41E+05	3.66E+07	7.68E+08	1.38E+09	1.29E+09	2.87E+08	1.27E+09	1.56E+09	4.61E+04	3.95E+04	8.00E+08	1.07E+06

Table F2 Effect size at various dimensions.

	10D	30D	50D	100D
AEHMO vs AOA	0.873	0.873	0.873	0.873
AEHMO vs HEOA	0.865	0.873	0.873	0.873
AEHMO vs HMO	0.873	0.873	0.873	0.873
AEHMO vs HOA	0.865	0.873	0.873	0.873
AEHMO vs IVY	0.873	0.873	0.873	0.873
AEHMO vs MGTOA	0.873	0.873	0.873	0.873
AEHMO vs MHABC-PSO	0.873	0.873	0.873	0.873
AEHMO vs GWO	0.873	0.869	0.869	0.869
AEHMO vs MHCSA	0.873	0.869	0.869	0.865
AEHMO vs AOA-HHO	0.873	0.865	0.865	0.861
AEHMO vs ASG-HMO	0.865	0.865	0.861	0.853

Appendix G: WSN Ablation Study

To quantify the individual contribution of each enhancement strategy, an ablation study was conducted in which each strategy was evaluated in isolation, with all others disabled. The results of the ablation study for both 100-node and 150-node scenarios are presented in Table G1, in terms of their impact on FND, HND, LND, and average energy consumption. The baseline HMO is used as the reference, and the following variants are evaluated: AEHMO-w (adaptive parameters only), AEHMO-M (MDMS only), AEHMO-D (DDS only), AEHMO-C (CRL only), and AEHMO (all four strategies combined).

Table G1 Ablation study results for 100-node and 150-node scenarios.

Variant	100 Nodes				150 Nodes			
	FND	HND	LND	Energy (J)	FND	HND	LND	Energy (J)
**HMO**	481	1498	2571	0.412	833	1631	2841	0.424
**AEHMO-w**	594	1561	2698	0.401	943	1709	2928	0.414
**AEHMO-M**	615	1587	2754	0.394	981	1746	2981	0.407
**AEHMO-D**	641	1606	2809	0.388	1016	1778	3016	0.401
**AEHMO-C**	678	1639	2861	0.385	1071	1805	3063	0.398
**AEHMO (Full)**	**752**	**1694**	**3010**	**0.381**	**1150**	**1853**	**3136**	**0.395**

The results indicate that each enhancement strategy contributes positively to overall performance of AEHMO, in which the full integration can achieve significantly better results. The baseline HMO shows FND of 481/833 rounds and 0.412/0.424J energy consumption for 100/150 nodes, respectively. Individual strategies added show incremental improvements. AEHMO-w shows 23.5%/13.2% FND improvement and 2.7%/2.4% energy reduction. AEHMO-M shows 27.9%/17.8% FND improvement with 4.4%/4.0% energy reduction. AEHMO-D (DDS only) shows 33.3%/22.0% FND improvement and 5.8%/5.4% energy reduction. However, the full algorithm AEHMO is significantly superior to the single strategies with 752/1150 rounds FND (56.3%/38.1% improvement over HMO) and 0.381/0.395 J energy consumption (7.5%/6.8% reduction). Critically, full AEHMO shows 10.9%/7.4% FND improvement and 1.0%/0.8% further energy reduction compared to the best single strategy variant (AEHMO-C) demonstrating clear synergistic effects. The hierarchical interaction among strategies — where adaptive parameters provide the control framework, DDS and CRL maintain exploration and diversity, and MDMS enhances exploitation

— produces a cooperative mechanism whose combined effect exceeds the sum of individual contributions. The results of this ablation study strongly support the necessity of utilizing all four enhancement strategies for optimal energy efficient cluster head selection in WSNs.

Appendix H: WSN Sensitivity Analysis

Effect of Chaotic iteration depth (z) in CRL for WSN

The level of structured randomness introduced to the CH selection is controlled by a chaotic iteration depth z and has a direct effect on the ability of the algorithm to escape suboptimal cluster configurations. Table H1 presents the WSN performance metrics obtained for different values of z in the 150-node scenario.

Table H1 Sensitivity analysis of chaotic iteration depth (z) for WSN (150 nodes).

***z* value**	**FND**	**HND**	**LND**
10	987	1721	2989
15	1089	1798	3074
20	**1150**	**1853**	**3136**
25	1126	1831	3108
30	1042	1763	3021

The results show that a low number of chaotic iterations (z = 10) leads to inadequate exploration of the CH solution space, which leads to premature convergence to suboptimal cluster configurations with FND of only 987 rounds. As z is increased to 15, the performance is greatly improved with FND reaching 1089 rounds and LND to 3074 rounds, demonstrating that moderate chaotic depth enhances diversity in CH candidate evaluation. Optimal performance is achieved at z = 20 with FND of 1150 rounds, HND of 1853 rounds and LND of 3136 rounds. This confirms that z = 20 introduces sufficient structured randomness to identify optimal CH arrangements while preserving convergence stability. Values close to the optimum (z = 15 and z = 25) give competitive but slightly worse results and excessive chaotic iterations (z = 30) decrease performance due to the overhead for computation and too many perturbations to disrupt the refinement of promising cluster configurations. The consistency between CEC benchmarks and the WSN application suggests that z = 20 is a good selection in various problem domains.

Effect of MDMS mutation percentage for WSN

The mutation percentage in the MDMS strategy determines the percentage of elite CH solutions to be refined at each iteration, which has a direct influence on the trade-off between the exploitation of the configuration of clusters and the stability of the network. Table H2 shows the WSN performance for different mutation percentages ranging from 10% to 50%.

Table H2 Sensitivity analysis of MDMS mutation percentage for WSN (150 nodes).

**Mutation %**	**FND**	**HND**	**LND**
10%	941	1684	2897
20%	1098	1812	3089
25%	**1150**	**1853**	**3136**
30%	1134	1839	3118
35%	1067	1771	3042
40%	963	1702	2934
45%	1034	1765	3071
50%	997	1806	2971

According to the results in Table H2, mutating only 10% of the elite population results in insufficient exploitation of the high-quality CH candidates, thus resulting in poor FND of 941 rounds and limited LND of 2897 rounds. Increasing mutation percentage to 20% gives significant improvement with FND of 1098 rounds and LND of 3089 rounds indicating that moderate elite refinement improves convergence toward longer-lasting cluster configurations. The best performance is obtained at 25% mutation and FND of 1150 rounds, HND of 1853 rounds and LND of 3136 rounds. Notably, this differs slightly from the CEC optimal value of 30% and thus, it seems that the WSN applications benefit from slightly more conservative elite refinement to ensure network stability and avoid too much disruption of established cluster structures. Values of 30% are still competitive (FND = 1134 rounds, LND = 3118 rounds), but higher mutation percentages (35%-50%) result in a progressively decreasing performance due to the destabilization of the cluster formations and the interruption of the continuity of the effective CH arrangements by excessive perturbations. The gradual performance degradation outside the 20–30% range confirms that AEHMO is not overly sensitive to the mutation percentage, demonstrating robustness across a reasonable parameter interval.

Effect of scaling factor (c) of MDMS for WSN

The scaling factor c controls the strength of the Gaussian and Cauchy mutations of elite CH solutions which has a direct effect on the step size of configuration refinement of clusters and the lifetime of networks. WSN performance for c values ranging from 0.1 to 0.9 is summarized in Table H3.

Table H3 Sensitivity analysis of scaling factor (c) for WSN (150 nodes).

c value	FND	HND	LND
0.1	892	1642	2841
0.3	1018	1739	2976
0.5	1087	1794	3062
0.7	**1150**	**1853**	**3136**
0.8	1134	1839	3118
0.9	1096	1807	3074

Small values of c (0.1–0.3) produce overly conservative mutations, limiting exploration of alternative CH configurations and consequently poor FND (892-1018 rounds), and network lifetimes (LND = 2841-2976 rounds). The nodes suffer from an unequal distribution of loads and premature failures as the algorithm gets stuck in locally optimal, but globally suboptimal arrangements of clusters. Increasing c to 0.5 progressively improves performance where in this case FND achieves 1087 rounds, and LND grows to 3062 rounds, because bigger mutation steps result in better exploration of the CH solution space. The maximum performance is obtained for c = 0.7, which gives FND = 1150 rounds, HND = 1853 rounds, and LND = 3136 rounds. This differs from the CEC benchmark optimal value of c = 0.8 since it reflects the unique characteristics of WSN cluster head selection where slightly smaller mutation steps provide better balance between the discovery of new CH configurations and stability in the established cluster formations. The physical limitations of WSN (such as the discrete nature of node positions, the limitations of the communication range and spatial topology) prefer more moderate perturbations, which account for the structural characteristics of the network. Values of c = 0.8 are still very competitive (FND = 1134 rounds, LND = 3118 rounds) and values of c=0.9 have only slight degradation (FND = 1096 rounds, LND = 3074 rounds) indicating robust performance for a large range of parameters. The smooth performance trend across the range 0.5–0.9 attests to the stability of AEHMO, confirming the principled and generalizable nature of the chosen parameter value, rather than being problem-specific.

Sensitivity analysis of fitness function weight combinations

The coefficients of weight (α, β, γ) in the fitness function control the relative importance of residual energy suitability (FP_1), CH-to-BS proximity (FP_2) and intra-cluster compactness (FP_3) respectively, subject to the following normalization constraint: $\alpha + \beta + \gamma = 1$. FP_4 has a fixed unit weight as non-negotiable heterogeneity constraint, and is therefore not included in this sensitivity analysis.

The hierarchical weight ordering $\alpha > \gamma > \beta$ is derived from first principles based on the physical energy consumption characteristics of cluster-based WSNs:

- α assigned highest priority ($\alpha > \beta, \alpha > \gamma$): CHs are in charge of the most energy-consuming activities in the network, such as continuous reception of data from all the cluster members, local aggregation and long-distance forwarding to the BS. These operations are performed on each round and are not interrupted. A CH with insufficient residual energy dies prematurely, causing network partitions, energy holes, and cascading node failures that cannot be compensated for by spatial optimization alone. Residual energy is therefore the most important factor in determining the sustainability of the network and needs to have the highest weight.
- γ ranked second (γ>β): Intra-cluster communication is the most frequent communication event in cluster-based WSNs where every member node transmits to its CH every single round. Since transmission energy is proportional to the square of the distance in the free space radio model, even modest reductions in the average node-to-CH distance have a large cumulative energy savings effect over thousands of rounds. The high frequency of intra-cluster communication makes intra-cluster compactness the second most influential factor in determining overall network lifetime, thereby justifying $\gamma > \beta$.
- The weight β is assigned the lowest but non-negligible value ($\beta > 0$) because CH-to-BS transmission occurs only once per round per CH, which is significantly less frequent than the multiple member-to-CH transmissions within each cluster. Although CH-to-BS communication is individually more energy-intensive due to longer transmission distances, its cumulative impact is reduced by its lower frequency and the multi-hop routing strategy. Consequently, its overall contribution to network energy consumption remains smaller than that of intra-cluster communication. However, β must remain strictly positive to prevent CHs from being selected at prohibitively large distances from the BS.

These physically grounded arguments establish the strict ordering $\alpha > \gamma > \beta > 0$ with α + β+ γ = 1. Thus, five scenarios are systematically designed to probe for both the compliant and violating configurations of this hierarchy as shown in Table H4.

- Scenario 1 (α=0.50, β=0.20, γ=0.30): Satisfies $\alpha > \gamma > \beta$ but over-concentrates on residual energy. FND=800, HND=1450, LND=2893. Despite respecting the ordering, excessive α suppresses FP_2 and FP_3, causing spatially suboptimal CH selection and loose cluster compactness. This confirms that the hierarchy governs relative ordering but must not be exaggerated in magnitude.
- Scenario 2 (α=0.40, β=0.25, γ=0.35): The only configuration that strictly and proportionately satisfies $\alpha > \gamma > \beta$ with a balanced distribution. FND=840, HND=1509, LND=2997 is the best performance across all metrics. Each criterion contributes meaningfully without any single factor dominating, confirming this as the principled optimal configuration consistent with the baseline weights.
- Scenario 3 (α=0.35, β=0.25, γ=0.40): Mildly violates the hierarchy by setting $\gamma > \alpha$. In this case, FND=830, HND=1483, LND=2924. Over-prioritizing intra-cluster compactness at the expense of residual energy sustainability produces compact but energy-vulnerable CH configurations, demonstrating that even a mild inversion of the α and γ ordering measurably degrades all three network lifetime metrics compared to Scenario 2.
- Scenario 4 (α=0.35, β=0.30, γ=0.35): A borderline case setting $\alpha = \gamma$ while elevating β. In this case, FND=810, HND=1462, LND=2853. Equalizing α and γ while raising β simultaneously reduces energy protection and over-prioritizes CH-to-BS proximity, producing the second-worst

performance and confirming that both the $\alpha > \gamma$ and $\gamma > \beta$ orderings must be strictly maintained.

- Scenario 5 (α=0.30, β=0.25, γ=0.45): Clearly violates the hierarchy with γ as the dominant weight. In this case, FND=800, HND=1450, LND=2820 is the worst performance overall. Forcing AEHMO to prioritize cluster compactness above all else causes selection of spatially tight but energy-deficient CH configurations, accelerating CH depletion and network degradation in later rounds.

Table H4 Different scenario combinations of weights of the fitness function.

Scenario	α	β	γ	FND	HND	LND
1	0.50	0.20	0.30	800	1450	2893
2	0.40	0.25	0.35	840	1509	2997
3	0.35	0.25	0.40	830	1483	2924
4	0.35	0.30	0.35	810	1462	2853
5	0.30	0.25	0.45	800	1450	2820

The results in Table H4 provide strong empirical validation of the established hierarchy using a systematic progression from compliant to violating configurations. The performance ordering can be clearly interpreted in terms of physical energy dynamics: Scenario 2 > Scenario 1 > Scenario 3 > Scenario 4 > Scenario 5, reflecting a progressive decrease in compliance with the hierarchical constraint. Scenario 2, the only configuration that strictly and proportionally satisfies the condition $\alpha > \gamma > \beta$, achieves the best FND, HND and LND at the same time. Scenario 1, although hierarchy compliant, indicates that excessive emphasis on a single criterion can degrade performance, even within the valid ordering region. Scenarios 3 and 5, which progressively reverse the $\gamma-\alpha$ relationship, exhibit monotonically decreasing network lifetime, confirming that γ must remain strictly subordinate to α. Scenario 4, the borderline case, yields the second-lowest performance despite involving only minor weight equalization, which shows that even small deviations from the strict ordering have measurable negative consequences. This systematic performance degradation with increasing hierarchy violation provides strong empirical support for the physically grounded ordering $\alpha > \gamma > \beta$ and for the baseline weights (α=0.40, β=0.25, γ=0.35) being a principled optimal configuration rather than an arbitrary assignment.

Appendix I: BS at Corner Results

To further evaluate the robustness of AEHMO under an asymmetric network topology, the BS was placed at the corner of the sensing field at coordinates (100, 100) in a 100-node deployment. This configuration results presented in Table I1 in non-uniform transmission distance in which CHs that are further away from the BS have a higher communication energy cost, making it harder to maintain an equilibrium of energy dissipation throughout the network. Under this more difficult environment, AEHMO achieves a FND of 785 rounds, an HND of 1420 rounds and an LND of 2815 rounds which is the highest achieved by all compared algorithms as shown in Table I1. The sustained separation between FND, HND, and LND indicates a stable and gradual energy depletion process rather than sudden node failures, i.e., balanced cluster formation and controlled CH rotation during lifetime of the network. When compared with the closest competitor, HMO, AEHMO has 43 rounds more (785 vs. 742), 72 rounds more (1420 vs. 1348) and 126 rounds more (2815 vs. 2689), which is about 5.8%, 5.3% and 4.7% improvement, respectively. The performance benefit is more significant when compared to classical algorithms such as GWO and AOA where AEHMO is better by 431 and 666 rounds, respectively. These results show that AEHMO is effective to mitigate the uneven energy burden caused by the corner BS placement by keeping the adaptive CH selection and avoiding the premature energy concentration in the remote network. The stable ranking

superiority among FND, HND and LND confirms the stability/scalability of the proposed approach under non-ideal and energy-demanding deployment situations.

Table I1 Results when the BS is at the corner using 100 nodes.

Algorithm	FND	HND	LND
**AEHMO**	**785**	**1420**	**2815**
HMO	742	1348	2689
ASG-HMO	728	1322	2641
MHCSA	715	1306	2598
MGTOA	701	1284	2556
MHABC-PSO	688	1262	2494
AOA-HHO	662	1227	2433
GWO	648	1195	2384
HOA	632	1171	2315
HEOA	611	1124	2247
IVY	604	1103	2196
AOA	590	1078	2149

Appendix J: Communication Overhead

The comparison of the communication overhead on different network scales is shown in Table J1, and it shows that AEHMO scales efficiently in handling control message exchange needed for CH selection and network maintenance. For small-scale deployments with 50 nodes, the overhead is minimal as the network size is still below the threshold where simple LEACH-like protocols can efficiently run without any extra coordination complexity. However, with scaling up to 100 nodes differences in overhead emerge with AEHMO showing the lowest overhead of 2.5% compared with MHCSA (2.8%), HMO (3.5%), ASG-HMO (3.2%), GWO (3.8%), AOA (4.2%), HEOA (4.5%) and IVY (4.8%). This 47.9% reduction in overhead from the worst performer IVY (4.8% vs. 2.5%) is owing to AEHMO's efficient population-based optimization that requires fewer iterations of fitness evaluation due to faster convergence enabled by the adaptive parameters w_1 and w_2 that adaptively vary the exploration intensity and avoid unnecessary prolonged search phases. The overhead advantage is more notable at 150 nodes, where AEHMO has 4.0% overhead (with MHCSA at 4.5%, ASG-HMO at 4.8%, HMO at 5.2%, GWO at 5.8%, AOA at 6.5%, HEOA at 7.2% and IVY at 7.8%), which is almost 49% less than IVY and 38.5% less than HEOA. At 500 nodes (medium-scale industrial deployments) AEHMO has 8.5% overhead, always maintaining its efficiency lead over MHCSA (9.2%), ASG-HMO (9.8%), HMO (10.8%), GWO (11.5%), AOA (12.8%), HEOA (14.5%) and IVY (15.2%), with an overhead 44.1% lower than IVY, and 41.4% lower than HEOA.

The most important case occurs at 1000 nodes, which is comparable to the heterogeneous deployment with 1200 nodes discussed in previous subsections, in which communication overhead is a significant factor affecting the network performance and energy efficiency. AEHMO shows outstanding scalability with 12.0% overhead, which is significantly less than all the competitors: MHCSA (13.2%), ASG-HMO (14.0%), HMO (15.5%), GWO (16.8%), AOA (18.5%), HEOA (21.2%) and IVY (22.5%). This corresponds to a 46.7% reduction when compared to IVY and 43.4% reduction when compared to HEOA, which translates into substantial savings in energy consumption when extrapolated to thousands of sensor nodes running for a large period of time. The low overhead can be attributed to several design factors: (1) the memory-based recall mechanism of AEHMO has reduced the redundant fitness evaluations by using the historical solution quality information, without requiring the fitness of similar CH configurations to be evaluated repeatedly; (2) the DDS strategy enables the quick identification of promising search regions at the start of the optimization process, which reduces the total number of iterations required for the

optimization process to converge; (3) MDMS focuses mutation operations on the top 20-30% elite solutions instead of the whole population, which reduces the computational and communication costs associated with widespread population updates;

Table J1 communication overhead for each algorithm versus number of nodes.

Number of Nodes	AOA (%)	GWO (%)	HMO (%)	MHCSA (%)	ASG-HMO (%)	HEOA (%)	IVY (%)	AEHMO (%)
50	0	0	0	0	0	0	0	0
100	4.2	3.8	3.5	2.8	3.2	4.5	4.8	2.5
150	6.5	5.8	5.2	4.5	4.8	7.2	7.8	4.0
500	12.8	11.5	10.8	9.2	9.8	14.5	15.2	8.5
1000	18.5	16.8	15.5	13.2	14.0	21.2	22.5	12.0

Appendix

Engineering problems formulation

Mathematical formulation of Piston Lever Design (PLD) problem

Consider

$$x = [x_1, x_2, x_3, x_4] = [H, B, D, V]$$

Objective function

$$f(X) = \left(\frac{1}{4}\right)\pi\, x_3^2 (L_2 - L_1)$$

Subject to

$$g_{1(X)} = QL\, cos(\theta) - RF \leq 0,$$

$$g_{2(X)} = Q(L - x_4) - M \leq 0,$$

$$g_{3(X)} = 1.2(L_2 - L_1) - L_1 \leq 0,$$

$$g_{4(X)} = \left(\frac{x_3}{2}\right) - x_2 \leq 0,$$

Where

$$F = \frac{\pi P x^3 2}{4}, L_1 = \sqrt{(x_4 - x_2)^2 + {x_1}^2},$$

$$L_2 = \sqrt{(x_4 \sin\theta + x_1)^2 + (x_2 - x_4 \cos\theta)^2}$$

$$R = |-x_4(x_4 \sin\theta + x_1) + x_1(x_2 - x_4\cos\theta)|/L_1,\ \theta = 45°,\ Q = 10{,}000 \text{ lbs}$$

$$M = 1.8 \times 10^6 lbs, P = 1500 psi, L = 240 in$$

Boundaries

$$0.05 \leq x_1, x_2, x_3 \leq 500$$

$$0.05 \leq x_4 \leq 120$$

Mathematical formulation of Tension and compression spring design (TCSD) problem

Consider

$$x = [x_1 x_2 x_3] = [dDN]$$

Objective function

$$f(x) = (x_3 + 2) \times x_2 \times x_1^2$$

Subject to

$$g_1(x) = 1 - \frac{x_3 \times x_2^3}{71785 \times x_1^4} \leq 0$$

$$g_2(x) = \frac{4 \times x_2^2 - x_1 \times x_2}{12566 \times x_1^4} + \frac{1}{5108 \times x_1^2} - 1 \leq 0$$

$$g_3(x) = 1 - \frac{140.45 \times x_1}{x_2^2 \times x_3} \leq 0$$

$$g_4(x) = \frac{x_1 + x_2}{1.5} - 1 \leq 0$$

Boundaries

$$0.05 \leq x_1 \leq 2.0$$
$$0.25 \leq x_2 \leq 1.3$$
$$2.0 \leq x_3 \leq 15.0$$

Mathematical formulation of Tubular column design (TCD) problem

Consider

$$\mathrm{X} = [x_1 x_2]$$

Objective function

$$f(x) = 9.8\, x_1\, x_2 + 2\, x_1,$$

Subject to

$$g_1(x) = P \,/\, (\pi\, x_1\, x_2\, \sigma\_y) - 1 \leq 0,$$

$$g_2(x) = (8\, P\, L^2) \,/\, (\pi^3\, E\, x_1\, x_2\, (x_1{}^2 + x_2{}^2)) - 1 \leq 0,$$

$$g_3(x) = 2.0 \,/\, x_1 - 1 \leq 0,$$

$$g_4(x) = x_1 \,/\, 14 - 1 \leq 0,$$

$$g_5(x) = 0.2 \,/\, x_2 - 1 \leq 0,$$

$$g_6(x) = x_2 \,/\, 8 - 1 \leq 0,$$

Boundaries

$$2 \leq x_1 \leq 14, 0.2 \leq x_2 \leq 0.8.$$

Mathematical formulation of Tension and compression spring design (TCSD) problem

Minimize

$$f(b) = 0.0624(b_1 + b_2 + b_3 + b_4 + b_5), b_i > 0$$

subject to

$$g_1(b) = \frac{61}{b_1^3} + \frac{37}{b_2^3} + \frac{19}{b_3^3} + \frac{7}{b_4^3} + \frac{1}{b_5^3} \leq 1.$$

Variable range:

$$0.01 \leq b_i \leq 100, i = 1,2,3,4,5$$

Mathematical formulation of Corrugated bulkhead design (CBD) problem

$$\text{Min} f(x) = \frac{5.885x_4(x_1 + x_3)}{x_1 + \sqrt{|x_3^2 - x_2^2|}}$$

Subject to

$$g_1(x) = -x_4x_2\left(0.4x_1 + \frac{x_3}{6}\right) + 8.94\left(x_1 + \sqrt{|x_3^2 - x_2^2|}\right) \leq 0$$

$$g_2(x) = -x_4x_2^2\left(0.3x_1 + \frac{x_3}{12}\right) + 2.2\left(8.94\left(x_1 + \sqrt{|x_3^2 - x_2^2|}\right)\right)^{\frac{4}{3}} \leq 0$$

$$g_3(x) = -x_4 + 0.0156x_1 + 0.15 \leq 0$$

$$g_4(x) = -x_4 + 0.0156x_3 + 0.15 \leq 0$$

$$g_5(x) = -x_4 + 1.05 \leq 0$$

$$g_6(x) = -x_3 + x_2 \leq 0$$

where,

$$0 \leq x_1, x_2, x_3 \leq 1000 \leq x_4 \leq 5$$
